# Supplementary figures and images for: Evaluation of “Caserotek” a low cost and effective artificial blood-feeding device for mosquitoes
Source: PLoS Negl Trop Dis. 2023 Aug 25;17(8):e0011563. doi: 10.1371/journal.pntd.0011563 (PMC10484425; doi:10.1371/journal.pntd.0011563)

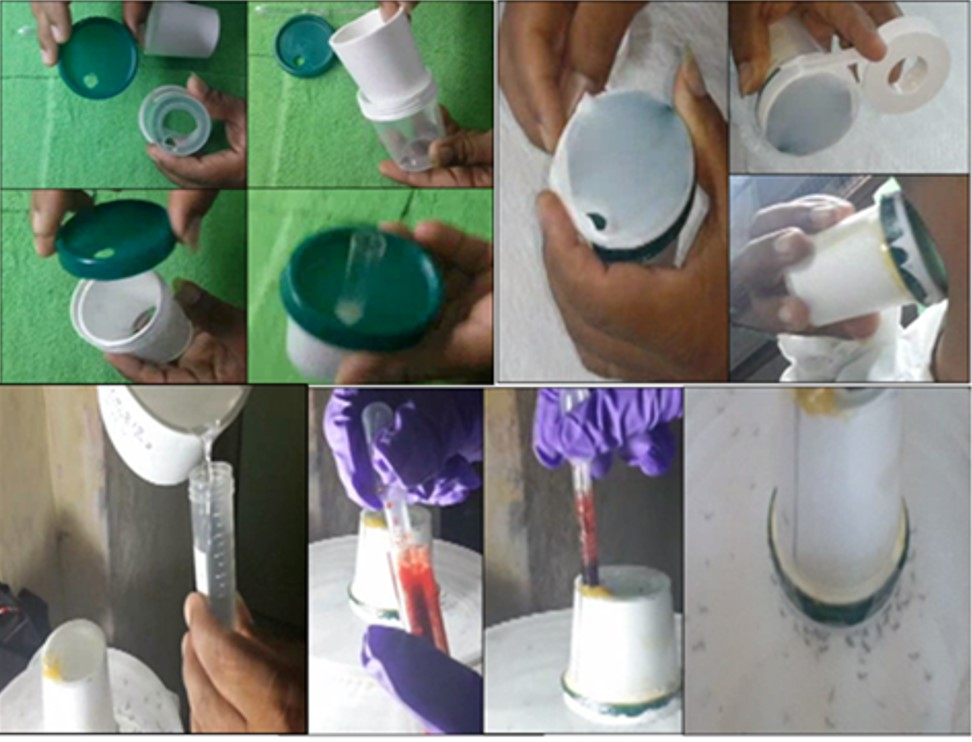

Supplement: S1 Fig — (TIF) [file pntd.0011563.s001.tif]

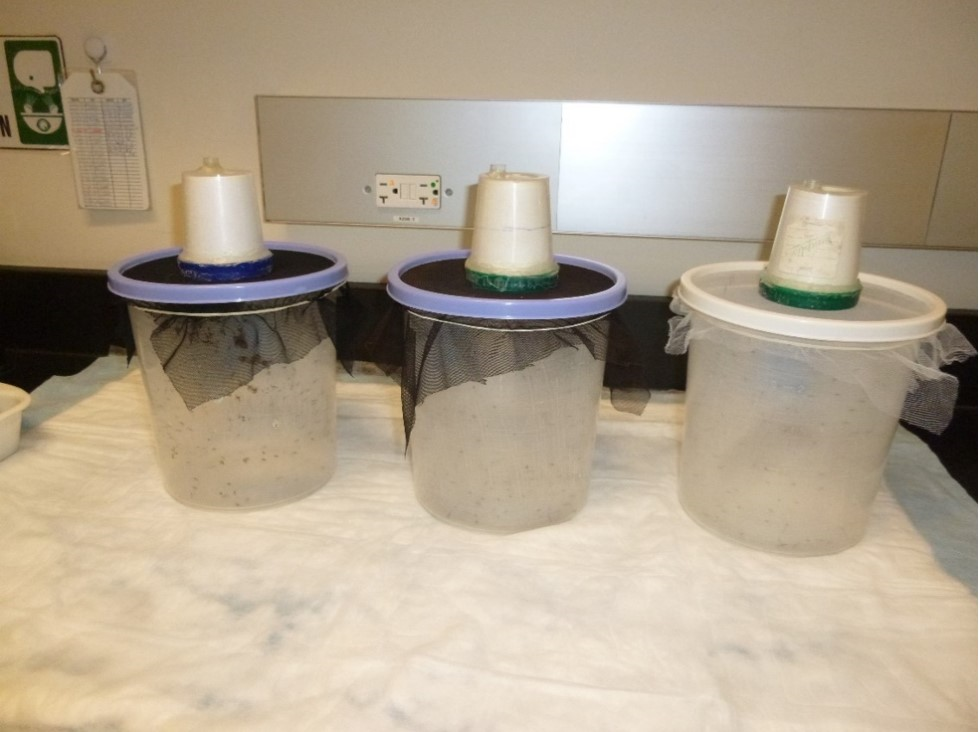

Supplement: S2 Fig — (TIF) [file pntd.0011563.s002.tif]

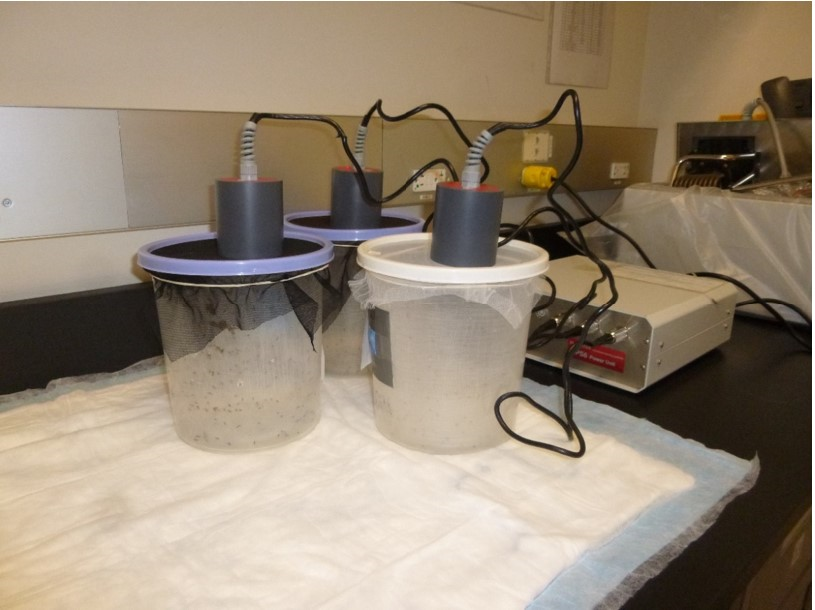

Supplement: S3 Fig — (TIF) [file pntd.0011563.s003.tif]

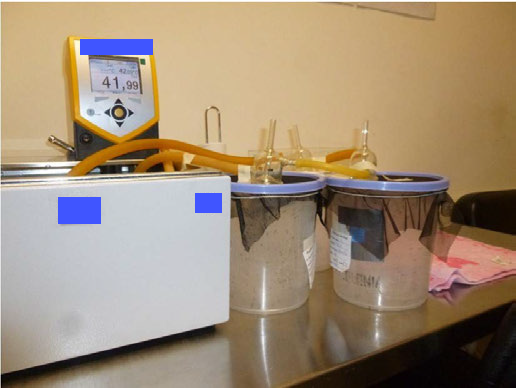

Supplement: S4 Fig — (TIF) [file pntd.0011563.s004.tif]

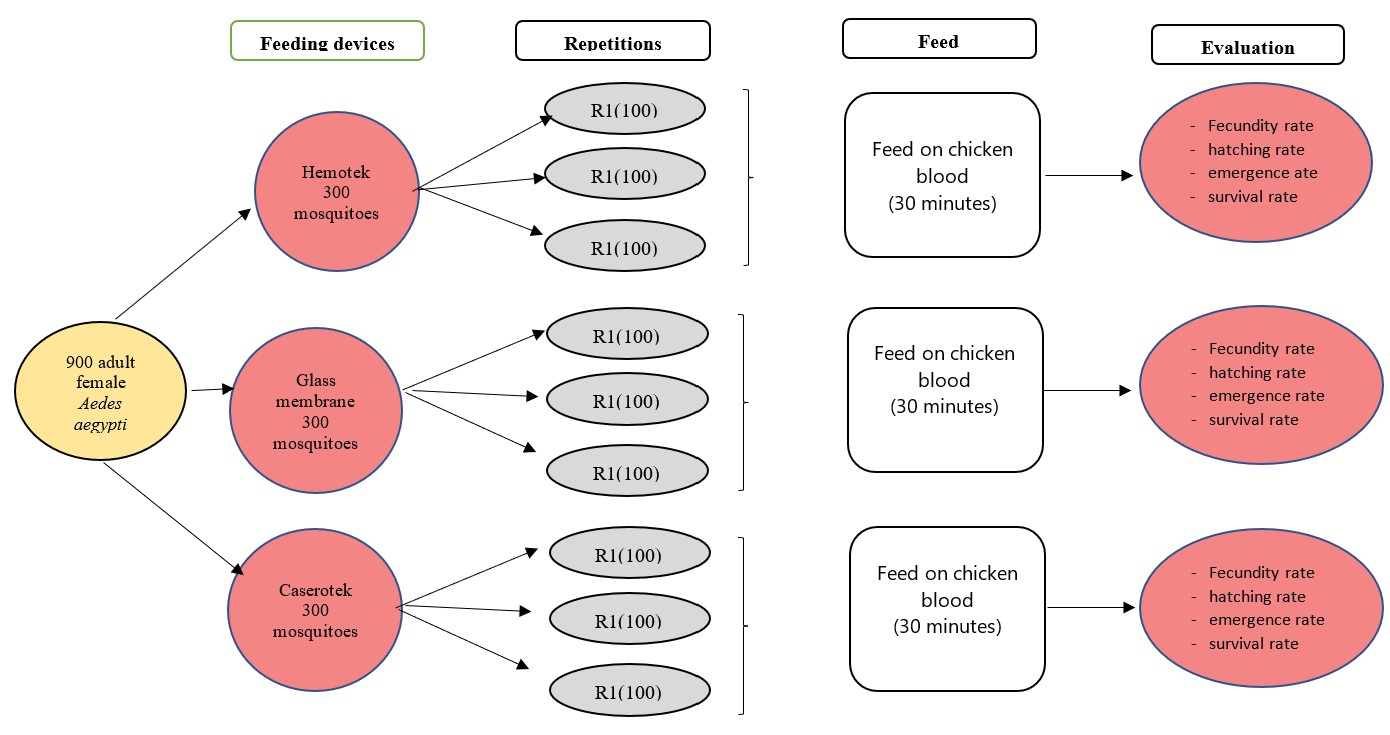

Supplement: S5 Fig — (JPG) [file pntd.0011563.s005.jpg]

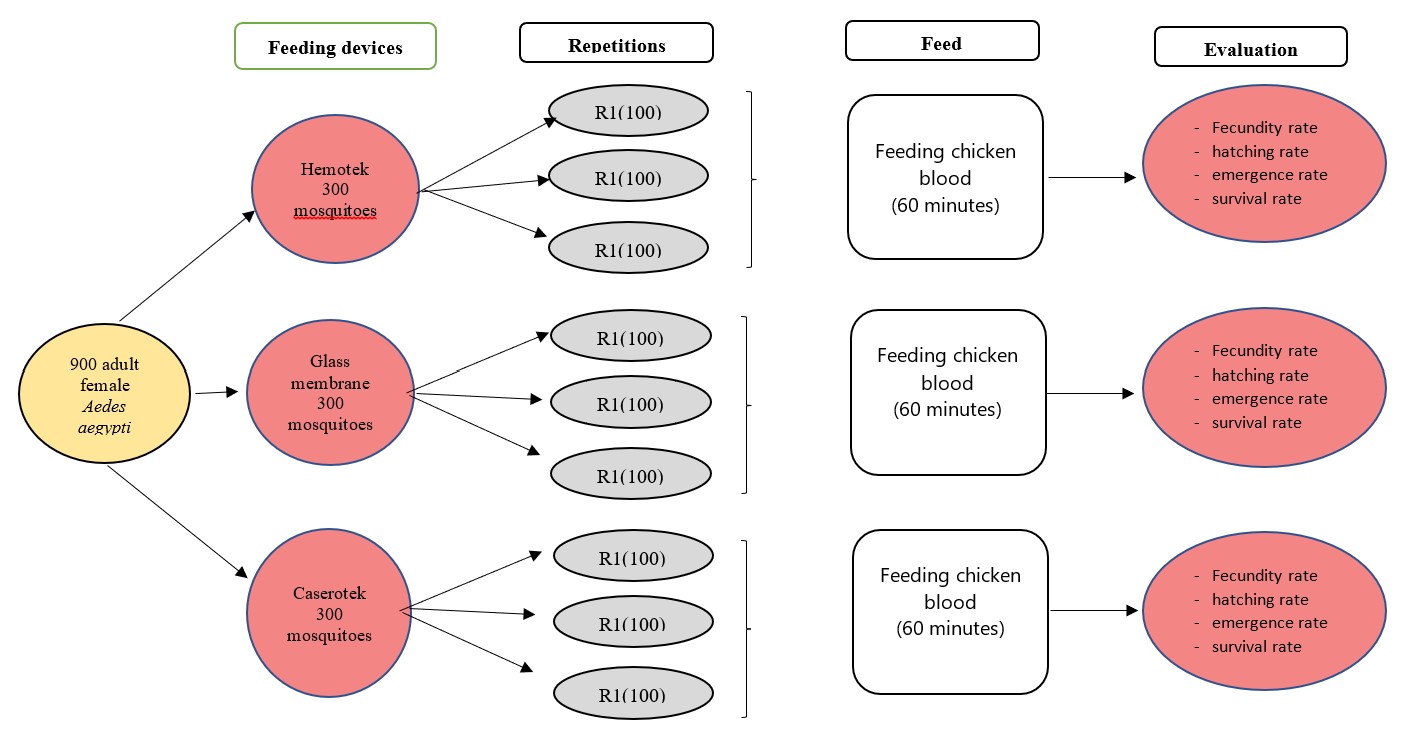

Supplement: S6 Fig — (JPG) [file pntd.0011563.s006.jpg]

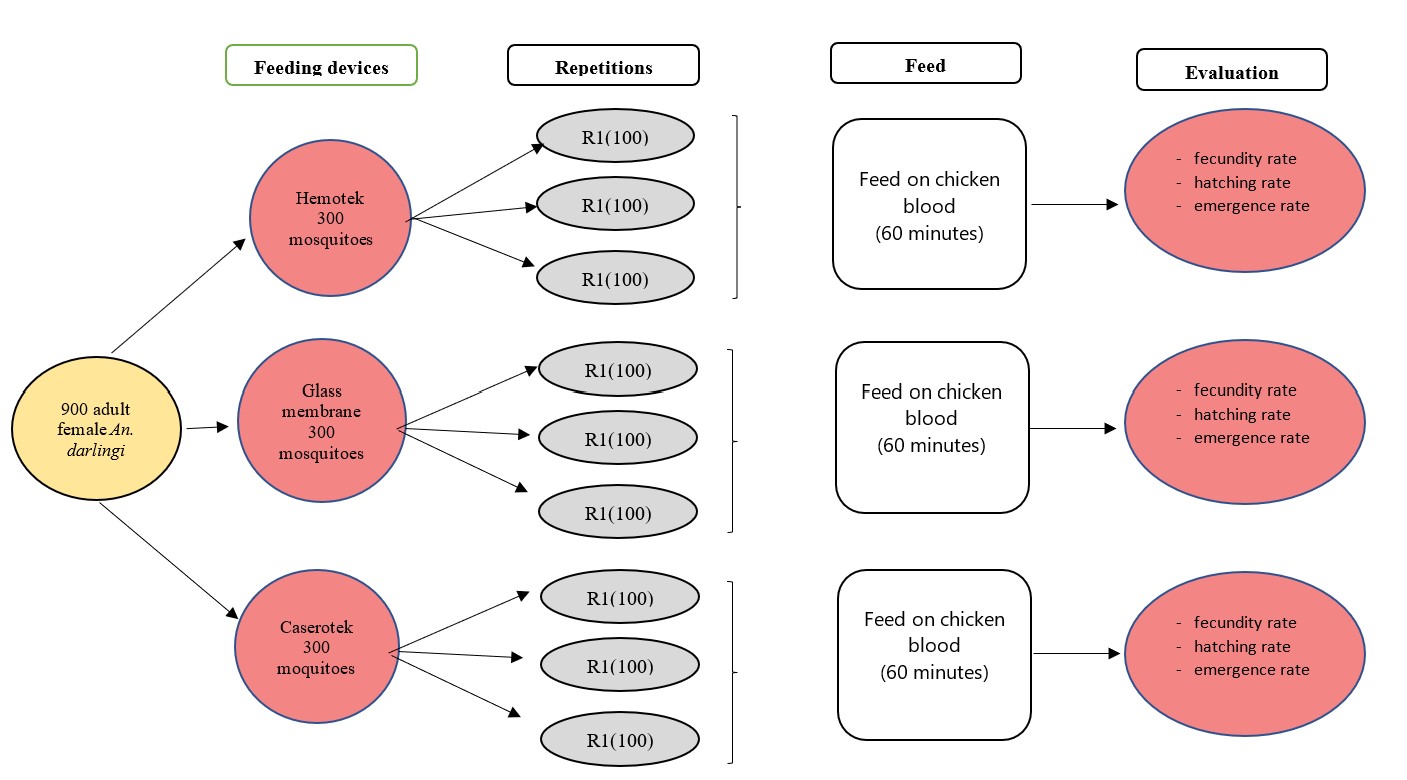

Supplement: S7 Fig — (JPG) [file pntd.0011563.s007.jpg]

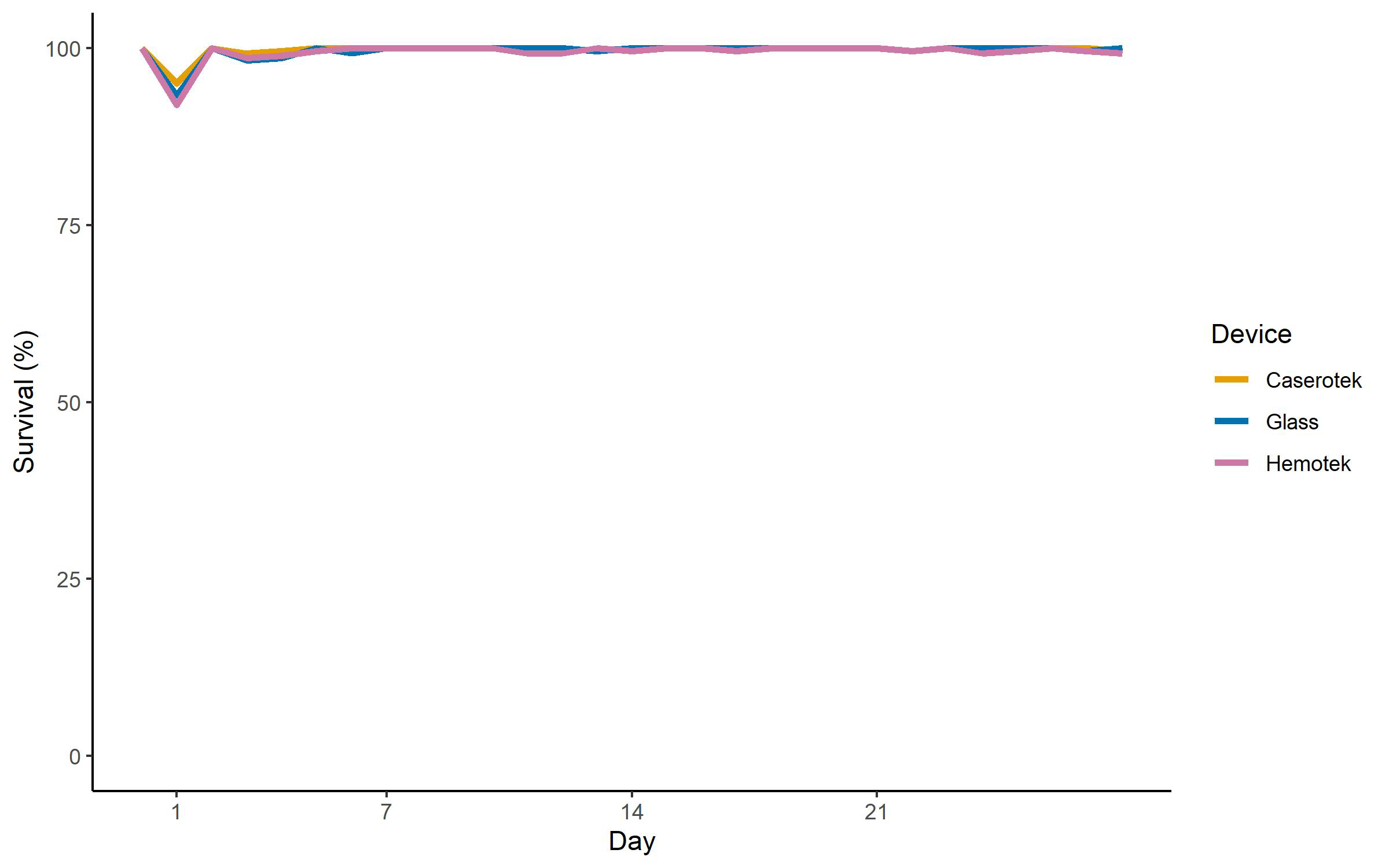

Supplement: S8 Fig — (TIFF) [file pntd.0011563.s008.tiff]
