# Supplementary material for: Evaluation of “Caserotek” a low cost and effective artificial blood-feeding device for mosquitoes
Source: PLoS Negl Trop Dis. 2023 Aug 25;17(8):e0011563. doi: 10.1371/journal.pntd.0011563 (PMC10484425; doi:10.1371/journal.pntd.0011563)
Supplement: S1 File — (PDF) [file pntd.0011563.s009.pdf]

U.S. NAVAL MEDICAL RESEARCH UNIT SIX

COLONIZATION AND MAINTENANCE OF *AEDES AEGYPTI* COLONIES

DO NOT FILL THIS SPACE	Effective Date: DO NOT FILL THIS SPACE	Version: Which corresponds	Page:1 of 13

Author: Helvio Astete/Victor López-Sifuentes
Department Head: LCDR Ryan Larson
Affected: Entomology

1. PURPOSE

Describes the process of *Aedes aegypti* adult production at the laboratory with the purpose to support entomological research studies.

2. SCOPE

These procedures apply to personnel working in the Entomology Department Lima and Iquitos.

3. GENERALCONSIDERATIONS

a) PPE

Nitrile gloves, safety glasses and lab coats will be worn while processing specimens.

b) ASEPTIC TECHNIQUES

- Discarded water from trays and cups with eggs/larvae or pupae must be boiled before discard through a drain
- Sugar pads removed from the adult cages must be washed with water tap and then disposed in the trash
- Any accidental release within the procedure room or environmental room must be immediately addressed using the prokopack aspirator or killing all released mosquitoes using the Electric LED bug fly mosquito's zapper.
- Larval trays must be covered with mesh.
- One strain of *Aedes aegypti* must be reared per room

c) UNIVERSAL PRECAUTIONS

- All personnel trained for performing this procedure must have completed the Hepatitis B vaccine schedule in place recommended by Occupational Health Program.
- Occupational Health Program recommend that all personnel handling chicken blood must have received vaccine for Influenza and TDaP (Diptheria, Tetanus and Pertusis) and be involved in the Program of Animal use.

- During mosquitoes feeding process with blood (chicken blood), personnel working in the lab must use a lab coat, closed toe shoes, gloves and safety glasses as protection.
- All contaminated material must be disposed in biohazardous waste containers.
- Following Standard operations procedures (SOP) when a laboratory accident has occurred during the execution and then report it to Laboratory command line in the act.

d) COMPLICATIONS

- If infectious material (chicken blood) splashed on your eyes or mucus membranes, wash eyes for at least 10 minutes with plenty of water, raising upper and lower eyelids. Immediately contact your supervisor and the Occupational Health Officer to inform him/her about what happened.
- Supervisor must be informed to record the mishap and a mishap report must be filled out and submitted.

4. DEFINITIONS

- SOP STANDARD OPERATION PROCEDURES
- PPE PERSONNEL PROTECTION EQUIPMENT
- mL MILILITERS
- µL MICROLITERS
- mg MILLIGRAMS
- oz OUNCE
- cm CENTIMETER
- ºC CELSIUS

5. RESPONSIBILITIES

a) Head, Department

The Head of Department is responsible for:

- Establishing and reviewing this procedure.
- Assuring to provide specific training to personnel in charge.

- Determine PPE requirements in the laboratory, in consultation with the Occupational Health Officer and the Biosafety specialists.
- Update this SOP annually or as needed.

b) Laboratory Supervisor

The Laboratory Supervisor is responsible for:

- Coordinate all logistical requirements (supply requests, receiving, etc.) to ensure a consistent and adequate supply of PPE.
- Implementing this SOP and ensuring that all personnel are trained.
- Assuring that the current version of the SOP is being used by laboratory personnel and technicians.
- Assuring the laboratory personnel receive training before starting the procedures.
- Evaluating personnel performance.

c) Laboratory personnel

The laboratory personnel are responsible for:

- Understanding this SOP prior to performing the procedures described.
- Demanding training in case they have not developed appropriate working skills yet.
- Reviewing this SOP each time before starting to work.
- Review this SOP annually and sign it.

6. PROCEDURE

a) Material

- Bucket (4 L) or cage 45cm^3
- Bucket (2.5 L) or cage 30cm^3
- Metal rack
- Trays (26x36 cm)
- Transfer pipette, 3mL
- Ovitraps (Plastic cups for oviposition, 4 oz)
- Plastic container (1, 1.5L)
- Towel paper
- Aspirator filter

- Ziploc bag (3x5x0.002 inch)
- Plastic container (85mm X 64mm)
- Plastic or glass tube (75mm)
- Styrofoam (8 ounces)
- Teflon film (1 inch)
- Cotton balls
- Mouth aspirator
- Sleeves (Bio Quip 1452A)
- Paper towel
- Rubber
- Parafilm
- Gloves
- Safety glasses
- Biosafety bags
- Biosafety containers
- Thermos for blood sample
- Lab coat
- Notebook
- Plastic bottle (2.5 L)

b) Equipment

- Hemotek feeding artificial System
- Caserotek feeding artificial System
- Thermo-hygrometer
- Refrigerator (5-7 ºC)
- Thermometer
- Electric LED bug fly mosquito's zapper
- Prokopack
- Stinger indoor insect trap
- Battery 12V
- Stereoscope

c) Biological Material

- *Aedes aegypti* strains (Rockefeller , New Orleans, wild)
- Chicken blood

d) Reagents
- Sodium hypochlorite (5%)
- Sodium heparin tubes
- Ethanol 70%

e) Others

- 10% hay Infusion
- 10% sugared solution
- Cinnamon and clove tea (1.2 gr)
- Food (Super cat, mix of flour [wheat + fish], rodent food)
- Distilled water/ tape water/filtered water

f) Quality Control

N/A

g) Methodology

1. <u>Eggs obtaining and storing</u>

- In the laboratory the strains were maintained, placing from 400 to 500 adults in a bucket (4L) or cages of ($30cm^3$) 500 to 1000 adults and in cages of ($45cm^3$) 1000 to 3000 adults were placed until mating, and kept in an adult room. In strains of wild origin, the number of adults may differ, since it depends on the specimens collected in the field (larvae, eggs, adult).
- 3-4-days is waited for what mosquitoes (females/males) can mate into bucket, during this time period, adults only receive sugared solution (10%) soaked in cotton as meal, every two days this sugared solution is replaced for another fresh.
- After 4-5 days, adults into cage or bucket are prepared, meal is removed (6-16hours) to then provide them a blood feeding, it is provided chicken blood through the Hemotek artificial feeding system (SOP for Hemotek artificial feeding system), a feeder with blood is put on the screened bucket top for a time of 45 -60 minutes, then it is provided sugared solution (10%) to complement the feed until they can eggs lay.
- Caserotek.The device uses a plastic urine collection container (85mm x 64mm). A plastic tube (75mm) is run through the base of the cup flush with the lid, so that blood will flow to the space between the top of the lid an the 10mm lid lip. Two holes are cut in the base of the cup to add hot water (18mm) and to run the tube for blood (10mm). The interior of the cup is lined with an 8 oz Styrofoam from a coffee cup.

- A Teflon film (plumbers' tape) covers the elevated lip of the lid allowing a narrow space between the lid top and the Teflon film. Mosquitos feed easily through the film. Hot water is placed in the cup, (40^0C) then the blood added to the tube (2ml). Hot water is

replaced at 20-minute intervals. Prior to feeding we rubbed the membrane area on sweat of our laboratory technician, which aided in stimulating feeding.

- Two or three feeders are placed on top of each cage or bucket for 45-60 minutes, then sugar water (10%) is placed inside the cages.
- 1-day after feeding, two ovitraps (plastic cup 4 oz) are placed in the cage or bucket, and then are let for 2- 6 days to getting females eggs lay. The ovitraps are prepared previously, this is done placing 70 mL filtered water in a plastic cup 4 oz, then a filter paper strip (5 cm) is lined on inner of cup in contact with water, so that the paper is always moist, where females lay their eggs.
- The ovitraps are took out from cages or buckets and placed on a metal rack inside room, then the filter strips with eggs are transferred to a dry towel paper conditioned in a tray, these strips are left for 2-day for their dried.
- Strips eggs number are accounted under a stereoscope, a way to estimate the strip eggs total number is to measure a 1cm of strip, to count the eggs number at this measure and then multiply it for the strip total measure.
- Once eggs are accounted, to bend the strip in 4 parts for the eggs side, and then put it in plastic Ziploc bag (3x5x0.002 inch), it is can put varies strips in the bag, but it has to be of same generation.
- Each bag with eggs must be labeled with the generation number, number of eggs, date of collection, date of storage and name of the strain.
- Labeled eggs bags are placed in a plastic container (1, 1.5 L) closed hermetically and left in environment on a metal rack. Also, the eggs can be stored into a refrigerator. Eggs stored both to environment or refrigerator have a time of viability, this can be between 3-12 months, it depends of storage conditions, stored eggs to environment are better stored that who's placed in a refrigerator.
- Appropriate temperature and humidity conditions inside room to eggs production and storing should range 26-28 ºC and 60-70 %.

2. <u>Larvae hatching</u>

- For the hatching of dry-eggs it is necessary to stimulate them in 2 ways: these can be done placing the eggs in warm infusions (35-40 º C). Infusions can be prepared previously and be stored to be used every time is required; one of laboratory-used regularly infusions are prepared by doing to boil (10 minutes) a grass brought from field, then this infusion is stored in a plastic bottle.
- Prior to placing the eggs strips for their hatching, the infusion (the quantity should be calculated) is mixed (60:40) with filtered water and

then warmed in a microwave for 1.5 minutes, by using a thermometer adjust temperature 35-40ºC, this warmed water is placed in a tray (26x36 cm, then the eggs strips, it is necessary that eggs staying on water surface and not go to bottom of tray, after 1 hour can be observed the larvae hatching, and after 3 hours 80-90% should have hatched.

- The 2nd way to hatch the eggs, place 1 liter of hot filtered water (40^0C) in a tray (30cm x 22 cm), add 2 sachets of cinnamon tea and cloves then immerse the paper towel strips containing *Ae.aegypti* eggs. up to 1-24 hours.
- In the case eggs are not hatching, repeat the same process with same eggs batch, sometimes need to do stimulated more than one time.
- Before placing the eggs to hatching them, it is necessary to verify the eggs viability under a stereoscope, this will allow us to observe the eggs status (physical characteristic, opened operculum, turgor) and then to calculate the number to be hatched.
- Larvae hatched do not need to be fed at least until 24 hours.

3. <u>Larval development and pupae collection</u>

- 2-days old Larvae at stage L1 are transferred to other trays (30x35 cm) to a density of 200 to 300 for each tray, a volume of 1 a 1.5 L filtered water is added to each tray; this is an adequate volume to larval development.
- Larvae are fed with ground rodent food and sifted (150 um), the food proportion is calculated for 200 Larvae (adapted of Gerberg et al. 1994), it is as follow: Day1= 40 mg, Day 2= 60 mg, Day 3= 80 mg, Day 4- 7 = 120 mg.
- The food is provided manually to the larvae through a transfer pipette (3mL), the food is provided as solution (the ground rodent food is diluted in filtered water, the quantity should be calculated according to larva number in rearing), the frequency of feeding is as follow: once day (stage L1-L2), two-time day (stage L3-L4).
- After 6-day it is started the pupation, this can range 6-8 days.
- Collections of pupae is manually performed through a transfer pipette (3 mL), 200-250 pupae are placed in plastic cups (4 oz) with 80 mL filtered water, and then pupae are put into a screened bucket (4L) to the emergence of adult mosquitoes, which happens between 1-2 days. Up to 500 pupae can be placed in a bucket. Up to 3000 pupae can be placed in cages (45 cm^3).
- If you need only female, sort the female pupae according to size; female pupae are bigger than male.
- Temperature and humidity inside the larvae room should be ranged 26-28 ºC and 60-80%, and water temperature should be ranged 25-27 ºC.

- The food of larvae always must be stored into a refrigerator (5 to 7 ºC), this allow to maintain the feed quality and to avoid the contamination.

4. Adult maintenance
   - After the emergence of the adults (2 days) in the buckets, provide them with a sugar solution (10%) soaked in cotton balls and place them in buckets with mesh, in the case of the cages, cotton balls soaked with sugar (10%) They are placed inside the cages in the form of lighters. The cottons must be changed every two days for new ones.
   - Adults produced to continue a new generation is kept at the same cages or buckets, and then to follow the same procedure to egg production described in the first part of the Methodology.
   - Females' adults produced for the experiments are transferred to buckets (2.5 L) to maintain them alive, soaked cotton balls with sugared solution (10 %) are provided, every 2-days cottons are changed for other new until the mosquitoes are used at the experiments.
   - Temperature and humidity inside the adult room should be ranged 26-28 ºC and 60-80%.

5. Management schedule for *Aedes aegypti* strains

Management schedule for *Aedes aegypti* strains

DATE	STRAINS	GENERATION	# EGGS TO BE FLOODED	PURPOSE	OBSERVATIONS

h) Results

The data on the colonies are recorded in 2 books, book 1 the information is recorded from the date of flooding of eggs, number of hatching of larvae, pupae, adults and percentage of females fed on Aedes aegypti. Book 2 records the number of eggs for each strain, then all the information is entered into the database.

i) Interpretation

N/A

7. REFERENCES

- Gerberg EJ, Barnard DR, Ward RA. 1994. Manual for mosquito rearing and experimental techniques. Am Mosq Contr Assoc; 5:98.
- SOP- Hemotek feeding System for mosquitoes
- USAID.2019. Manual Práctico: Procedimientos de cría de la especie *Aedes aegypti* y principios básicos de Bioseguridad.

8. ATTACHMENTS

Artificial feeding system

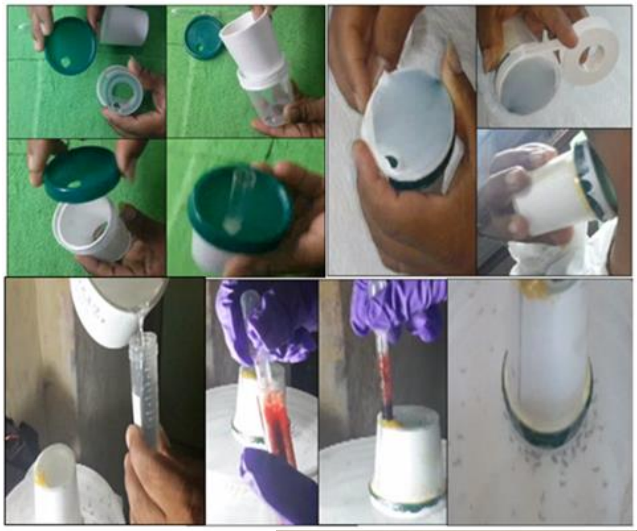

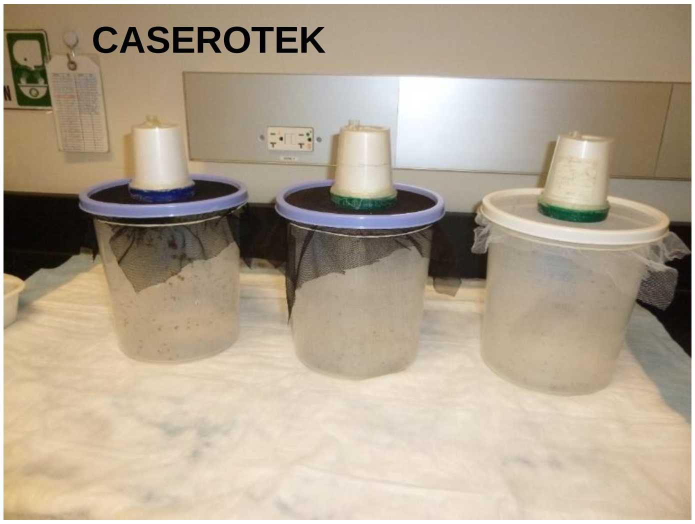

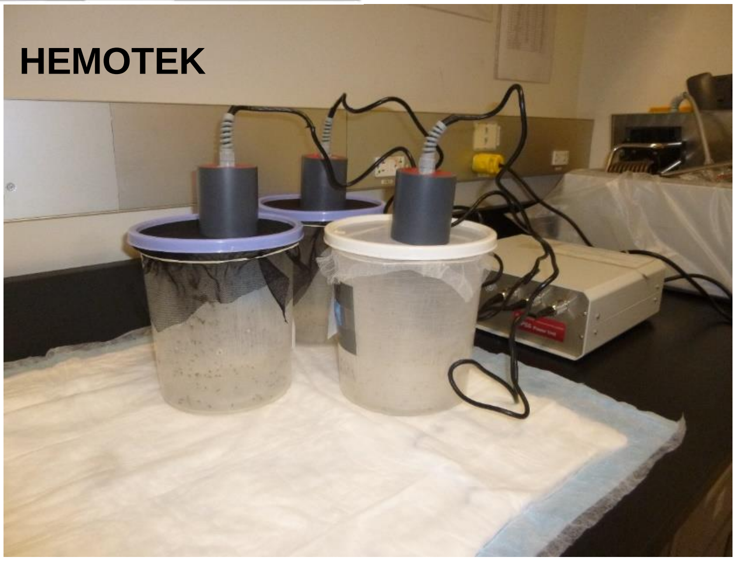

Ovitraps

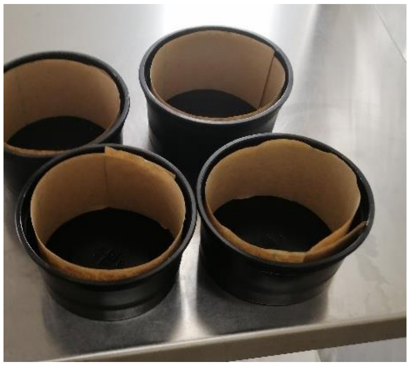

egg drying

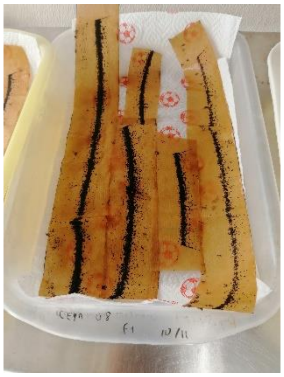

Hatching of eggs

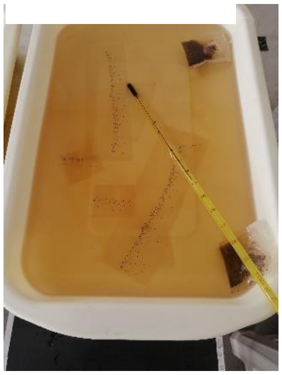

Larvae count

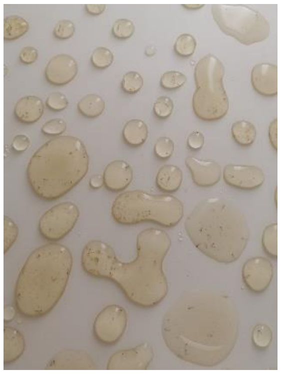

Larvae

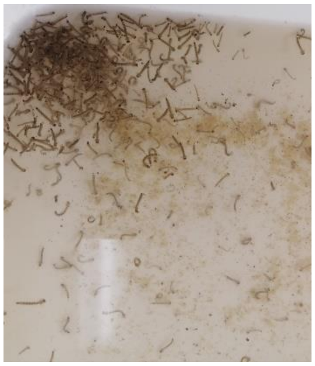

Pupae

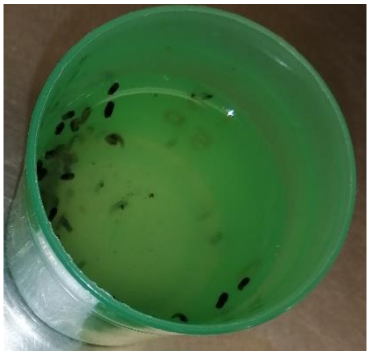

Aedes aegypti Adults

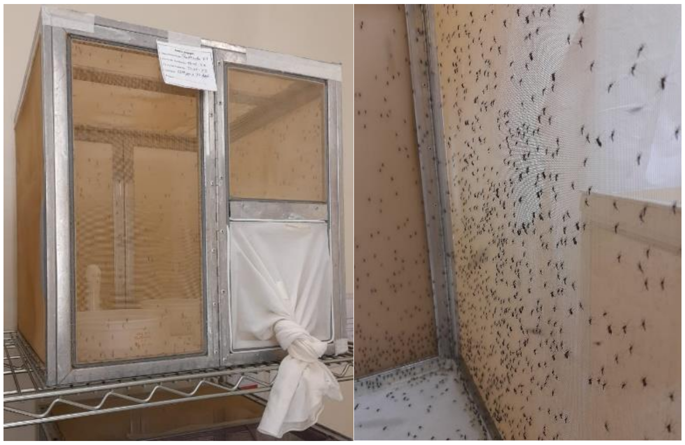

9. REVISION REVIEWED AND APPROVED BY

(LCDR Ryan Larson) DATE
HEAD, DEPARTMENT OF ENTOMOLOGY

______________________________ ____________

(Victor M. López-Sifuentes) DATE
LABORATORY SUPERVISOR

IMPLEMENTED BY

DO NOT FILL THIS SPACE

______________________________ ____________

(Victor M. López-Sifuentes) DATE

ANNUAL SOP REVIEW LOG:

BY:______________________________DATE:____________
BY:______________________________DATE:____________
BY:______________________________DATE:____________
BY:______________________________DATE:____________
BY:______________________________DATE:____________
BY:______________________________DATE:____________
BY:______________________________DATE:____________
BY:______________________________DATE:____________
BY:______________________________DATE:____________
BY:______________________________DATE:____________
BY:______________________________DATE:____________
BY:______________________________DATE:____________

10. REVISION HISTORY

VERSION	BRIEF GENERAL DESCRIPTION AND JUSTIFICATION OF CHANGES	EFFECTIVE DATE
A	NEW SOP	DO NOT FILL THIS SPACE
B	Third version. Some changes was performed at the procedures and edited to new version of SOP template.	DO NOT FILL THIS SPACE
C		DO NOT FILL THIS SPACE
D		DO NOT FILL THIS SPACE
E		DO NOT FILL THIS SPACE
