# Supplementary material for: Evaluation of “Caserotek” a low cost and effective artificial blood-feeding device for mosquitoes
Source: PLoS Negl Trop Dis. 2023 Aug 25;17(8):e0011563. doi: 10.1371/journal.pntd.0011563 (PMC10484425; doi:10.1371/journal.pntd.0011563)
Supplement: S2 File — (PDF) [file pntd.0011563.s010.pdf]

Experiment #1 General Linear Models (GLM) for:

1. Feeding Rate
    a. Replicates with <10 mosquitoes removed.
    b. All data points included.
2. Egg production
    a. Replicates with <10 mosquitoes removed.
    b. All data points included.
3. Survival
    a. Replicates with <10 mosquitoes removed.
    b. All data points included.

The study design for Experiment #1 eliminated mosquitoes did not feed after 30 minutes resulting in some replicates with < 10 mosquitoes. Elimination of these data points had no impact on the statistical significance of differences between blood feeding device or time or the device*time interaction term. We present our SAS code used to analyze S1 Data File followed by model output directly from SAS.

Feeding Rate

```
/*Engorgement rates Experiment #1*/
/*Import S1_File_July2023 Sheet Feed$*/

DATA EXP1_FEED;
SET FEED;
if EXP = 1;
run;
```

Replicates with <10 mosquitoes removed.

```
ODS RTF FILE='Feedmodel.RTF';
PROC GLM DATA=EXP1_FEED;
WHERE include=1;
CLASS  Day Repeat Device feedrate;
MODEL Feedrate =  Day Device day*device /SS3;
OUTPUT OUT=R RESIDUAL = RES;
LSMEANS DAY / STDERR PDIFF TDIFF;
*LSMEANS SITE*TYPE / STDERR PDIFF TDIFF;
LSMEANS DEVICE / STDERR PDIFF TDIFF;
LSMEANS Day*Device / STDERR PDIFF TDIFF;

PROC UNIVARIATE NORMAL PLOT DATA=R; VAR RES; RUN;

proc means data=feed mean std stderr;
var Feedrate;
class day device;
run;
ODS RTF CLOSE;
```

All data points included.

```
ODS RTF FILE='Feedmodel_alldata.RTF';
PROC GLM DATA=EXP1_FEED;
CLASS  Day Repeat Device feedrate;
MODEL Feedrate =  Day Device day*device /SS3;
OUTPUT OUT=R RESIDUAL = RES;
LSMEANS DAY / STDERR PDIFF TDIFF;
*LSMEANS SITE*TYPE / STDERR PDIFF TDIFF;
LSMEANS DEVICE / STDERR PDIFF TDIFF;
LSMEANS Day*Device / STDERR PDIFF TDIFF;

PROC UNIVARIATE NORMAL PLOT DATA=R; VAR RES; RUN;

proc means data=feed mean std stderr;
var Feedrate;
class day device;
run;
ODS RTF CLOSE;
```

The SAS System

The GLM Procedure

Class Level Information		
**Class**	**Levels**	**Values**
**Day**	3	1 7 14
**Repeat**	3	A B C
**Device**	3	casero glass hemo
**feedrate**	22	2 17 18 32 36 57.142857143 64.285714286 64.516129032 67 74.285714286 77.142857143 80 85.714285714 88.571428571 90.909090909 92.424242424 92.708333333 92.783505155 93.939393939 94.186046512 99 100

**Number of Observations Read**	25
**Number of Observations Used**	25

The SAS System

The GLM Procedure

Dependent Variable: feedrate feedrate

Source	DF	Sum of Squares	Mean Square	F Value	Pr > F
**Model**	8	18193.28668	2274.16083	19.38	<.0001
**Error**	16	1877.84033	117.36502		
**Corrected Total**	24	20071.12701			

R-Square	Coeff Var	Root MSE	feedrate Mean
0.906441	15.94602	10.83351	67.93867

Source	DF	Type III SS	Mean Square	F Value	Pr > F
**Day**	2	6603.476612	3301.738306	28.13	<.0001
**Device**	2	7398.634816	3699.317408	31.52	<.0001
**Day*Device**	4	2936.789347	734.197337	6.26	0.0031

The SAS System

The GLM Procedure

Dependent Variable: feedrate feedrate

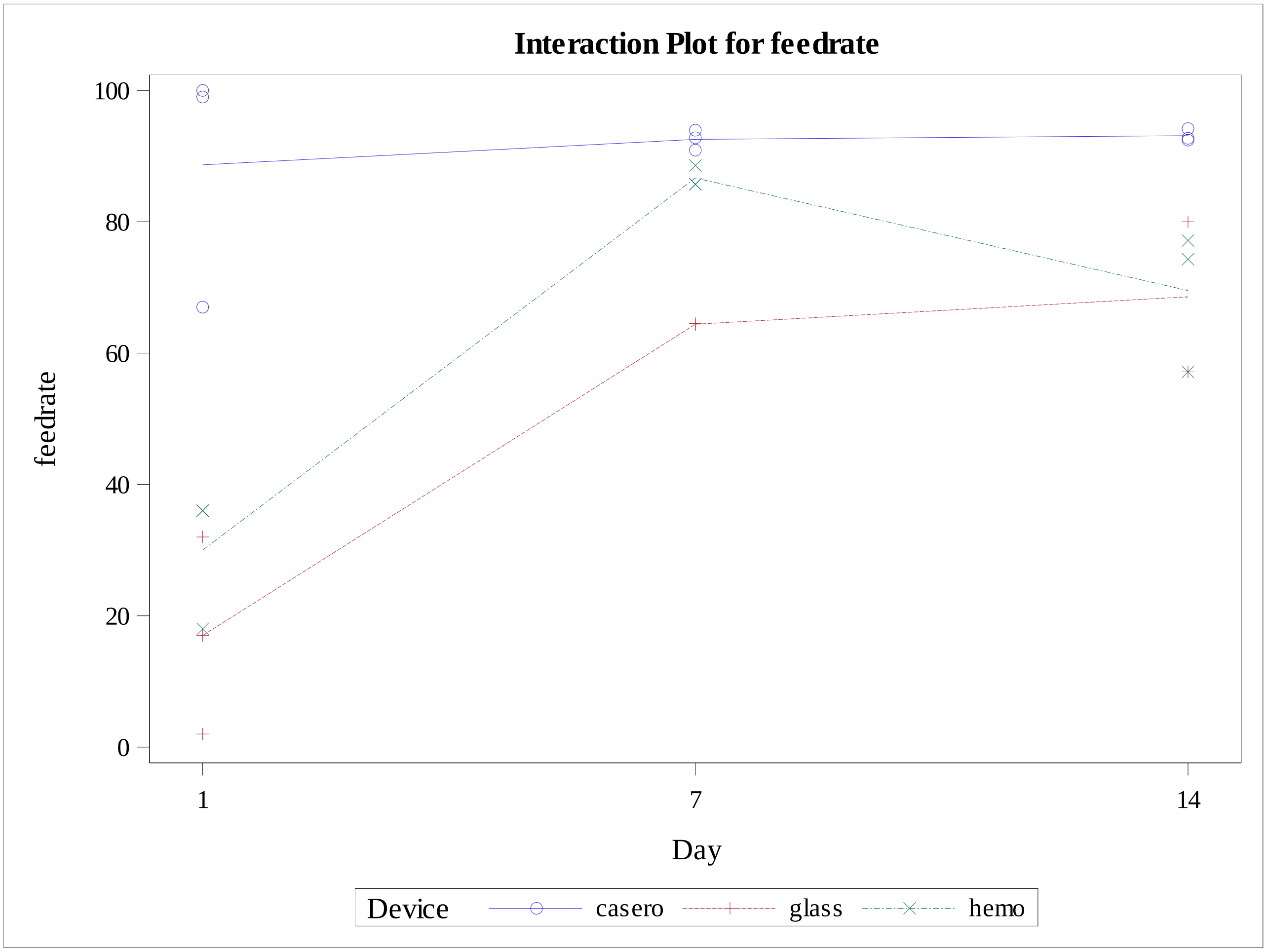

The SAS System

The GLM Procedure
Least Squares Means

Day	feedrate LSMEAN	Standard Error	Pr > |t|	LSMEAN Number
**1**	45.2222222	3.6111713	<.0001	1
**7**	81.2038617	3.9005108	<.0001	2
**14**	77.0671485	3.9005108	<.0001	3

Least Squares Means for Effect Day
t for H0: LSMean(i)=LSMean(j) / Pr > |t|

Dependent Variable: feedrate

i/j	1	2	3
**1**		-6.76919<br><.0001	-5.99096<br><.0001
**2**	6.769192<br><.0001		0.749927<br>0.4642
**3**	5.990956<br><.0001	-0.74993<br>0.4642	

The SAS System

The GLM Procedure
Least Squares Means

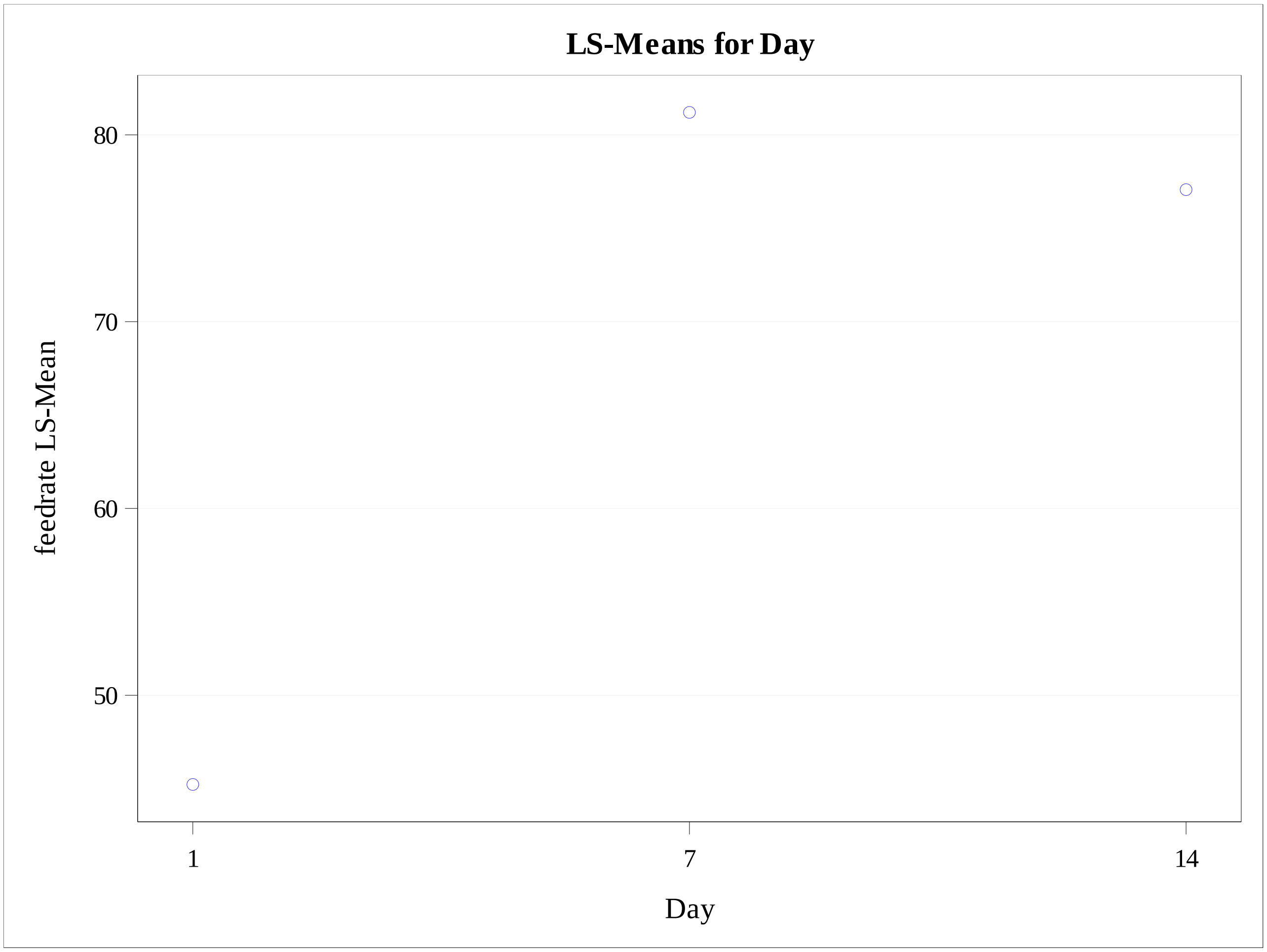

The SAS System

The GLM Procedure
Least Squares Means

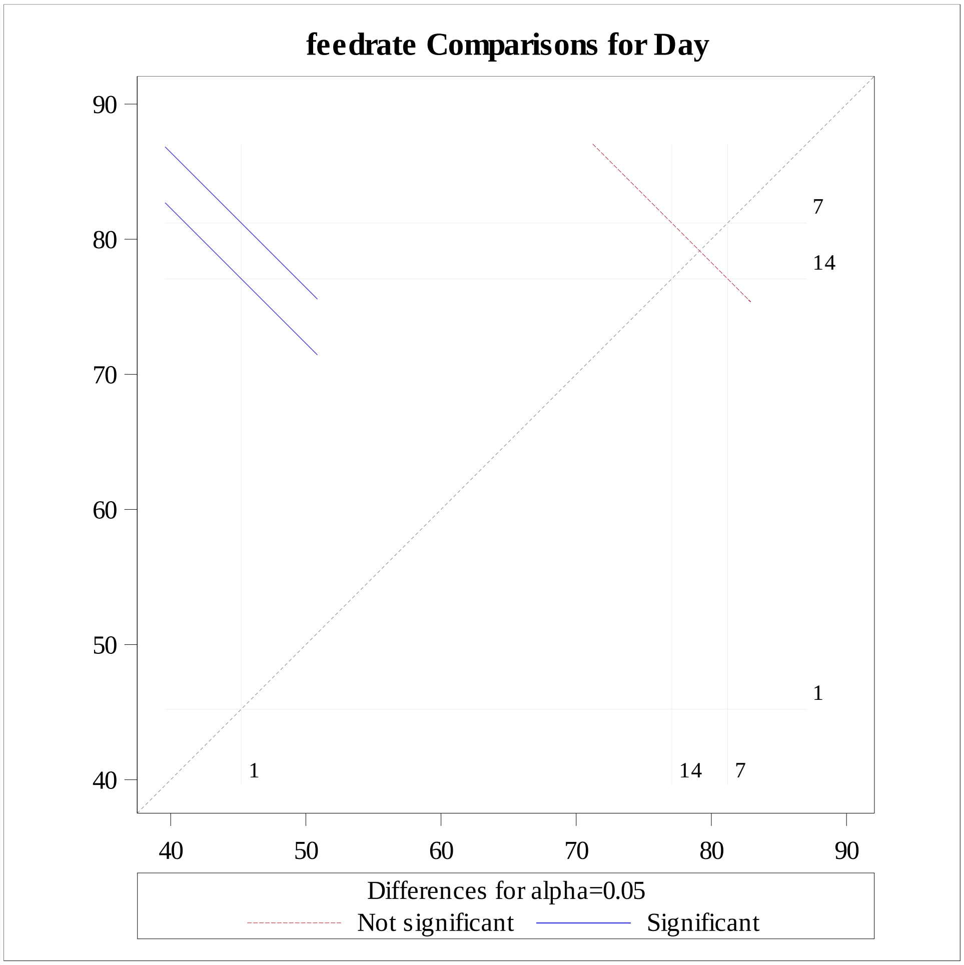

Note: To ensure overall protection level, only probabilities associated with pre-planned comparisons should be used.

The SAS System

The GLM Procedure
Least Squares Means

Device	feedrate LSMEAN	Standard Error	Pr > |t|	LSMEAN Number
casero	91.4389569	3.6111713	<.0001	1
glass	49.9907834	4.1698214	<.0001	2
hemo	62.0634921	3.6111713	<.0001	3

Least Squares Means for Effect Device
t for H0: LSMean(i)=LSMean(j) / Pr > |t|

Dependent Variable: feedrate

i/j	1	2	3
1		7.513961<br><.0001	5.752037<br><.0001
2	-7.51396<br><.0001		-2.18861<br>0.0438
3	-5.75204<br><.0001	2.188609<br>0.0438	

The SAS System

The GLM Procedure
Least Squares Means

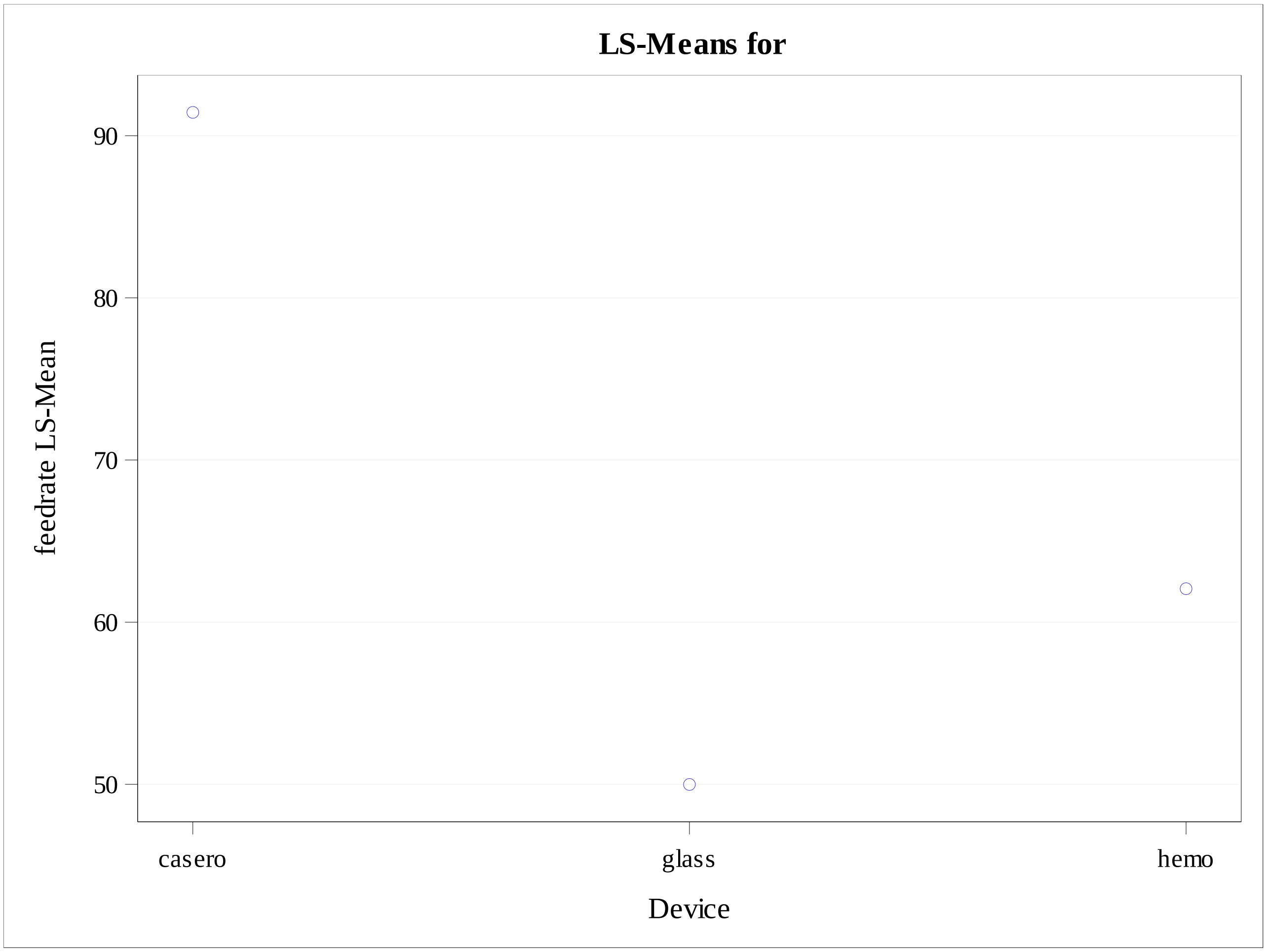

The SAS System

The GLM Procedure
Least Squares Means

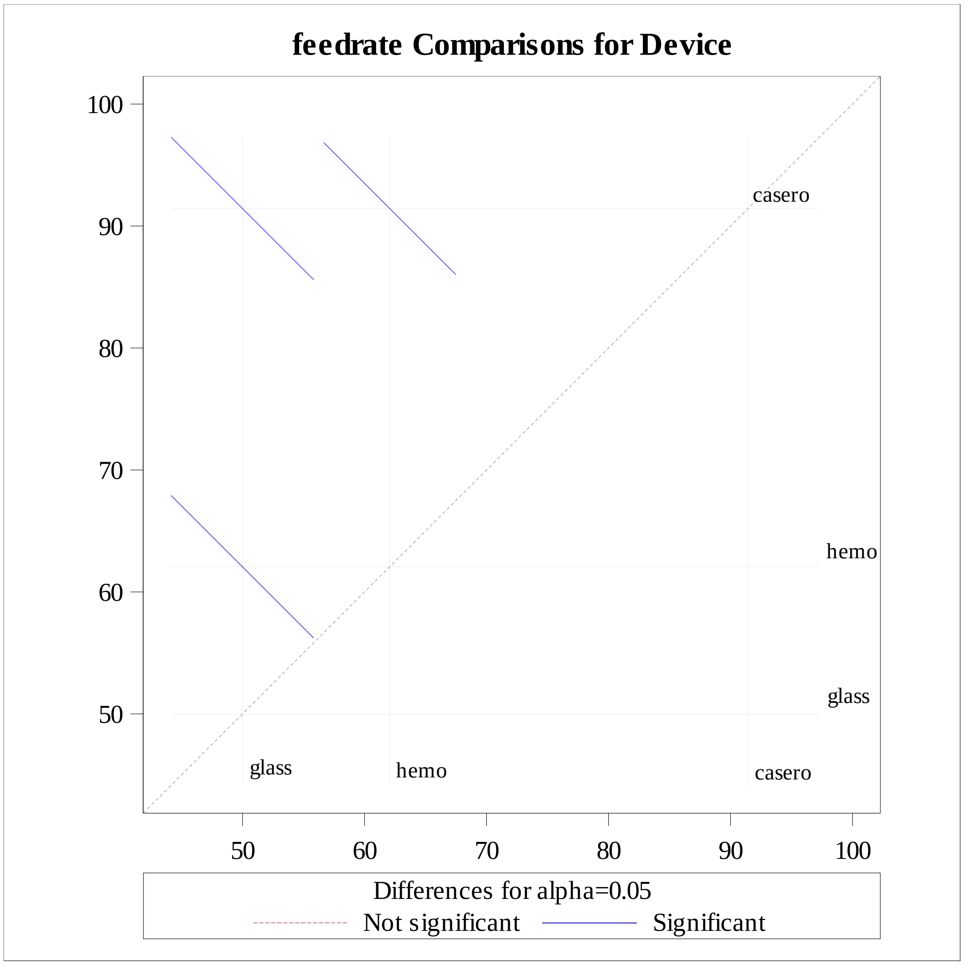

Note: To ensure overall protection level, only probabilities associated with pre-planned comparisons should be used.

The SAS System

The GLM Procedure
Least Squares Means

Day	Device	feedrate LSMEAN	Standard Error	Pr > |t|	LSMEAN Number
1	casero	88.6666667	6.2547321	<.0001	1
1	glass	17.0000000	6.2547321	0.0152	2
1	hemo	30.0000000	6.2547321	0.0002	3
7	casero	92.5439967	6.2547321	<.0001	4
7	glass	64.4009217	7.6604511	<.0001	5
7	hemo	86.6666667	6.2547321	<.0001	6
14	casero	93.1062074	6.2547321	<.0001	7
14	glass	68.5714286	7.6604511	<.0001	8
14	hemo	69.5238095	6.2547321	<.0001	9

Least Squares Means for Effect Day*Device
t for H0: LSMean(i)=LSMean(j) / Pr > |t|

Dependent Variable: feedrate

i/j	1	2	3	4	5	6	7	8	9
1		8.102023<br><.0001	6.632354<br><.0001	-0.43834<br>0.6670	2.453663<br>0.0260	0.226103<br>0.8240	-0.5019<br>0.6226	2.031957<br>0.0591	2.164129<br>0.0459
2	-8.10202<br><.0001		-1.46967<br>0.1610	-8.54036<br><.0001	-4.79301<br>0.0002	-7.87592<br><.0001	-8.60392<br><.0001	-5.21471<br><.0001	-5.93789<br><.0001
3	-6.63235<br><.0001	1.469669<br>0.1610		-7.07069<br><.0001	-3.47849<br>0.0031	-6.40625<br><.0001	-7.13425<br><.0001	-3.9002<br>0.0013	-4.46823<br>0.0004
4	0.438338<br>0.6670	8.540361<br><.0001	7.070692<br><.0001		2.845724<br>0.0117	0.664441<br>0.5159	-0.06356<br>0.9501	2.424018<br>0.0276	2.602466<br>0.0192
5	-2.45366<br>0.0260	4.793007<br>0.0002	3.478495<br>0.0031	-2.84572<br>0.0117		-2.25143<br>0.0388	-2.90257<br>0.0104	-0.38496<br>0.7053	-0.51801<br>0.6115
6	-0.2261<br>0.8240	7.87592<br><.0001	6.406251<br><.0001	-0.66444<br>0.5159	2.25143<br>0.0388		-0.728<br>0.4771	1.829724<br>0.0860	1.938026<br>0.0705
7	0.501897<br>0.6226	8.60392<br><.0001	7.134251<br><.0001	0.063559<br>0.9501	2.902573<br>0.0104	0.728<br>0.4771		2.480867<br>0.0246	2.666025<br>0.0169
8	-2.03196<br>0.0591	5.214713<br><.0001	3.900201<br>0.0013	-2.42402<br>0.0276	0.384963<br>0.7053	-1.82972<br>0.0860	-2.48087<br>0.0246		-0.0963<br>0.9245
9	-2.16413<br>0.0459	5.937895<br><.0001	4.468226<br>0.0004	-2.60247<br>0.0192	0.518008<br>0.6115	-1.93803<br>0.0705	-2.66603<br>0.0169	0.096301<br>0.9245	

The SAS System

The GLM Procedure
Least Squares Means

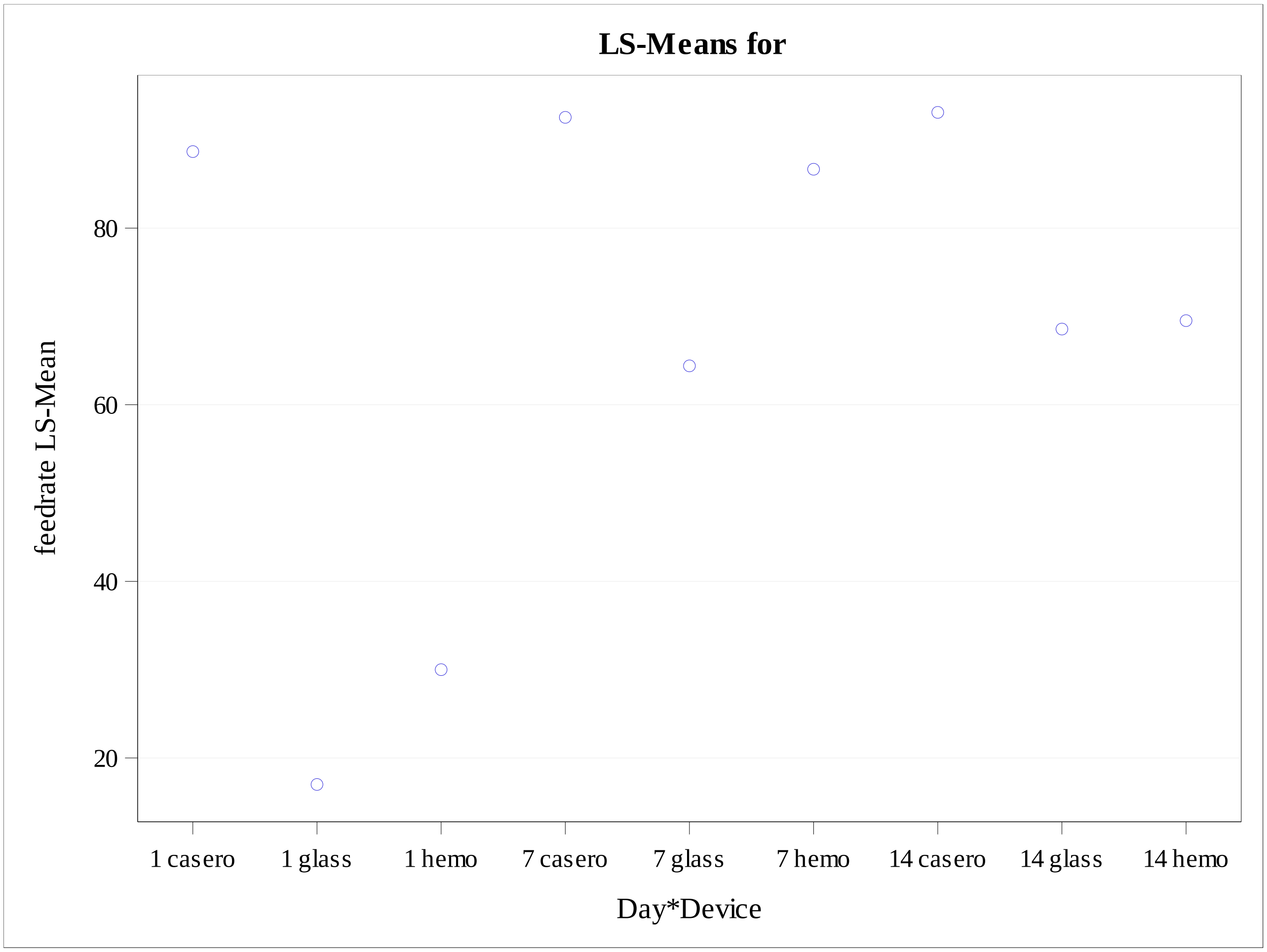

The SAS System

The GLM Procedure
Least Squares Means

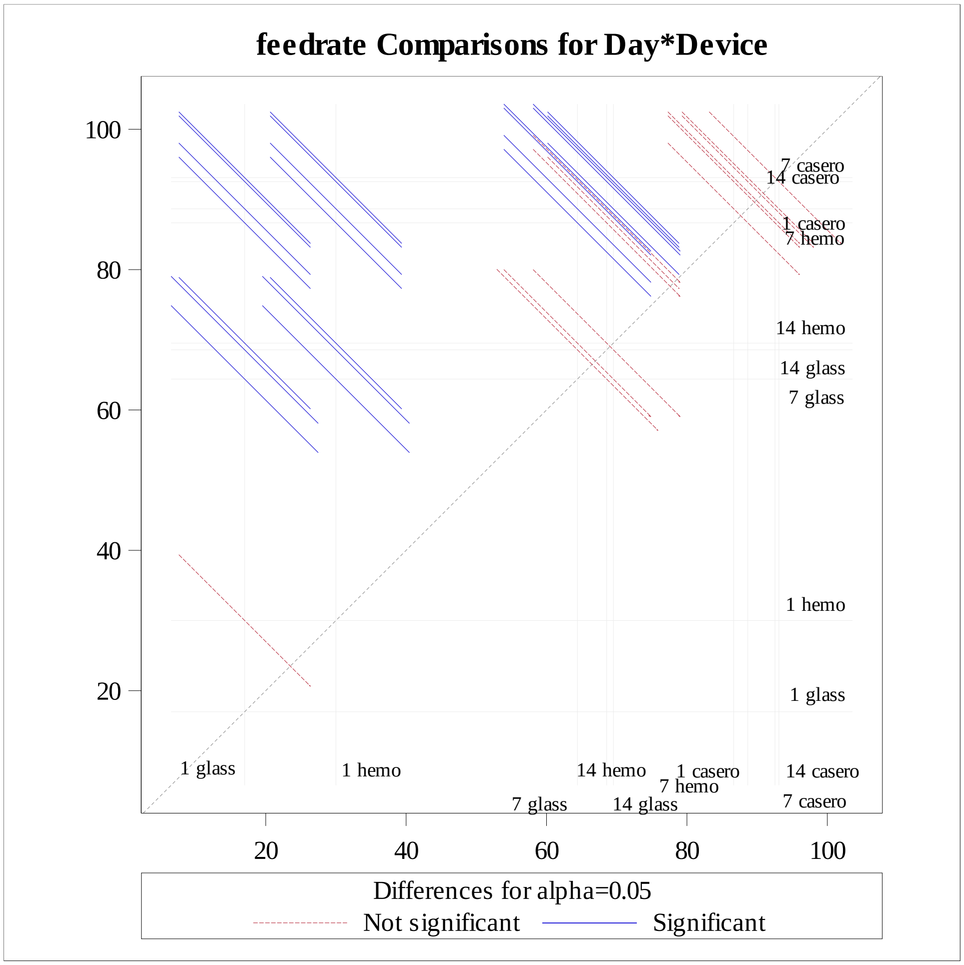

Note: To ensure overall protection level, only probabilities associated with pre-planned comparisons should be used.

The SAS System

The UNIVARIATE Procedure
Variable: RES

Moments			
**N**	25	**Sum Weights**	25
**Mean**	0	**Sum Observations**	0
**Std Deviation**	8.84552696	**Variance**	78.2433471
**Skewness**	-0.6708299	**Kurtosis**	0.38276006
**Uncorrected SS**	1877.84033	**Corrected SS**	1877.84033
**Coeff Variation**	.	**Std Error Mean**	1.76910539

Basic Statistical Measures			
**Location**		**Variability**	
**Mean**	0.00000	**Std Deviation**	8.84553
**Median**	0.11521	**Variance**	78.24335
**Mode**	-0.95238	**Range**	36.66667
		**Interquartile Range**	6.95238

Note: The mode displayed is the smallest of 2 modes with a count of 2.

Tests for Location: Mu0=0				
**Test**	**Statistic**		**p Value**	
**Student's t**	**t**	0	**Pr > |t|**	1.0000
**Sign**	**M**	0.5	**Pr >= |M|**	1.0000
**Signed Rank**	**S**	10.5	**Pr >= |S|**	0.7839

Tests for Normality				
**Test**	**Statistic**		**p Value**	
**Shapiro-Wilk**	**W**	0.929366	**Pr < W**	0.0840
**Kolmogorov-Smirnov**	**D**	0.226682	**Pr > D**	<0.0100
**Cramer-von Mises**	**W-Sq**	0.168498	**Pr > W-Sq**	0.0131
**Anderson-Darling**	**A-Sq**	0.841954	**Pr > A-Sq**	0.0256

The SAS System

The UNIVARIATE Procedure
Variable: RES

Quantiles (Definition 5)	
**Level**	**Quantile**
**100% Max**	15.000000
**99%**	15.000000
**95%**	11.428571
**90%**	11.333333
**75% Q3**	6.000000
**50% Median**	0.115207
**25% Q1**	-0.952381
**10%**	-12.380952
**5%**	-15.000000
**1%**	-21.666667
**0% Min**	-21.666667

Extreme Observations			
**Lowest**		**Highest**	
**Value**	**Obs**	**Value**	**Obs**
-21.6667	4	7.61905	22
-15.0000	2	10.33333	1
-12.3810	19	11.33333	7
-12.0000	3	11.42857	24
-11.4286	21	15.00000	8

The SAS System

The UNIVARIATE Procedure

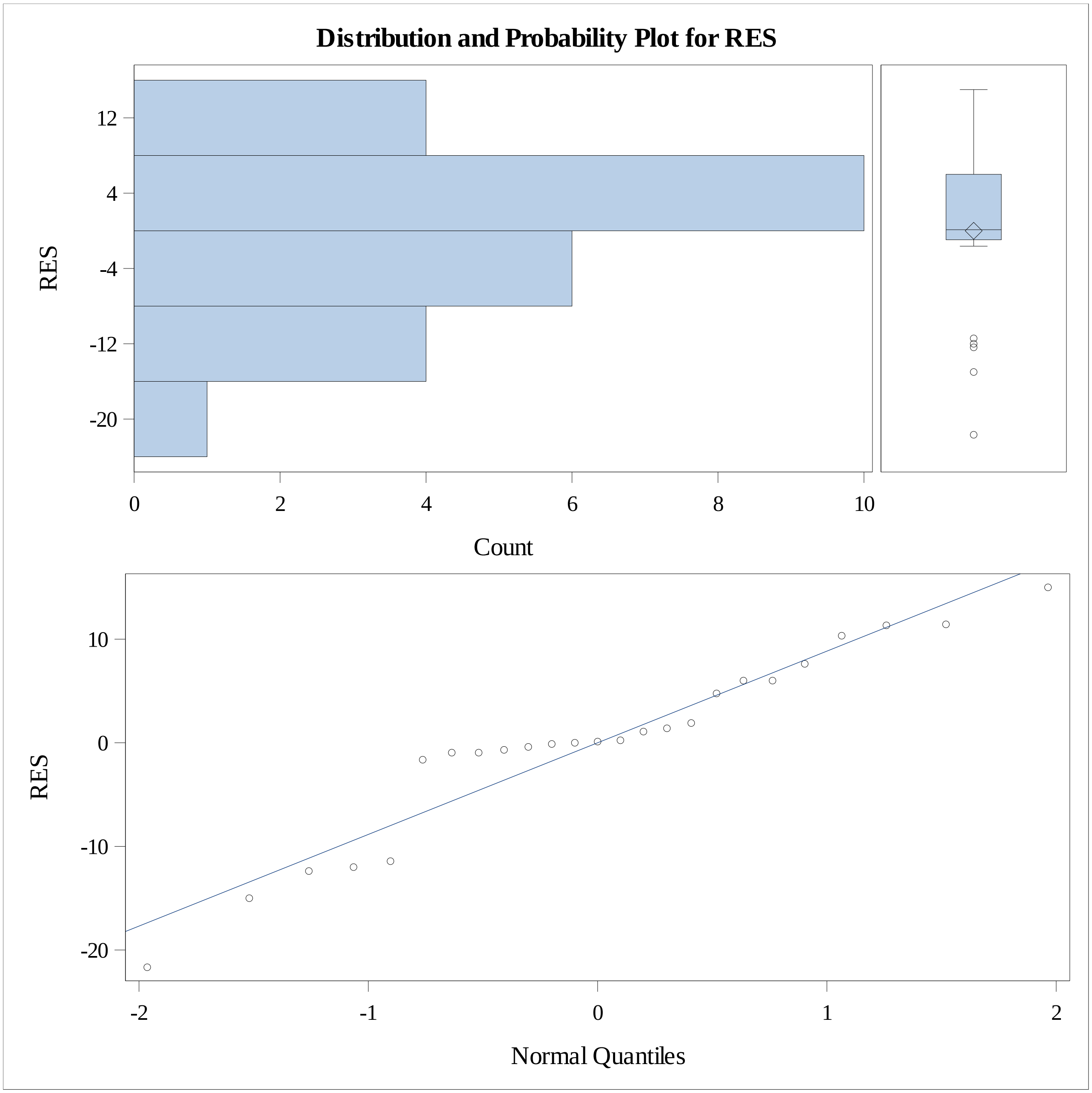

The SAS System

The MEANS Procedure

Analysis Variable : feedrate feedrate					
**Day**	**Device**	**N Obs**	**Mean**	**Std Dev**	**Std Error**
1	casero	15	80.9546004	13.5284342	3.4930267
	glass	15	61.7055777	25.2799490	6.5272548
	hemo	15	56.0296397	25.7948681	6.6602063
7	casero	6	94.5781551	3.1992682	1.3060958
	glass	6	86.7446009	17.4075570	7.1066054
	hemo	6	91.6151203	5.6093694	2.2900155
14	casero	6	95.7079172	3.0717545	1.2540385
	glass	6	78.5824662	20.3720383	8.3168498
	hemo	6	80.9348113	14.2813882	5.8303523
21	casero	3	98.6394558	2.3565317	1.3605442
	glass	3	98.2673931	0.5889738	0.3400442
	hemo	3	97.9238259	0.0124001	0.0071592

The SAS System

The GLM Procedure

Class Level Information		
**Class**	**Levels**	**Values**
**Day**	3	1 7 14
**Repeat**	3	A B C
**Device**	3	casero glass hemo
**feedrate**	23	2 17 18 32 36 50 57.142857143 64.285714286 64.516129032 67 74.285714286 77.142857143 80 85.714285714 88.571428571 90.909090909 92.424242424 92.708333333 92.783505155 93.939393939 94.186046512 99 100

**Number of Observations Read**	27
**Number of Observations Used**	27

The SAS System

The GLM Procedure

Dependent Variable: feedrate feedrate

Source	DF	Sum of Squares	Mean Square	F Value	Pr > F
**Model**	8	18460.82954	2307.60369	14.07	<.0001
**Error**	18	2952.63522	164.03529		
**Corrected Total**	26	21413.46476			

R-Square	Coeff Var	Root MSE	feedrate Mean
0.862113	18.70772	12.80763	68.46173

Source	DF	Type III SS	Mean Square	F Value	Pr > F
**Day**	2	7755.127757	3877.563879	23.64	<.0001
**Device**	2	7593.778493	3796.889247	23.15	<.0001
**Day*Device**	4	3111.923289	777.980822	4.74	0.0086

The SAS System

The GLM Procedure

Dependent Variable: feedrate feedrate

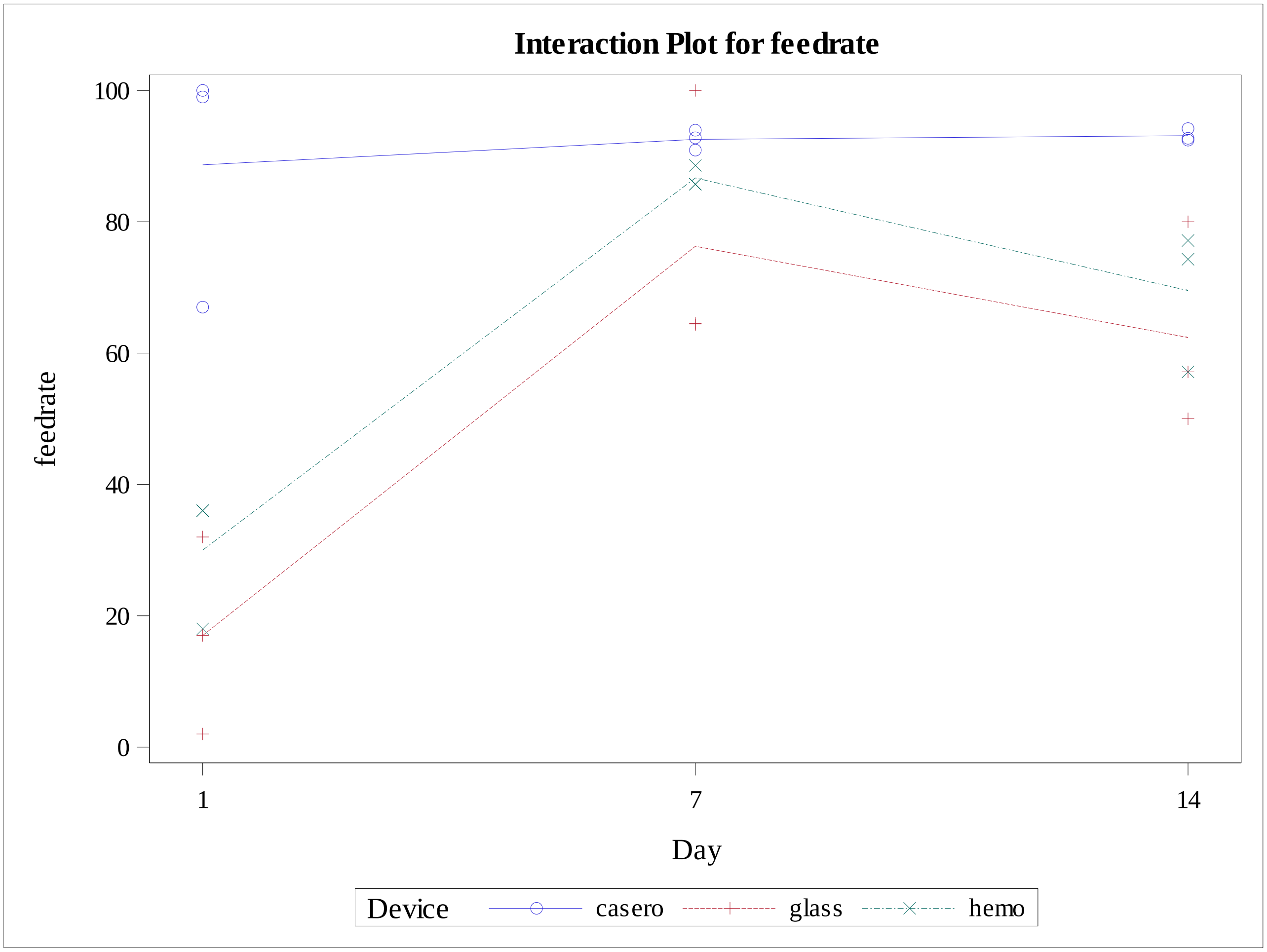

The SAS System

The GLM Procedure
Least Squares Means

Day	feedrate LSMEAN	Standard Error	Pr > |t|	LSMEAN Number
**1**	45.2222222	4.2692088	<.0001	1
**7**	85.1593148	4.2692088	<.0001	2
**14**	75.0036564	4.2692088	<.0001	3

Least Squares Means for Effect Day
t for H0: LSMean(i)=LSMean(j) / Pr > |t|

Dependent Variable: feedrate

i/j	1	2	3
**1**		-6.61476<br><.0001	-4.93268<br>0.0001
**2**	6.614759<br><.0001		1.682076<br>0.1098
**3**	4.932683<br>0.0001	-1.68208<br>0.1098	

The SAS System

The GLM Procedure
Least Squares Means

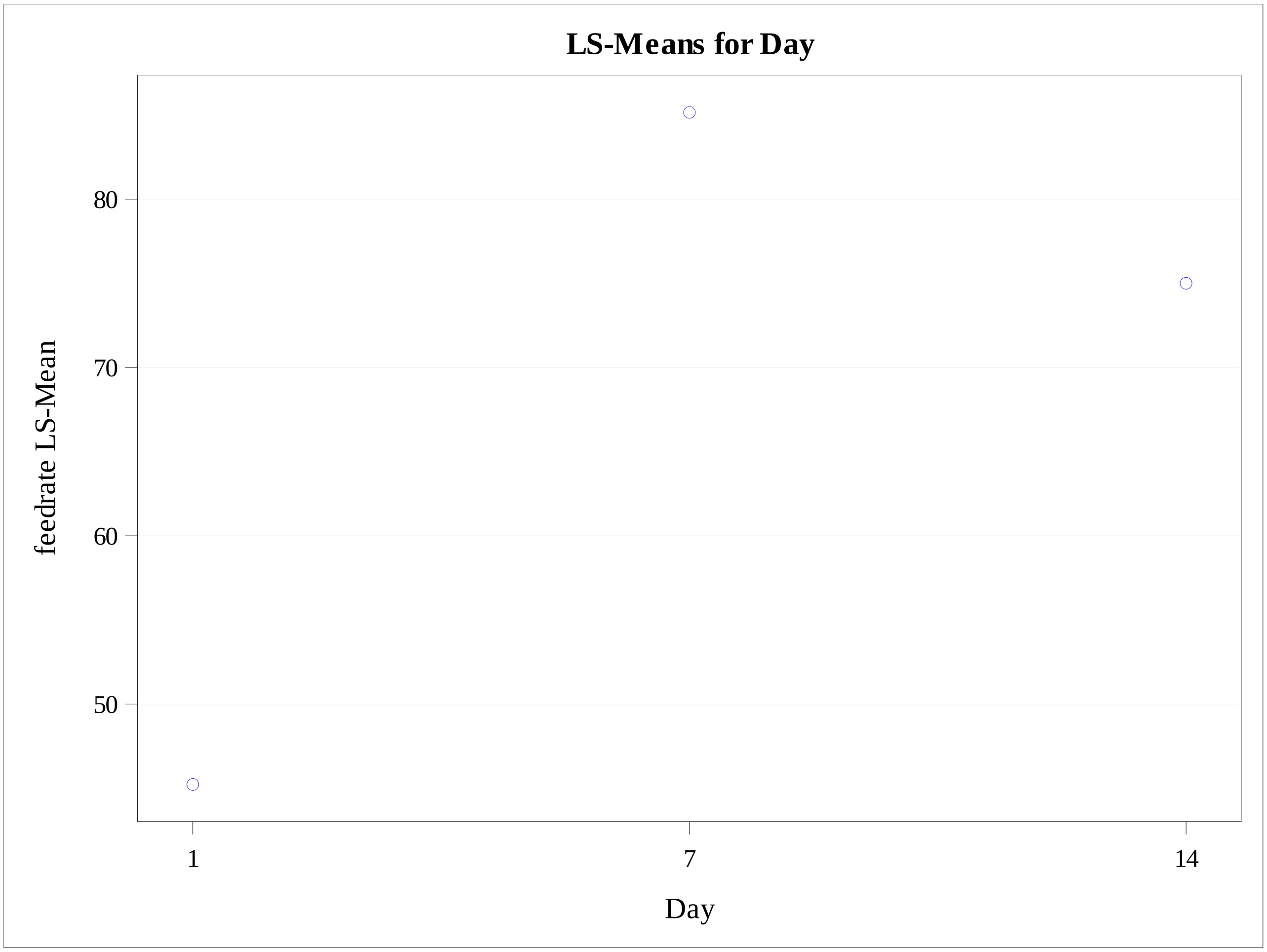

The SAS System

The GLM Procedure
Least Squares Means

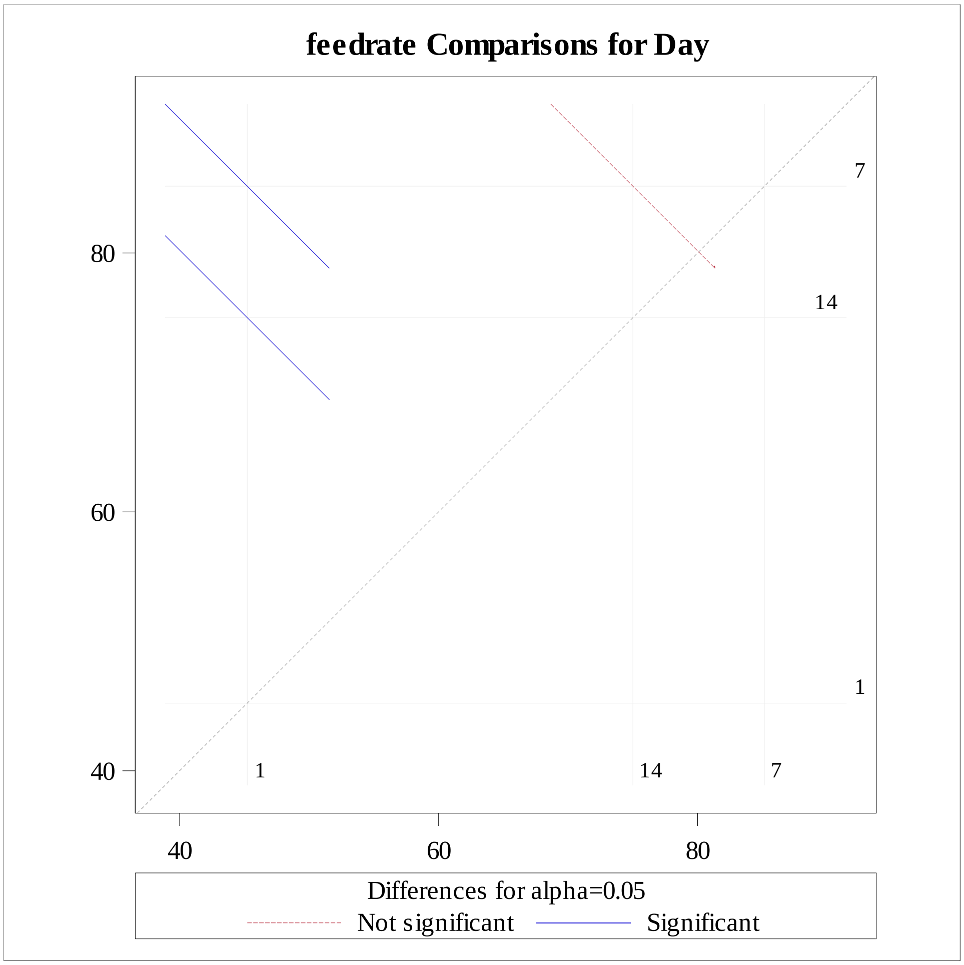

Note: To ensure overall protection level, only probabilities associated with pre-planned comparisons should be used.

The SAS System

The GLM Procedure
Least Squares Means

Device	feedrate LSMEAN	Standard Error	Pr > |t|	LSMEAN Number
casero	91.4389569	4.2692088	<.0001	1
glass	51.8827445	4.2692088	<.0001	2
hemo	62.0634921	4.2692088	<.0001	3

Least Squares Means for Effect Device
t for H0: LSMean(i)=LSMean(j) / Pr > |t|

Dependent Variable: feedrate

i/j	1	2	3
1		6.551674<br><.0001	4.865443<br>0.0001
2	-6.55167<br><.0001		-1.68623<br>0.1090
3	-4.86544<br>0.0001	1.686232<br>0.1090	

The SAS System

The GLM Procedure
Least Squares Means

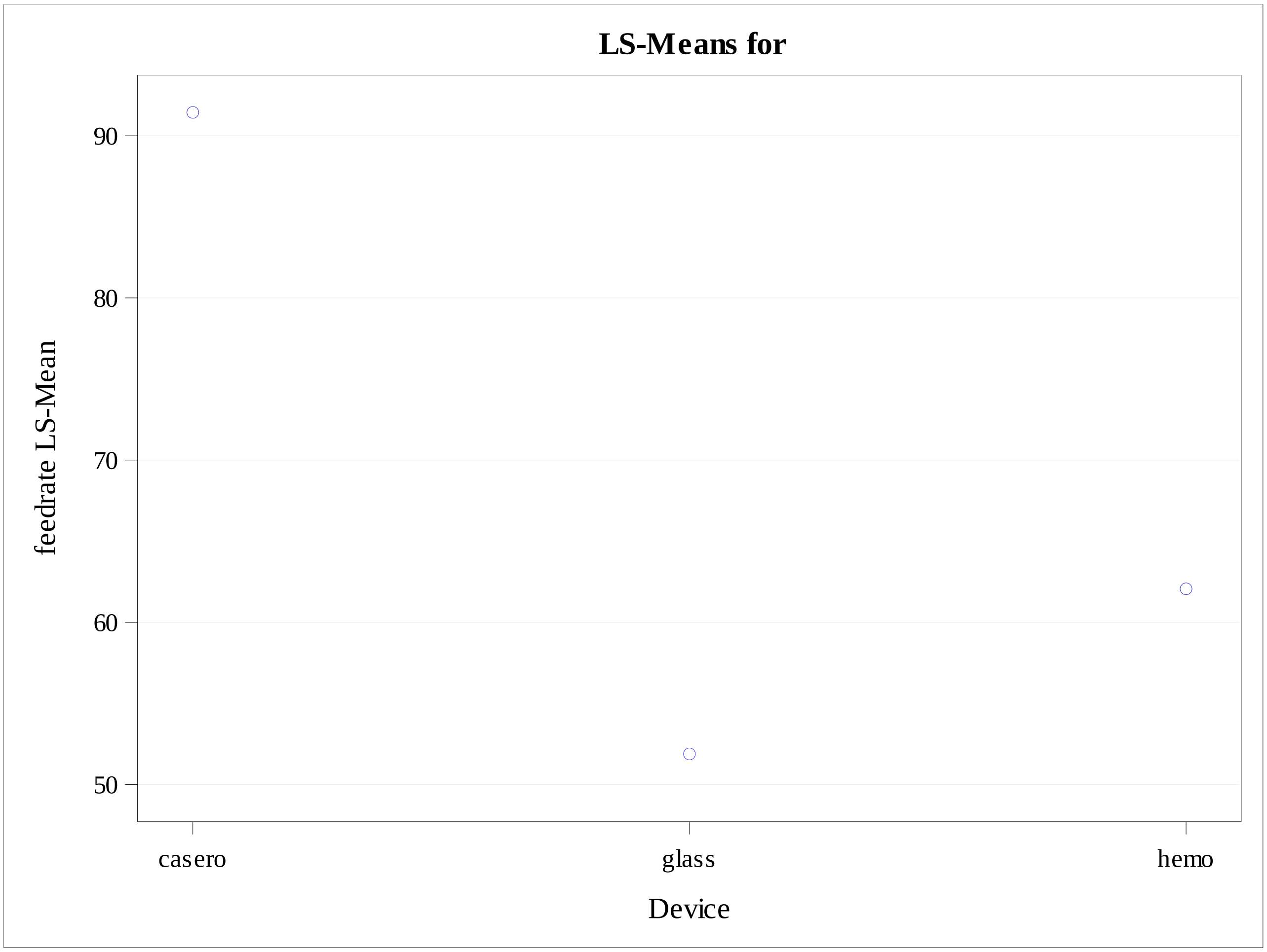

The SAS System

The GLM Procedure
Least Squares Means

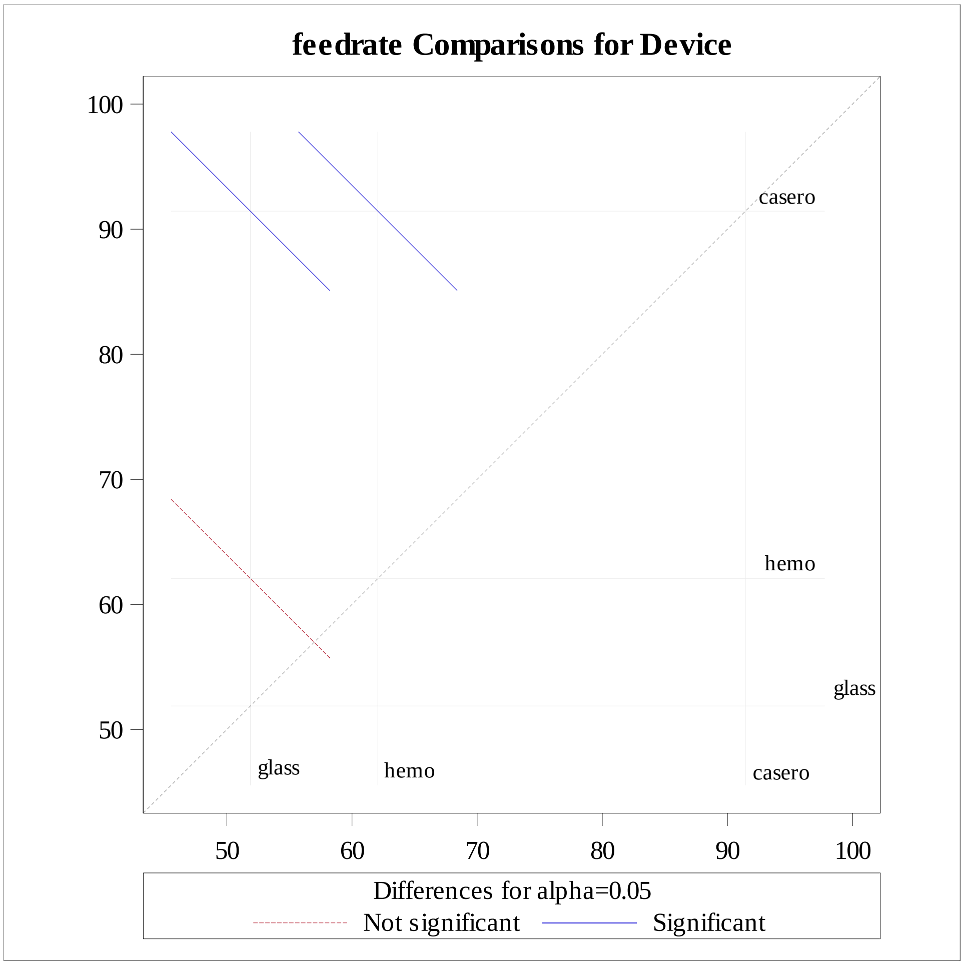

Note: To ensure overall protection level, only probabilities associated with pre-planned comparisons should be used.

The SAS System

The GLM Procedure
Least Squares Means

Day	Device	feedrate LSMEAN	Standard Error	Pr > |t|	LSMEAN Number
**1**	**casero**	88.6666667	7.3944865	<.0001	1
**1**	**glass**	17.0000000	7.3944865	0.0337	2
**1**	**hemo**	30.0000000	7.3944865	0.0007	3
**7**	**casero**	92.5439967	7.3944865	<.0001	4
**7**	**glass**	76.2672811	7.3944865	<.0001	5
**7**	**hemo**	86.6666667	7.3944865	<.0001	6
**14**	**casero**	93.1062074	7.3944865	<.0001	7
**14**	**glass**	62.3809524	7.3944865	<.0001	8
**14**	**hemo**	69.5238095	7.3944865	<.0001	9

Least Squares Means for Effect Day*Device
t for H0: LSMean(i)=LSMean(j) / Pr > |t|

Dependent Variable: feedrate

i/j	1	2	3	4	5	6	7	8	9
**1**		6.853212<br><.0001	5.610072<br><.0001	-0.37077<br>0.7151	1.185706<br>0.2512	0.191252<br>0.8505	-0.42454<br>0.6762	2.513603<br>0.0217	1.830559<br>0.0838
**2**	-6.85321<br><.0001		-1.24314<br>0.2298	-7.22399<br><.0001	-5.66751<br><.0001	-6.66196<br><.0001	-7.27775<br><.0001	-4.33961<br>0.0004	-5.02265<br><.0001
**3**	-5.61007<br><.0001	1.243141<br>0.2298		-5.98085<br><.0001	-4.42437<br>0.0003	-5.41882<br><.0001	-6.03461<br><.0001	-3.09647<br>0.0062	-3.77951<br>0.0014
**4**	0.370774<br>0.7151	7.223987<br><.0001	5.980846<br><.0001		1.556481<br>0.1370	0.562027<br>0.5810	-0.05376<br>0.9577	2.884378<br>0.0099	2.201333<br>0.0410
**5**	-1.18571<br>0.2512	5.667506<br><.0001	4.424365<br>0.0003	-1.55648<br>0.1370		-0.99445<br>0.3332	-1.61024<br>0.1247	1.327897<br>0.2008	0.644853<br>0.5272
**6**	-0.19125<br>0.8505	6.66196<br><.0001	5.418819<br><.0001	-0.56203<br>0.5810	0.994454<br>0.3332		-0.61579<br>0.5457	2.322351<br>0.0321	1.639307<br>0.1185
**7**	0.424536<br>0.6762	7.277749<br><.0001	6.034608<br><.0001	0.053762<br>0.9577	1.610243<br>0.1247	0.615789<br>0.5457		2.93814<br>0.0088	2.255096<br>0.0368
**8**	-2.5136<br>0.0217	4.339609<br>0.0004	3.096468<br>0.0062	-2.88438<br>0.0099	-1.3279<br>0.2008	-2.32235<br>0.0321	-2.93814<br>0.0088		-0.68304<br>0.5033
**9**	-1.83056<br>0.0838	5.022653<br><.0001	3.779512<br>0.0014	-2.20133<br>0.0410	-0.64485<br>0.5272	-1.63931<br>0.1185	-2.2551<br>0.0368	0.683044<br>0.5033	

The SAS System

The GLM Procedure
Least Squares Means

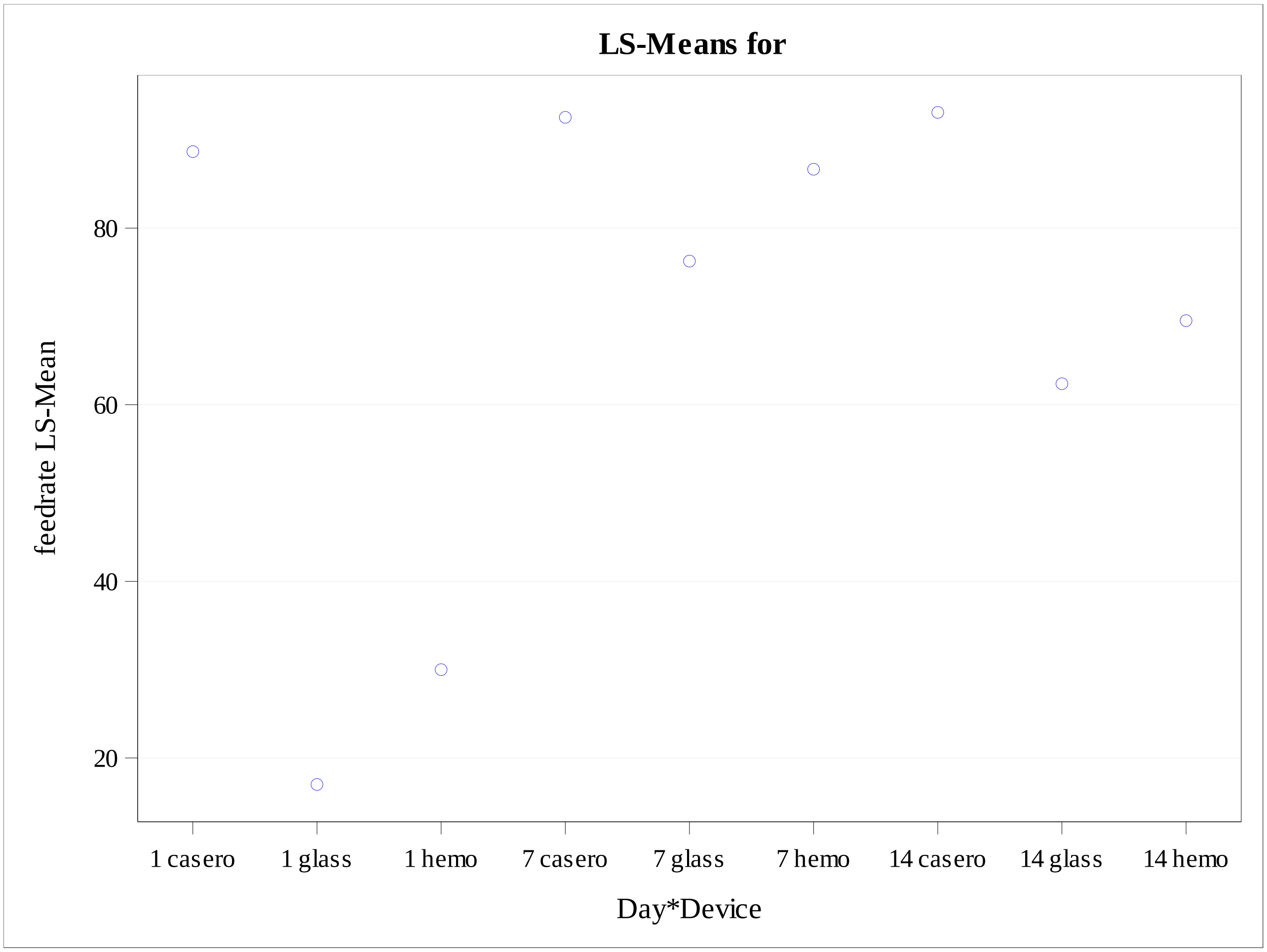

The SAS System

The GLM Procedure
Least Squares Means

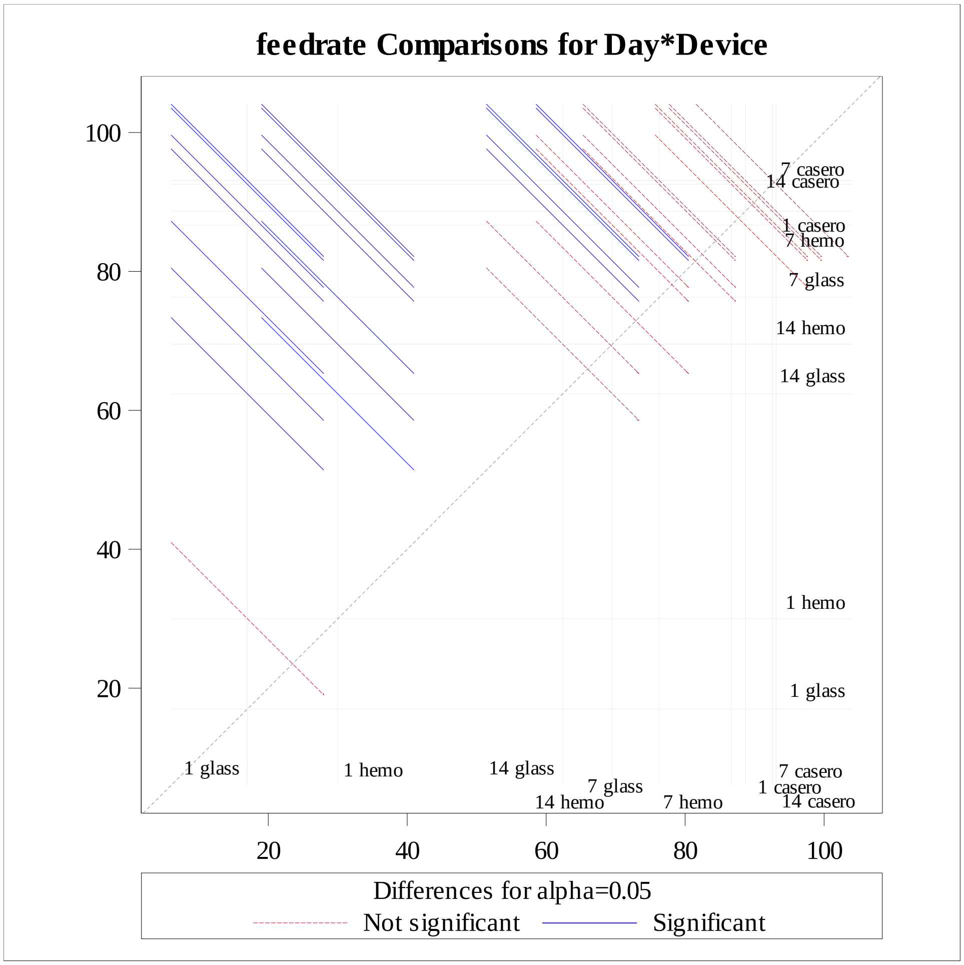

Note: To ensure overall protection level, only probabilities associated with pre-planned comparisons should be used.

The SAS System

The UNIVARIATE Procedure
Variable:
RES

Moments			
**N**	27	**Sum Weights**	27
**Mean**	0	**Sum Observations**	0
**Std Deviation**	10.6565892	**Variance**	113.562893
**Skewness**	0.10987194	**Kurtosis**	-0.0814723
**Uncorrected SS**	2952.63522	**Corrected SS**	2952.63522
**Coeff Variation**	.	**Std Error Mean**	2.05086155

Basic Statistical Measures			
**Location**		**Variability**	
**Mean**	0.0000	**Std Deviation**	10.65659
**Median**	0.0000	**Variance**	113.56289
**Mode**	-12.3810	**Range**	45.39939
		**Interquartile Range**	17.75115

Note: The mode displayed is the smallest of 3 modes with a count of 2.

Tests for Location: Mu0=0				
**Test**	**Statistic**		**p Value**	
**Student's t**	**t**	0	**Pr > |t|**	1.0000
**Sign**	**M**	0	**Pr >= |M|**	1.0000
**Signed Rank**	**S**	0	**Pr >= |S|**	1.0000

Tests for Normality				
**Test**	**Statistic**		**p Value**	
**Shapiro-Wilk**	**W**	0.971878	**Pr < W**	0.6520
**Kolmogorov-Smirnov**	**D**	0.142738	**Pr > D**	>0.1500
**Cramer-von Mises**	**W-Sq**	0.075577	**Pr > W-Sq**	0.2325
**Anderson-Darling**	**A-Sq**	0.414571	**Pr > A-Sq**	>0.2500

The SAS System

The UNIVARIATE Procedure
Variable: RES

Quantiles (Definition 5)	
**Level**	**Quantile**
**100% Max**	23.7327
**99%**	23.7327
**95%**	17.6190
**90%**	15.0000
**75% Q3**	6.0000
**50% Median**	0.0000
**25% Q1**	-11.7512
**10%**	-12.3810
**5%**	-15.0000
**1%**	-21.6667
**0% Min**	-21.6667

Extreme Observations			
**Lowest**		**Highest**	
**Value**	**Obs**	**Value**	**Obs**
-21.6667	4	10.3333	1
-15.0000	2	11.3333	7
-12.3810	21	15.0000	8
-12.3810	20	17.6190	26
-12.0000	3	23.7327	11

The SAS System

The UNIVARIATE Procedure

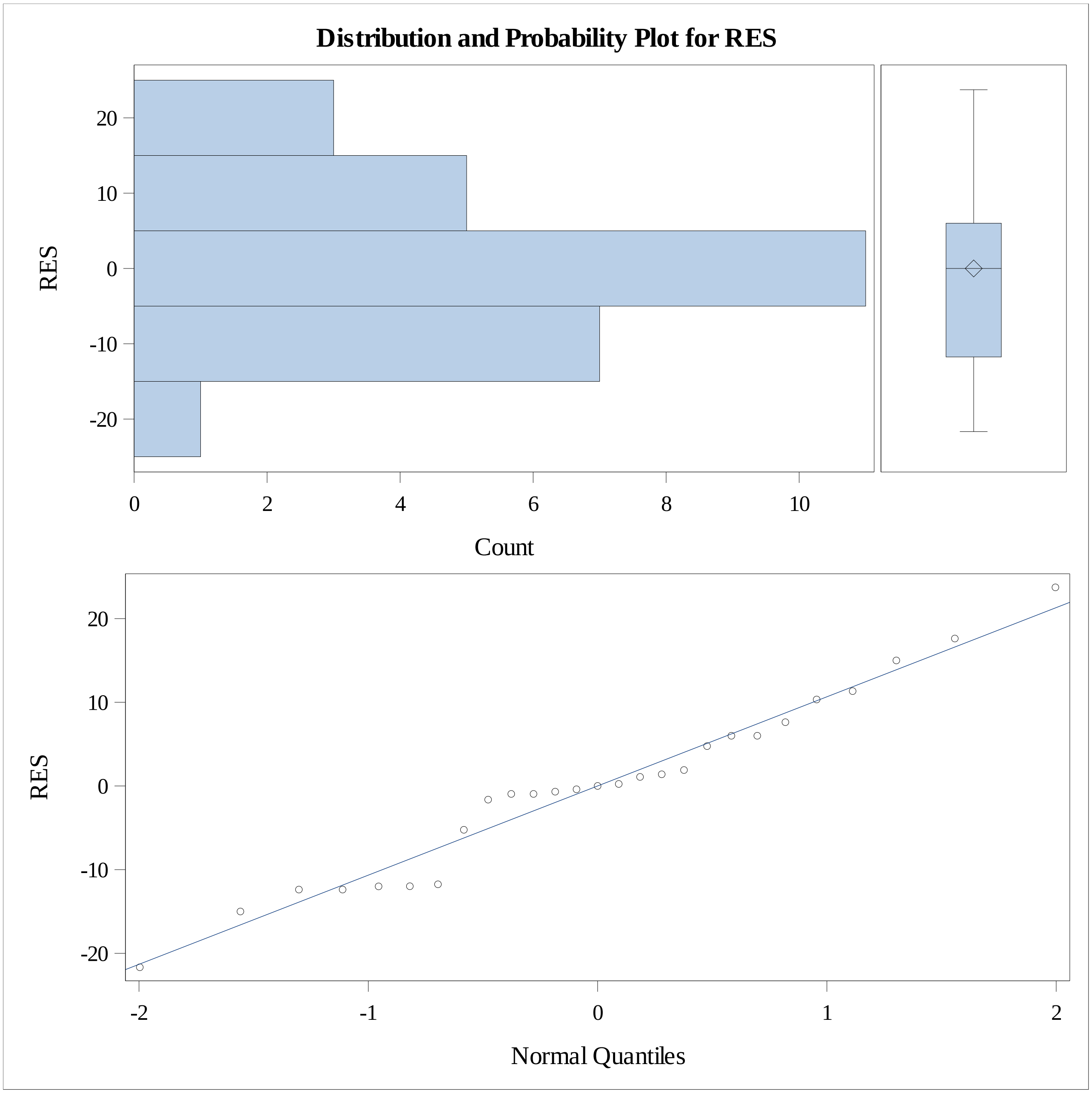

The SAS System

The MEANS Procedure

Analysis Variable : feedrate feedrate					
**Day**	**Device**	**N Obs**	**Mean**	**Std Dev**	**Std Error**
1	casero	15	80.9546004	13.5284342	3.4930267
	glass	15	61.7055777	25.2799490	6.5272548
	hemo	15	56.0296397	25.7948681	6.6602063
7	casero	6	94.5781551	3.1992682	1.3060958
	glass	6	86.7446009	17.4075570	7.1066054
	hemo	6	91.6151203	5.6093694	2.2900155
14	casero	6	95.7079172	3.0717545	1.2540385
	glass	6	78.5824662	20.3720383	8.3168498
	hemo	6	80.9348113	14.2813882	5.8303523
21	casero	3	98.6394558	2.3565317	1.3605442
	glass	3	98.2673931	0.5889738	0.3400442
	hemo	3	97.9238259	0.0124001	0.0071592

Egg production

```
 /*Eggs/female Experiment #1*/
/*Import S1_File_July2023 Sheet Eggs$*/

DATA EXP1_EGGS;
SET EGGS;
IF EXP = 1;
RUN;
```

Replicates with <10 mosquitoes removed.

```
ODS RTF FILE='Exp1_Eggs.RTF';
PROC GLM DATA=EXP1_EGGS;
where include = 1;
CLASS  Week repeat Device;
MODEL egg_mosq =  Week repeat Device week*Device/SS3;
OUTPUT OUT=R RESIDUAL = RES;
LSMEANS Week / STDERR PDIFF TDIFF;
LSMEANS device / STDERR PDIFF TDIFF;
LSMEANS repeat / STDERR PDIFF TDIFF;
LSMEANS week*DEVICE / STDERR PDIFF TDIFF;
*LSMEANS Day*Device / STDERR PDIFF TDIFF;
PROC UNIVARIATE NORMAL PLOT DATA=R; VAR RES; RUN;

proc means data=EXP1_EGGS;
var egg_mosq;
class device;
run;

proc means data=EXP1_EGGS;
var mosq;
class device;
run;
ODS RTF CLOSE;
```

All data points included.

```
ODS RTF FILE='Exp1_Eggs_alldata.RTF';
PROC GLM DATA=EXP1_EGGS;
CLASS  Week repeat Device;
MODEL egg_mosq =  Week repeat Device week*Device/SS3;
OUTPUT OUT=R RESIDUAL = RES;
LSMEANS Week / STDERR PDIFF TDIFF;
LSMEANS device / STDERR PDIFF TDIFF;
LSMEANS repeat / STDERR PDIFF TDIFF;
LSMEANS week*DEVICE / STDERR PDIFF TDIFF;
*LSMEANS Day*Device / STDERR PDIFF TDIFF;
PROC UNIVARIATE NORMAL PLOT DATA=R; VAR RES; RUN;
proc means data=EXP1_EGGS;
var egg_mosq;
class device;
run;
PROC MEANS DATA=EXP1_EGGS MEAN STD MIN MAX;
VAR MOSQ;
CLASS DEVICE;
RUN;
ODS RTF CLOSE;
```

The SAS System

The GLM Procedure

Class Level Information		
**Class**	**Levels**	**Values**
**Week**	3	1 2 3
**Repeat**	3	A B C
**Device**	3	CASERO GLASS HEMO

**Number of Observations Read**	24
**Number of Observations Used**	24

The SAS System

The GLM Procedure

Dependent Variable: egg_mosq
egg_mosq

Source	DF	Sum of Squares	Mean Square	F Value	Pr > F
Model	10	7640.75915	764.07591	0.99	0.4988
Error	13	10074.51847	774.96296		
Corrected Total	23	17715.27762			

R-Square	Coeff Var	Root MSE	egg_mosq Mean
0.431309	72.57333	27.83816	38.35866

Source	DF	Type III SS	Mean Square	F Value	Pr > F
Week	2	766.193478	383.096739	0.49	0.6210
Repeat	2	3014.694705	1507.347352	1.95	0.1824
Device	2	4072.782271	2036.391135	2.63	0.1100
Week*Device	4	896.675241	224.168810	0.29	0.8797

The SAS System

The GLM Procedure
Least Squares Means

Week	egg_mosq LSMEAN	Standard Error	Pr > |t|	LSMEAN Number
1	42.8739564	10.1415000	0.0010	1
2	49.0155638	10.1415000	0.0003	2
3	34.9587436	10.1415000	0.0043	3

Least Squares Means for Effect Week
t for H0: LSMean(i)=LSMean(j) / Pr > |t|

Dependent Variable: egg_mosq

i/j	1	2	3
1		-0.43329<br>0.6719	0.558412<br>0.5861
2	0.433286<br>0.6719		0.991698<br>0.3394
3	-0.55841<br>0.5861	-0.9917<br>0.3394	

The SAS System

The GLM Procedure
Least Squares Means

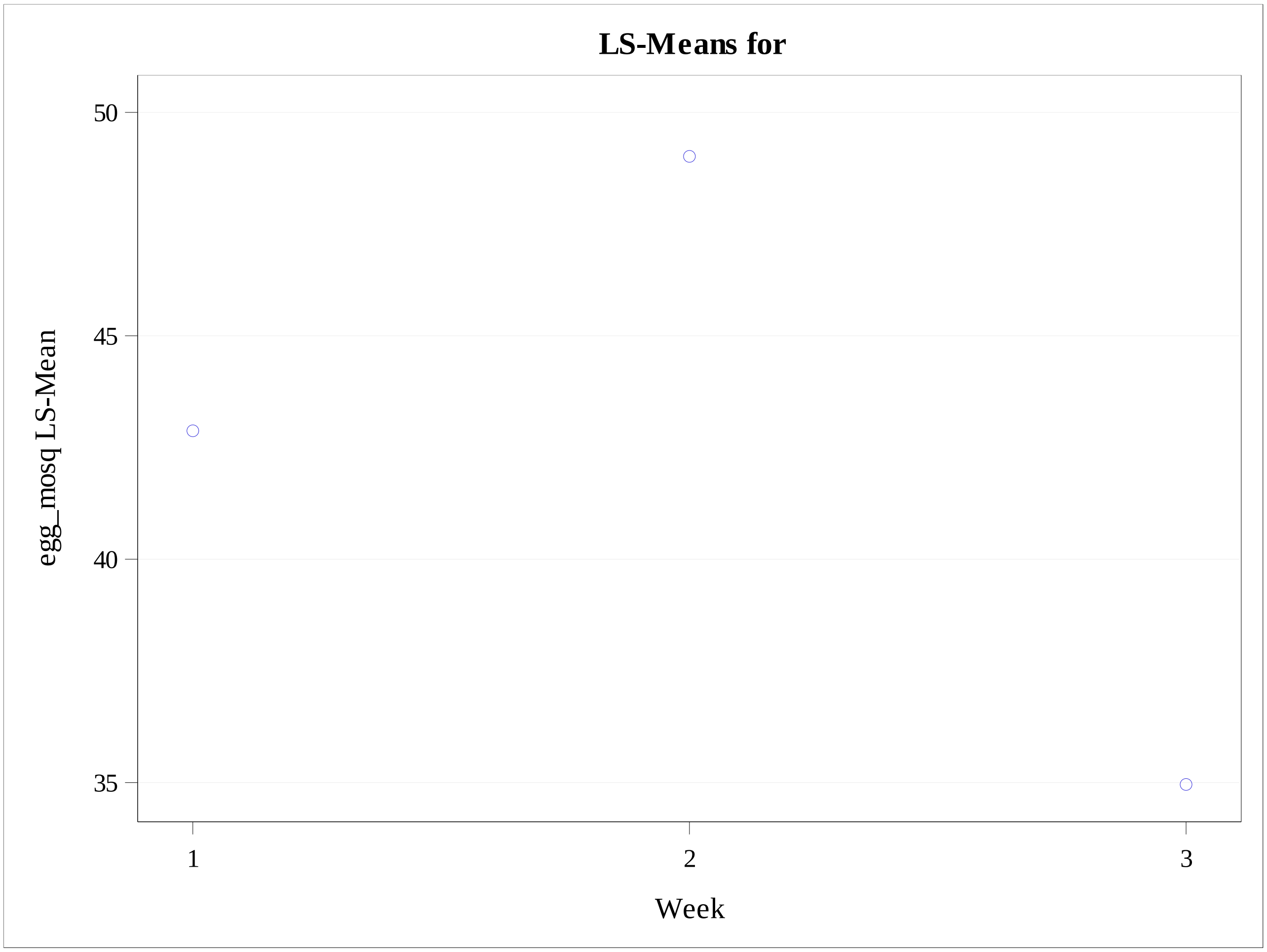

The SAS System

The GLM Procedure
Least Squares Means

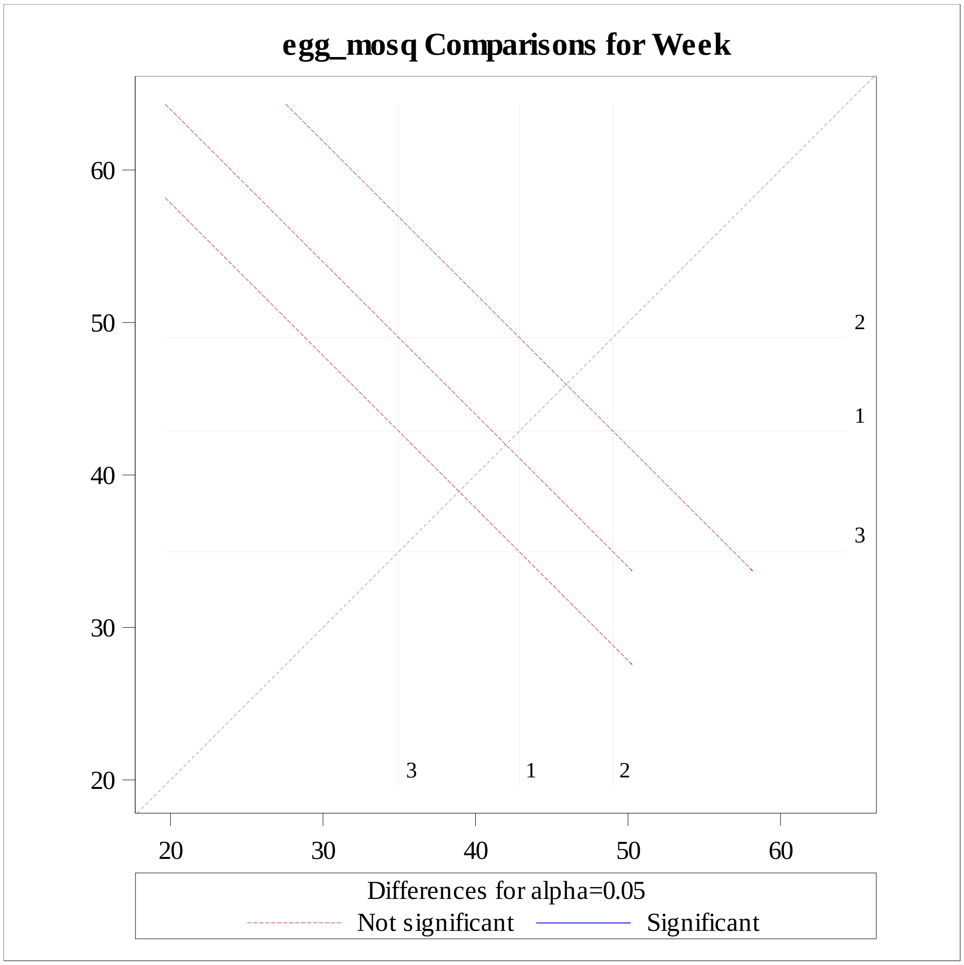

Note: To ensure overall protection level, only probabilities associated with pre-planned comparisons should be used.

The SAS System

The GLM Procedure
Least Squares Means

Device	egg_mosq LSMEAN	Standard Error	Pr > |t|	LSMEAN Number
**CASERO**	27.8694103	9.2793855	0.0102	1
**GLASS**	62.7094973	12.2754732	0.0002	2
**HEMO**	36.2693563	9.2793855	0.0018	3

Least Squares Means for Effect Device<br>t for H0: LSMean(i)=LSMean(j) / Pr > |t| <br>Dependent Variable: egg_mosq			
**i/j**	**1**	**2**	**3**
**1**		-2.26409<br>0.0413	-0.64009<br>0.5332
**2**	2.26409<br>0.0413		1.718218<br>0.1095
**3**	0.640092<br>0.5332	-1.71822<br>0.1095	

The SAS System

The GLM Procedure
Least Squares Means

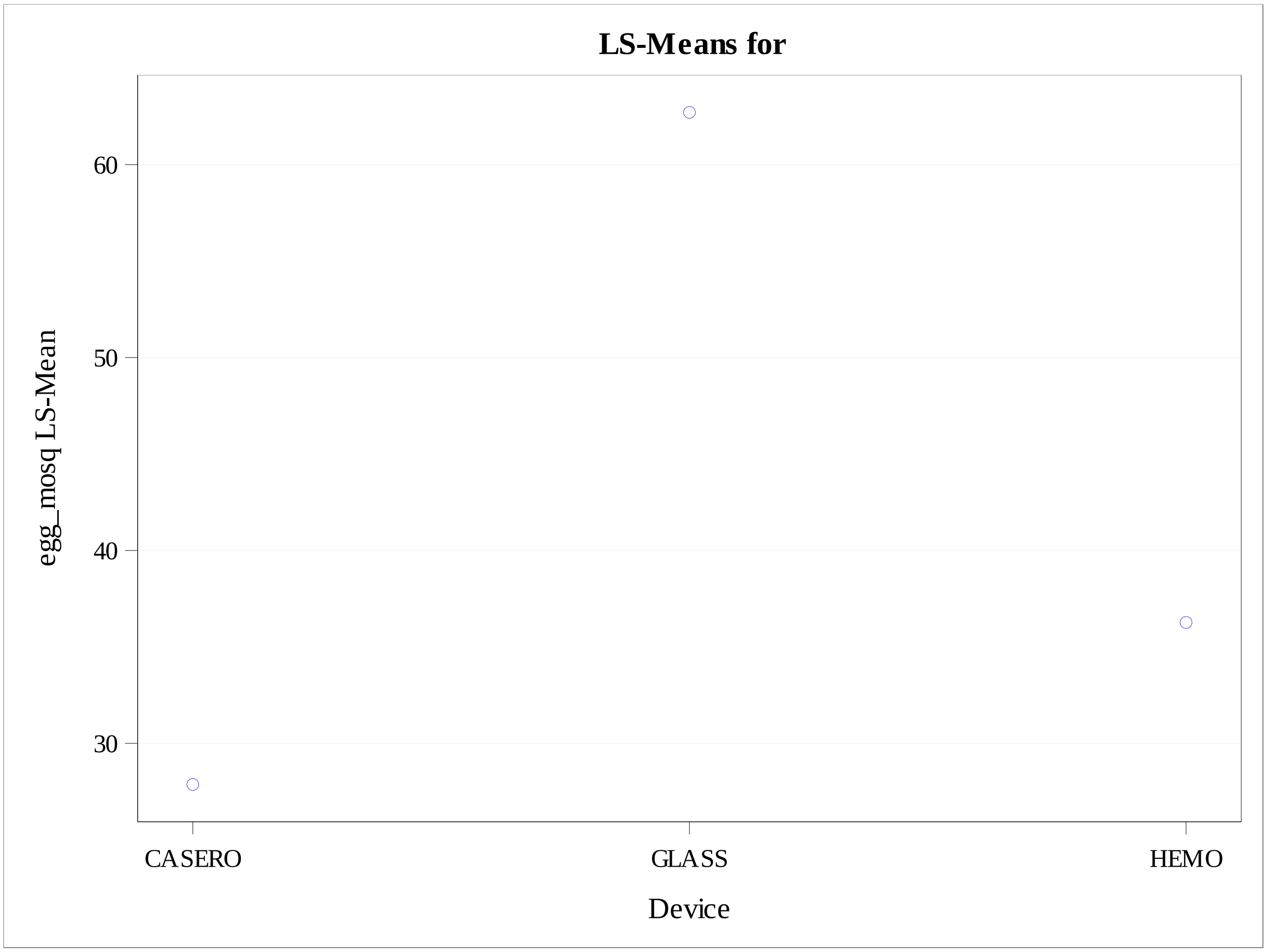

The SAS System

The GLM Procedure
Least Squares Means

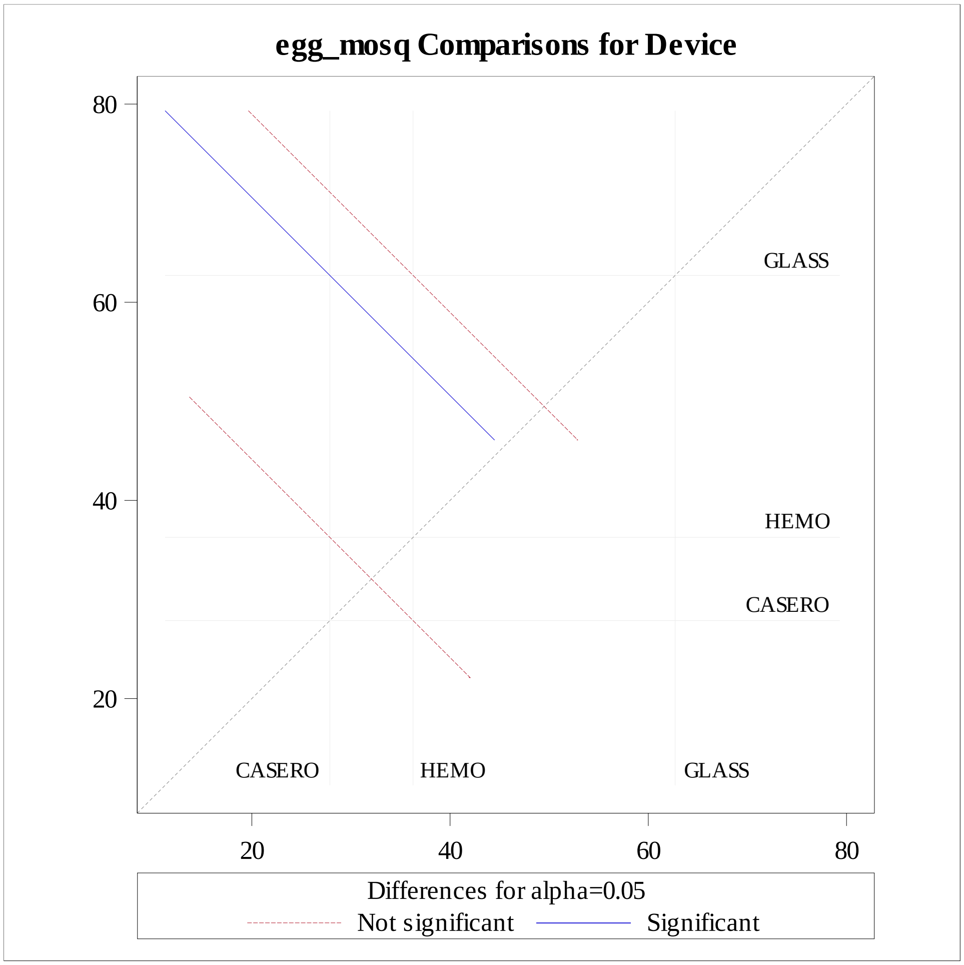

Note: To ensure overall protection level, only probabilities associated with pre-planned comparisons should be used.

The SAS System

The GLM Procedure
Least Squares Means

Repeat	egg_mosq LSMEAN	Standard Error	Pr > |t|	LSMEAN Number
**A**	53.2487775	12.2754732	0.0008	1
**B**	47.1610274	9.2793855	0.0002	2
**C**	26.4384590	9.2793855	0.0137	3

Least Squares Means for Effect Repeat<br>t for H0: LSMean(i)=LSMean(j) / Pr > |t| <br>Dependent Variable: egg_mosq			
**i/j**	**1**	**2**	**3**
**1**		0.395614<br>0.6988	1.742274<br>0.1051
**2**	-0.39561<br>0.6988		1.579099<br>0.1383
**3**	-1.74227<br>0.1051	-1.5791<br>0.1383	

The SAS System

The GLM Procedure
Least Squares Means

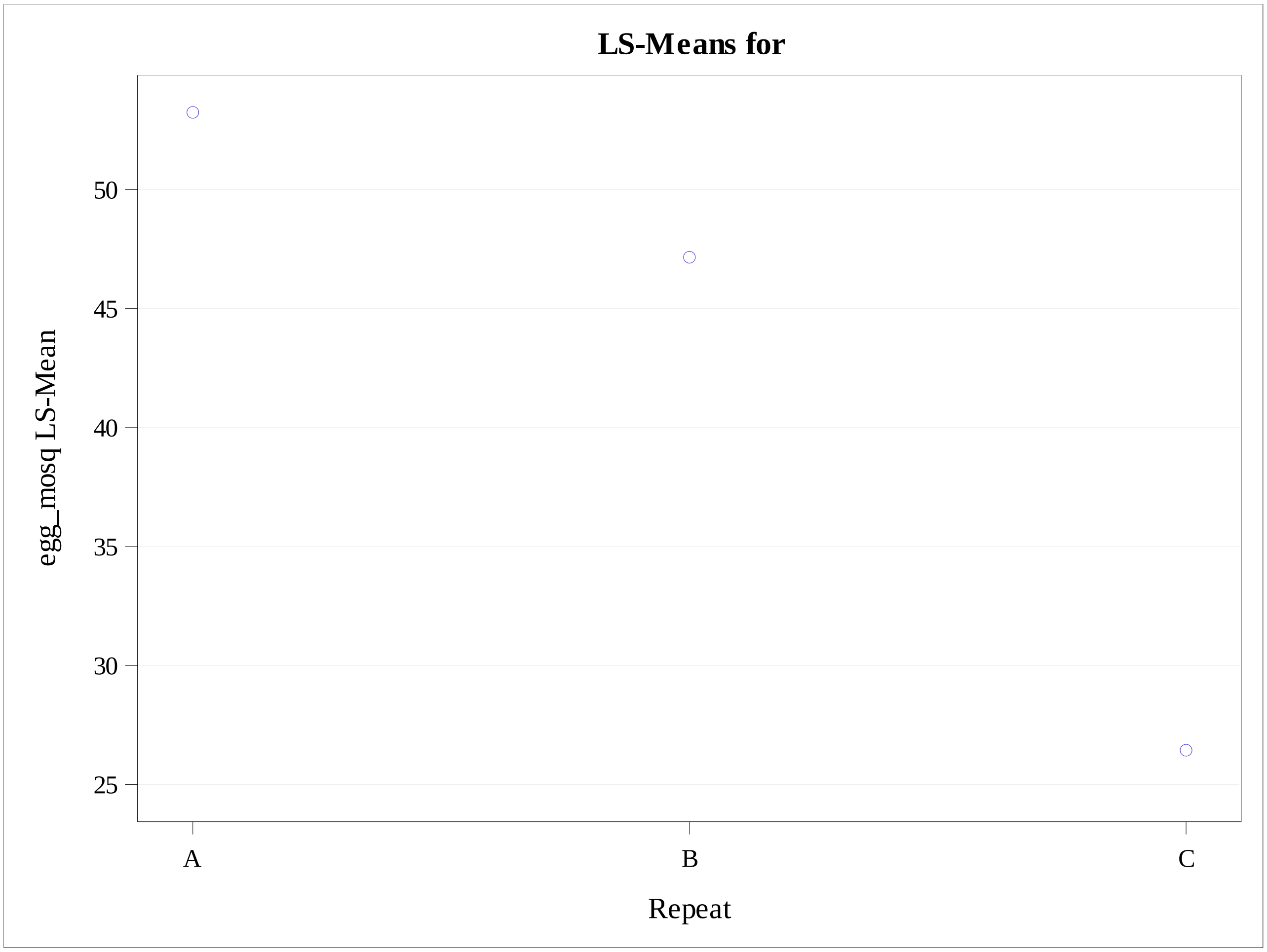

The SAS System

The GLM Procedure
Least Squares Means

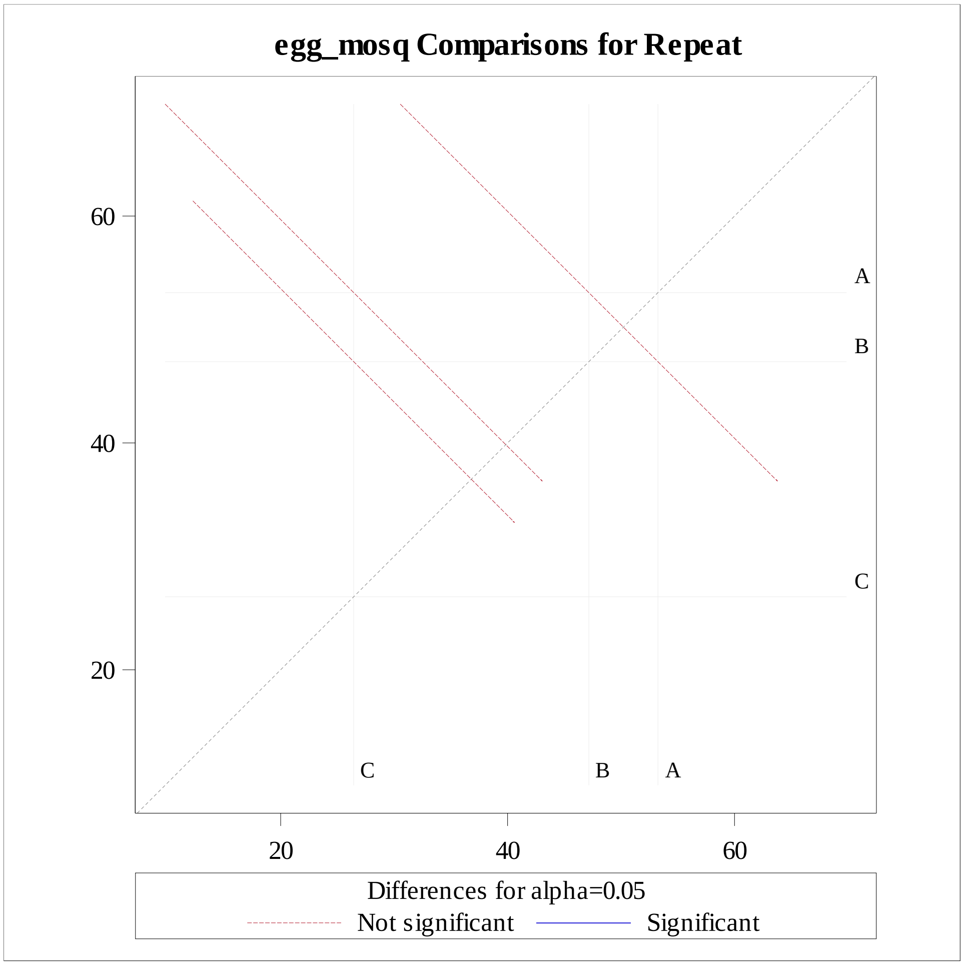

Note: To ensure overall protection level, only probabilities associated with pre-planned comparisons should be used.

The SAS System

The GLM Procedure
Least Squares Means

Week	Device	egg_mosq LSMEAN	Standard Error	Pr > |t|	LSMEAN Number
1	CASERO	30.4484927	16.0723672	0.0806	1
1	GLASS	57.5260222	20.2239519	0.0138	2
1	HEMO	40.6473545	16.0723672	0.0252	3
2	CASERO	25.9612990	16.0723672	0.1302	4
2	GLASS	81.9425352	20.2239519	0.0014	5
2	HEMO	39.1428571	16.0723672	0.0300	6
3	CASERO	27.1984391	16.0723672	0.1144	7
3	GLASS	48.6599345	20.2239519	0.0317	8
3	HEMO	29.0178571	16.0723672	0.0942	9

Least Squares Means for Effect Week*Device
t for H0: LSMean(i)=LSMean(j) / Pr > |t|

Dependent Variable: egg_mosq

i/j	1	2	3	4	5	6	7	8	9
1		-1.04819<br>0.3136	-0.4487<br>0.6610	0.197415<br>0.8466	-1.99337<br>0.0676	-0.38251<br>0.7083	0.142987<br>0.8885	-0.70498<br>0.4933	0.062941<br>0.9508
2	1.048187<br>0.3136		0.653383<br>0.5249	1.221889<br>0.2434	-0.87709<br>0.3964	0.711623<br>0.4893	1.173999<br>0.2615	0.318487<br>0.7552	1.103568<br>0.2898
3	0.448701<br>0.6610	-0.65338<br>0.5249		0.646116<br>0.5294	-1.59856<br>0.1339	0.066191<br>0.9482	0.591688<br>0.5642	-0.31017<br>0.7613	0.511642<br>0.6175
4	-0.19741<br>0.8466	-1.22189<br>0.2434	-0.64612<br>0.5294		-2.16707<br>0.0494	-0.57993<br>0.5719	-0.05443<br>0.9574	-0.87868<br>0.3955	-0.13447<br>0.8951
5	1.993365<br>0.0676	0.877088<br>0.3964	1.598561<br>0.1339	2.167067<br>0.0494		1.656801<br>0.1215	2.119177<br>0.0539	1.195575<br>0.2532	2.048746<br>0.0612
6	0.38251<br>0.7083	-0.71162<br>0.4893	-0.06619<br>0.9482	0.579925<br>0.5719	-1.6568<br>0.1215		0.525497<br>0.6081	-0.36841<br>0.7185	0.445451<br>0.6633
7	-0.14299<br>0.8885	-1.174<br>0.2615	-0.59169<br>0.5642	0.054428<br>0.9574	-2.11918<br>0.0539	-0.5255<br>0.6081		-0.83079<br>0.4211	-0.08005<br>0.9374
8	0.704976<br>0.4933	-0.31849<br>0.7552	0.310172<br>0.7613	0.878678<br>0.3955	-1.19557<br>0.2532	0.368412<br>0.7185	0.830787<br>0.4211		0.760357<br>0.4606
9	-0.06294<br>0.9508	-1.10357<br>0.2898	-0.51164<br>0.6175	0.134474<br>0.8951	-2.04875<br>0.0612	-0.44545<br>0.6633	0.080046<br>0.9374	-0.76036<br>0.4606	

The SAS System

The GLM Procedure
Least Squares Means

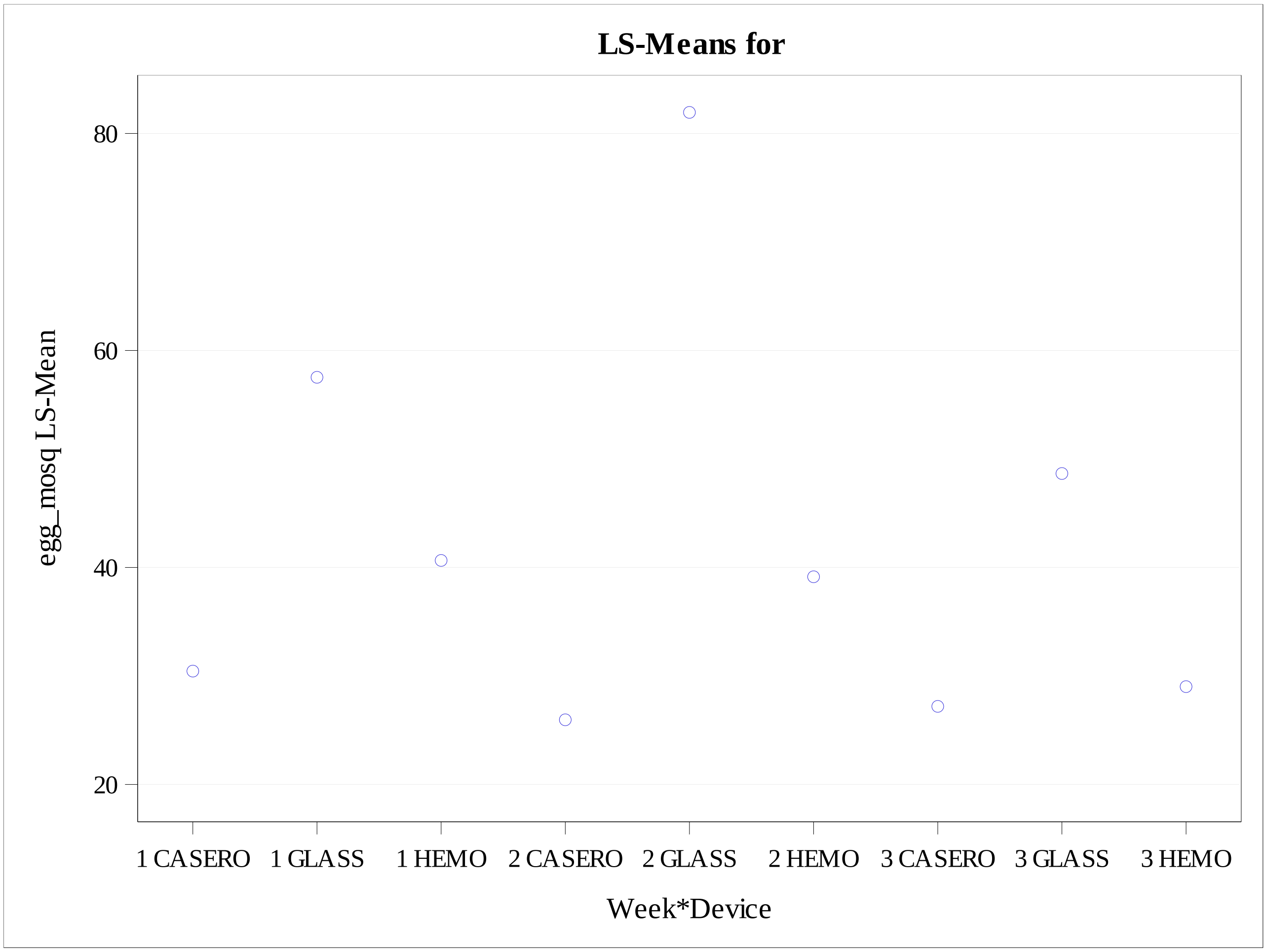

The SAS System

The GLM Procedure
Least Squares Means

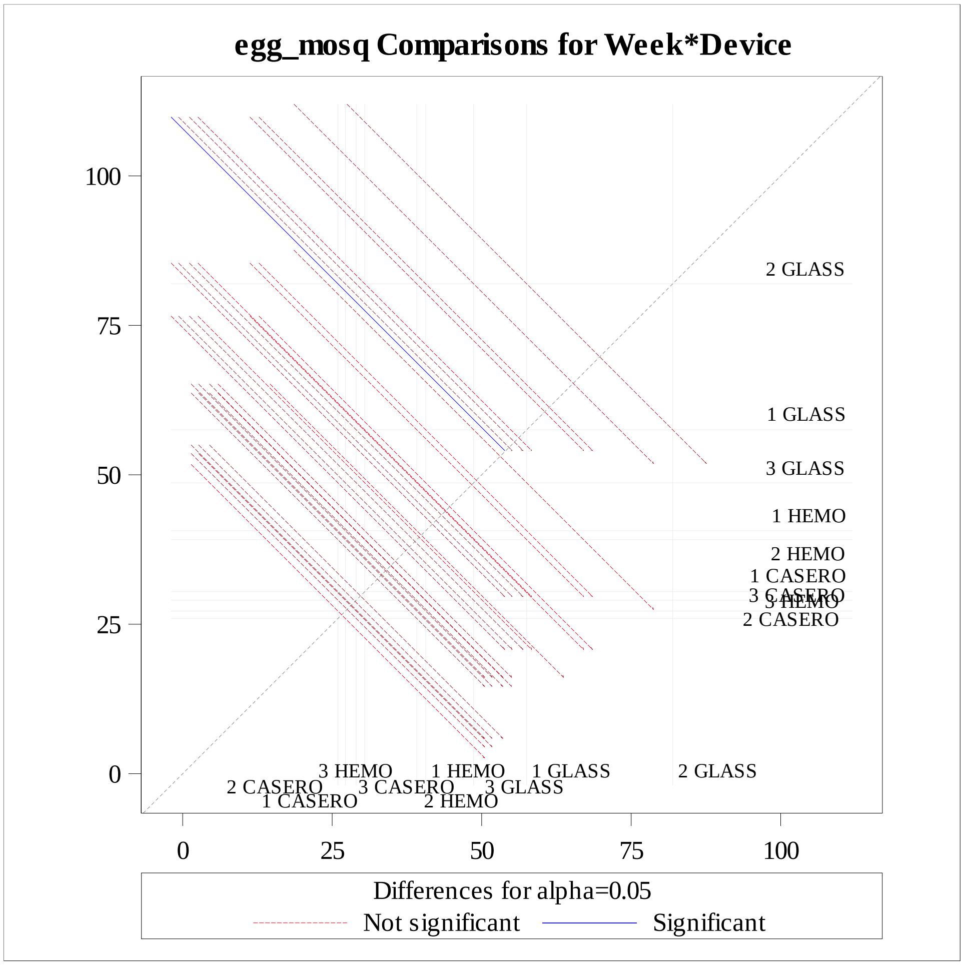

Note: To ensure overall protection level, only probabilities associated with pre-planned comparisons should be used.

The SAS System

The UNIVARIATE Procedure
Variable: RES

Moments			
N	24	Sum Weights	24
Mean	0	Sum Observations	0
Std Deviation	20.9289881	Variance	438.022542
Skewness	-0.005032	Kurtosis	3.06993325
Uncorrected SS	10074.5185	Corrected SS	10074.5185
Coeff Variation	.	Std Error Mean	4.2721118

Basic Statistical Measures			
Location		Variability	
Mean	0.00000	Std Deviation	20.92899
Median	-1.36094	Variance	438.02254
Mode	.	Range	112.92981
		Interquartile Range	15.75080

Tests for Location: Mu0=0				
Test	Statistic		p Value	
Student's t	t	0	Pr > |t|	1.0000
Sign	M	-2	Pr >= |M|	0.5413
Signed Rank	S	-0.5	Pr >= |S|	0.9890

Tests for Normality				
Test	Statistic		p Value	
Shapiro-Wilk	W	0.931403	Pr < W	0.1048
Kolmogorov-Smirnov	D	0.14166	Pr > D	>0.1500
Cramer-von Mises	W-Sq	0.120002	Pr > W-Sq	0.0580
Anderson-Darling	A-Sq	0.699729	Pr > A-Sq	0.0614

The SAS System

The UNIVARIATE Procedure
Variable: RES

Quantiles (Definition 5)	
Level	Quantile
100% Max	56.46491
99%	56.46491
95%	26.73763
90%	22.61564
75% Q3	7.87218
50% Median	-1.36094
25% Q1	-7.87862
10%	-22.61564
5%	-26.73763
1%	-56.46491
0% Min	-56.46491

Extreme Observations			
Lowest		Highest	
Value	Obs	Value	Obs
-56.4649	15	10.6845	6
-26.7376	4	16.8157	16
-22.6156	23	22.6156	20
-16.7068	13	26.7376	7
-13.8961	21	56.4649	12

The SAS System

The UNIVARIATE Procedure

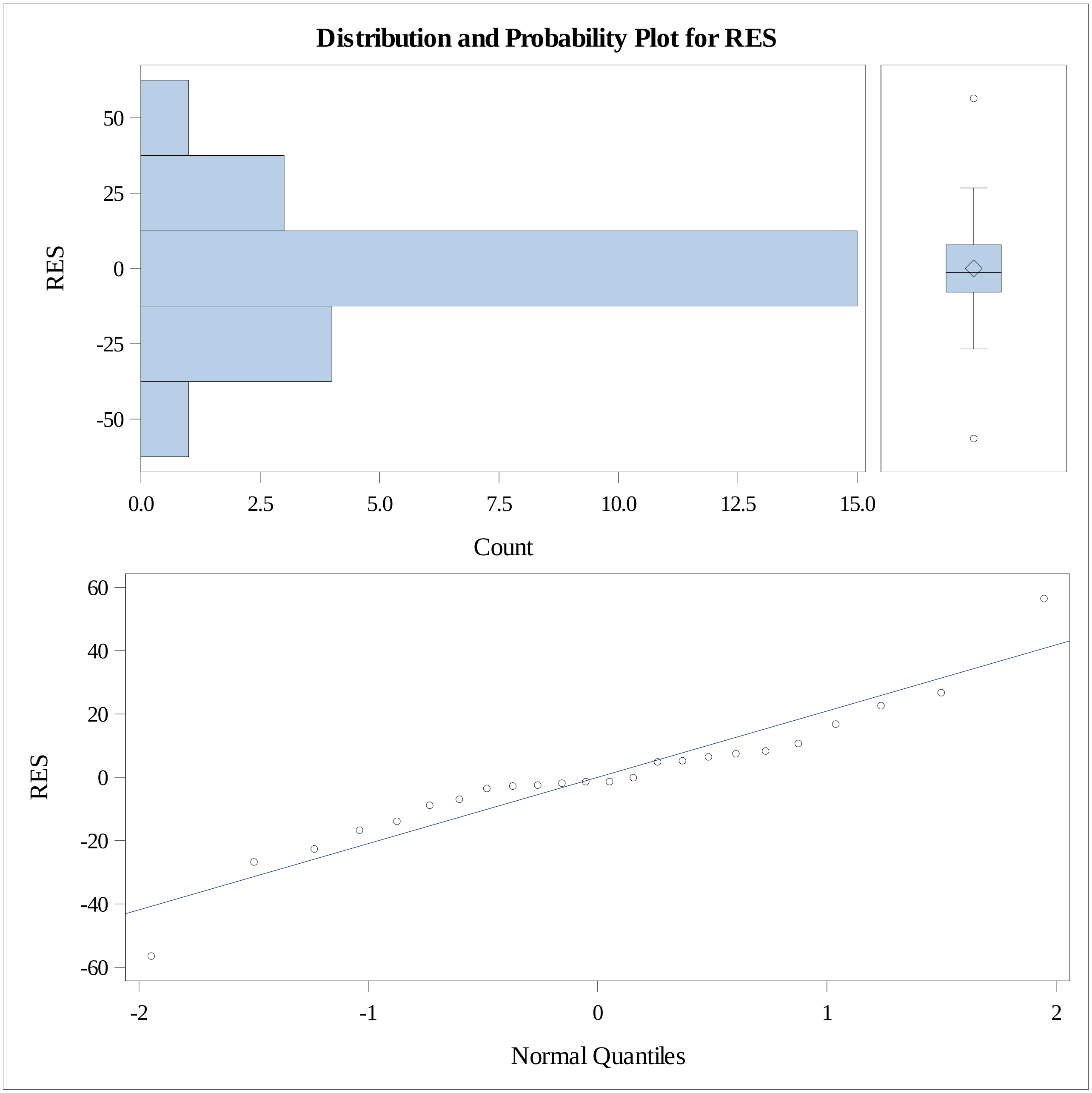

The SAS System

The MEANS Procedure

Analysis Variable : egg_mosq egg_mosq						
**Device**	**N Obs**	**N**	**Mean**	**Std Dev**	**Minimum**	**Maximum**
CASERO	9	9	27.8694103	8.1041380	16.2210526	39.5568182
GLASS	9	9	58.8732128	42.3831008	9.6333333	143.2857143
HEMO	9	9	36.2693563	12.2129322	19.6250000	50.0000000

The SAS System

The MEANS Procedure

Analysis Variable : mosq mosq						
**Device**	**N Obs**	**N**	**Mean**	**Std Dev**	**Minimum**	**Maximum**
CASERO	9	9	82.5555556	13.6208582	64.0000000	97.0000000
GLASS	9	9	15.4444444	12.3299545	2.0000000	31.0000000
HEMO	9	9	27.7777778	10.3896316	14.0000000	36.0000000

The SAS System

The MEANS Procedure

Analysis Variable : egg_mosq egg_mosq						
**Device**	**N Obs**	**N**	**Mean**	**Std Dev**	**Minimum**	**Maximum**
CASERO	9	9	27.8694103	8.1041380	16.2210526	39.5568182
GLASS	9	9	58.8732128	42.3831008	9.6333333	143.2857143
HEMO	9	9	36.2693563	12.2129322	19.6250000	50.0000000

The SAS System

The GLM Procedure

Class Level Information		
**Class**	**Levels**	**Values**
**Week**	3	1 2 3
**Repeat**	3	A B C
**Device**	3	CASERO GLASS HEMO

**Number of Observations Read**	27
**Number of Observations Used**	27

The SAS System

The GLM Procedure

Dependent Variable: egg_mosq egg_mosq

Source	DF	Sum of Squares	Mean Square	F Value	Pr > F
**Model**	10	8328.58891	832.85889	1.08	0.4325
**Error**	16	12388.87866	774.30492		
**Corrected Total**	26	20717.46757			

R-Square	Coeff Var	Root MSE	egg_mosq Mean
0.402008	67.86250	27.82633	41.00399

Source	DF	Type III SS	Mean Square	F Value	Pr > F
**Week**	2	562.420194	281.210097	0.36	0.7011
**Repeat**	2	2886.900532	1443.450266	1.86	0.1872
**Device**	2	4628.187586	2314.093793	2.99	0.0789
**Week*Device**	4	251.080597	62.770149	0.08	0.9871

The SAS System

The GLM Procedure
Least Squares Means

Week	egg_mosq LSMEAN	Standard Error	Pr > |t|	LSMEAN Number
1	45.4303959	9.2754450	0.0002	1
2	42.8590574	9.2754450	0.0003	2
3	34.7225261	9.2754450	0.0018	3

Least Squares Means for Effect Week<br>t for H0: LSMean(i)=LSMean(j) / Pr > |t| <br>Dependent Variable: egg_mosq			
i/j	1	2	3
1		0.196024<br>0.8471	0.816307<br>0.4263
2	-0.19602<br>0.8471		0.620283<br>0.5438
3	-0.81631<br>0.4263	-0.62028<br>0.5438	

The SAS System

The GLM Procedure
Least Squares Means

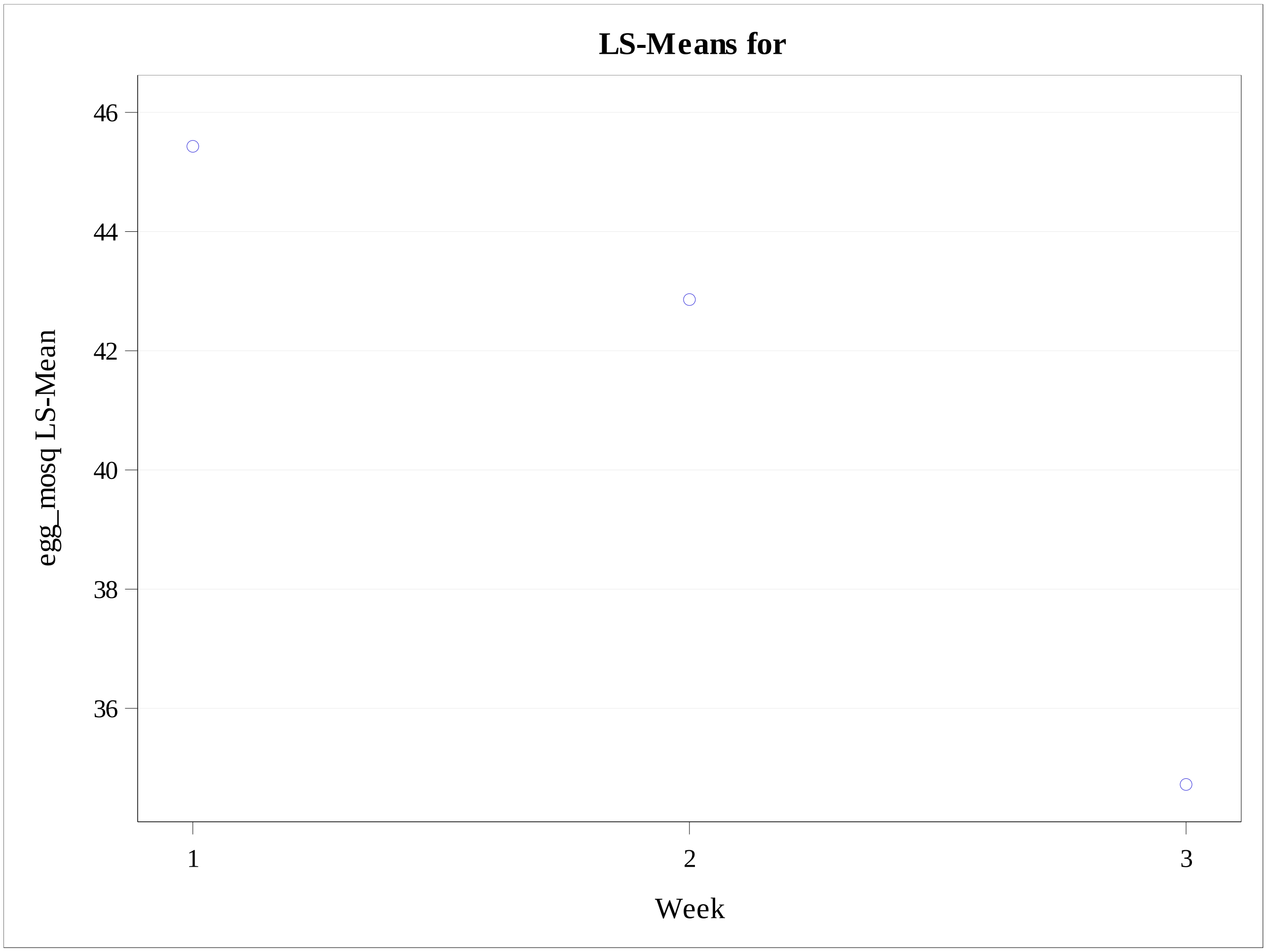

The SAS System

The GLM Procedure
Least Squares Means

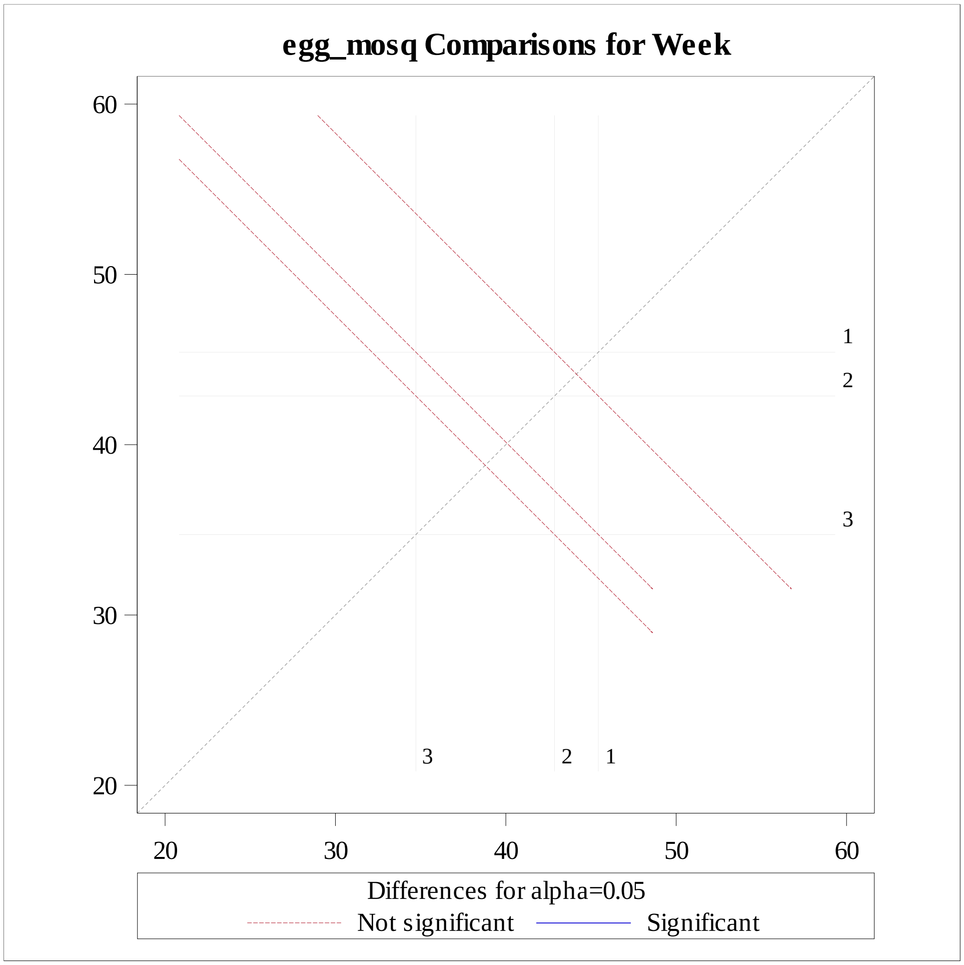

Note: To ensure overall protection level, only probabilities associated with pre-planned comparisons should be used.

The SAS System

The GLM Procedure
Least Squares Means

Device	egg_mosq LSMEAN	Standard Error	Pr > |t|	LSMEAN Number
**CASERO**	27.8694103	9.2754450	0.0084	1
**GLASS**	58.8732128	9.2754450	<.0001	2
**HEMO**	36.2693563	9.2754450	0.0012	3

Least Squares Means for Effect Device<br>t for H0: LSMean(i)=LSMean(j) / Pr > |t| <br>Dependent Variable: egg_mosq			
**i/j**	**1**	**2**	**3**
**1**		-2.36355<br>0.0311	-0.64036<br>0.5310
**2**	2.363552<br>0.0311		1.723189<br>0.1041
**3**	0.640364<br>0.5310	-1.72319<br>0.1041	

The SAS System

The GLM Procedure
Least Squares Means

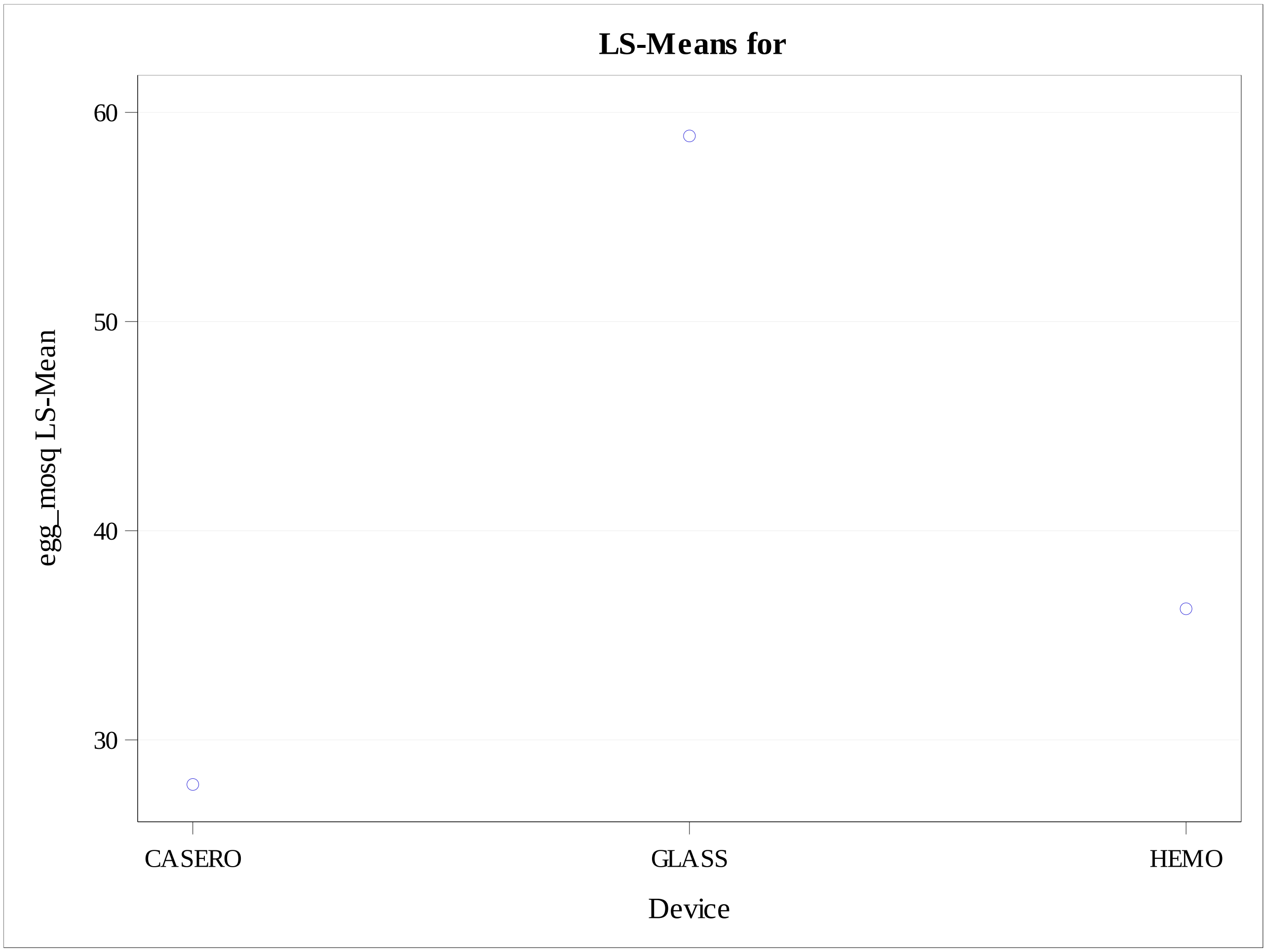

The SAS System

The GLM Procedure
Least Squares Means

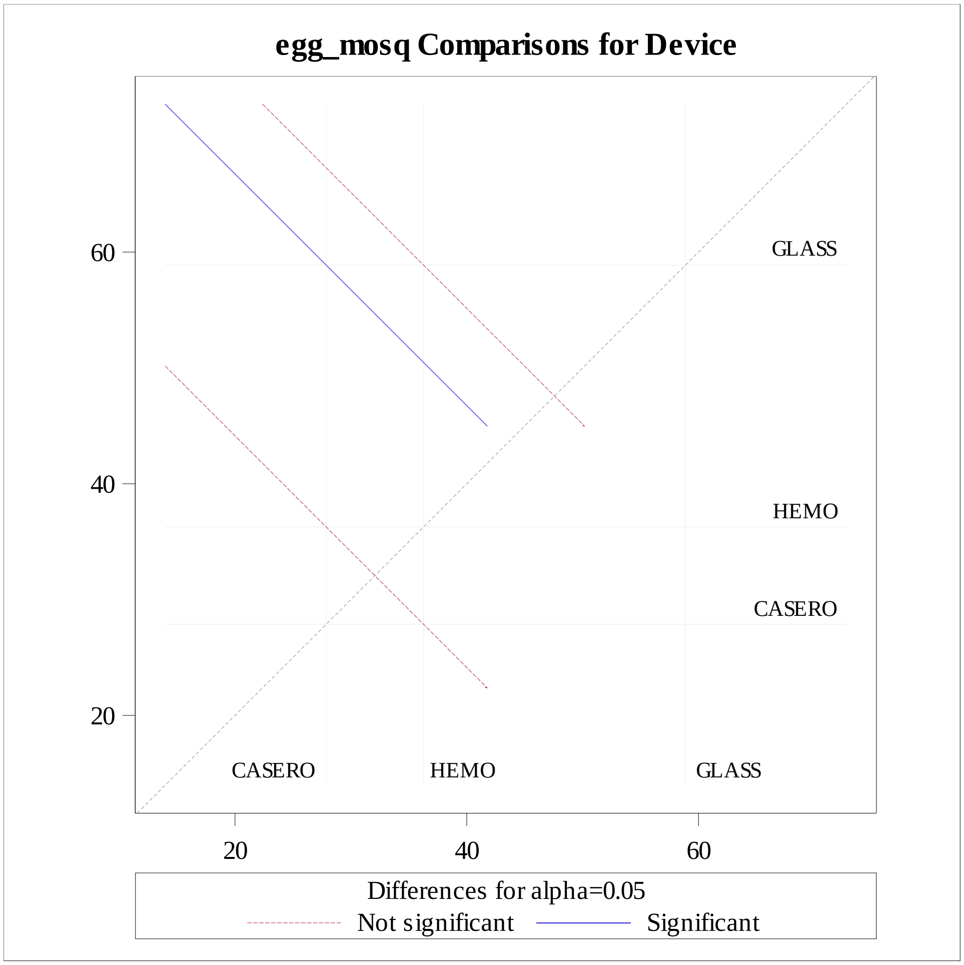

Note: To ensure overall protection level, only probabilities associated with pre-planned comparisons should be used.

The SAS System

The GLM Procedure
Least Squares Means

Repeat	egg_mosq LSMEAN	Standard Error	Pr > |t|	LSMEAN Number
A	49.4124930	9.2754450	<.0001	1
B	47.1610274	9.2754450	0.0001	2
C	26.4384590	9.2754450	0.0116	3

Least Squares Means for Effect Repeat
t for H0: LSMean(i)=LSMean(j) / Pr > |t|

Dependent Variable: egg_mosq

i/j	1	2	3
1		0.171639<br>0.8659	1.751409<br>0.0990
2	-0.17164<br>0.8659		1.57977<br>0.1337
3	-1.75141<br>0.0990	-1.57977<br>0.1337	

The SAS System

The GLM Procedure
Least Squares Means

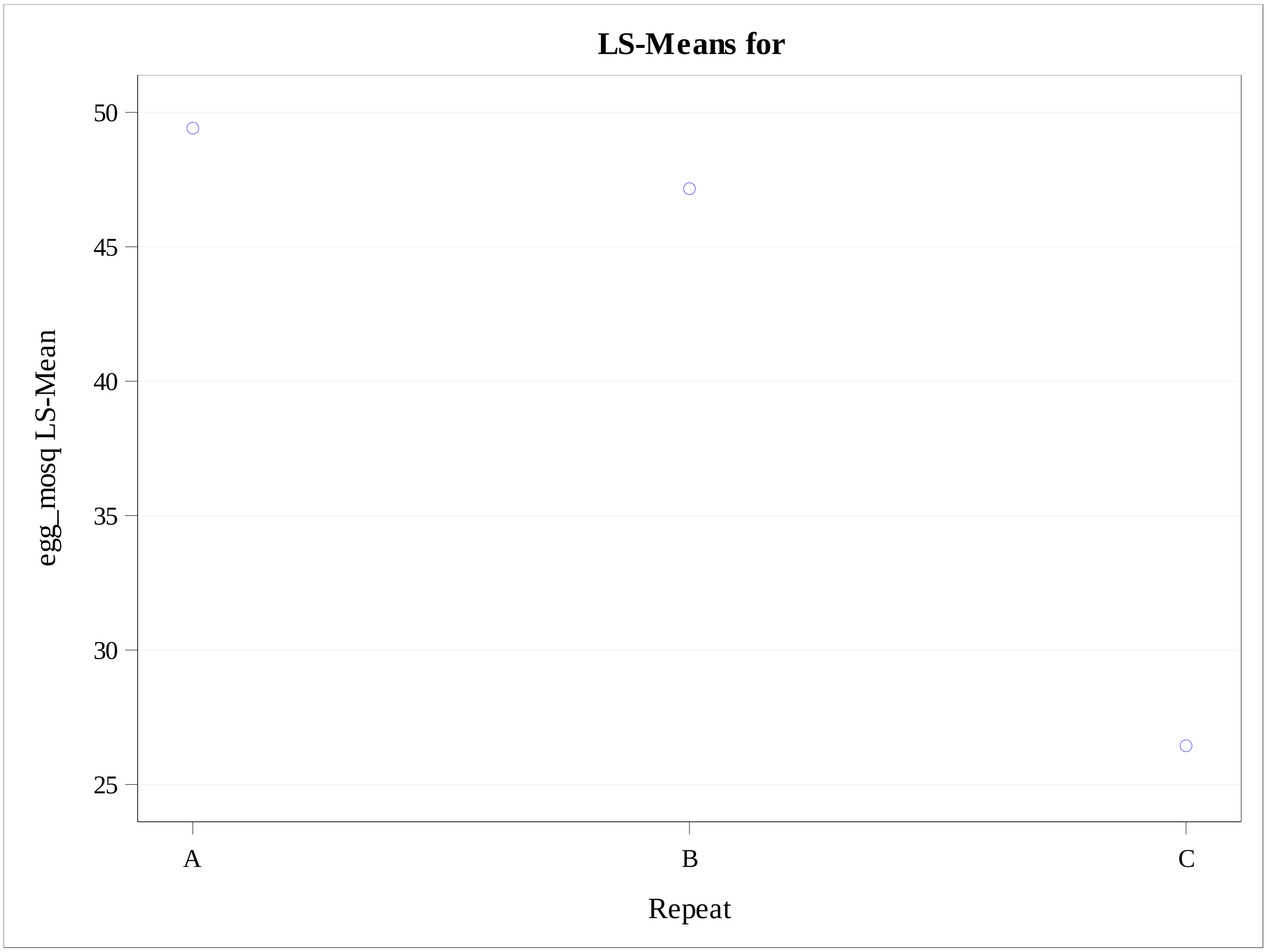

The SAS System

The GLM Procedure
Least Squares Means

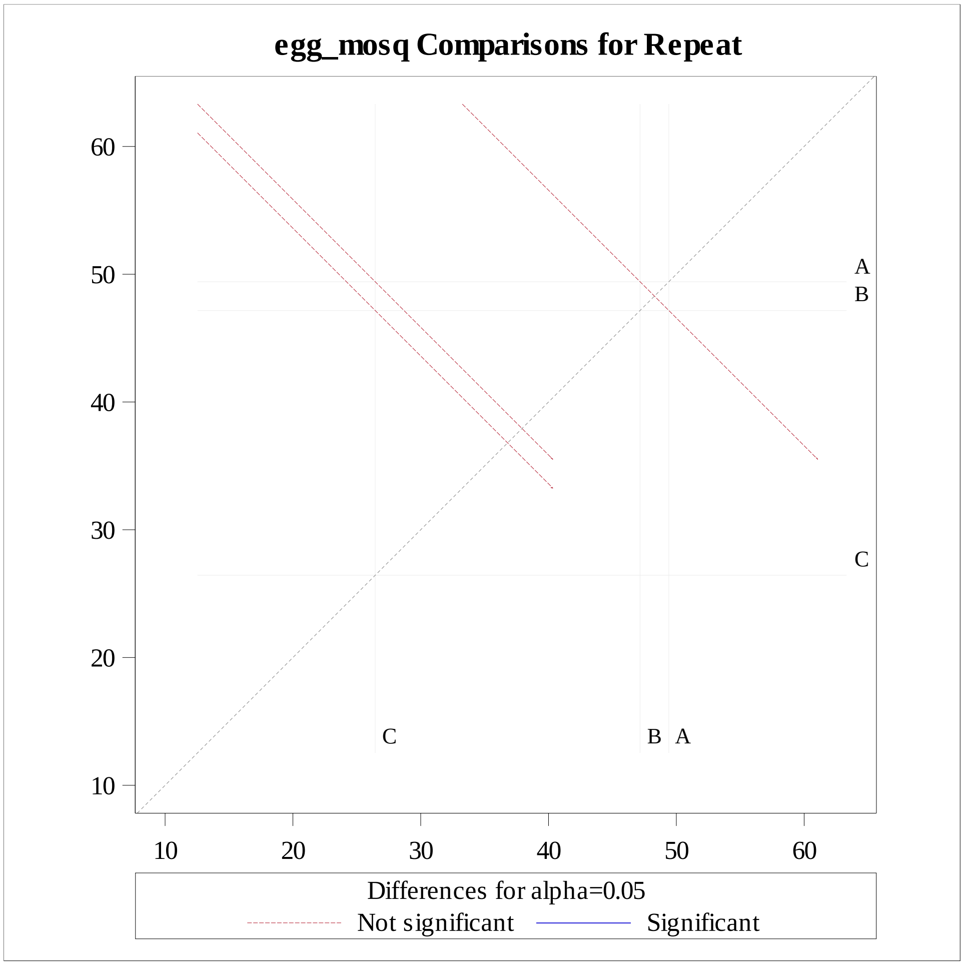

Note: To ensure overall protection level, only probabilities associated with pre-planned comparisons should be used.

The SAS System

The GLM Procedure
Least Squares Means

Week	Device	egg_mosq LSMEAN	Standard Error	Pr > |t|	LSMEAN Number
**1**	**CASERO**	30.4484927	16.0655420	0.0763	1
**1**	**GLASS**	65.1953405	16.0655420	0.0009	2
**1**	**HEMO**	40.6473545	16.0655420	0.0223	3
**2**	**CASERO**	25.9612990	16.0655420	0.1256	4
**2**	**GLASS**	63.4730159	16.0655420	0.0011	5
**2**	**HEMO**	39.1428571	16.0655420	0.0269	6
**3**	**CASERO**	27.1984391	16.0655420	0.1098	7
**3**	**GLASS**	47.9512821	16.0655420	0.0088	8
**3**	**HEMO**	29.0178571	16.0655420	0.0897	9

Least Squares Means for Effect Week*Device
t for H0: LSMean(i)=LSMean(j) / Pr > |t|

Dependent Variable: egg_mosq

i/j	1	2	3	4	5	6	7	8	9
**1**		-1.52934<br>0.1457	-0.44889<br>0.6595	0.197499<br>0.8459	-1.45354<br>0.1654	-0.38267<br>0.7070	0.143047<br>0.8880	-0.77037<br>0.4523	0.062968<br>0.9506
**2**	1.529343<br>0.1457		1.080452<br>0.2960	1.726842<br>0.1034	0.075806<br>0.9405	1.146671<br>0.2684	1.672391<br>0.1139	0.758978<br>0.4589	1.592311<br>0.1309
**3**	0.448891<br>0.6595	-1.08045<br>0.2960		0.64639<br>0.5272	-1.00465<br>0.3300	0.066219<br>0.9480	0.591939<br>0.5622	-0.32147<br>0.7520	0.511859<br>0.6157
**4**	-0.1975<br>0.8459	-1.72684<br>0.1034	-0.64639<br>0.5272		-1.65104<br>0.1182	-0.58017<br>0.5699	-0.05445<br>0.9572	-0.96786<br>0.3475	-0.13453<br>0.8947
**5**	1.453537<br>0.1654	-0.07581<br>0.9405	1.004646<br>0.3300	1.651036<br>0.1182		1.070865<br>0.3001	1.596585<br>0.1299	0.683172<br>0.5043	1.516505<br>0.1489
**6**	0.382673<br>0.7070	-1.14667<br>0.2684	-0.06622<br>0.9480	0.580171<br>0.5699	-1.07086<br>0.3001		0.52572<br>0.6063	-0.38769<br>0.7034	0.445641<br>0.6618
**7**	-0.14305<br>0.8880	-1.67239<br>0.1139	-0.59194<br>0.5622	0.054451<br>0.9572	-1.59658<br>0.1299	-0.52572<br>0.6063		-0.91341<br>0.3746	-0.08008<br>0.9372
**8**	0.770366<br>0.4523	-0.75898<br>0.4589	0.321474<br>0.7520	0.967864<br>0.3475	-0.68317<br>0.5043	0.387693<br>0.7034	0.913413<br>0.3746		0.833333<br>0.4169
**9**	-0.06297<br>0.9506	-1.59231<br>0.1309	-0.51186<br>0.6157	0.134531<br>0.8947	-1.51651<br>0.1489	-0.44564<br>0.6618	0.08008<br>0.9372	-0.83333<br>0.4169	

The SAS System

The GLM Procedure
Least Squares Means

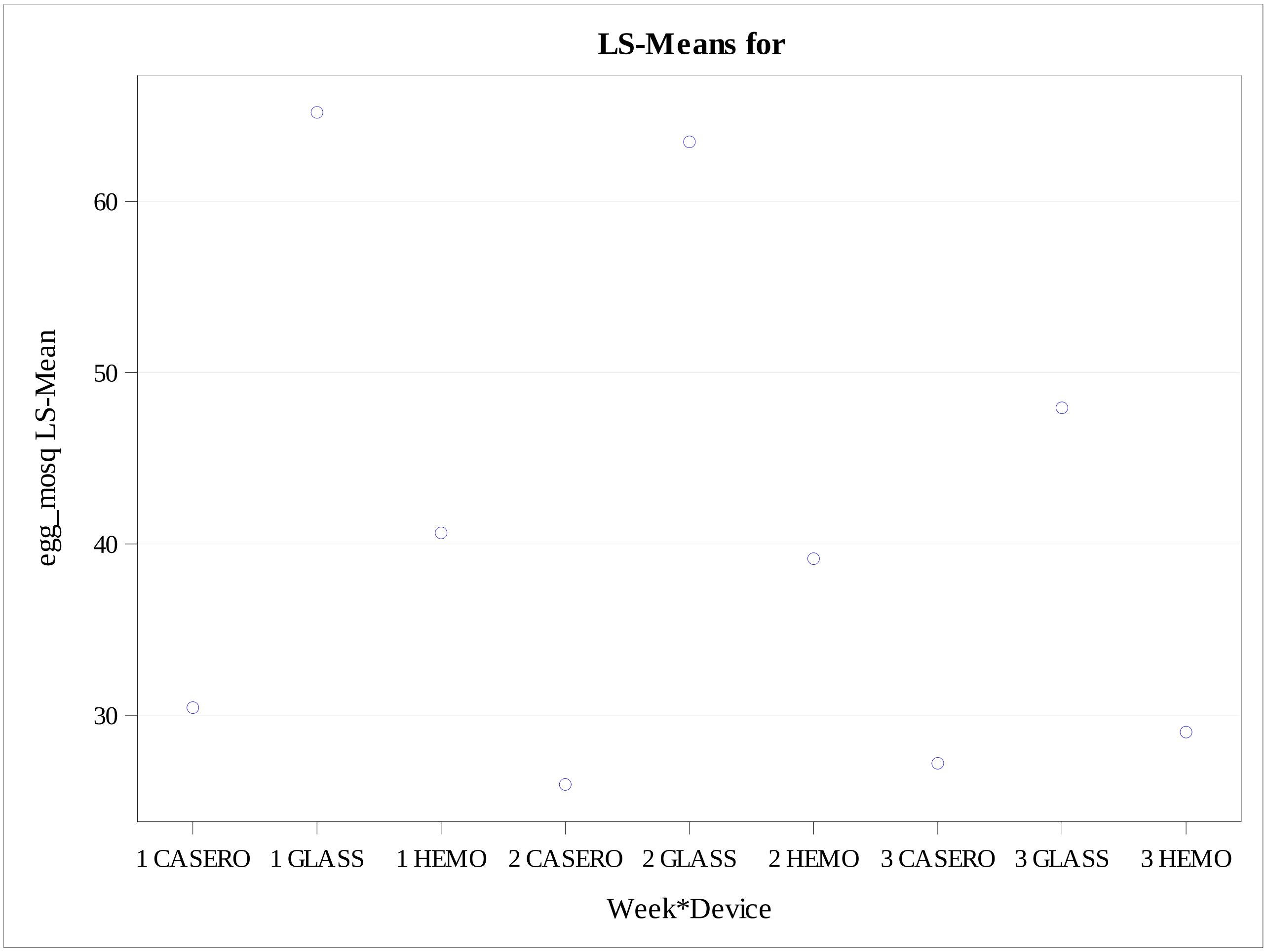

The SAS System

The GLM Procedure
Least Squares Means

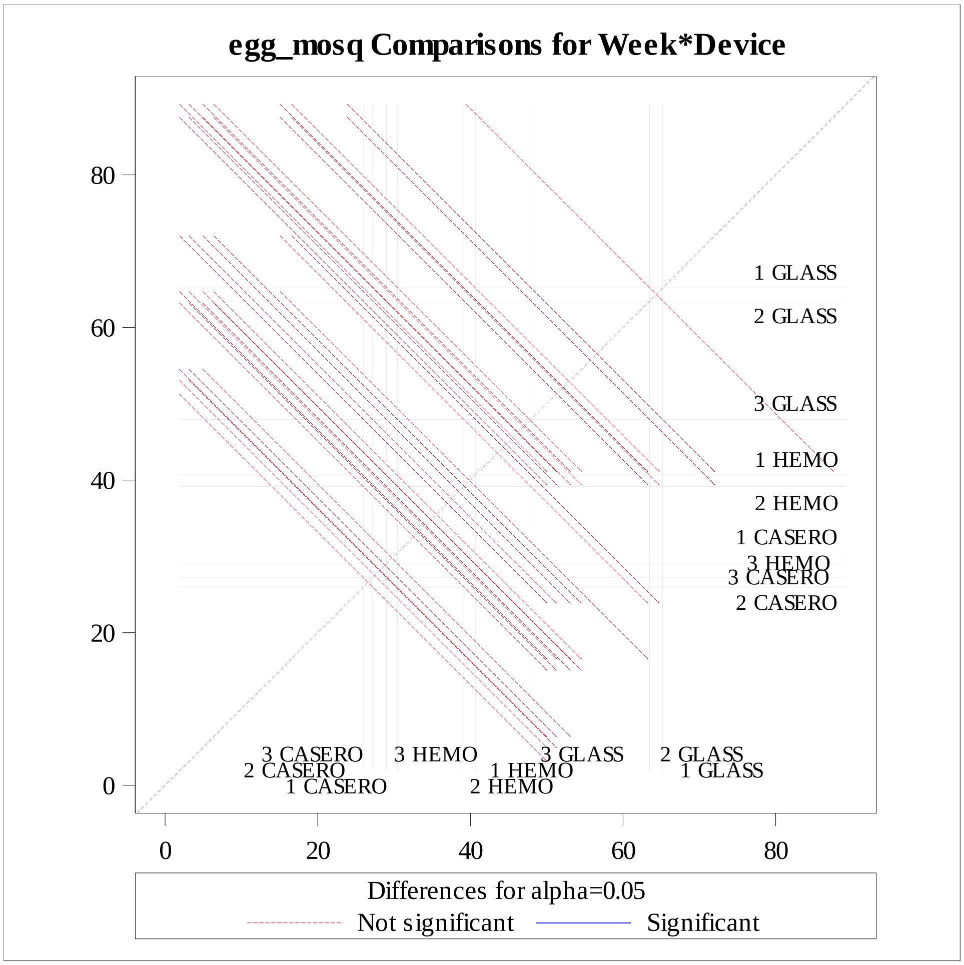

Note: To ensure overall protection level, only probabilities associated with pre-planned comparisons should be used.

The SAS System

The UNIVARIATE Procedure
Variable:
RES

Moments			
**N**	27	**Sum Weights**	27
**Mean**	0	**Sum Observations**	0
**Std Deviation**	21.8287731	**Variance**	476.495333
**Skewness**	1.02904439	**Kurtosis**	4.26368927
**Uncorrected SS**	12388.8787	**Corrected SS**	12388.8787
**Coeff Variation**	.	**Std Error Mean**	4.20094933

Basic Statistical Measures			
**Location**		**Variability**	
**Mean**	0.000000	**Std Deviation**	21.82877
**Median**	1.188341	**Variance**	476.49533
**Mode**	.	**Range**	112.92981
		**Interquartile Range**	19.51123

Tests for Location: Mu0=0				
**Test**	**Statistic**		**p Value**	
**Student's t**	**t**	0	**Pr > |t|**	1.0000
**Sign**	**M**	2.5	**Pr >= |M|**	0.4421
**Signed Rank**	**S**	5	**Pr >= |S|**	0.9070

Tests for Normality				
**Test**	**Statistic**		**p Value**	
**Shapiro-Wilk**	**W**	0.886824	**Pr < W**	0.0068
**Kolmogorov-Smirnov**	**D**	0.138215	**Pr > D**	>0.1500
**Cramer-von Mises**	**W-Sq**	0.143251	**Pr > W-Sq**	0.0274
**Anderson-Darling**	**A-Sq**	0.901962	**Pr > A-Sq**	0.0197

The SAS System

The UNIVARIATE Procedure
Variable: RES

Quantiles (Definition 5)	
**Level**	**Quantile**
**100% Max**	73.65566
**99%**	73.65566
**95%**	22.04553
**90%**	17.89616
**75% Q3**	9.40570
**50% Median**	1.18834
**25% Q1**	-10.10553
**10%**	-34.38152
**5%**	-35.68571
**1%**	-39.27415
**0% Min**	-39.27415

Extreme Observations			
**Lowest**		**Highest**	
**Value**	**Obs**	**Value**	**Obs**
-39.2741	17	15.5370	18
-35.6857	5	17.7895	8
-34.3815	11	17.8962	2
-23.1857	26	22.0455	23
-17.9856	15	73.6557	14

The SAS System

The UNIVARIATE Procedure

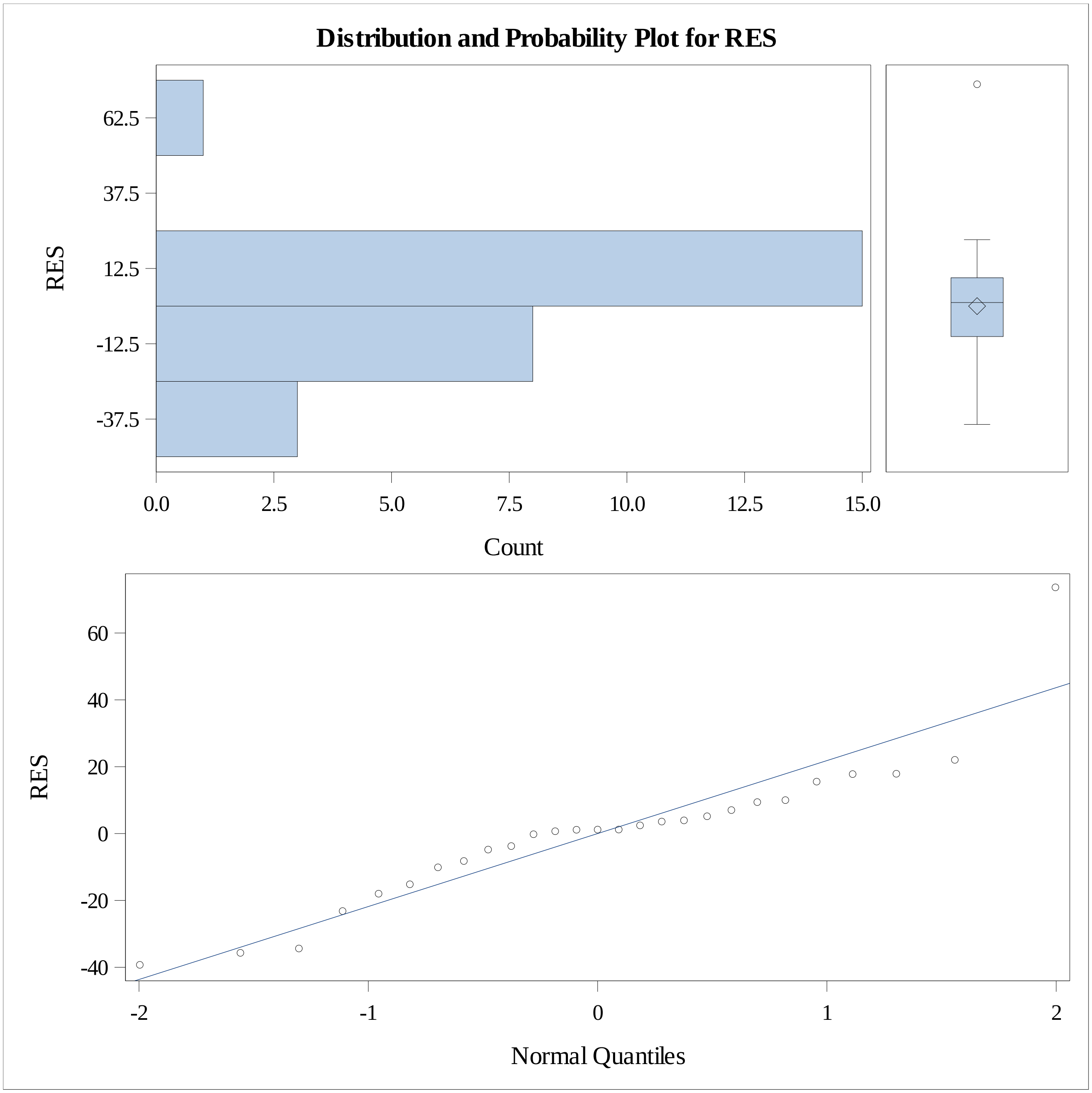

The SAS System

The MEANS Procedure

Analysis Variable : egg_mosq egg_mosq						
**Device**	**N Obs**	**N**	**Mean**	**Std Dev**	**Minimum**	**Maximum**
CASERO	9	9	27.8694103	8.1041380	16.2210526	39.5568182
GLASS	9	9	58.8732128	42.3831008	9.6333333	143.2857143
HEMO	9	9	36.2693563	12.2129322	19.6250000	50.0000000

The SAS System

The MEANS Procedure

Analysis Variable : mosq mosq						
**Device**	**N Obs**	**N**	**Mean**	**Std Dev**	**Minimum**	**Maximum**
CASERO	9	9	82.5555556	13.6208582	64.0000000	97.0000000
GLASS	9	9	15.4444444	12.3299545	2.0000000	31.0000000
HEMO	9	9	27.7777778	10.3896316	14.0000000	36.0000000

The SAS System

The MEANS Procedure

Analysis Variable : egg_mosq egg_mosq						
**Device**	**N Obs**	**N**	**Mean**	**Std Dev**	**Minimum**	**Maximum**
CASERO	9	9	27.8694103	8.1041380	16.2210526	39.5568182
GLASS	9	9	58.8732128	42.3831008	9.6333333	143.2857143
HEMO	9	9	36.2693563	12.2129322	19.6250000	50.0000000

Survival

```
/*Survival rates Experiment #1*/
/*Import S1_File_July2023 Sheet Survival$*/

DATA EXP1_SURVIVE;
SET SURVIVE;
IF EXP = 1;
RUN;
```

Replicates with <10 mosquitoes removed.

```
ODS RTF FILE='Survivalalldata.RTF';
/*Import survive*/
PROC GLM DATA=EXP1_SURVIVE;
CLASS  repeat Device;
MODEL SR =  repeat Device /SS3;
OUTPUT OUT=R RESIDUAL = RES;
LSMEANS device / STDERR PDIFF TDIFF;
LSMEANS repeat / STDERR PDIFF TDIFF;
*LSMEANS repeat*DEVICE / STDERR PDIFF TDIFF;
*LSMEANS Day*Device / STDERR PDIFF TDIFF;
PROC UNIVARIATE NORMAL PLOT DATA=R; VAR RES; RUN;

proc means data=EXP1_SURVIVE;
var SR;
class device;
run;
ODS RTF CLOSE;
```

All data points included.

```
ODS RTF FILE='Survival.RTF';
/*Import survive*/
PROC GLM DATA=EXP1_SURVIVE;
where include = 1;
CLASS  repeat Device;
MODEL SR =  repeat Device /SS3;
OUTPUT OUT=R RESIDUAL = RES;
LSMEANS device / STDERR PDIFF TDIFF;
LSMEANS repeat / STDERR PDIFF TDIFF;
*LSMEANS repeat*DEVICE / STDERR PDIFF TDIFF;
*LSMEANS Day*Device / STDERR PDIFF TDIFF;
PROC UNIVARIATE NORMAL PLOT DATA=R; VAR RES; RUN;

proc means data=EXP1_SURVIVE;
var SR;
class device;
run;
ODS RTF CLOSE;
```

The SAS System

The GLM Procedure

Class Level Information		
**Class**	**Levels**	**Values**
**Repeat**	3	A B C
**Device**	3	casero glass hemo

**Number of Observations Read**	8
**Number of Observations Used**	8

The SAS System

The GLM Procedure

Dependent Variable: SR
SR

Source	DF	Sum of Squares	Mean Square	F Value	Pr > F
**Model**	4	195.9672483	48.9918121	0.77	0.6081
**Error**	3	189.7891673	63.2630558		
**Corrected Total**	7	385.7564156			

R-Square	Coeff Var	Root MSE	SR Mean
0.508008	9.211465	7.953808	86.34682

Source	DF	Type III SS	Mean Square	F Value	Pr > F
**Repeat**	2	126.4972995	63.2486498	1.00	0.4648
**Device**	2	113.3135701	56.6567851	0.90	0.4955

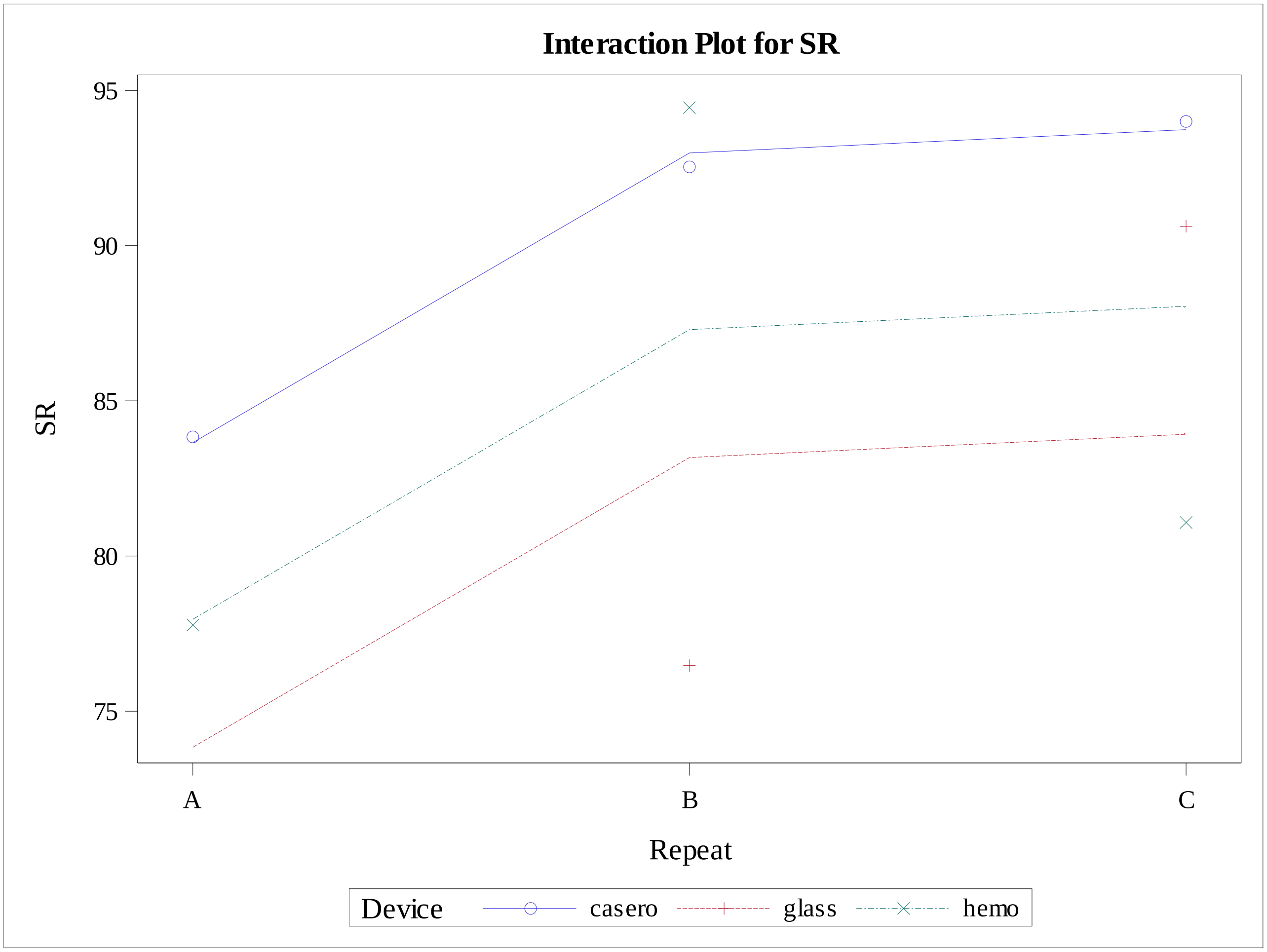

The SAS System

The GLM Procedure
Least Squares Means

Device	SR LSMEAN	Standard Error	Pr > |t|	LSMEAN Number
casero	90.1252324	4.5921330	0.0003	1
glass	80.3119178	6.0748209	0.0009	2
hemo	84.4344344	4.5921330	0.0004	3

Least Squares Means for Effect Device
t for H0: LSMean(i)=LSMean(j) / Pr > |t|

Dependent Variable: SR

i/j	1	2	3
1		1.28865<br>0.2879	0.876282<br>0.4454
2	-1.28865<br>0.2879		-0.54135<br>0.6259
3	-0.87628<br>0.4454	0.541354<br>0.6259	

The SAS System

The GLM Procedure
Least Squares Means

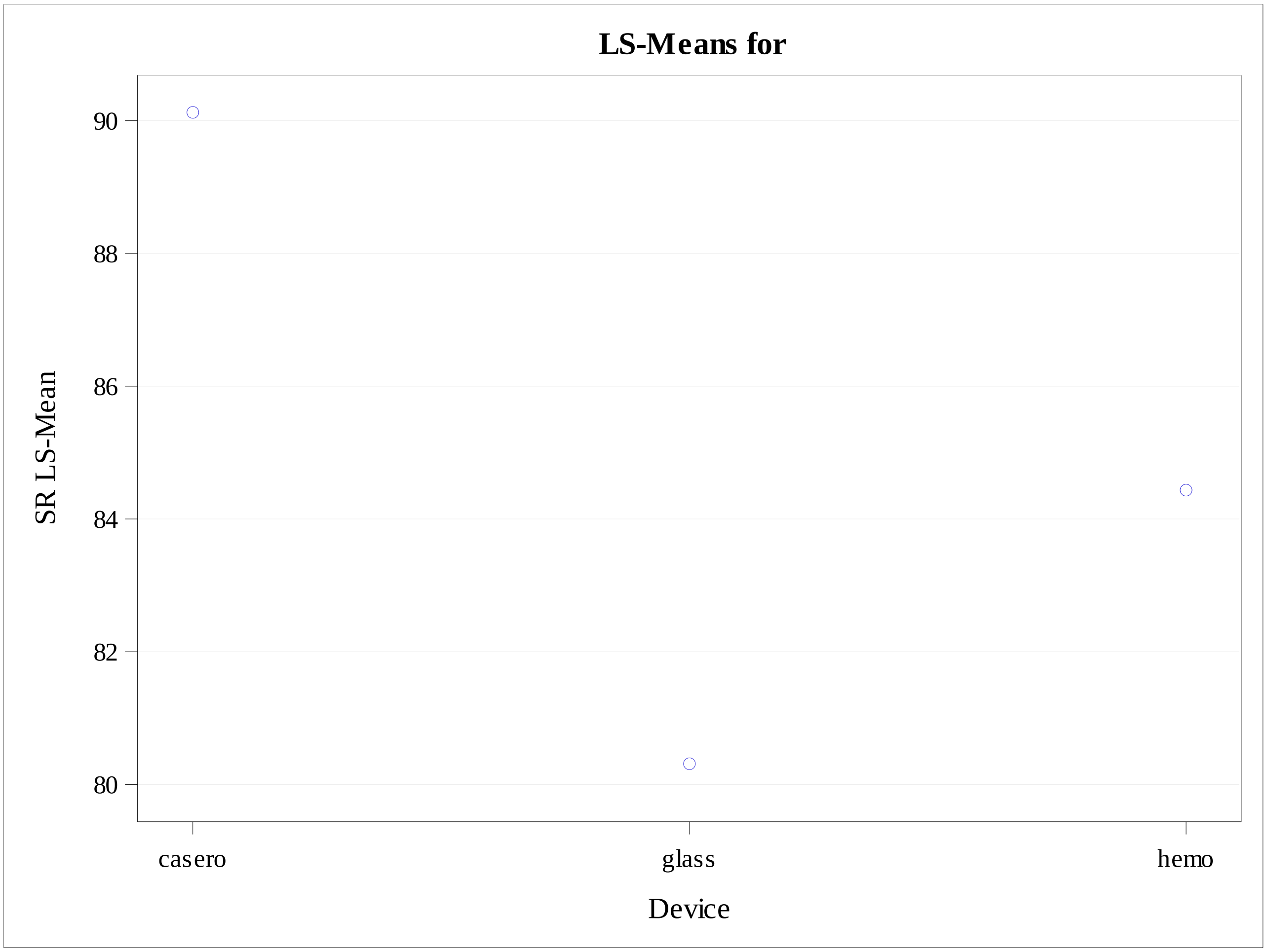

The SAS System

The GLM Procedure
Least Squares Means

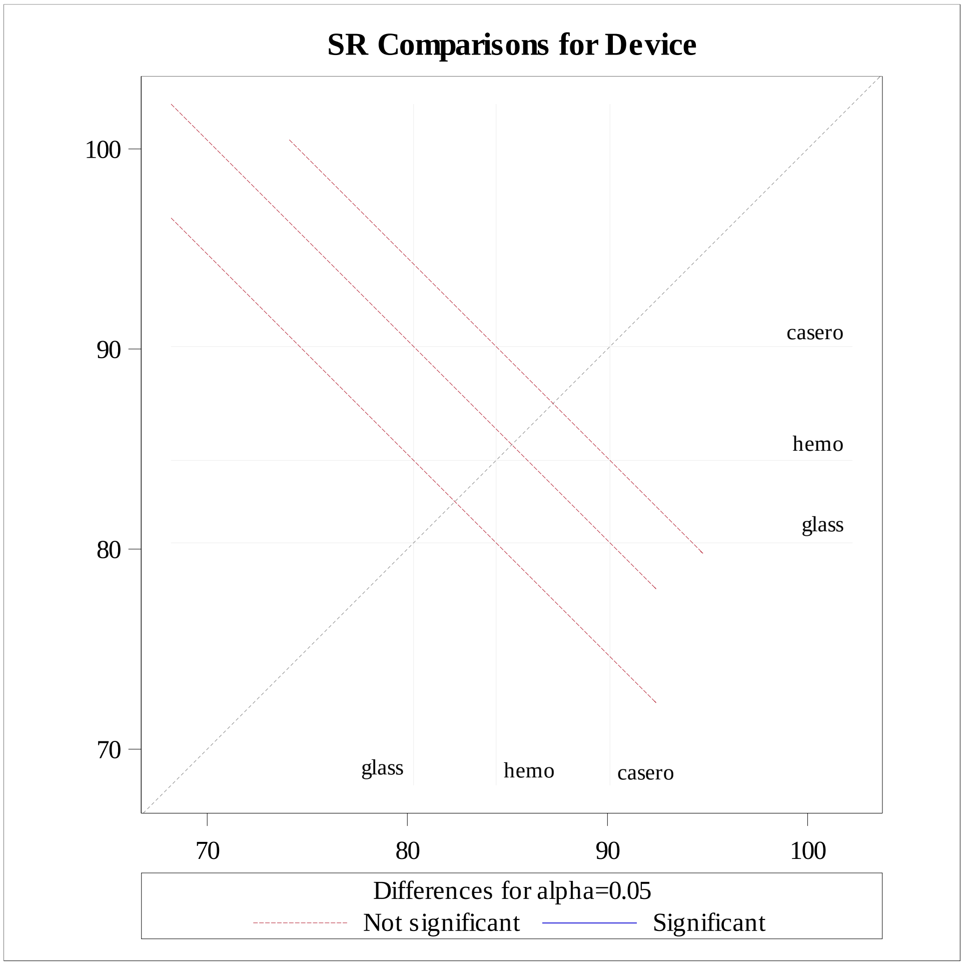

Note: To ensure overall protection level, only probabilities associated with pre-planned comparisons should be used.

The SAS System

The GLM Procedure
Least Squares Means

Repeat	SR LSMEAN	Standard Error	Pr > |t|	LSMEAN Number
**A**	78.4854423	6.0748209	0.0010	1
**B**	87.8174487	4.5921330	0.0003	2
**C**	88.5686937	4.5921330	0.0003	3

Least Squares Means for Effect Repeat
t for H0: LSMean(i)=LSMean(j) / Pr > |t|

Dependent Variable: SR

i/j	1	2	3
**1**		-1.22545<br>0.3078	-1.3241<br>0.2773
**2**	1.225446<br>0.3078		-0.11568<br>0.9152
**3**	1.324097<br>0.2773	0.115678<br>0.9152	

The SAS System

The GLM Procedure
Least Squares Means

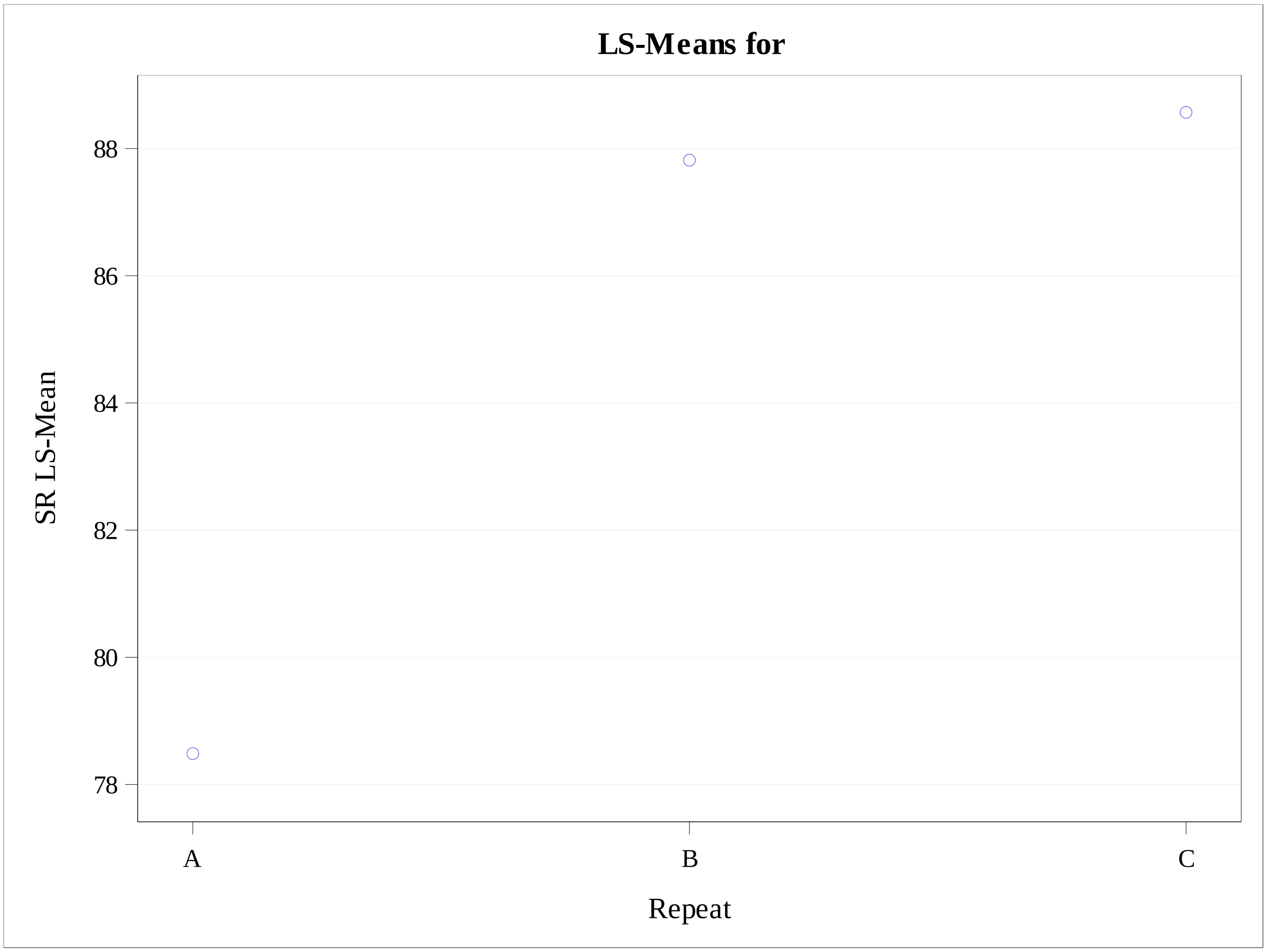

The SAS System

The GLM Procedure
Least Squares Means

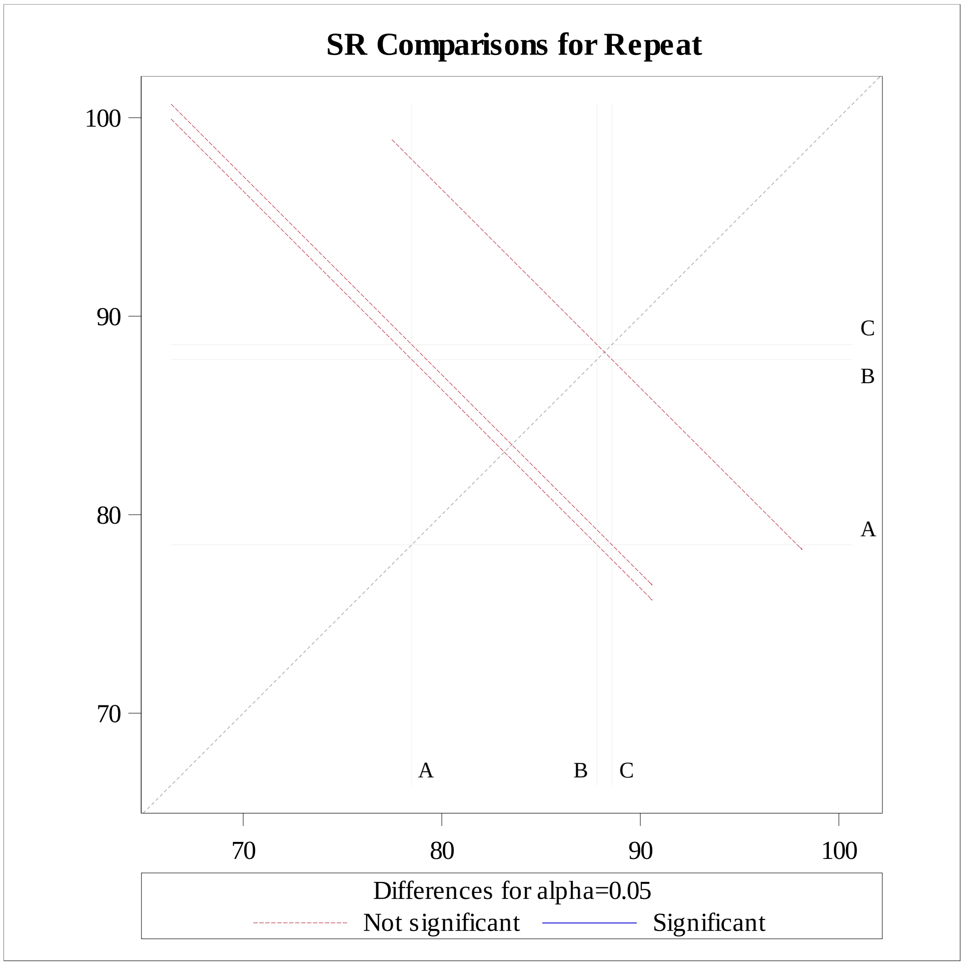

Note: To ensure overall protection level, only probabilities associated with pre-planned comparisons should be used.

The SAS System

The UNIVARIATE Procedure
Variable:
RES

Moments			
**N**	8	**Sum Weights**	8
**Mean**	0	**Sum Observations**	0
**Std Deviation**	5.20698936	**Variance**	27.1127382
**Skewness**	0.03718101	**Kurtosis**	-0.7021485
**Uncorrected SS**	189.789167	**Corrected SS**	189.789167
**Coeff Variation**	.	**Std Error Mean**	1.84094874

Basic Statistical Measures			
**Location**		**Variability**	
**Mean**	0	**Std Deviation**	5.20699
**Median**	-711E-17	**Variance**	27.11274
**Mode**	.	**Range**	14.11461
		**Interquartile Range**	7.05730

Tests for Location: Mu0=0				
**Test**	**Statistic**		**p Value**	
**Student's t**	**t**	0	**Pr > |t|**	1.0000
**Sign**	**M**	0	**Pr >= |M|**	1.0000
**Signed Rank**	**S**	0	**Pr >= |S|**	1.0000

Tests for Normality				
**Test**	**Statistic**		**p Value**	
**Shapiro-Wilk**	**W**	0.880234	**Pr < W**	0.1893
**Kolmogorov-Smirnov**	**D**	0.229838	**Pr > D**	>0.1500
**Cramer-von Mises**	**W-Sq**	0.083498	**Pr > W-Sq**	0.1634
**Anderson-Darling**	**A-Sq**	0.483091	**Pr > A-Sq**	0.1672

The SAS System

The UNIVARIATE Procedure
Variable: RES

Quantiles (Definition 5)	
**Level**	**Quantile**
**100% Max**	7.14976
**99%**	7.14976
**95%**	7.14976
**90%**	7.14976
**75% Q3**	3.48243
**50% Median**	-0.00000
**25% Q1**	-3.57488
**10%**	-6.96485
**5%**	-6.96485
**1%**	-6.96485
**0% Min**	-6.96485

Extreme Observations			
**Lowest**		**Highest**	
**Value**	**Obs**	**Value**	**Obs**
-6.964852	3	-0.184904	1
-6.701583	4	0.184904	6
-0.448173	7	0.263269	8
-0.184904	1	6.701583	5
0.184904	6	7.149756	2

The SAS System

The UNIVARIATE Procedure

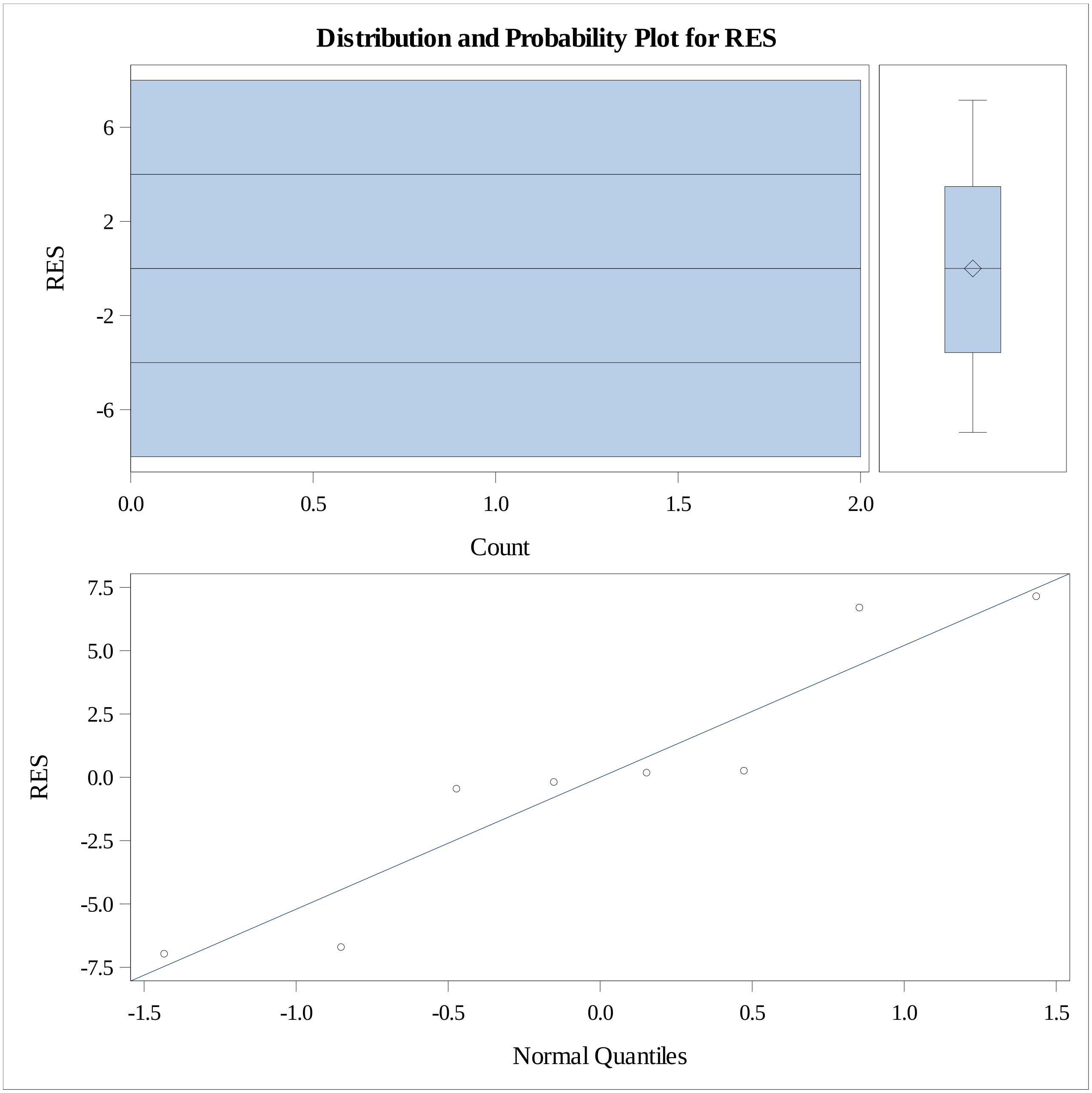

The SAS System

The MEANS Procedure

Analysis Variable : SR SR						
Device	N Obs	N	Mean	Std Dev	Minimum	Maximum
casero	3	3	90.1252324	5.4934699	83.8383838	94.0000000
glass	3	3	89.0318627	11.8453311	76.4705882	100.0000000
hemo	3	3	84.4344344	8.8248614	77.7777778	94.4444444

The SAS System

The GLM Procedure

Class Level Information		
**Class**	**Levels**	**Values**
**Repeat**	3	A B C
**Device**	3	casero glass hemo

**Number of Observations Read**	9
**Number of Observations Used**	9

The SAS System

The GLM Procedure

Dependent Variable: SR
SR

Source	DF	Sum of Squares	Mean Square	F Value	Pr > F
**Model**	4	57.5145790	14.3786447	0.12	0.9696
**Error**	4	493.9389262	123.4847316		
**Corrected Total**	8	551.4535052			

R-Square	Coeff Var	Root MSE	SR Mean
0.104296	12.64726	11.11237	87.86384

Source	DF	Type III SS	Mean Square	F Value	Pr > F
**Repeat**	2	2.79759289	1.39879645	0.01	0.9888
**Device**	2	54.71698608	27.35849304	0.22	0.8105

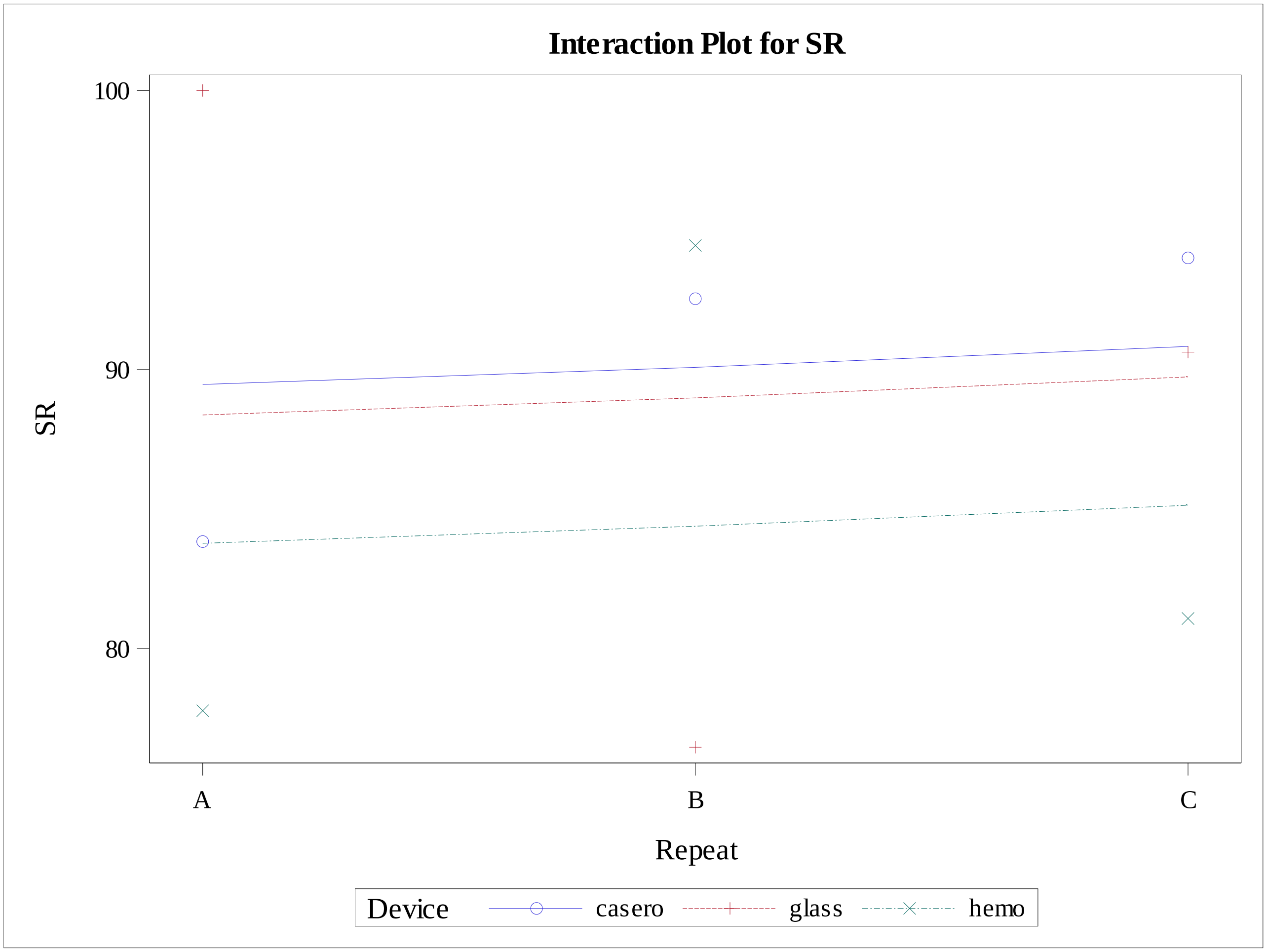

The SAS System

The GLM Procedure
Least Squares Means

Device	SR LSMEAN	Standard Error	Pr > |t|	LSMEAN Number
**casero**	90.1252324	6.4157289	0.0001	1
**glass**	89.0318627	6.4157289	0.0002	2
**hemo**	84.4344344	6.4157289	0.0002	3

Least Squares Means for Effect Device
t for H0: LSMean(i)=LSMean(j) / Pr > |t|

Dependent Variable: SR

i/j	1	2	3
**1**		0.120505<br>0.9099	0.627209<br>0.5645
**2**	-0.12051<br>0.9099		0.506704<br>0.6390
**3**	-0.62721<br>0.5645	-0.5067<br>0.6390	

The SAS System

The GLM Procedure
Least Squares Means

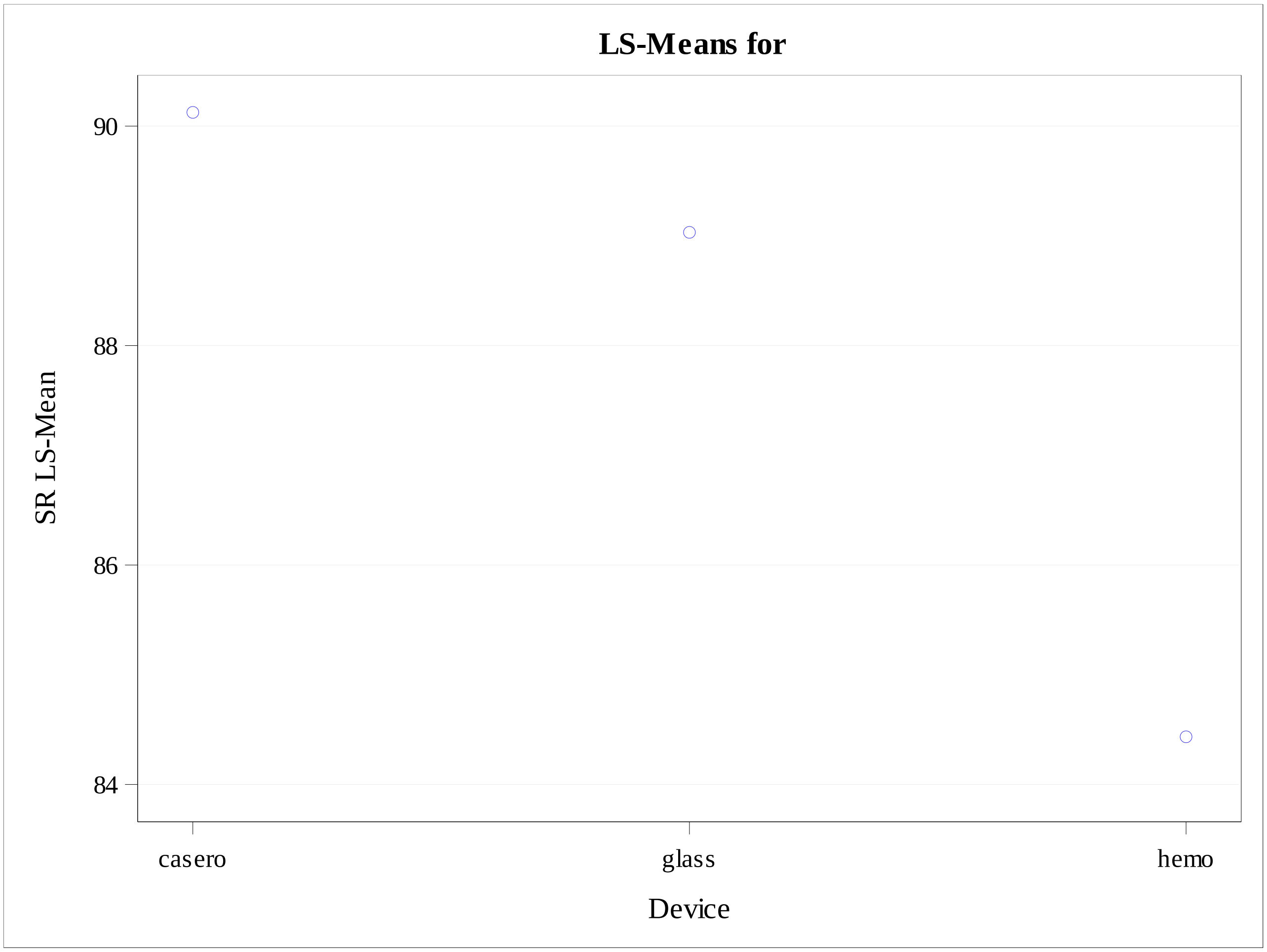

The SAS System

The GLM Procedure
Least Squares Means

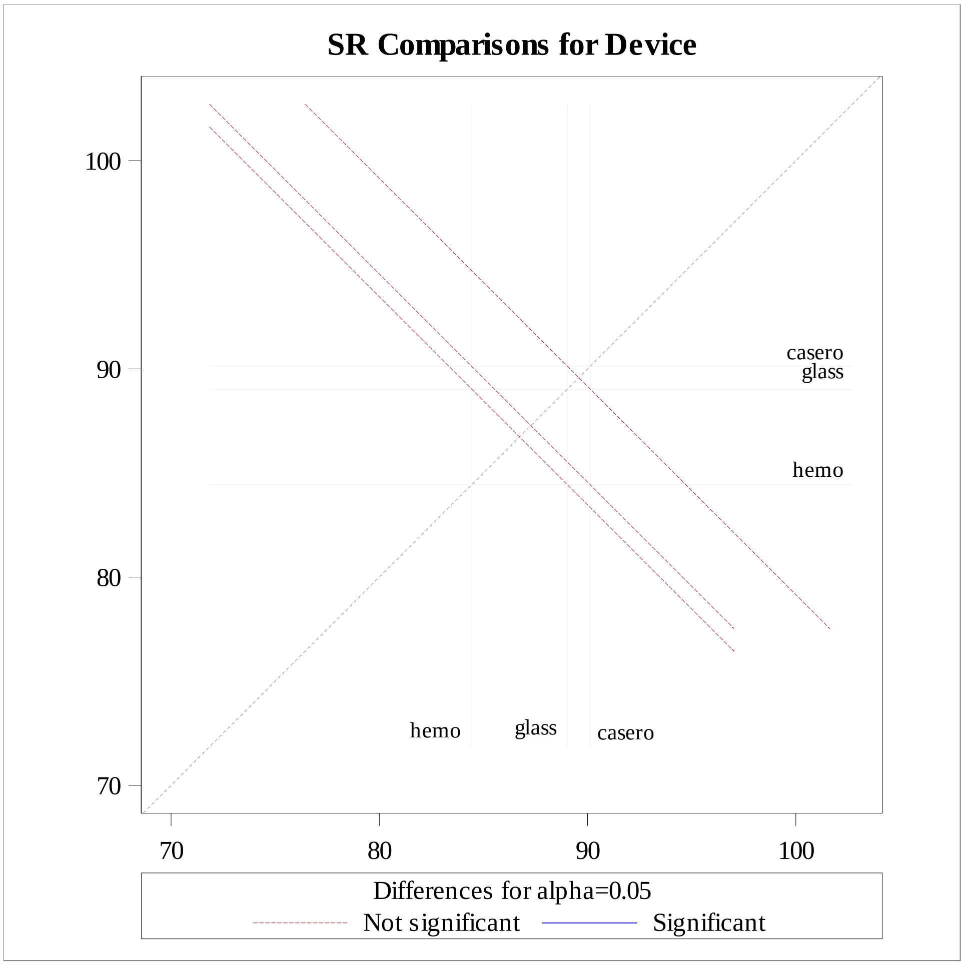

Note: To ensure overall protection level, only probabilities associated with pre-planned comparisons should be used.

The SAS System

The GLM Procedure
Least Squares Means

Repeat	SR LSMEAN	Standard Error	Pr > |t|	LSMEAN Number
A	87.2053872	6.4157289	0.0002	1
B	87.8174487	6.4157289	0.0002	2
C	88.5686937	6.4157289	0.0002	3

Least Squares Means for Effect Repeat
t for H0: LSMean(i)=LSMean(j) / Pr > |t|

Dependent Variable: SR

i/j	1	2	3
1		-0.06746<br>0.9495	-0.15026<br>0.8878
2	0.067458<br>0.9495		-0.0828<br>0.9380
3	0.150256<br>0.8878	0.082798<br>0.9380	

The SAS System

The GLM Procedure
Least Squares Means

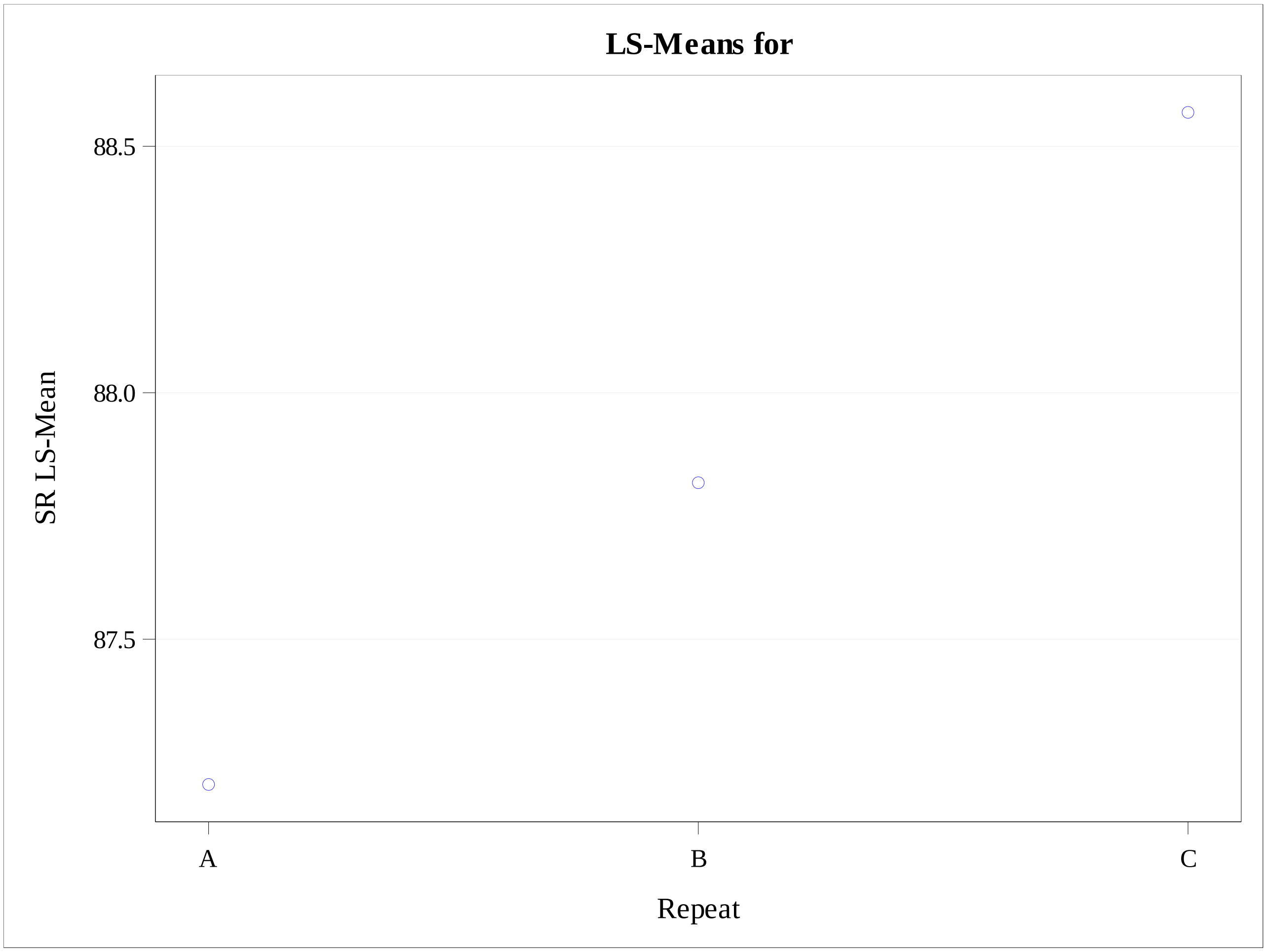

The SAS System

The GLM Procedure
Least Squares Means

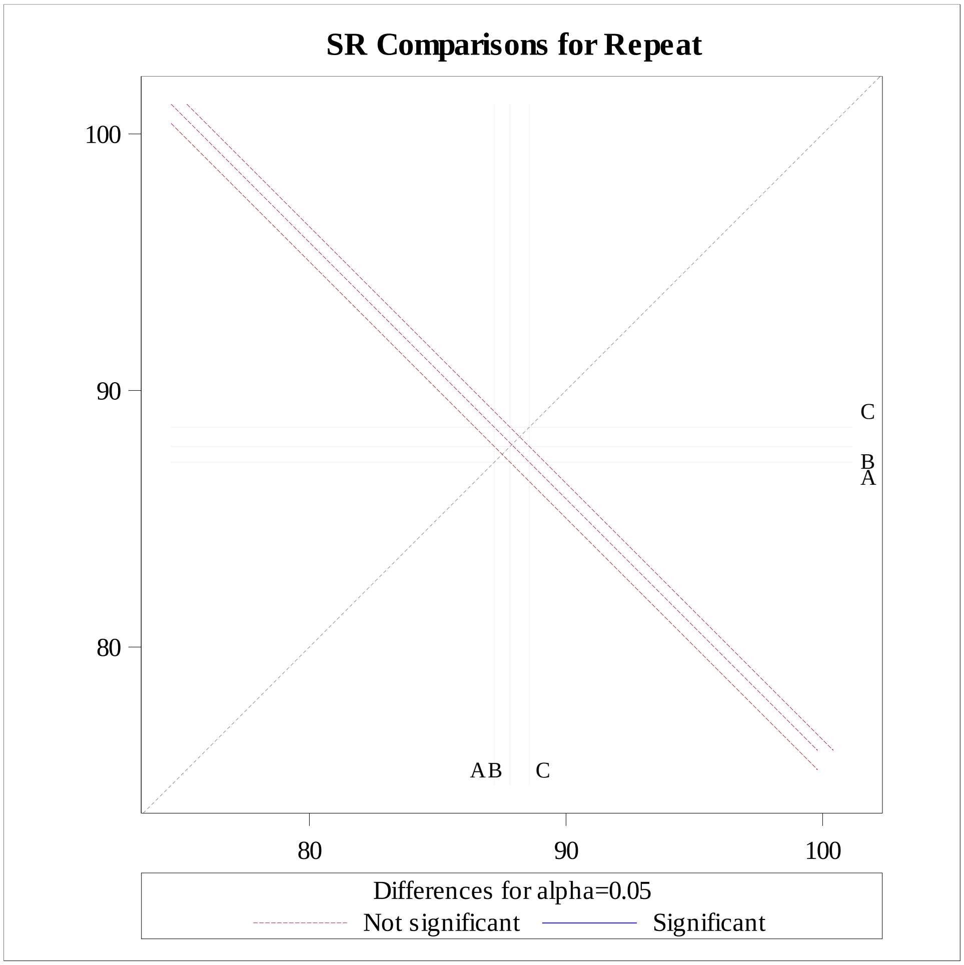

Note: To ensure overall protection level, only probabilities associated with pre-planned comparisons should be used.

The SAS System

The UNIVARIATE Procedure
Variable: RES

Moments			
**N**	9	**Sum Weights**	9
**Mean**	0	**Sum Observations**	0
**Std Deviation**	7.85763105	**Variance**	61.7423658
**Skewness**	0.07123458	**Kurtosis**	-0.654991
**Uncorrected SS**	493.938926	**Corrected SS**	493.938926
**Coeff Variation**	.	**Std Error Mean**	2.61921035

Basic Statistical Measures			
**Location**		**Variability**	
**Mean**	0.000000	**Std Deviation**	7.85763
**Median**	0.888287	**Variance**	61.74237
**Mode**	.	**Range**	24.14147
		**Interquartile Range**	8.79831

Tests for Location: Mu0=0				
**Test**	**Statistic**		**p Value**	
**Student's t**	**t**	0	**Pr > |t|**	1.0000
**Sign**	**M**	0.5	**Pr >= |M|**	1.0000
**Signed Rank**	**S**	-1.5	**Pr >= |S|**	0.9102

Tests for Normality				
**Test**	**Statistic**		**p Value**	
**Shapiro-Wilk**	**W**	0.960448	**Pr < W**	0.8030
**Kolmogorov-Smirnov**	**D**	0.14168	**Pr > D**	>0.1500
**Cramer-von Mises**	**W-Sq**	0.032535	**Pr > W-Sq**	>0.2500
**Anderson-Darling**	**A-Sq**	0.218174	**Pr > A-Sq**	>0.2500

The SAS System

The UNIVARIATE Procedure
Variable: RES

Quantiles (Definition 5)	
**Level**	**Quantile**
**100% Max**	11.626593
**99%**	11.626593
**95%**	11.626593
**90%**	11.626593
**75% Q3**	3.169917
**50% Median**	0.888287
**25% Q1**	-5.628393
**10%**	-12.514880
**5%**	-12.514880
**1%**	-12.514880
**0% Min**	-12.514880

Extreme Observations			
**Lowest**		**Highest**	
**Value**	**Obs**	**Value**	**Obs**
-12.514880	5	0.888287	6
-5.998201	1	2.458476	8
-5.628393	7	3.169917	9
-4.058204	3	10.056405	2
0.888287	6	11.626593	4

The SAS System

The UNIVARIATE Procedure

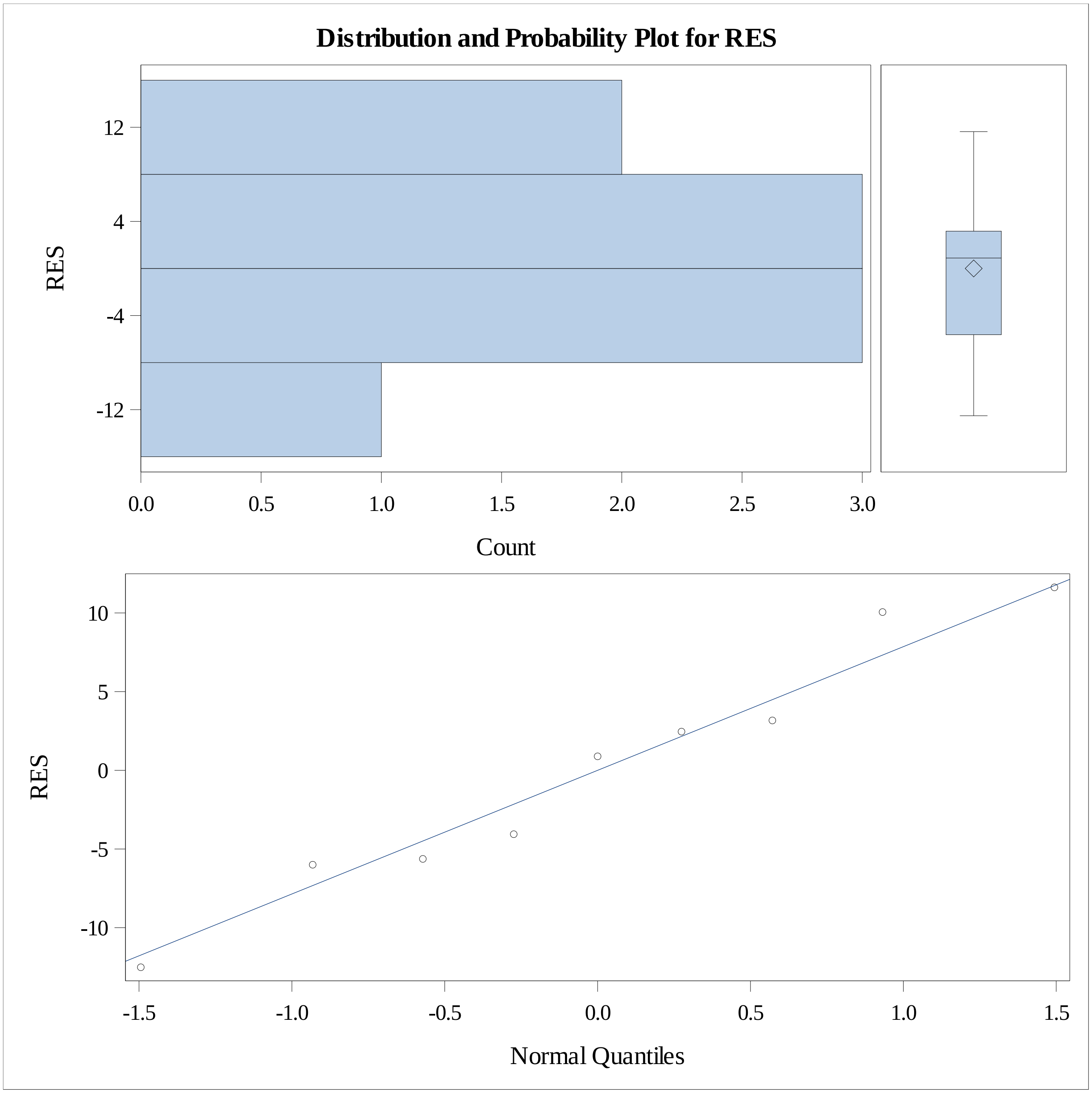

The SAS System

The MEANS Procedure

Analysis Variable : SR SR						
Device	N Obs	N	Mean	Std Dev	Minimum	Maximum
casero	3	3	90.1252324	5.4934699	83.8383838	94.0000000
glass	3	3	89.0318627	11.8453311	76.4705882	100.0000000
hemo	3	3	84.4344344	8.8248614	77.7777778	94.4444444

Experiment #2 General Linear Models (GLM) for:

1. Feeding Rate
2. Egg production
3. Survival

The study design for Experiment #2 in contrast to Experiment #1 did not eliminate mosquitoes that did not feed after being offered blood for 1 hour (Experiment #1 in contrast provided blood for 30 minutes). There were sufficient mosquitoes for all replicates, so a single model is provided for each parameter listed above. We present our SAS code used to analyze S1 Data File followed by model output directly from SAS.

Feeding Rate

```
/*Engorgement rates Experiment #2*/
/*Import S1_File_July2023 Sheet Feed$*/

DATA EXP2_FEED;
SET FEED;
if EXP = 2;
run;

ODS RTF FILE='Feed_exp2.RTF';
PROC GLM DATA=EXP2_FEED;
CLASS  Day Repeat Device ;
MODEL feedrate =  Day Device  day*device /SS3;
OUTPUT OUT=R RESIDUAL = RES;
LSMEANS DAY / STDERR PDIFF TDIFF;
*LSMEANS REPEAT / STDERR PDIFF TDIFF;
LSMEANS DEVICE / STDERR PDIFF TDIFF;
LSMEANS Day*Device / STDERR PDIFF TDIFF;

PROC UNIVARIATE NORMAL PLOT DATA=R; VAR RES; RUN;


proc means data=EXP2_FEED mean std stderr;
var percent;
class day device;
run;
ODS RTF CLOSE;
```

The SAS System

The GLM Procedure

Class Level Information		
**Class**	**Levels**	**Values**
**Day**	4	1 7 14 21
**Repeat**	3	A B C
**Device**	3	casero glass hemo

**Number of Observations Read**	36
**Number of Observations Used**	36

The SAS System

The GLM Procedure

Dependent Variable: feedrate feedrate

Source	DF	Sum of Squares	Mean Square	F Value	Pr > F
**Model**	11	14751.10401	1341.00946	40.86	<.0001
**Error**	24	787.64184	32.81841		
**Corrected Total**	35	15538.74585			

R-Square	Coeff Var	Root MSE	feedrate Mean
0.949311	6.580543	5.728735	87.05566

Source	DF	Type III SS	Mean Square	F Value	Pr > F
**Day**	3	10174.91941	3391.63980	103.35	<.0001
**Device**	2	1382.88733	691.44366	21.07	<.0001
**Day*Device**	6	3193.29727	532.21621	16.22	<.0001

The SAS System

The GLM Procedure

Dependent Variable: feedrate
feedrate

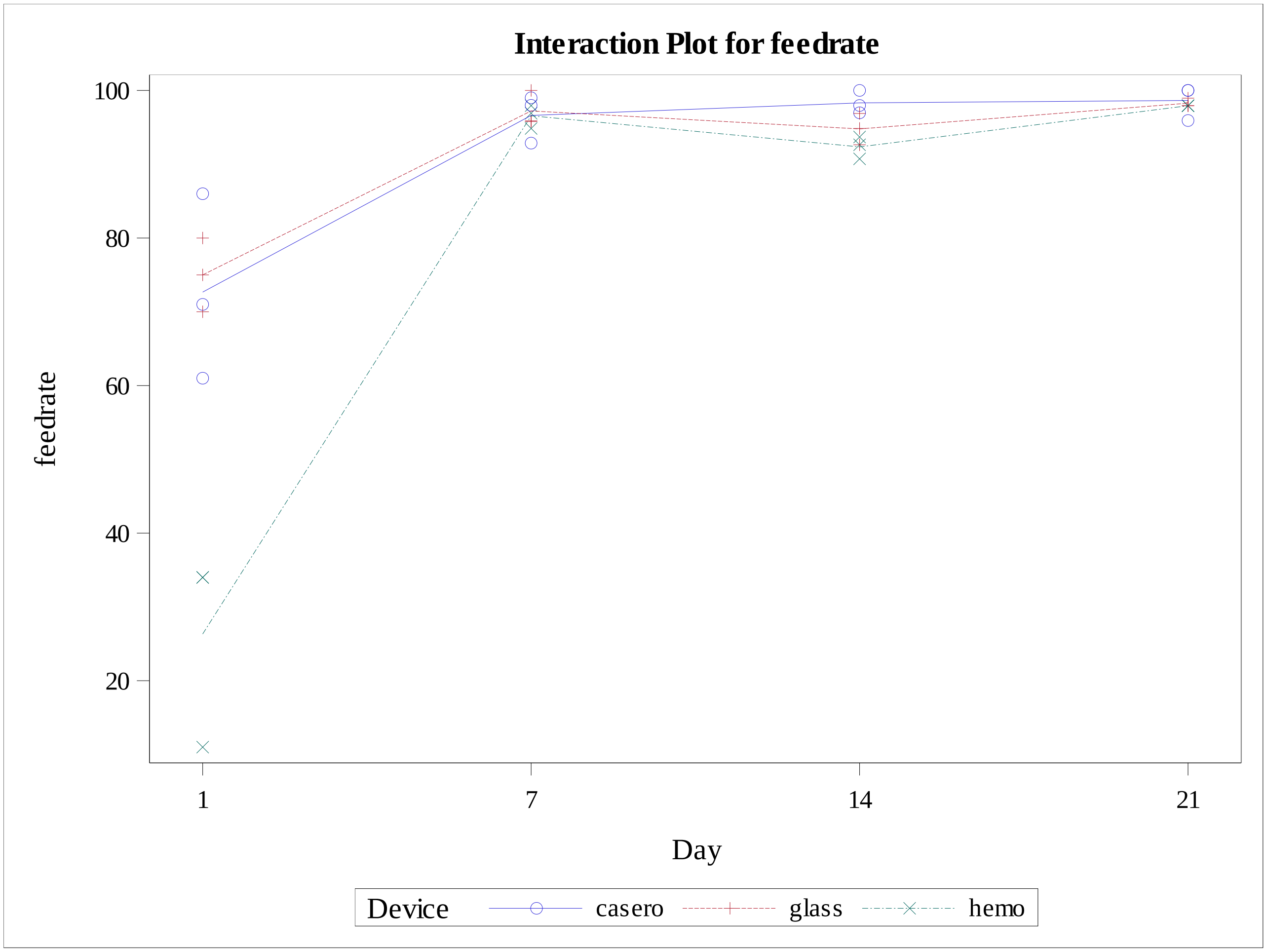

The SAS System

The GLM Procedure
Least Squares Means

Day	feedrate LSMEAN	Standard Error	Pr > |t|	LSMEAN Number
1	58.0000000	1.9095785	<.0001	1
7	96.7992694	1.9095785	<.0001	2
14	95.1464733	1.9095785	<.0001	3
21	98.2768916	1.9095785	<.0001	4

Least Squares Means for Effect Day
t for H0: LSMean(i)=LSMean(j) / Pr > |t|

Dependent Variable: feedrate

i/j	1	2	3	4
1		-14.3672<br><.0001	-13.7551<br><.0001	-14.9143<br><.0001
2	14.36716<br><.0001		0.612022<br>0.5463	-0.54716<br>0.5893
3	13.75514<br><.0001	-0.61202<br>0.5463		-1.15918<br>0.2578
4	14.91432<br><.0001	0.547156<br>0.5893	1.159177<br>0.2578	

The SAS System

The GLM Procedure
Least Squares Means

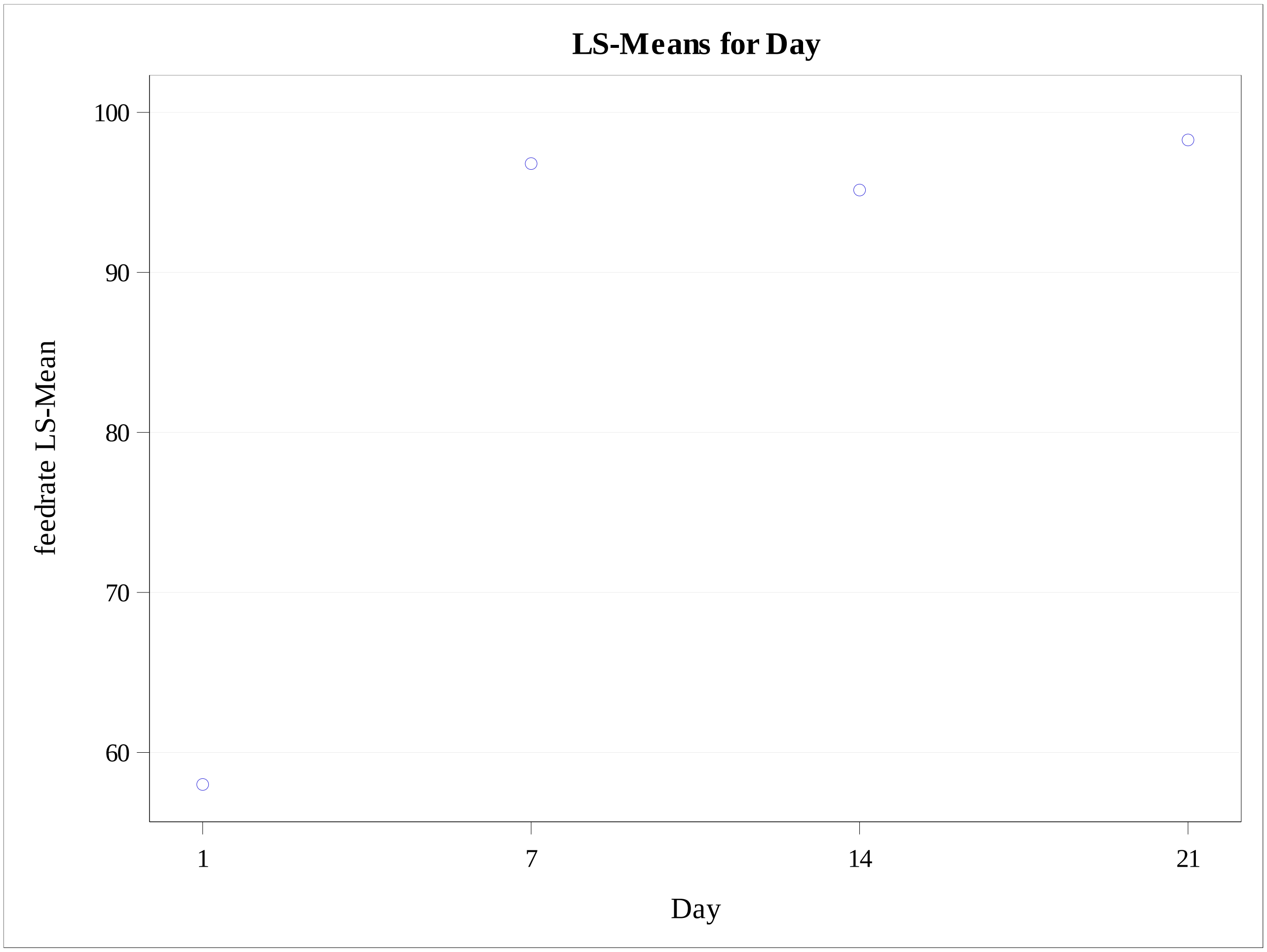

The SAS System

The GLM Procedure
Least Squares Means

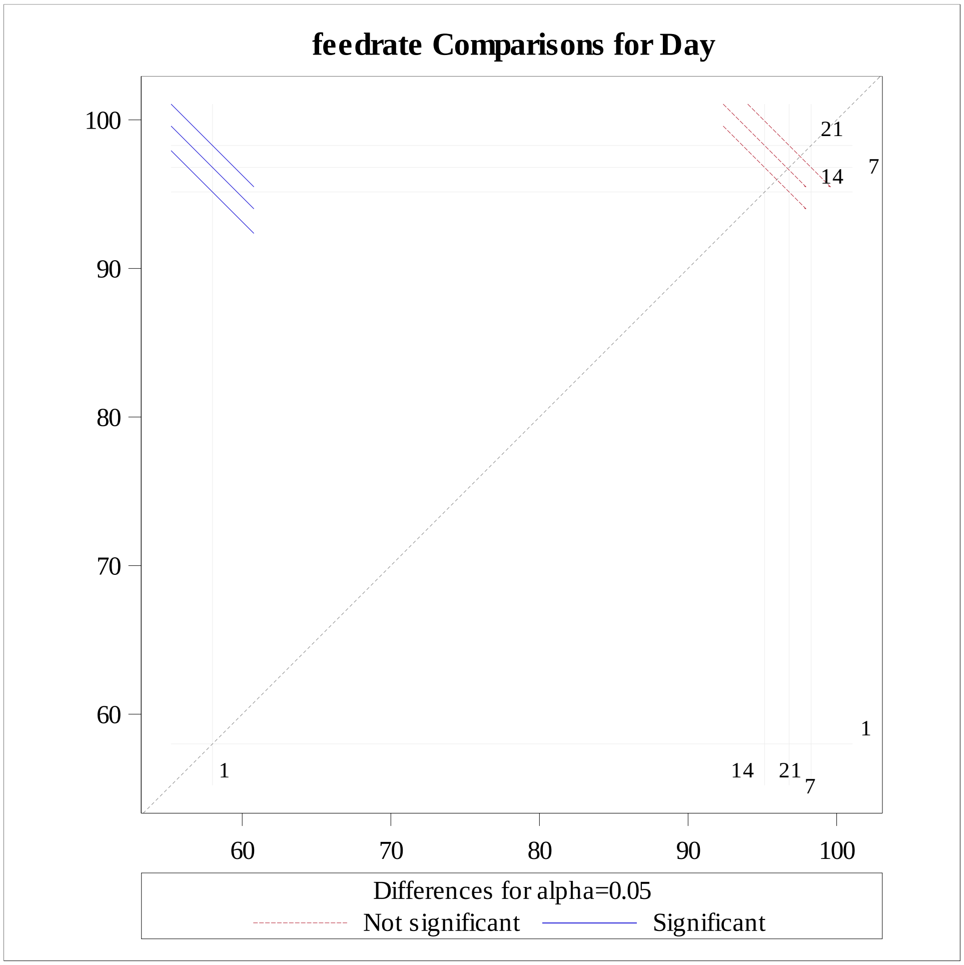

Note: To ensure overall protection level, only probabilities associated with pre-planned comparisons should be used.

The SAS System

The GLM Procedure
Least Squares Means

Device	feedrate LSMEAN	Standard Error	Pr > |t|	LSMEAN Number
casero	91.5570157	1.6537435	<.0001	1
glass	91.3183235	1.6537435	<.0001	2
hemo	78.2916365	1.6537435	<.0001	3

Least Squares Means for Effect Device
t for H0: LSMean(i)=LSMean(j) / Pr > |t|

Dependent Variable: feedrate

i/j	1	2	3
1		0.10206<br>0.9196	5.672004<br><.0001
2	-0.10206<br>0.9196		5.569944<br><.0001
3	-5.672<br><.0001	-5.56994<br><.0001	

The SAS System

The GLM Procedure
Least Squares Means

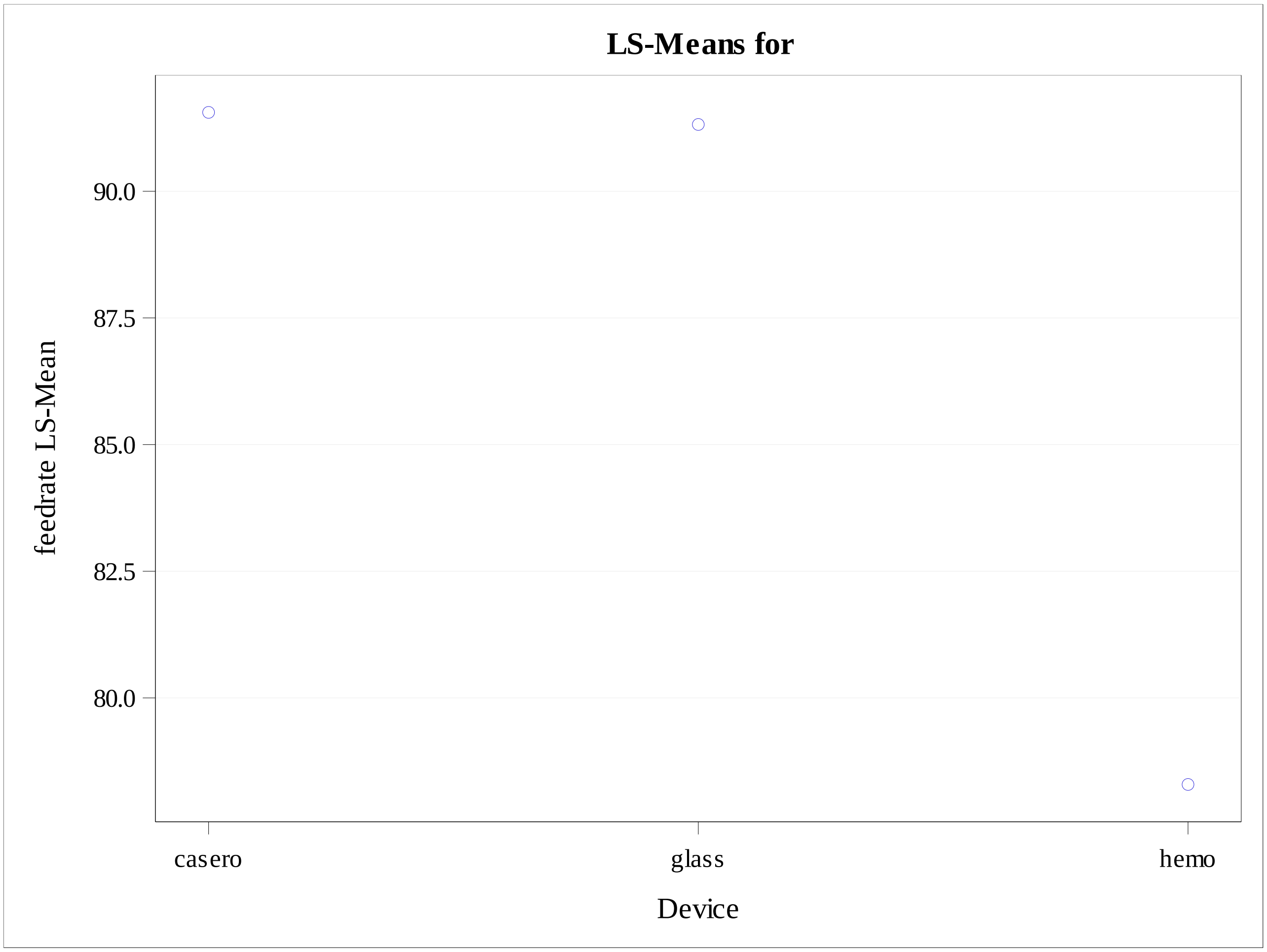

The SAS System

The GLM Procedure
Least Squares Means

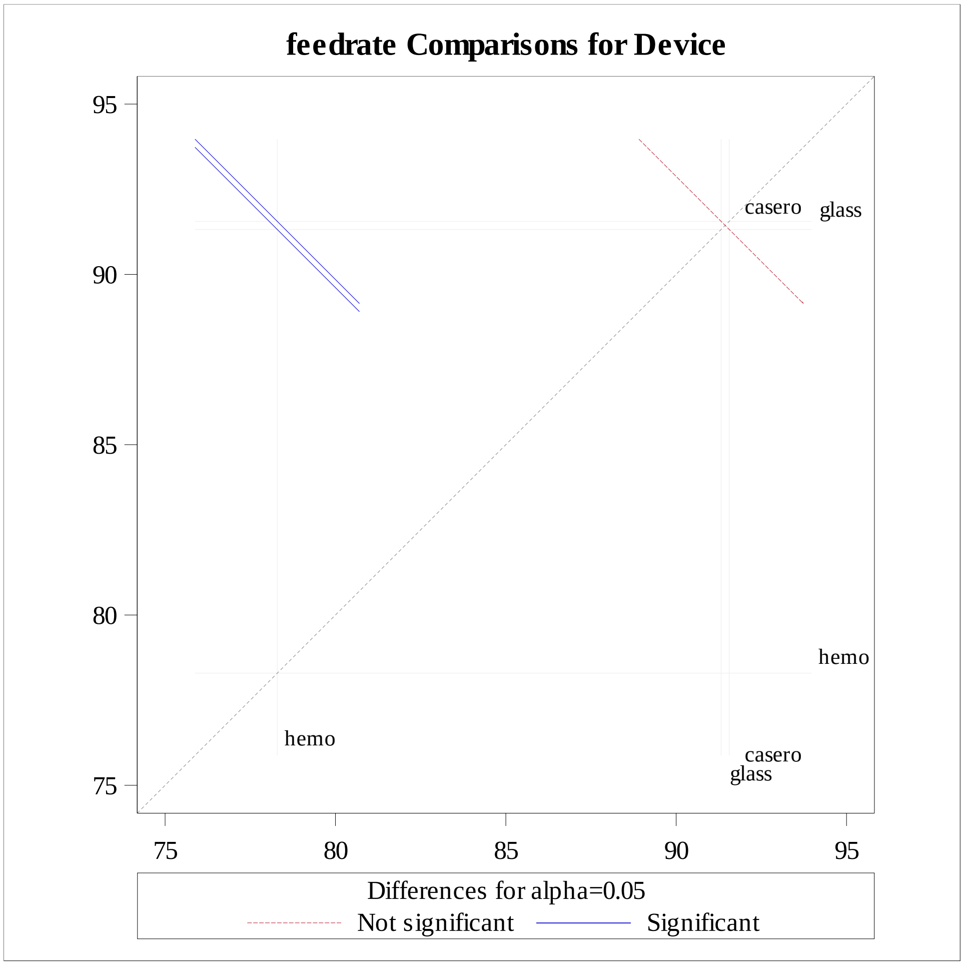

Note: To ensure overall protection level, only probabilities associated with pre-planned comparisons should be used.

The SAS System

The GLM Procedure
Least Squares Means

Day	Device	feedrate LSMEAN	Standard Error	Pr > |t|	LSMEAN Number
**1**	**casero**	72.6666667	3.3074870	<.0001	1
**1**	**glass**	75.0000000	3.3074870	<.0001	2
**1**	**hemo**	26.3333333	3.3074870	<.0001	3
**7**	**casero**	96.6123136	3.3074870	<.0001	4
**7**	**glass**	97.2219208	3.3074870	<.0001	5
**7**	**hemo**	96.5635739	3.3074870	<.0001	6
**14**	**casero**	98.3096269	3.3074870	<.0001	7
**14**	**glass**	94.7839799	3.3074870	<.0001	8
**14**	**hemo**	92.3458130	3.3074870	<.0001	9
**21**	**casero**	98.6394558	3.3074870	<.0001	10
**21**	**glass**	98.2673931	3.3074870	<.0001	11
**21**	**hemo**	97.9238259	3.3074870	<.0001	12

The SAS System

The GLM Procedure
Least Squares Means

Least Squares Means for Effect Day*Device
t for H0: LSMean(i)=LSMean(j) / Pr > |t|

Dependent Variable: feedrate

i/j	1	2	3	4	5	6	7	8	9	10	11	12
**1**		-0.49884<br>0.6224	9.905591<br><.0001	-5.11933<br><.0001	-5.24966<br><.0001	-5.10891<br><.0001	-5.4822<br><.0001	-4.72845<br><.0001	-4.2072<br>0.0003	-5.55272<br><.0001	-5.47317<br><.0001	-5.39972<br><.0001
**2**	0.498843<br>0.6224		10.40443<br><.0001	-4.62049<br>0.0001	-4.75082<br><.0001	-4.61007<br>0.0001	-4.98336<br><.0001	-4.22961<br>0.0003	-3.70836<br>0.0011	-5.05387<br><.0001	-4.97433<br><.0001	-4.90088<br><.0001
**3**	-9.90559<br><.0001	-10.4044<br><.0001		-15.0249<br><.0001	-15.1553<br><.0001	-15.0145<br><.0001	-15.3878<br><.0001	-14.634<br><.0001	-14.1128<br><.0001	-15.4583<br><.0001	-15.3788<br><.0001	-15.3053<br><.0001
**4**	5.119334<br><.0001	4.620491<br>0.0001	15.02492<br><.0001		-0.13033<br>0.8974	0.01042<br>0.9918	-0.36287<br>0.7199	0.390879<br>0.6993	0.912134<br>0.3708	-0.43338<br>0.6686	-0.35384<br>0.7265	-0.28039<br>0.7816
**5**	5.249661<br><.0001	4.750819<br><.0001	15.15525<br><.0001	0.130328<br>0.8974		0.140748<br>0.8892	-0.23254<br>0.8181	0.521207<br>0.6070	1.042462<br>0.3076	-0.30305<br>0.7645	-0.22351<br>0.8250	-0.15006<br>0.8820
**6**	5.108914<br><.0001	4.610071<br>0.0001	15.0145<br><.0001	-0.01042<br>0.9918	-0.14075<br>0.8892		-0.37329<br>0.7122	0.380459<br>0.7070	0.901714<br>0.3762	-0.4438<br>0.6612	-0.36426<br>0.7189	-0.29081<br>0.7737
**7**	5.482202<br><.0001	4.983359<br><.0001	15.38779<br><.0001	0.362868<br>0.7199	0.23254<br>0.8181	0.373288<br>0.7122		0.753747<br>0.4583	1.275002<br>0.2145	-0.07051<br>0.9444	0.009029<br>0.9929	0.08248<br>0.9349
**8**	4.728455<br><.0001	4.229612<br>0.0003	14.63405<br><.0001	-0.39088<br>0.6993	-0.52121<br>0.6070	-0.38046<br>0.7070	-0.75375<br>0.4583		0.521255<br>0.6070	-0.82426<br>0.4179	-0.74472<br>0.4637	-0.67127<br>0.5085
**9**	4.2072<br>0.0003	3.708357<br>0.0011	14.11279<br><.0001	-0.91213<br>0.3708	-1.04246<br>0.3076	-0.90171<br>0.3762	-1.275<br>0.2145	-0.52126<br>0.6070		-1.34552<br>0.1910	-1.26597<br>0.2177	-1.19252<br>0.2447
**10**	5.552716<br><.0001	5.053873<br><.0001	15.45831<br><.0001	0.433382<br>0.6686	0.303054<br>0.7645	0.443802<br>0.6612	0.070514<br>0.9444	0.824261<br>0.4179	1.345516<br>0.1910		0.079543<br>0.9373	0.152994<br>0.8797
**11**	5.473173<br><.0001	4.97433<br><.0001	15.37876<br><.0001	0.353839<br>0.7265	0.223511<br>0.8250	0.364259<br>0.7189	-0.00903<br>0.9929	0.744718<br>0.4637	1.265973<br>0.2177	-0.07954<br>0.9373		0.073451<br>0.9421
**12**	5.399722<br><.0001	4.900879<br><.0001	15.30531<br><.0001	0.280388<br>0.7816	0.15006<br>0.8820	0.290808<br>0.7737	-0.08248<br>0.9349	0.671267<br>0.5085	1.192522<br>0.2447	-0.15299<br>0.8797	-0.07345<br>0.9421	

The SAS System

The GLM Procedure
Least Squares Means

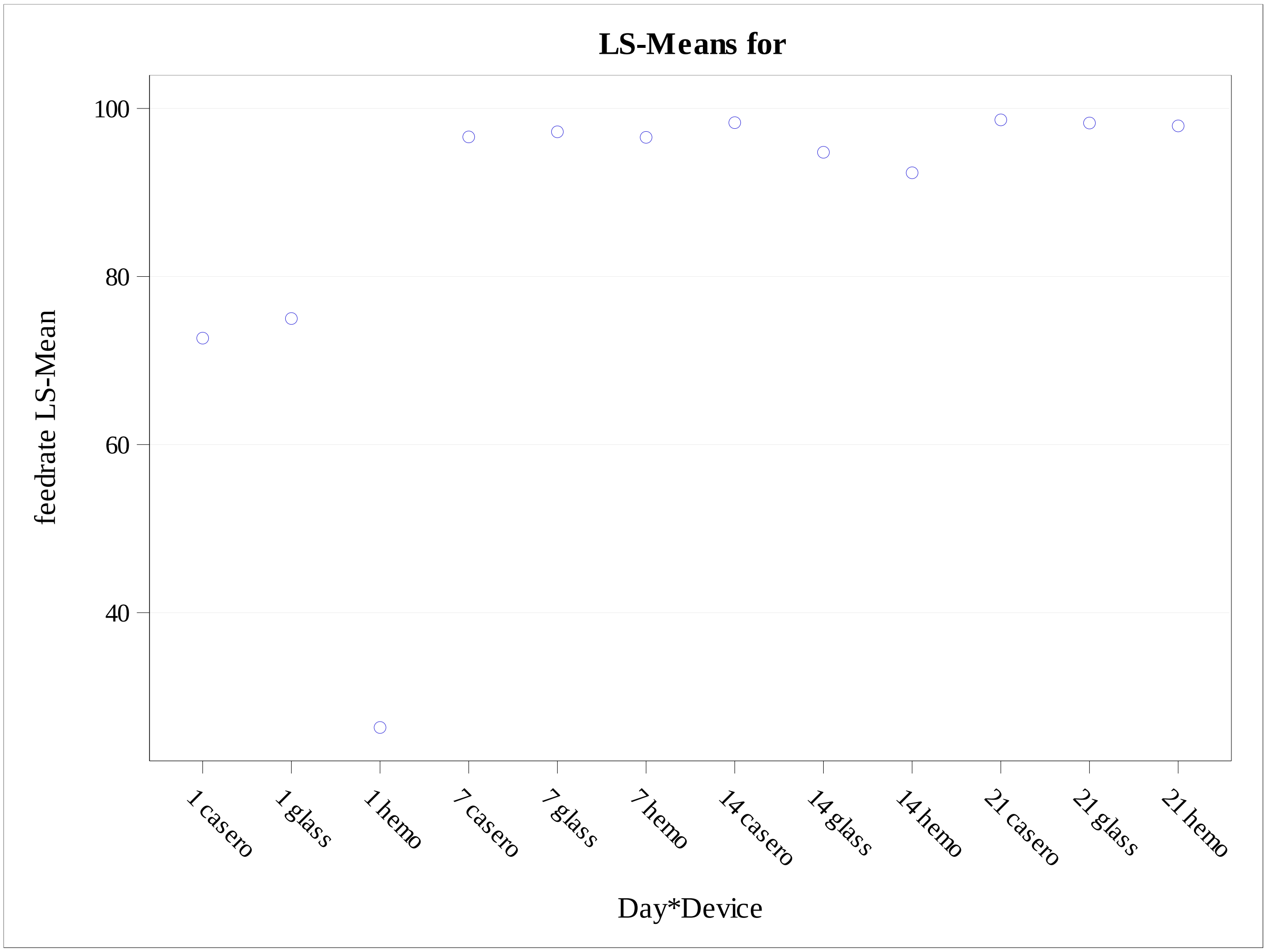

The SAS System

The GLM Procedure
Least Squares Means

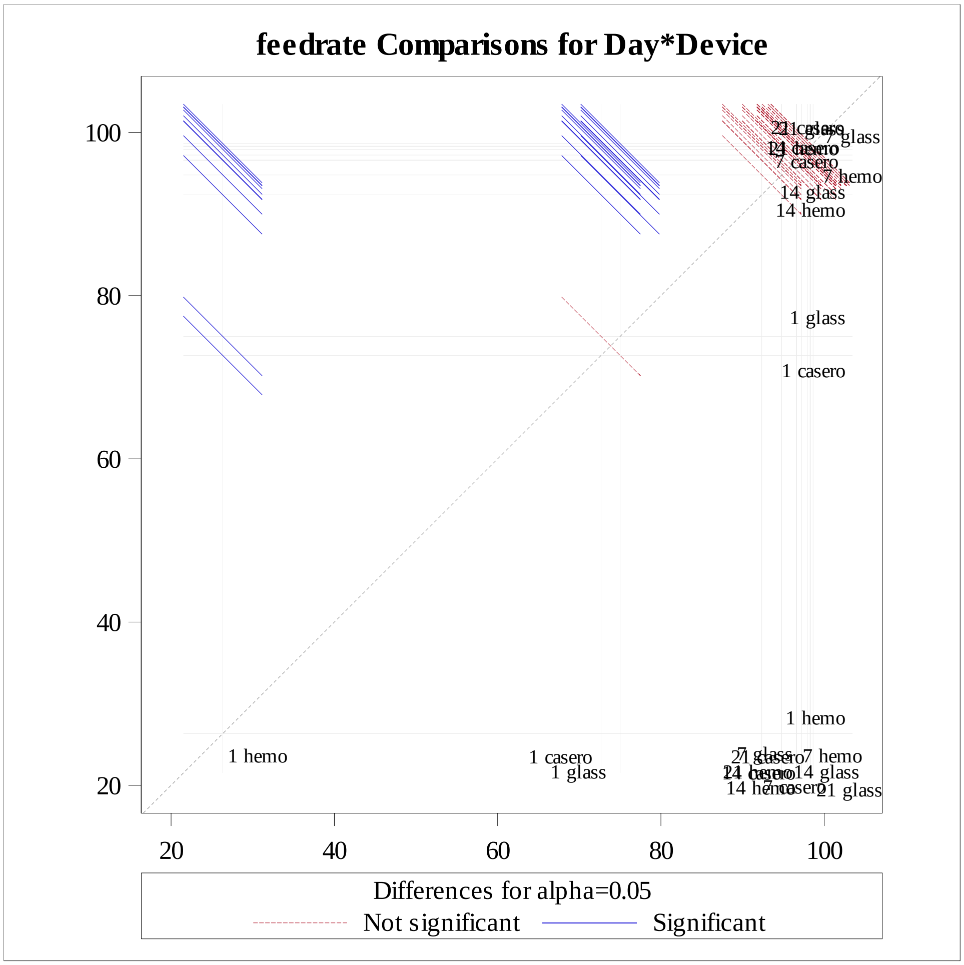

Note: To ensure overall protection level, only probabilities associated with pre-planned comparisons should be used.

The SAS System

The UNIVARIATE Procedure
Variable: RES

Moments			
**N**	36	**Sum Weights**	36
**Mean**	0	**Sum Observations**	0
**Std Deviation**	4.74384365	**Variance**	22.5040526
**Skewness**	-0.5566485	**Kurtosis**	4.3608506
**Uncorrected SS**	787.64184	**Corrected SS**	787.64184
**Coeff Variation**	.	**Std Error Mean**	0.79064061

Basic Statistical Measures			
**Location**		**Variability**	
**Mean**	0.00000	**Std Deviation**	4.74384
**Median**	0.00716	**Variance**	22.50405
**Mode**	-0.00716	**Range**	28.66667
		**Interquartile Range**	2.89933

Note: The mode displayed is the smallest of 3 modes with a count of 2.

Tests for Location: Mu0=0				
**Test**	**Statistic**		**p Value**	
**Student's t**	**t**	0	**Pr > |t|**	1.0000
**Sign**	**M**	1	**Pr >= |M|**	0.8679
**Signed Rank**	**S**	10.5	**Pr >= |S|**	0.8717

Tests for Normality				
**Test**	**Statistic**		**p Value**	
**Shapiro-Wilk**	**W**	0.856954	**Pr < W**	0.0003
**Kolmogorov-Smirnov**	**D**	0.191935	**Pr > D**	<0.0100
**Cramer-von Mises**	**W-Sq**	0.375892	**Pr > W-Sq**	<0.0050
**Anderson-Darling**	**A-Sq**	2.063103	**Pr > A-Sq**	<0.0050

The SAS System

The UNIVARIATE Procedure
Variable: RES

Quantiles (Definition 5)	
**Level**	**Quantile**
**100% Max**	13.33333333
**99%**	13.33333333
**95%**	7.66666667
**90%**	5.00000000
**75% Q3**	1.37102741
**50% Median**	0.00715922
**25% Q1**	-1.52830530
**10%**	-3.75517076
**5%**	-11.66666667
**1%**	-15.33333333
**0% Min**	-15.33333333

Extreme Observations			
**Lowest**		**Highest**	
**Value**	**Obs**	**Value**	**Obs**
-15.33333	4	2.77808	17
-11.66667	9	5.00000	8
-5.00000	5	7.66667	1
-3.75517	12	7.66667	7
-2.72109	30	13.33333	6

The SAS System

The UNIVARIATE Procedure

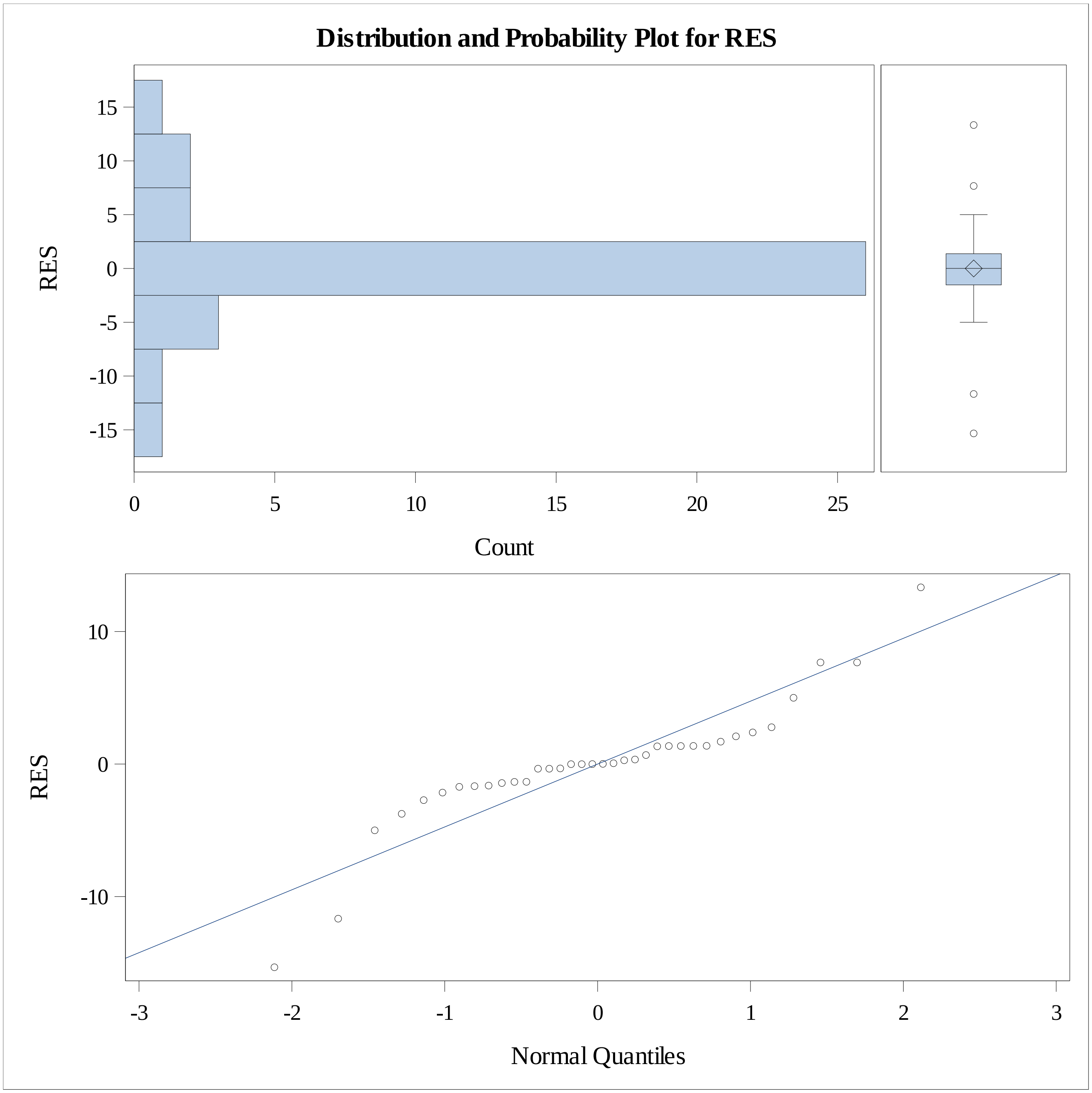

Survival

```
/*Survival rates Experiment #2*/
/*Import S1_File_July2023 Sheet Survival$*/

DATA EXP2_SURVIVE;
SET SURVIVE;
IF EXP = 2;
RUN;

ODS RTF FILE='SurviveExp2.RTF';
PROC GLM DATA=EXP2_SURVIVE;
CLASS  day Device;
MODEL SR = day Device day*device /SS3;
OUTPUT OUT=R RESIDUAL = RES;
LSMEANS day / STDERR PDIFF TDIFF;
LSMEANS device / STDERR PDIFF TDIFF;
*LSMEANS repeat / STDERR PDIFF TDIFF;
*LSMEANS repeat*DEVICE / STDERR PDIFF TDIFF;
LSMEANS Day*Device / STDERR PDIFF TDIFF;
PROC UNIVARIATE NORMAL PLOT DATA=R; VAR RES; RUN;

proc means data=EXP2_SURVIVE;
var SR;
class device;
run;
ODS RTF CLOSE;
```

The SAS System

The GLM Procedure

Class Level Information		
**Class**	**Levels**	**Values**
**Day**	29	0 1 2 3 4 5 6 7 8 9 10 11 12 13 14 15 16 17 18 19 20 21 22 23 24 25 26 27 28
**Device**	3	casero glass hemo

**Number of Observations Read**	261
**Number of Observations Used**	261

The SAS System

The GLM Procedure

Dependent Variable: SR
SR

Source	DF	Sum of Squares	Mean Square	F Value	Pr > F
**Model**	86	28.90086873	0.33605661	2.12	<.0001
**Error**	174	27.60600147	0.15865518		
**Corrected Total**	260	56.50687020			

R-Square	Coeff Var	Root MSE	SR Mean
0.511458	0.398989	0.398315	99.83111

Source	DF	Type III SS	Mean Square	F Value	Pr > F
**Day**	28	19.42160602	0.69362879	4.37	<.0001
**Device**	2	1.43327743	0.71663872	4.52	0.0122
**Day*Device**	56	8.04598527	0.14367831	0.91	0.6606

The SAS System

The GLM Procedure

Dependent Variable: SR
SR

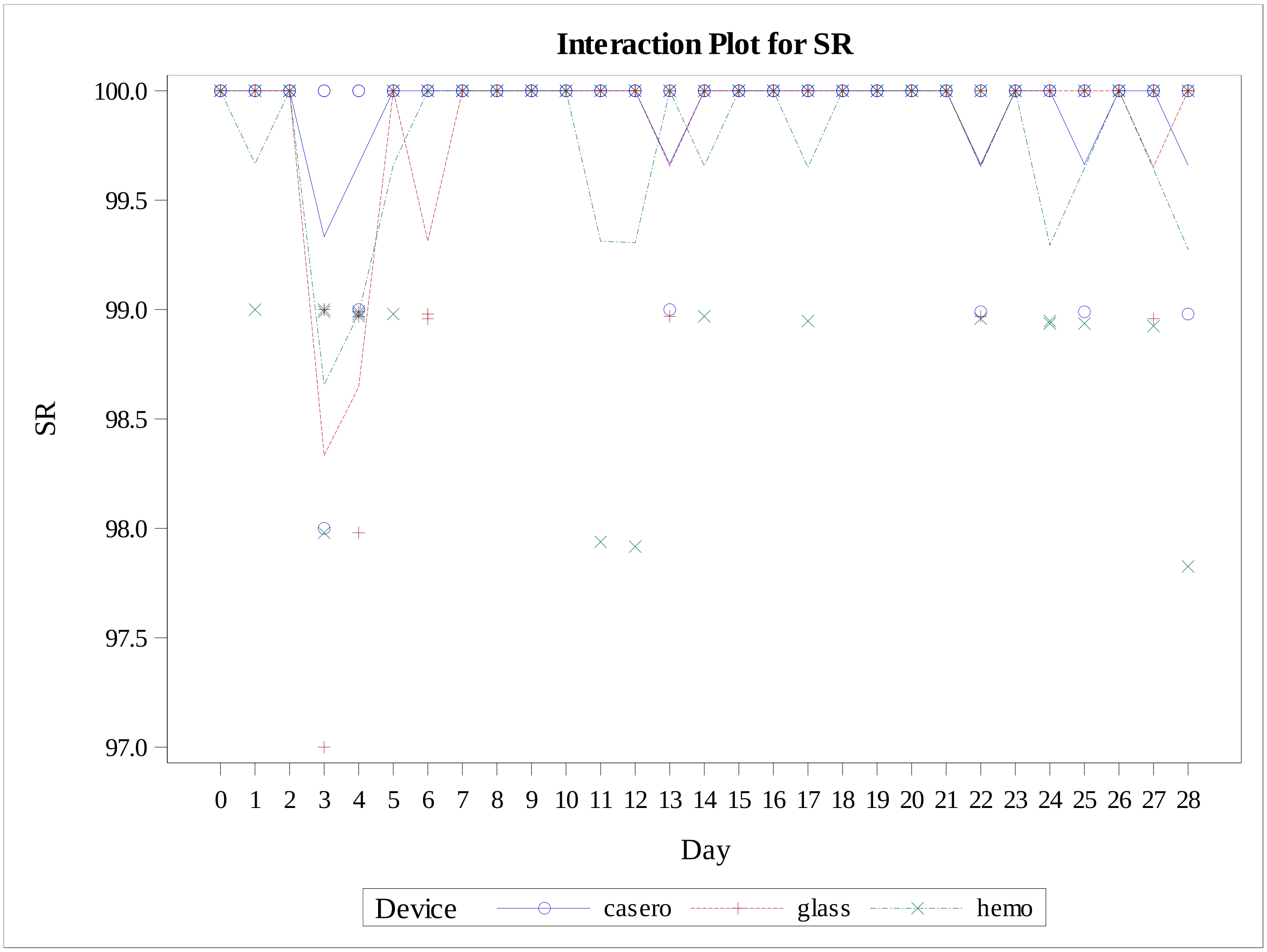

The SAS System

The GLM Procedure
Least Squares Means

Day	SR LSMEAN	Standard Error	Pr > |t|	LSMEAN Number
0	100.000000	0.132772	<.0001	1
1	99.888889	0.132772	<.0001	2
2	100.000000	0.132772	<.0001	3
3	98.774411	0.132772	<.0001	4
4	99.097481	0.132772	<.0001	5
5	99.886621	0.132772	<.0001	6
6	99.770881	0.132772	<.0001	7
7	100.000000	0.132772	<.0001	8
8	100.000000	0.132772	<.0001	9
9	100.000000	0.132772	<.0001	10
10	100.000000	0.132772	<.0001	11
11	99.770905	0.132772	<.0001	12
12	99.768519	0.132772	<.0001	13
13	99.774341	0.132772	<.0001	14
14	99.885452	0.132772	<.0001	15
15	100.000000	0.132772	<.0001	16
16	100.000000	0.132772	<.0001	17
17	99.883041	0.132772	<.0001	18
18	100.000000	0.132772	<.0001	19
19	100.000000	0.132772	<.0001	20
20	100.000000	0.132772	<.0001	21
21	100.000000	0.132772	<.0001	22
22	99.657478	0.132772	<.0001	23
23	100.000000	0.132772	<.0001	24
24	99.764838	0.132772	<.0001	25
25	99.769563	0.132772	<.0001	26
26	100.000000	0.132772	<.0001	27
27	99.764785	0.132772	<.0001	28
28	99.645075	0.132772	<.0001	29

The SAS System

The GLM Procedure
Least Squares Means

Least Squares Means for Effect Day<br>t for H0: LSMean(i)=LSMean(j) / Pr > |t| <br>Dependent Variable: SR											
**i/j**	**1**	**2**	**3**	**4**	**5**	**6**	**7**	**8**	**9**	**10**	**11**
**1**		0.591748<br>0.5548	0<br>1.0000	6.527157<br><.0001	4.80657<br><.0001	0.603824<br>0.5467	1.220228<br>0.2240	0<br>1.0000	0<br>1.0000	0<br>1.0000	0<br>1.0000
**2**	-0.59175<br>0.5548		-0.59175<br>0.5548	5.935409<br><.0001	4.214823<br><.0001	0.012076<br>0.9904	0.62848<br>0.5305	-0.59175<br>0.5548	-0.59175<br>0.5548	-0.59175<br>0.5548	-0.59175<br>0.5548
**3**	0<br>1.0000	0.591748<br>0.5548		6.527157<br><.0001	4.80657<br><.0001	0.603824<br>0.5467	1.220228<br>0.2240	0<br>1.0000	0<br>1.0000	0<br>1.0000	0<br>1.0000
**4**	-6.52716<br><.0001	-5.93541<br><.0001	-6.52716<br><.0001		-1.72059<br>0.0871	-5.92333<br><.0001	-5.30693<br><.0001	-6.52716<br><.0001	-6.52716<br><.0001	-6.52716<br><.0001	-6.52716<br><.0001
**5**	-4.80657<br><.0001	-4.21482<br><.0001	-4.80657<br><.0001	1.720587<br>0.0871		-4.20275<br><.0001	-3.58634<br>0.0004	-4.80657<br><.0001	-4.80657<br><.0001	-4.80657<br><.0001	-4.80657<br><.0001
**6**	-0.60382<br>0.5467	-0.01208<br>0.9904	-0.60382<br>0.5467	5.923333<br><.0001	4.202746<br><.0001		0.616404<br>0.5384	-0.60382<br>0.5467	-0.60382<br>0.5467	-0.60382<br>0.5467	-0.60382<br>0.5467
**7**	-1.22023<br>0.2240	-0.62848<br>0.5305	-1.22023<br>0.2240	5.306929<br><.0001	3.586342<br>0.0004	-0.6164<br>0.5384		-1.22023<br>0.2240	-1.22023<br>0.2240	-1.22023<br>0.2240	-1.22023<br>0.2240
**8**	0<br>1.0000	0.591748<br>0.5548	0<br>1.0000	6.527157<br><.0001	4.80657<br><.0001	0.603824<br>0.5467	1.220228<br>0.2240		0<br>1.0000	0<br>1.0000	0<br>1.0000
**9**	0<br>1.0000	0.591748<br>0.5548	0<br>1.0000	6.527157<br><.0001	4.80657<br><.0001	0.603824<br>0.5467	1.220228<br>0.2240	0<br>1.0000		0<br>1.0000	0<br>1.0000
**10**	0<br>1.0000	0.591748<br>0.5548	0<br>1.0000	6.527157<br><.0001	4.80657<br><.0001	0.603824<br>0.5467	1.220228<br>0.2240	0<br>1.0000	0<br>1.0000		0<br>1.0000
**11**	0<br>1.0000	0.591748<br>0.5548	0<br>1.0000	6.527157<br><.0001	4.80657<br><.0001	0.603824<br>0.5467	1.220228<br>0.2240	0<br>1.0000	0<br>1.0000	0<br>1.0000	
**12**	-1.2201<br>0.2241	-0.62835<br>0.5306	-1.2201<br>0.2241	5.307059<br><.0001	3.586472<br>0.0004	-0.61627<br>0.5385	0.00013<br>0.9999	-1.2201<br>0.2241	-1.2201<br>0.2241	-1.2201<br>0.2241	-1.2201<br>0.2241
**13**	-1.23281<br>0.2193	-0.64106<br>0.5223	-1.23281<br>0.2193	5.294349<br><.0001	3.573763<br>0.0005	-0.62898<br>0.5302	-0.01258<br>0.9900	-1.23281<br>0.2193	-1.23281<br>0.2193	-1.23281<br>0.2193	-1.23281<br>0.2193
**14**	-1.2018<br>0.2311	-0.61005<br>0.5426	-1.2018<br>0.2311	5.32536<br><.0001	3.604773<br>0.0004	-0.59797<br>0.5506	0.018431<br>0.9853	-1.2018<br>0.2311	-1.2018<br>0.2311	-1.2018<br>0.2311	-1.2018<br>0.2311
**15**	-0.61005<br>0.5426	-0.0183<br>0.9854	-0.61005<br>0.5426	5.917108<br><.0001	4.196521<br><.0001	-0.00622<br>0.9950	0.610179<br>0.5425	-0.61005<br>0.5426	-0.61005<br>0.5426	-0.61005<br>0.5426	-0.61005<br>0.5426
**16**	0<br>1.0000	0.591748<br>0.5548	0<br>1.0000	6.527157<br><.0001	4.80657<br><.0001	0.603824<br>0.5467	1.220228<br>0.2240	0<br>1.0000	0<br>1.0000	0<br>1.0000	0<br>1.0000
**17**	0<br>1.0000	0.591748<br>0.5548	0<br>1.0000	6.527157<br><.0001	4.80657<br><.0001	0.603824<br>0.5467	1.220228<br>0.2240	0<br>1.0000	0<br>1.0000	0<br>1.0000	0<br>1.0000
**18**	-0.62289<br>0.5342	-0.03114<br>0.9752	-0.62289<br>0.5342	5.904265<br><.0001	4.183678<br><.0001	-0.01907<br>0.9848	0.597336<br>0.5511	-0.62289<br>0.5342	-0.62289<br>0.5342	-0.62289<br>0.5342	-0.62289<br>0.5342
**19**	0<br>1.0000	0.591748<br>0.5548	0<br>1.0000	6.527157<br><.0001	4.80657<br><.0001	0.603824<br>0.5467	1.220228<br>0.2240	0<br>1.0000	0<br>1.0000	0<br>1.0000	0<br>1.0000

The SAS System

The GLM Procedure
Least Squares Means

Least Squares Means for Effect Day
t for H0: LSMean(i)=LSMean(j) / Pr > |t|

Dependent Variable: SR

i/j	1	2	3	4	5	6	7	8	9	10	11
20	0<br>1.0000	0.591748<br>0.5548	0<br>1.0000	6.527157<br><.0001	4.80657<br><.0001	0.603824<br>0.5467	1.220228<br>0.2240	0<br>1.0000	0<br>1.0000	0<br>1.0000	0<br>1.0000
21	0<br>1.0000	0.591748<br>0.5548	0<br>1.0000	6.527157<br><.0001	4.80657<br><.0001	0.603824<br>0.5467	1.220228<br>0.2240	0<br>1.0000	0<br>1.0000	0<br>1.0000	0<br>1.0000
22	0<br>1.0000	0.591748<br>0.5548	0<br>1.0000	6.527157<br><.0001	4.80657<br><.0001	0.603824<br>0.5467	1.220228<br>0.2240	0<br>1.0000	0<br>1.0000	0<br>1.0000	0<br>1.0000
23	-1.82418<br>0.0698	-1.23243<br>0.2195	-1.82418<br>0.0698	4.702979<br><.0001	2.982392<br>0.0033	-1.22035<br>0.2240	-0.60395<br>0.5467	-1.82418<br>0.0698	-1.82418<br>0.0698	-1.82418<br>0.0698	-1.82418<br>0.0698
24	0<br>1.0000	0.591748<br>0.5548	0<br>1.0000	6.527157<br><.0001	4.80657<br><.0001	0.603824<br>0.5467	1.220228<br>0.2240	0<br>1.0000	0<br>1.0000	0<br>1.0000	0<br>1.0000
25	-1.25241<br>0.2121	-0.66066<br>0.5097	-1.25241<br>0.2121	5.274746<br><.0001	3.554159<br>0.0005	-0.64859<br>0.5175	-0.03218<br>0.9744	-1.25241<br>0.2121	-1.25241<br>0.2121	-1.25241<br>0.2121	-1.25241<br>0.2121
26	-1.22724<br>0.2214	-0.6355<br>0.5259	-1.22724<br>0.2214	5.299913<br><.0001	3.579327<br>0.0004	-0.62342<br>0.5338	-0.00702<br>0.9944	-1.22724<br>0.2214	-1.22724<br>0.2214	-1.22724<br>0.2214	-1.22724<br>0.2214
27	0<br>1.0000	0.591748<br>0.5548	0<br>1.0000	6.527157<br><.0001	4.80657<br><.0001	0.603824<br>0.5467	1.220228<br>0.2240	0<br>1.0000	0<br>1.0000	0<br>1.0000	0<br>1.0000
28	-1.25269<br>0.2120	-0.66094<br>0.5095	-1.25269<br>0.2120	5.274465<br><.0001	3.553879<br>0.0005	-0.64887<br>0.5173	-0.03246<br>0.9741	-1.25269<br>0.2120	-1.25269<br>0.2120	-1.25269<br>0.2120	-1.25269<br>0.2120
29	-1.89023<br>0.0604	-1.29848<br>0.1958	-1.89023<br>0.0604	4.636925<br><.0001	2.916338<br>0.0040	-1.28641<br>0.2000	-0.67<br>0.5037	-1.89023<br>0.0604	-1.89023<br>0.0604	-1.89023<br>0.0604	-1.89023<br>0.0604

Least Squares Means for Effect Day
t for H0: LSMean(i)=LSMean(j) / Pr > |t|

Dependent Variable: SR

i/j	12	13	14	15	16	17	18	19	20	21	22
1	1.220098<br>0.2241	1.232808<br>0.2193	1.201797<br>0.2311	0.610049<br>0.5426	0<br>1.0000	0<br>1.0000	0.622892<br>0.5342	0<br>1.0000	0<br>1.0000	0<br>1.0000	0<br>1.0000
2	0.628351<br>0.5306	0.64106<br>0.5223	0.610049<br>0.5426	0.018301<br>0.9854	-0.59175<br>0.5548	-0.59175<br>0.5548	0.031145<br>0.9752	-0.59175<br>0.5548	-0.59175<br>0.5548	-0.59175<br>0.5548	-0.59175<br>0.5548
3	1.220098<br>0.2241	1.232808<br>0.2193	1.201797<br>0.2311	0.610049<br>0.5426	0<br>1.0000	0<br>1.0000	0.622892<br>0.5342	0<br>1.0000	0<br>1.0000	0<br>1.0000	0<br>1.0000
4	-5.30706<br><.0001	-5.29435<br><.0001	-5.32536<br><.0001	-5.91711<br><.0001	-6.52716<br><.0001	-6.52716<br><.0001	-5.90426<br><.0001	-6.52716<br><.0001	-6.52716<br><.0001	-6.52716<br><.0001	-6.52716<br><.0001
5	-3.58647<br>0.0004	-3.57376<br>0.0005	-3.60477<br>0.0004	-4.19652<br><.0001	-4.80657<br><.0001	-4.80657<br><.0001	-4.18368<br><.0001	-4.80657<br><.0001	-4.80657<br><.0001	-4.80657<br><.0001	-4.80657<br><.0001
6	0.616274<br>0.5385	0.628984<br>0.5302	0.597973<br>0.5506	0.006225<br>0.9950	-0.60382<br>0.5467	-0.60382<br>0.5467	0.019068<br>0.9848	-0.60382<br>0.5467	-0.60382<br>0.5467	-0.60382<br>0.5467	-0.60382<br>0.5467

The SAS System

The GLM Procedure
Least Squares Means

Least Squares Means for Effect Day
t for H0: LSMean(i)=LSMean(j) / Pr > |t|

Dependent Variable: SR

i/j	12	13	14	15	16	17	18	19	20	21	22
**7**	-0.00013<br>0.9999	0.01258<br>0.9900	-0.01843<br>0.9853	-0.61018<br>0.5425	-1.22023<br>0.2240	-1.22023<br>0.2240	-0.59734<br>0.5511	-1.22023<br>0.2240	-1.22023<br>0.2240	-1.22023<br>0.2240	-1.22023<br>0.2240
**8**	1.220098<br>0.2241	1.232808<br>0.2193	1.201797<br>0.2311	0.610049<br>0.5426	0<br>1.0000	0<br>1.0000	0.622892<br>0.5342	0<br>1.0000	0<br>1.0000	0<br>1.0000	0<br>1.0000
**9**	1.220098<br>0.2241	1.232808<br>0.2193	1.201797<br>0.2311	0.610049<br>0.5426	0<br>1.0000	0<br>1.0000	0.622892<br>0.5342	0<br>1.0000	0<br>1.0000	0<br>1.0000	0<br>1.0000
**10**	1.220098<br>0.2241	1.232808<br>0.2193	1.201797<br>0.2311	0.610049<br>0.5426	0<br>1.0000	0<br>1.0000	0.622892<br>0.5342	0<br>1.0000	0<br>1.0000	0<br>1.0000	0<br>1.0000
**11**	1.220098<br>0.2241	1.232808<br>0.2193	1.201797<br>0.2311	0.610049<br>0.5426	0<br>1.0000	0<br>1.0000	0.622892<br>0.5342	0<br>1.0000	0<br>1.0000	0<br>1.0000	0<br>1.0000
**12**		0.012709<br>0.9899	-0.0183<br>0.9854	-0.61005<br>0.5426	-1.2201<br>0.2241	-1.2201<br>0.2241	-0.59721<br>0.5511	-1.2201<br>0.2241	-1.2201<br>0.2241	-1.2201<br>0.2241	-1.2201<br>0.2241
**13**	-0.01271<br>0.9899		-0.03101<br>0.9753	-0.62276<br>0.5343	-1.23281<br>0.2193	-1.23281<br>0.2193	-0.60992<br>0.5427	-1.23281<br>0.2193	-1.23281<br>0.2193	-1.23281<br>0.2193	-1.23281<br>0.2193
**14**	0.018301<br>0.9854	0.031011<br>0.9753		-0.59175<br>0.5548	-1.2018<br>0.2311	-1.2018<br>0.2311	-0.5789<br>0.5634	-1.2018<br>0.2311	-1.2018<br>0.2311	-1.2018<br>0.2311	-1.2018<br>0.2311
**15**	0.610049<br>0.5426	0.622759<br>0.5343	0.591748<br>0.5548		-0.61005<br>0.5426	-0.61005<br>0.5426	0.012843<br>0.9898	-0.61005<br>0.5426	-0.61005<br>0.5426	-0.61005<br>0.5426	-0.61005<br>0.5426
**16**	1.220098<br>0.2241	1.232808<br>0.2193	1.201797<br>0.2311	0.610049<br>0.5426		0<br>1.0000	0.622892<br>0.5342	0<br>1.0000	0<br>1.0000	0<br>1.0000	0<br>1.0000
**17**	1.220098<br>0.2241	1.232808<br>0.2193	1.201797<br>0.2311	0.610049<br>0.5426	0<br>1.0000		0.622892<br>0.5342	0<br>1.0000	0<br>1.0000	0<br>1.0000	0<br>1.0000
**18**	0.597206<br>0.5511	0.609915<br>0.5427	0.578905<br>0.5634	-0.01284<br>0.9898	-0.62289<br>0.5342	-0.62289<br>0.5342		-0.62289<br>0.5342	-0.62289<br>0.5342	-0.62289<br>0.5342	-0.62289<br>0.5342
**19**	1.220098<br>0.2241	1.232808<br>0.2193	1.201797<br>0.2311	0.610049<br>0.5426	0<br>1.0000	0<br>1.0000	0.622892<br>0.5342		0<br>1.0000	0<br>1.0000	0<br>1.0000
**20**	1.220098<br>0.2241	1.232808<br>0.2193	1.201797<br>0.2311	0.610049<br>0.5426	0<br>1.0000	0<br>1.0000	0.622892<br>0.5342	0<br>1.0000		0<br>1.0000	0<br>1.0000
**21**	1.220098<br>0.2241	1.232808<br>0.2193	1.201797<br>0.2311	0.610049<br>0.5426	0<br>1.0000	0<br>1.0000	0.622892<br>0.5342	0<br>1.0000	0<br>1.0000		0<br>1.0000
**22**	1.220098<br>0.2241	1.232808<br>0.2193	1.201797<br>0.2311	0.610049<br>0.5426	0<br>1.0000	0<br>1.0000	0.622892<br>0.5342	0<br>1.0000	0<br>1.0000	0<br>1.0000	
**23**	-0.60408<br>0.5466	-0.59137<br>0.5550	-0.62238<br>0.5345	-1.21413<br>0.2263	-1.82418<br>0.0698	-1.82418<br>0.0698	-1.20129<br>0.2313	-1.82418<br>0.0698	-1.82418<br>0.0698	-1.82418<br>0.0698	-1.82418<br>0.0698
**24**	1.220098<br>0.2241	1.232808<br>0.2193	1.201797<br>0.2311	0.610049<br>0.5426	0<br>1.0000	0<br>1.0000	0.622892<br>0.5342	0<br>1.0000	0<br>1.0000	0<br>1.0000	0<br>1.0000
**25**	-0.03231<br>0.9743	-0.0196<br>0.9844	-0.05061<br>0.9597	-0.64236<br>0.5215	-1.25241<br>0.2121	-1.25241<br>0.2121	-0.62952<br>0.5298	-1.25241<br>0.2121	-1.25241<br>0.2121	-1.25241<br>0.2121	-1.25241<br>0.2121

The SAS System

The GLM Procedure
Least Squares Means

Least Squares Means for Effect Day
t for H0: LSMean(i)=LSMean(j) / Pr > |t|

Dependent Variable: SR

i/j	12	13	14	15	16	17	18	19	20	21	22
**26**	-0.00715<br>0.9943	0.005564<br>0.9956	-0.02545<br>0.9797	-0.61719<br>0.5379	-1.22724<br>0.2214	-1.22724<br>0.2214	-0.60435<br>0.5464	-1.22724<br>0.2214	-1.22724<br>0.2214	-1.22724<br>0.2214	-1.22724<br>0.2214
**27**	1.220098<br>0.2241	1.232808<br>0.2193	1.201797<br>0.2311	0.610049<br>0.5426	0<br>1.0000	0<br>1.0000	0.622892<br>0.5342	0<br>1.0000	0<br>1.0000	0<br>1.0000	0<br>1.0000
**28**	-0.03259<br>0.9740	-0.01988<br>0.9842	-0.05089<br>0.9595	-0.64264<br>0.5213	-1.25269<br>0.2120	-1.25269<br>0.2120	-0.6298<br>0.5297	-1.25269<br>0.2120	-1.25269<br>0.2120	-1.25269<br>0.2120	-1.25269<br>0.2120
**29**	-0.67013<br>0.5037	-0.65742<br>0.5118	-0.68844<br>0.4921	-1.28018<br>0.2022	-1.89023<br>0.0604	-1.89023<br>0.0604	-1.26734<br>0.2067	-1.89023<br>0.0604	-1.89023<br>0.0604	-1.89023<br>0.0604	-1.89023<br>0.0604

Least Squares Means for Effect Day
t for H0: LSMean(i)=LSMean(j) / Pr > |t|

Dependent Variable: SR

i/j	23	24	25	26	27	28	29
**1**	1.824178<br>0.0698	0<br>1.0000	1.252411<br>0.2121	1.227244<br>0.2214	0<br>1.0000	1.252692<br>0.2120	1.890232<br>0.0604
**2**	1.23243<br>0.2195	-0.59175<br>0.5548	0.660664<br>0.5097	0.635496<br>0.5259	-0.59175<br>0.5548	0.660944<br>0.5095	1.298485<br>0.1958
**3**	1.824178<br>0.0698	0<br>1.0000	1.252411<br>0.2121	1.227244<br>0.2214	0<br>1.0000	1.252692<br>0.2120	1.890232<br>0.0604
**4**	-4.70298<br><.0001	-6.52716<br><.0001	-5.27475<br><.0001	-5.29991<br><.0001	-6.52716<br><.0001	-5.27447<br><.0001	-4.63692<br><.0001
**5**	-2.98239<br>0.0033	-4.80657<br><.0001	-3.55416<br>0.0005	-3.57933<br>0.0004	-4.80657<br><.0001	-3.55388<br>0.0005	-2.91634<br>0.0040
**6**	1.220354<br>0.2240	-0.60382<br>0.5467	0.648587<br>0.5175	0.62342<br>0.5338	-0.60382<br>0.5467	0.648868<br>0.5173	1.286408<br>0.2000
**7**	0.60395<br>0.5467	-1.22023<br>0.2240	0.032183<br>0.9744	0.007016<br>0.9944	-1.22023<br>0.2240	0.032464<br>0.9741	0.670004<br>0.5037
**8**	1.824178<br>0.0698	0<br>1.0000	1.252411<br>0.2121	1.227244<br>0.2214	0<br>1.0000	1.252692<br>0.2120	1.890232<br>0.0604
**9**	1.824178<br>0.0698	0<br>1.0000	1.252411<br>0.2121	1.227244<br>0.2214	0<br>1.0000	1.252692<br>0.2120	1.890232<br>0.0604
**10**	1.824178<br>0.0698	0<br>1.0000	1.252411<br>0.2121	1.227244<br>0.2214	0<br>1.0000	1.252692<br>0.2120	1.890232<br>0.0604
**11**	1.824178<br>0.0698	0<br>1.0000	1.252411<br>0.2121	1.227244<br>0.2214	0<br>1.0000	1.252692<br>0.2120	1.890232<br>0.0604
**12**	0.60408<br>0.5466	-1.2201<br>0.2241	0.032313<br>0.9743	0.007145<br>0.9943	-1.2201<br>0.2241	0.032593<br>0.9740	0.670134<br>0.5037

The SAS System

The GLM Procedure
Least Squares Means

Least Squares Means for Effect Day<br>t for H0: LSMean(i)=LSMean(j) / Pr > |t| <br>Dependent Variable: SR							
**i/j**	**23**	**24**	**25**	**26**	**27**	**28**	**29**
**13**	0.59137<br>0.5550	-1.23281<br>0.2193	0.019603<br>0.9844	-0.00556<br>0.9956	-1.23281<br>0.2193	0.019884<br>0.9842	0.657425<br>0.5118
**14**	0.622381<br>0.5345	-1.2018<br>0.2311	0.050614<br>0.9597	0.025447<br>0.9797	-1.2018<br>0.2311	0.050895<br>0.9595	0.688435<br>0.4921
**15**	1.214129<br>0.2263	-0.61005<br>0.5426	0.642362<br>0.5215	0.617195<br>0.5379	-0.61005<br>0.5426	0.642643<br>0.5213	1.280183<br>0.2022
**16**	1.824178<br>0.0698	0<br>1.0000	1.252411<br>0.2121	1.227244<br>0.2214	0<br>1.0000	1.252692<br>0.2120	1.890232<br>0.0604
**17**	1.824178<br>0.0698	0<br>1.0000	1.252411<br>0.2121	1.227244<br>0.2214	0<br>1.0000	1.252692<br>0.2120	1.890232<br>0.0604
**18**	1.201286<br>0.2313	-0.62289<br>0.5342	0.629519<br>0.5298	0.604352<br>0.5464	-0.62289<br>0.5342	0.629799<br>0.5297	1.26734<br>0.2067
**19**	1.824178<br>0.0698	0<br>1.0000	1.252411<br>0.2121	1.227244<br>0.2214	0<br>1.0000	1.252692<br>0.2120	1.890232<br>0.0604
**20**	1.824178<br>0.0698	0<br>1.0000	1.252411<br>0.2121	1.227244<br>0.2214	0<br>1.0000	1.252692<br>0.2120	1.890232<br>0.0604
**21**	1.824178<br>0.0698	0<br>1.0000	1.252411<br>0.2121	1.227244<br>0.2214	0<br>1.0000	1.252692<br>0.2120	1.890232<br>0.0604
**22**	1.824178<br>0.0698	0<br>1.0000	1.252411<br>0.2121	1.227244<br>0.2214	0<br>1.0000	1.252692<br>0.2120	1.890232<br>0.0604
**23**		-1.82418<br>0.0698	-0.57177<br>0.5682	-0.59693<br>0.5513	-1.82418<br>0.0698	-0.57149<br>0.5684	0.066054<br>0.9474
**24**	1.824178<br>0.0698		1.252411<br>0.2121	1.227244<br>0.2214	0<br>1.0000	1.252692<br>0.2120	1.890232<br>0.0604
**25**	0.571767<br>0.5682	-1.25241<br>0.2121		-0.02517<br>0.9800	-1.25241<br>0.2121	0.000281<br>0.9998	0.637821<br>0.5244
**26**	0.596934<br>0.5513	-1.22724<br>0.2214	0.025167<br>0.9800		-1.22724<br>0.2214	0.025448<br>0.9797	0.662989<br>0.5082
**27**	1.824178<br>0.0698	0<br>1.0000	1.252411<br>0.2121	1.227244<br>0.2214		1.252692<br>0.2120	1.890232<br>0.0604
**28**	0.571486<br>0.5684	-1.25269<br>0.2120	-0.00028<br>0.9998	-0.02545<br>0.9797	-1.25269<br>0.2120		0.637541<br>0.5246
**29**	-0.06605<br>0.9474	-1.89023<br>0.0604	-0.63782<br>0.5244	-0.66299<br>0.5082	-1.89023<br>0.0604	-0.63754<br>0.5246	

The SAS System

The GLM Procedure
Least Squares Means

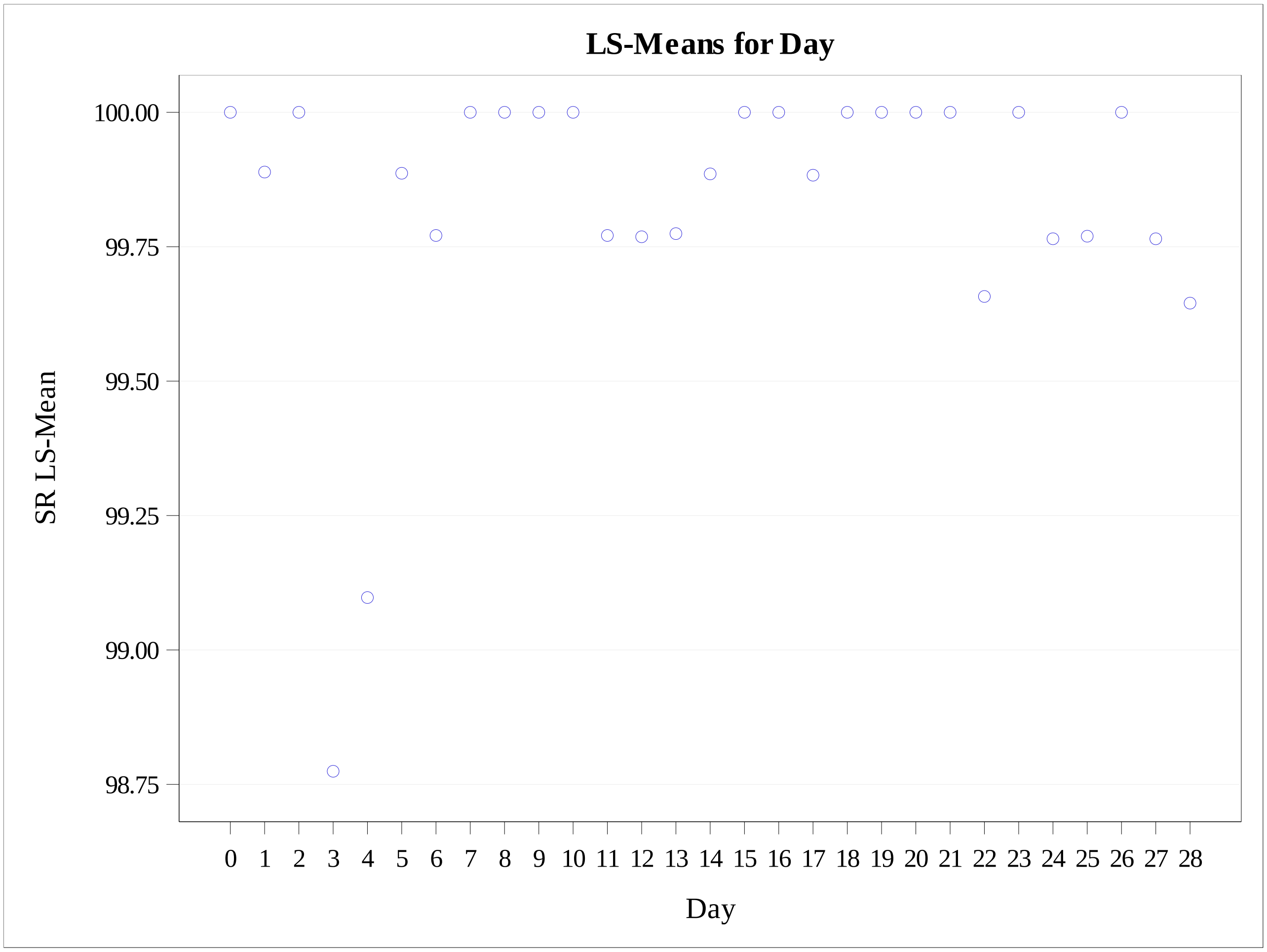

The SAS System

The GLM Procedure
Least Squares Means

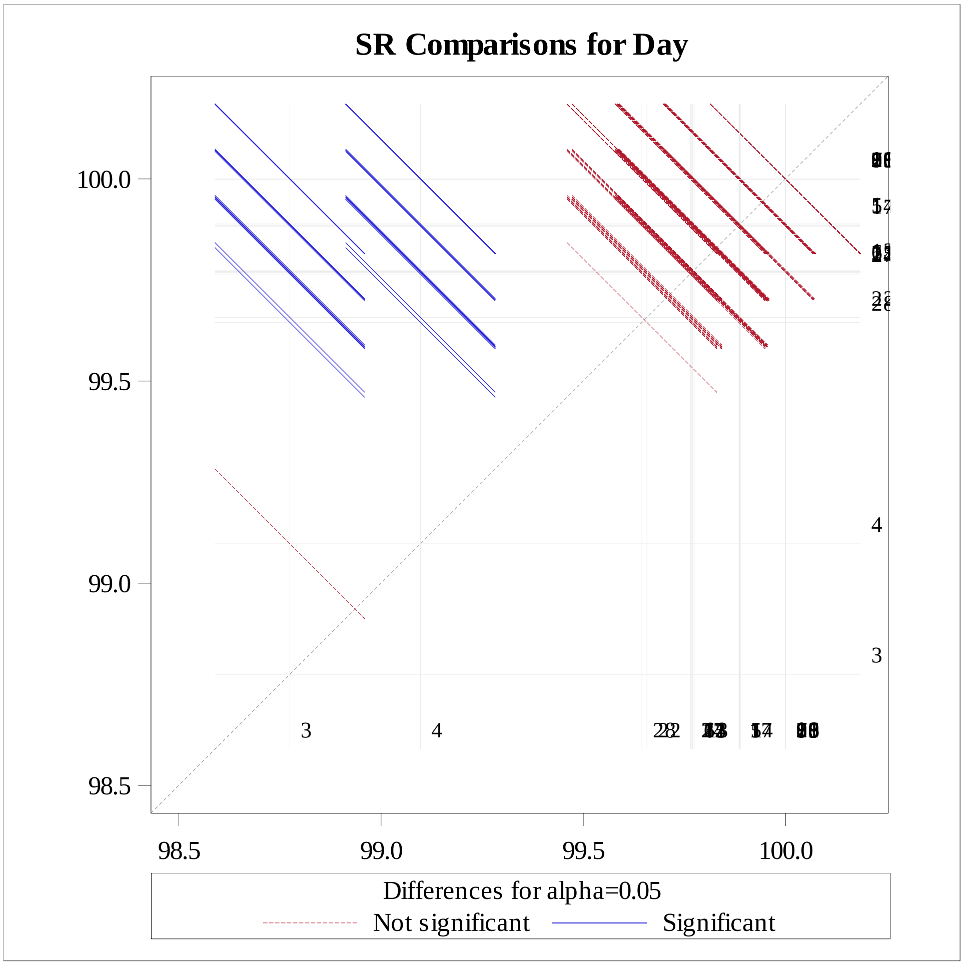

Note: To ensure overall protection level, only probabilities associated with pre-planned comparisons should be used.

The SAS System

The GLM Procedure
Least Squares Means

Device	SR LSMEAN	Standard Error	Pr > |t|	LSMEAN Number
**casero**	99.9190734	0.0427039	<.0001	1
**glass**	99.8364732	0.0427039	<.0001	2
**hemo**	99.7377927	0.0427039	<.0001	3

Least Squares Means for Effect Device
t for H0: LSMean(i)=LSMean(j) / Pr > |t|

Dependent Variable: SR

i/j	1	2	3
**1**		1.367724<br>0.1732	3.001713<br>0.0031
**2**	-1.36772<br>0.1732		1.633989<br>0.1041
**3**	-3.00171<br>0.0031	-1.63399<br>0.1041	

The SAS System

The GLM Procedure
Least Squares Means

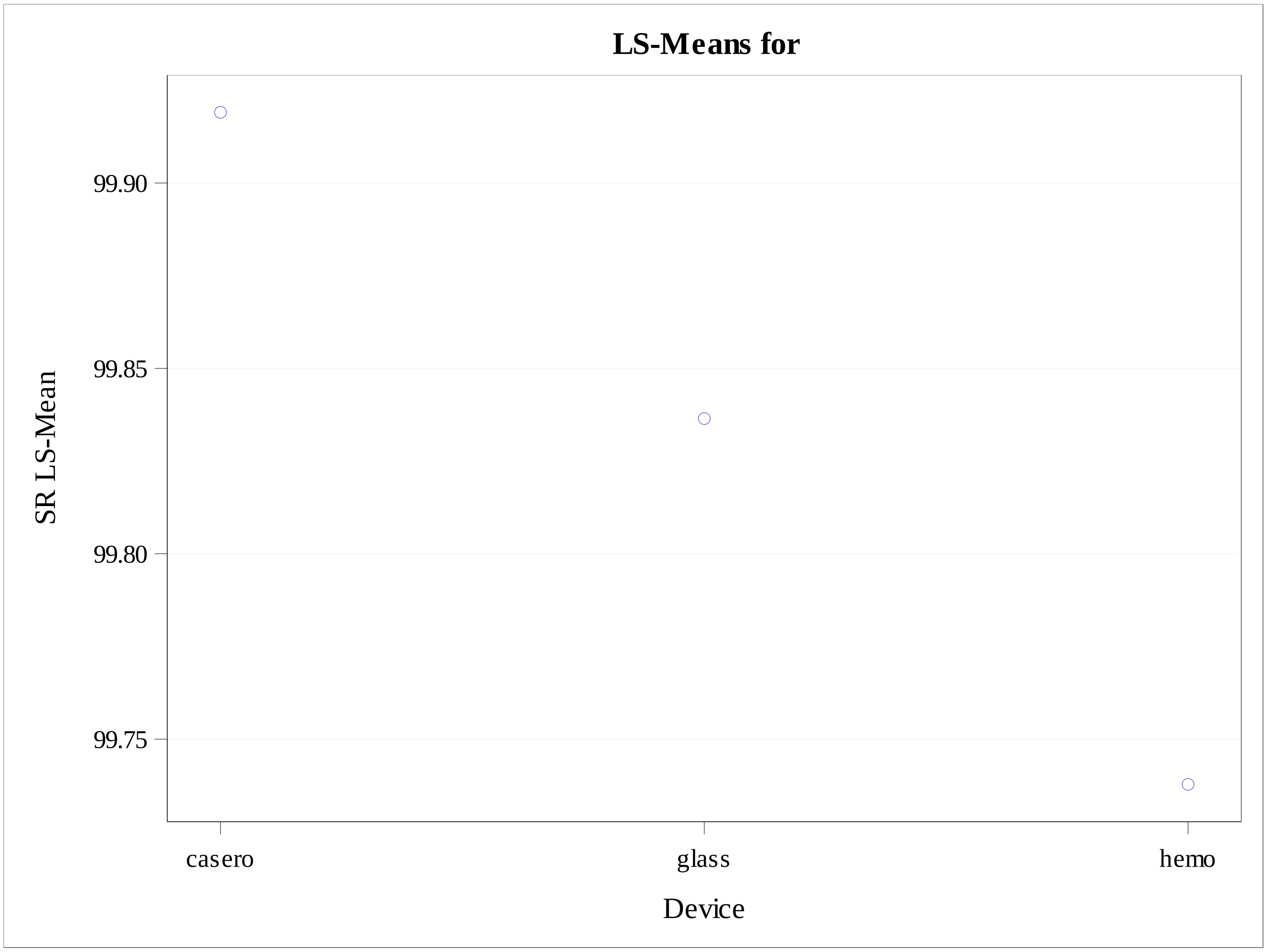

The SAS System

The GLM Procedure
Least Squares Means

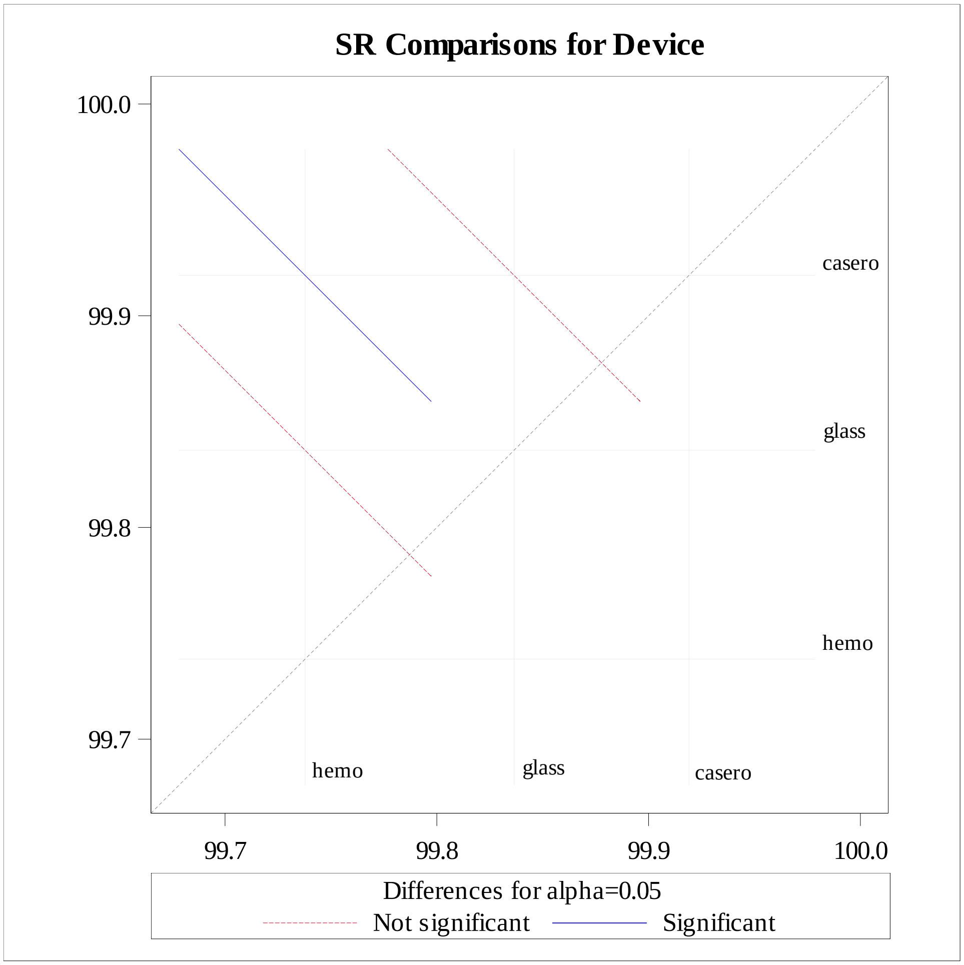

Note: To ensure overall protection level, only probabilities associated with pre-planned comparisons should be used.

The SAS System

The GLM Procedure
Least Squares Means

Day	Device	SR LSMEAN	Standard Error	Pr > |t|	LSMEAN Number
**0**	**casero**	100.000000	0.229968	<.0001	1
**0**	**glass**	100.000000	0.229968	<.0001	2
**0**	**hemo**	100.000000	0.229968	<.0001	3
**1**	**casero**	100.000000	0.229968	<.0001	4
**1**	**glass**	100.000000	0.229968	<.0001	5
**1**	**hemo**	99.666667	0.229968	<.0001	6
**2**	**casero**	100.000000	0.229968	<.0001	7
**2**	**glass**	100.000000	0.229968	<.0001	8
**2**	**hemo**	100.000000	0.229968	<.0001	9
**3**	**casero**	99.333333	0.229968	<.0001	10
**3**	**glass**	98.333333	0.229968	<.0001	11
**3**	**hemo**	98.656566	0.229968	<.0001	12
**4**	**casero**	99.666667	0.229968	<.0001	13
**4**	**glass**	98.646256	0.229968	<.0001	14
**4**	**hemo**	98.979521	0.229968	<.0001	15
**5**	**casero**	100.000000	0.229968	<.0001	16
**5**	**glass**	100.000000	0.229968	<.0001	17
**5**	**hemo**	99.659864	0.229968	<.0001	18
**6**	**casero**	100.000000	0.229968	<.0001	19
**6**	**glass**	99.312642	0.229968	<.0001	20
**6**	**hemo**	100.000000	0.229968	<.0001	21
**7**	**casero**	100.000000	0.229968	<.0001	22
**7**	**glass**	100.000000	0.229968	<.0001	23
**7**	**hemo**	100.000000	0.229968	<.0001	24
**8**	**casero**	100.000000	0.229968	<.0001	25
**8**	**glass**	100.000000	0.229968	<.0001	26
**8**	**hemo**	100.000000	0.229968	<.0001	27
**9**	**casero**	100.000000	0.229968	<.0001	28
**9**	**glass**	100.000000	0.229968	<.0001	29
**9**	**hemo**	100.000000	0.229968	<.0001	30
**10**	**casero**	100.000000	0.229968	<.0001	31
**10**	**glass**	100.000000	0.229968	<.0001	32

The SAS System

The GLM Procedure
Least Squares Means

Day	Device	SR LSMEAN	Standard Error	Pr > |t|	LSMEAN Number
**10**	**hemo**	100.000000	0.229968	<.0001	33
**11**	**casero**	100.000000	0.229968	<.0001	34
**11**	**glass**	100.000000	0.229968	<.0001	35
**11**	**hemo**	99.312715	0.229968	<.0001	36
**12**	**casero**	100.000000	0.229968	<.0001	37
**12**	**glass**	100.000000	0.229968	<.0001	38
**12**	**hemo**	99.305556	0.229968	<.0001	39
**13**	**casero**	99.666667	0.229968	<.0001	40
**13**	**glass**	99.656357	0.229968	<.0001	41
**13**	**hemo**	100.000000	0.229968	<.0001	42
**14**	**casero**	100.000000	0.229968	<.0001	43
**14**	**glass**	100.000000	0.229968	<.0001	44
**14**	**hemo**	99.656357	0.229968	<.0001	45
**15**	**casero**	100.000000	0.229968	<.0001	46
**15**	**glass**	100.000000	0.229968	<.0001	47
**15**	**hemo**	100.000000	0.229968	<.0001	48
**16**	**casero**	100.000000	0.229968	<.0001	49
**16**	**glass**	100.000000	0.229968	<.0001	50
**16**	**hemo**	100.000000	0.229968	<.0001	51
**17**	**casero**	100.000000	0.229968	<.0001	52
**17**	**glass**	100.000000	0.229968	<.0001	53
**17**	**hemo**	99.649123	0.229968	<.0001	54
**18**	**casero**	100.000000	0.229968	<.0001	55
**18**	**glass**	100.000000	0.229968	<.0001	56
**18**	**hemo**	100.000000	0.229968	<.0001	57
**19**	**casero**	100.000000	0.229968	<.0001	58
**19**	**glass**	100.000000	0.229968	<.0001	59
**19**	**hemo**	100.000000	0.229968	<.0001	60
**20**	**casero**	100.000000	0.229968	<.0001	61
**20**	**glass**	100.000000	0.229968	<.0001	62
**20**	**hemo**	100.000000	0.229968	<.0001	63
**21**	**casero**	100.000000	0.229968	<.0001	64

The SAS System

The GLM Procedure
Least Squares Means

Day	Device	SR LSMEAN	Standard Error	Pr > |t|	LSMEAN Number
**21**	**glass**	100.000000	0.229968	<.0001	65
**21**	**hemo**	100.000000	0.229968	<.0001	66
**22**	**casero**	99.663300	0.229968	<.0001	67
**22**	**glass**	99.656357	0.229968	<.0001	68
**22**	**hemo**	99.652778	0.229968	<.0001	69
**23**	**casero**	100.000000	0.229968	<.0001	70
**23**	**glass**	100.000000	0.229968	<.0001	71
**23**	**hemo**	100.000000	0.229968	<.0001	72
**24**	**casero**	100.000000	0.229968	<.0001	73
**24**	**glass**	100.000000	0.229968	<.0001	74
**24**	**hemo**	99.294513	0.229968	<.0001	75
**25**	**casero**	99.663300	0.229968	<.0001	76
**25**	**glass**	100.000000	0.229968	<.0001	77
**25**	**hemo**	99.645390	0.229968	<.0001	78
**26**	**casero**	100.000000	0.229968	<.0001	79
**26**	**glass**	100.000000	0.229968	<.0001	80
**26**	**hemo**	100.000000	0.229968	<.0001	81
**27**	**casero**	100.000000	0.229968	<.0001	82
**27**	**glass**	99.652778	0.229968	<.0001	83
**27**	**hemo**	99.641577	0.229968	<.0001	84
**28**	**casero**	99.659864	0.229968	<.0001	85
**28**	**glass**	100.000000	0.229968	<.0001	86
**28**	**hemo**	99.275362	0.229968	<.0001	87

The SAS System

The GLM Procedure
Least Squares Means

Least Squares Means for Effect Day*Device<br>t for H0: LSMean(i)=LSMean(j) / Pr > |t| <br>Dependent Variable: SR											
**i/j**	**1**	**2**	**3**	**4**	**5**	**6**	**7**	**8**	**9**	**10**	**11**
**1**		0<br>1.0000	0<br>1.0000	0<br>1.0000	0<br>1.0000	1.024937<br>0.3068	0<br>1.0000	0<br>1.0000	0<br>1.0000	2.049874<br>0.0419	5.124686<br><.0001
**2**	0<br>1.0000		0<br>1.0000	0<br>1.0000	0<br>1.0000	1.024937<br>0.3068	0<br>1.0000	0<br>1.0000	0<br>1.0000	2.049874<br>0.0419	5.124686<br><.0001
**3**	0<br>1.0000	0<br>1.0000		0<br>1.0000	0<br>1.0000	1.024937<br>0.3068	0<br>1.0000	0<br>1.0000	0<br>1.0000	2.049874<br>0.0419	5.124686<br><.0001
**4**	0<br>1.0000	0<br>1.0000	0<br>1.0000		0<br>1.0000	1.024937<br>0.3068	0<br>1.0000	0<br>1.0000	0<br>1.0000	2.049874<br>0.0419	5.124686<br><.0001
**5**	0<br>1.0000	0<br>1.0000	0<br>1.0000	0<br>1.0000		1.024937<br>0.3068	0<br>1.0000	0<br>1.0000	0<br>1.0000	2.049874<br>0.0419	5.124686<br><.0001
**6**	-1.02494<br>0.3068	-1.02494<br>0.3068	-1.02494<br>0.3068	-1.02494<br>0.3068	-1.02494<br>0.3068		-1.02494<br>0.3068	-1.02494<br>0.3068	-1.02494<br>0.3068	1.024937<br>0.3068	4.099749<br><.0001
**7**	0<br>1.0000	0<br>1.0000	0<br>1.0000	0<br>1.0000	0<br>1.0000	1.024937<br>0.3068		0<br>1.0000	0<br>1.0000	2.049874<br>0.0419	5.124686<br><.0001
**8**	0<br>1.0000	0<br>1.0000	0<br>1.0000	0<br>1.0000	0<br>1.0000	1.024937<br>0.3068	0<br>1.0000		0<br>1.0000	2.049874<br>0.0419	5.124686<br><.0001
**9**	0<br>1.0000	0<br>1.0000	0<br>1.0000	0<br>1.0000	0<br>1.0000	1.024937<br>0.3068	0<br>1.0000	0<br>1.0000		2.049874<br>0.0419	5.124686<br><.0001
**10**	-2.04987<br>0.0419	-2.04987<br>0.0419	-2.04987<br>0.0419	-2.04987<br>0.0419	-2.04987<br>0.0419	-1.02494<br>0.3068	-2.04987<br>0.0419	-2.04987<br>0.0419	-2.04987<br>0.0419		3.074812<br>0.0024
**11**	-5.12469<br><.0001	-5.12469<br><.0001	-5.12469<br><.0001	-5.12469<br><.0001	-5.12469<br><.0001	-4.09975<br><.0001	-5.12469<br><.0001	-5.12469<br><.0001	-5.12469<br><.0001	-3.07481<br>0.0024	
**12**	-4.13081<br><.0001	-4.13081<br><.0001	-4.13081<br><.0001	-4.13081<br><.0001	-4.13081<br><.0001	-3.10587<br>0.0022	-4.13081<br><.0001	-4.13081<br><.0001	-4.13081<br><.0001	-2.08093<br>0.0389	0.993878<br>0.3217
**13**	-1.02494<br>0.3068	-1.02494<br>0.3068	-1.02494<br>0.3068	-1.02494<br>0.3068	-1.02494<br>0.3068	0<br>1.0000	-1.02494<br>0.3068	-1.02494<br>0.3068	-1.02494<br>0.3068	1.024937<br>0.3068	4.099749<br><.0001
**14**	-4.16251<br><.0001	-4.16251<br><.0001	-4.16251<br><.0001	-4.16251<br><.0001	-4.16251<br><.0001	-3.13757<br>0.0020	-4.16251<br><.0001	-4.16251<br><.0001	-4.16251<br><.0001	-2.11263<br>0.0361	0.962179<br>0.3373
**15**	-3.13778<br>0.0020	-3.13778<br>0.0020	-3.13778<br>0.0020	-3.13778<br>0.0020	-3.13778<br>0.0020	-2.11284<br>0.0360	-3.13778<br>0.0020	-3.13778<br>0.0020	-3.13778<br>0.0020	-1.08791<br>0.2781	1.986905<br>0.0485
**16**	0<br>1.0000	0<br>1.0000	0<br>1.0000	0<br>1.0000	0<br>1.0000	1.024937<br>0.3068	0<br>1.0000	0<br>1.0000	0<br>1.0000	2.049874<br>0.0419	5.124686<br><.0001
**17**	0<br>1.0000	0<br>1.0000	0<br>1.0000	0<br>1.0000	0<br>1.0000	1.024937<br>0.3068	0<br>1.0000	0<br>1.0000	0<br>1.0000	2.049874<br>0.0419	5.124686<br><.0001
**18**	-1.04585<br>0.2971	-1.04585<br>0.2971	-1.04585<br>0.2971	-1.04585<br>0.2971	-1.04585<br>0.2971	-0.02092<br>0.9833	-1.04585<br>0.2971	-1.04585<br>0.2971	-1.04585<br>0.2971	1.00402<br>0.3168	4.078832<br><.0001
**19**	0<br>1.0000	0<br>1.0000	0<br>1.0000	0<br>1.0000	0<br>1.0000	1.024937<br>0.3068	0<br>1.0000	0<br>1.0000	0<br>1.0000	2.049874<br>0.0419	5.124686<br><.0001

The SAS System

The GLM Procedure
Least Squares Means

Least Squares Means for Effect Day*Device
t for H0: LSMean(i)=LSMean(j) / Pr > |t|

Dependent Variable: SR

i/j	1	2	3	4	5	6	7	8	9	10	11
**20**	-2.1135<br>0.0360	-2.1135<br>0.0360	-2.1135<br>0.0360	-2.1135<br>0.0360	-2.1135<br>0.0360	-1.08856<br>0.2779	-2.1135<br>0.0360	-2.1135<br>0.0360	-2.1135<br>0.0360	-0.06362<br>0.9493	3.011189<br>0.0030
**21**	0<br>1.0000	0<br>1.0000	0<br>1.0000	0<br>1.0000	0<br>1.0000	1.024937<br>0.3068	0<br>1.0000	0<br>1.0000	0<br>1.0000	2.049874<br>0.0419	5.124686<br><.0001
**22**	0<br>1.0000	0<br>1.0000	0<br>1.0000	0<br>1.0000	0<br>1.0000	1.024937<br>0.3068	0<br>1.0000	0<br>1.0000	0<br>1.0000	2.049874<br>0.0419	5.124686<br><.0001
**23**	0<br>1.0000	0<br>1.0000	0<br>1.0000	0<br>1.0000	0<br>1.0000	1.024937<br>0.3068	0<br>1.0000	0<br>1.0000	0<br>1.0000	2.049874<br>0.0419	5.124686<br><.0001
**24**	0<br>1.0000	0<br>1.0000	0<br>1.0000	0<br>1.0000	0<br>1.0000	1.024937<br>0.3068	0<br>1.0000	0<br>1.0000	0<br>1.0000	2.049874<br>0.0419	5.124686<br><.0001
**25**	0<br>1.0000	0<br>1.0000	0<br>1.0000	0<br>1.0000	0<br>1.0000	1.024937<br>0.3068	0<br>1.0000	0<br>1.0000	0<br>1.0000	2.049874<br>0.0419	5.124686<br><.0001
**26**	0<br>1.0000	0<br>1.0000	0<br>1.0000	0<br>1.0000	0<br>1.0000	1.024937<br>0.3068	0<br>1.0000	0<br>1.0000	0<br>1.0000	2.049874<br>0.0419	5.124686<br><.0001
**27**	0<br>1.0000	0<br>1.0000	0<br>1.0000	0<br>1.0000	0<br>1.0000	1.024937<br>0.3068	0<br>1.0000	0<br>1.0000	0<br>1.0000	2.049874<br>0.0419	5.124686<br><.0001
**28**	0<br>1.0000	0<br>1.0000	0<br>1.0000	0<br>1.0000	0<br>1.0000	1.024937<br>0.3068	0<br>1.0000	0<br>1.0000	0<br>1.0000	2.049874<br>0.0419	5.124686<br><.0001
**29**	0<br>1.0000	0<br>1.0000	0<br>1.0000	0<br>1.0000	0<br>1.0000	1.024937<br>0.3068	0<br>1.0000	0<br>1.0000	0<br>1.0000	2.049874<br>0.0419	5.124686<br><.0001
**30**	0<br>1.0000	0<br>1.0000	0<br>1.0000	0<br>1.0000	0<br>1.0000	1.024937<br>0.3068	0<br>1.0000	0<br>1.0000	0<br>1.0000	2.049874<br>0.0419	5.124686<br><.0001
**31**	0<br>1.0000	0<br>1.0000	0<br>1.0000	0<br>1.0000	0<br>1.0000	1.024937<br>0.3068	0<br>1.0000	0<br>1.0000	0<br>1.0000	2.049874<br>0.0419	5.124686<br><.0001
**32**	0<br>1.0000	0<br>1.0000	0<br>1.0000	0<br>1.0000	0<br>1.0000	1.024937<br>0.3068	0<br>1.0000	0<br>1.0000	0<br>1.0000	2.049874<br>0.0419	5.124686<br><.0001
**33**	0<br>1.0000	0<br>1.0000	0<br>1.0000	0<br>1.0000	0<br>1.0000	1.024937<br>0.3068	0<br>1.0000	0<br>1.0000	0<br>1.0000	2.049874<br>0.0419	5.124686<br><.0001
**34**	0<br>1.0000	0<br>1.0000	0<br>1.0000	0<br>1.0000	0<br>1.0000	1.024937<br>0.3068	0<br>1.0000	0<br>1.0000	0<br>1.0000	2.049874<br>0.0419	5.124686<br><.0001
**35**	0<br>1.0000	0<br>1.0000	0<br>1.0000	0<br>1.0000	0<br>1.0000	1.024937<br>0.3068	0<br>1.0000	0<br>1.0000	0<br>1.0000	2.049874<br>0.0419	5.124686<br><.0001
**36**	-2.11327<br>0.0360	-2.11327<br>0.0360	-2.11327<br>0.0360	-2.11327<br>0.0360	-2.11327<br>0.0360	-1.08834<br>0.2780	-2.11327<br>0.0360	-2.11327<br>0.0360	-2.11327<br>0.0360	-0.0634<br>0.9495	3.011413<br>0.0030
**37**	0<br>1.0000	0<br>1.0000	0<br>1.0000	0<br>1.0000	0<br>1.0000	1.024937<br>0.3068	0<br>1.0000	0<br>1.0000	0<br>1.0000	2.049874<br>0.0419	5.124686<br><.0001
**38**	0<br>1.0000	0<br>1.0000	0<br>1.0000	0<br>1.0000	0<br>1.0000	1.024937<br>0.3068	0<br>1.0000	0<br>1.0000	0<br>1.0000	2.049874<br>0.0419	5.124686<br><.0001

The SAS System

The GLM Procedure
Least Squares Means

Least Squares Means for Effect Day*Device
t for H0: LSMean(i)=LSMean(j) / Pr > |t|

Dependent Variable: SR

i/j	1	2	3	4	5	6	7	8	9	10	11
**39**	-2.13529<br>0.0341	-2.13529<br>0.0341	-2.13529<br>0.0341	-2.13529<br>0.0341	-2.13529<br>0.0341	-1.11035<br>0.2684	-2.13529<br>0.0341	-2.13529<br>0.0341	-2.13529<br>0.0341	-0.08541<br>0.9320	2.9894<br>0.0032
**40**	-1.02494<br>0.3068	-1.02494<br>0.3068	-1.02494<br>0.3068	-1.02494<br>0.3068	-1.02494<br>0.3068	0<br>1.0000	-1.02494<br>0.3068	-1.02494<br>0.3068	-1.02494<br>0.3068	1.024937<br>0.3068	4.099749<br><.0001
**41**	-1.05664<br>0.2921	-1.05664<br>0.2921	-1.05664<br>0.2921	-1.05664<br>0.2921	-1.05664<br>0.2921	-0.0317<br>0.9747	-1.05664<br>0.2921	-1.05664<br>0.2921	-1.05664<br>0.2921	0.993238<br>0.3220	4.06805<br><.0001
**42**	0<br>1.0000	0<br>1.0000	0<br>1.0000	0<br>1.0000	0<br>1.0000	1.024937<br>0.3068	0<br>1.0000	0<br>1.0000	0<br>1.0000	2.049874<br>0.0419	5.124686<br><.0001
**43**	0<br>1.0000	0<br>1.0000	0<br>1.0000	0<br>1.0000	0<br>1.0000	1.024937<br>0.3068	0<br>1.0000	0<br>1.0000	0<br>1.0000	2.049874<br>0.0419	5.124686<br><.0001
**44**	0<br>1.0000	0<br>1.0000	0<br>1.0000	0<br>1.0000	0<br>1.0000	1.024937<br>0.3068	0<br>1.0000	0<br>1.0000	0<br>1.0000	2.049874<br>0.0419	5.124686<br><.0001
**45**	-1.05664<br>0.2921	-1.05664<br>0.2921	-1.05664<br>0.2921	-1.05664<br>0.2921	-1.05664<br>0.2921	-0.0317<br>0.9747	-1.05664<br>0.2921	-1.05664<br>0.2921	-1.05664<br>0.2921	0.993238<br>0.3220	4.06805<br><.0001
**46**	0<br>1.0000	0<br>1.0000	0<br>1.0000	0<br>1.0000	0<br>1.0000	1.024937<br>0.3068	0<br>1.0000	0<br>1.0000	0<br>1.0000	2.049874<br>0.0419	5.124686<br><.0001
**47**	0<br>1.0000	0<br>1.0000	0<br>1.0000	0<br>1.0000	0<br>1.0000	1.024937<br>0.3068	0<br>1.0000	0<br>1.0000	0<br>1.0000	2.049874<br>0.0419	5.124686<br><.0001
**48**	0<br>1.0000	0<br>1.0000	0<br>1.0000	0<br>1.0000	0<br>1.0000	1.024937<br>0.3068	0<br>1.0000	0<br>1.0000	0<br>1.0000	2.049874<br>0.0419	5.124686<br><.0001
**49**	0<br>1.0000	0<br>1.0000	0<br>1.0000	0<br>1.0000	0<br>1.0000	1.024937<br>0.3068	0<br>1.0000	0<br>1.0000	0<br>1.0000	2.049874<br>0.0419	5.124686<br><.0001
**50**	0<br>1.0000	0<br>1.0000	0<br>1.0000	0<br>1.0000	0<br>1.0000	1.024937<br>0.3068	0<br>1.0000	0<br>1.0000	0<br>1.0000	2.049874<br>0.0419	5.124686<br><.0001
**51**	0<br>1.0000	0<br>1.0000	0<br>1.0000	0<br>1.0000	0<br>1.0000	1.024937<br>0.3068	0<br>1.0000	0<br>1.0000	0<br>1.0000	2.049874<br>0.0419	5.124686<br><.0001
**52**	0<br>1.0000	0<br>1.0000	0<br>1.0000	0<br>1.0000	0<br>1.0000	1.024937<br>0.3068	0<br>1.0000	0<br>1.0000	0<br>1.0000	2.049874<br>0.0419	5.124686<br><.0001
**53**	0<br>1.0000	0<br>1.0000	0<br>1.0000	0<br>1.0000	0<br>1.0000	1.024937<br>0.3068	0<br>1.0000	0<br>1.0000	0<br>1.0000	2.049874<br>0.0419	5.124686<br><.0001
**54**	-1.07888<br>0.2821	-1.07888<br>0.2821	-1.07888<br>0.2821	-1.07888<br>0.2821	-1.07888<br>0.2821	-0.05394<br>0.9570	-1.07888<br>0.2821	-1.07888<br>0.2821	-1.07888<br>0.2821	0.970993<br>0.3329	4.045805<br><.0001
**55**	0<br>1.0000	0<br>1.0000	0<br>1.0000	0<br>1.0000	0<br>1.0000	1.024937<br>0.3068	0<br>1.0000	0<br>1.0000	0<br>1.0000	2.049874<br>0.0419	5.124686<br><.0001
**56**	0<br>1.0000	0<br>1.0000	0<br>1.0000	0<br>1.0000	0<br>1.0000	1.024937<br>0.3068	0<br>1.0000	0<br>1.0000	0<br>1.0000	2.049874<br>0.0419	5.124686<br><.0001
**57**	0<br>1.0000	0<br>1.0000	0<br>1.0000	0<br>1.0000	0<br>1.0000	1.024937<br>0.3068	0<br>1.0000	0<br>1.0000	0<br>1.0000	2.049874<br>0.0419	5.124686<br><.0001

The SAS System

The GLM Procedure
Least Squares Means

Least Squares Means for Effect Day*Device
t for H0: LSMean(i)=LSMean(j) / Pr > |t|

Dependent Variable: SR

i/j	1	2	3	4	5	6	7	8	9	10	11
**58**	0<br>1.0000	0<br>1.0000	0<br>1.0000	0<br>1.0000	0<br>1.0000	1.024937<br>0.3068	0<br>1.0000	0<br>1.0000	0<br>1.0000	2.049874<br>0.0419	5.124686<br><.0001
**59**	0<br>1.0000	0<br>1.0000	0<br>1.0000	0<br>1.0000	0<br>1.0000	1.024937<br>0.3068	0<br>1.0000	0<br>1.0000	0<br>1.0000	2.049874<br>0.0419	5.124686<br><.0001
**60**	0<br>1.0000	0<br>1.0000	0<br>1.0000	0<br>1.0000	0<br>1.0000	1.024937<br>0.3068	0<br>1.0000	0<br>1.0000	0<br>1.0000	2.049874<br>0.0419	5.124686<br><.0001
**61**	0<br>1.0000	0<br>1.0000	0<br>1.0000	0<br>1.0000	0<br>1.0000	1.024937<br>0.3068	0<br>1.0000	0<br>1.0000	0<br>1.0000	2.049874<br>0.0419	5.124686<br><.0001
**62**	0<br>1.0000	0<br>1.0000	0<br>1.0000	0<br>1.0000	0<br>1.0000	1.024937<br>0.3068	0<br>1.0000	0<br>1.0000	0<br>1.0000	2.049874<br>0.0419	5.124686<br><.0001
**63**	0<br>1.0000	0<br>1.0000	0<br>1.0000	0<br>1.0000	0<br>1.0000	1.024937<br>0.3068	0<br>1.0000	0<br>1.0000	0<br>1.0000	2.049874<br>0.0419	5.124686<br><.0001
**64**	0<br>1.0000	0<br>1.0000	0<br>1.0000	0<br>1.0000	0<br>1.0000	1.024937<br>0.3068	0<br>1.0000	0<br>1.0000	0<br>1.0000	2.049874<br>0.0419	5.124686<br><.0001
**65**	0<br>1.0000	0<br>1.0000	0<br>1.0000	0<br>1.0000	0<br>1.0000	1.024937<br>0.3068	0<br>1.0000	0<br>1.0000	0<br>1.0000	2.049874<br>0.0419	5.124686<br><.0001
**66**	0<br>1.0000	0<br>1.0000	0<br>1.0000	0<br>1.0000	0<br>1.0000	1.024937<br>0.3068	0<br>1.0000	0<br>1.0000	0<br>1.0000	2.049874<br>0.0419	5.124686<br><.0001
**67**	-1.03529<br>0.3020	-1.03529<br>0.3020	-1.03529<br>0.3020	-1.03529<br>0.3020	-1.03529<br>0.3020	-0.01035<br>0.9918	-1.03529<br>0.3020	-1.03529<br>0.3020	-1.03529<br>0.3020	1.014584<br>0.3117	4.089396<br><.0001
**68**	-1.05664<br>0.2921	-1.05664<br>0.2921	-1.05664<br>0.2921	-1.05664<br>0.2921	-1.05664<br>0.2921	-0.0317<br>0.9747	-1.05664<br>0.2921	-1.05664<br>0.2921	-1.05664<br>0.2921	0.993238<br>0.3220	4.06805<br><.0001
**69**	-1.06764<br>0.2872	-1.06764<br>0.2872	-1.06764<br>0.2872	-1.06764<br>0.2872	-1.06764<br>0.2872	-0.04271<br>0.9660	-1.06764<br>0.2872	-1.06764<br>0.2872	-1.06764<br>0.2872	0.982231<br>0.3273	4.057043<br><.0001
**70**	0<br>1.0000	0<br>1.0000	0<br>1.0000	0<br>1.0000	0<br>1.0000	1.024937<br>0.3068	0<br>1.0000	0<br>1.0000	0<br>1.0000	2.049874<br>0.0419	5.124686<br><.0001
**71**	0<br>1.0000	0<br>1.0000	0<br>1.0000	0<br>1.0000	0<br>1.0000	1.024937<br>0.3068	0<br>1.0000	0<br>1.0000	0<br>1.0000	2.049874<br>0.0419	5.124686<br><.0001
**72**	0<br>1.0000	0<br>1.0000	0<br>1.0000	0<br>1.0000	0<br>1.0000	1.024937<br>0.3068	0<br>1.0000	0<br>1.0000	0<br>1.0000	2.049874<br>0.0419	5.124686<br><.0001
**73**	0<br>1.0000	0<br>1.0000	0<br>1.0000	0<br>1.0000	0<br>1.0000	1.024937<br>0.3068	0<br>1.0000	0<br>1.0000	0<br>1.0000	2.049874<br>0.0419	5.124686<br><.0001
**74**	0<br>1.0000	0<br>1.0000	0<br>1.0000	0<br>1.0000	0<br>1.0000	1.024937<br>0.3068	0<br>1.0000	0<br>1.0000	0<br>1.0000	2.049874<br>0.0419	5.124686<br><.0001
**75**	-2.16924<br>0.0314	-2.16924<br>0.0314	-2.16924<br>0.0314	-2.16924<br>0.0314	-2.16924<br>0.0314	-1.1443<br>0.2541	-2.16924<br>0.0314	-2.16924<br>0.0314	-2.16924<br>0.0314	-0.11937<br>0.9051	2.955446<br>0.0036
**76**	-1.03529<br>0.3020	-1.03529<br>0.3020	-1.03529<br>0.3020	-1.03529<br>0.3020	-1.03529<br>0.3020	-0.01035<br>0.9918	-1.03529<br>0.3020	-1.03529<br>0.3020	-1.03529<br>0.3020	1.014584<br>0.3117	4.089396<br><.0001

The SAS System

The GLM Procedure
Least Squares Means

Least Squares Means for Effect Day*Device
t for H0: LSMean(i)=LSMean(j) / Pr > |t|

Dependent Variable: SR

i/j	1	2	3	4	5	6	7	8	9	10	11
**77**	0<br>1.0000	0<br>1.0000	0<br>1.0000	0<br>1.0000	0<br>1.0000	1.024937<br>0.3068	0<br>1.0000	0<br>1.0000	0<br>1.0000	2.049874<br>0.0419	5.124686<br><.0001
**78**	-1.09036<br>0.2771	-1.09036<br>0.2771	-1.09036<br>0.2771	-1.09036<br>0.2771	-1.09036<br>0.2771	-0.06542<br>0.9479	-1.09036<br>0.2771	-1.09036<br>0.2771	-1.09036<br>0.2771	0.959516<br>0.3386	4.034327<br><.0001
**79**	0<br>1.0000	0<br>1.0000	0<br>1.0000	0<br>1.0000	0<br>1.0000	1.024937<br>0.3068	0<br>1.0000	0<br>1.0000	0<br>1.0000	2.049874<br>0.0419	5.124686<br><.0001
**80**	0<br>1.0000	0<br>1.0000	0<br>1.0000	0<br>1.0000	0<br>1.0000	1.024937<br>0.3068	0<br>1.0000	0<br>1.0000	0<br>1.0000	2.049874<br>0.0419	5.124686<br><.0001
**81**	0<br>1.0000	0<br>1.0000	0<br>1.0000	0<br>1.0000	0<br>1.0000	1.024937<br>0.3068	0<br>1.0000	0<br>1.0000	0<br>1.0000	2.049874<br>0.0419	5.124686<br><.0001
**82**	0<br>1.0000	0<br>1.0000	0<br>1.0000	0<br>1.0000	0<br>1.0000	1.024937<br>0.3068	0<br>1.0000	0<br>1.0000	0<br>1.0000	2.049874<br>0.0419	5.124686<br><.0001
**83**	-1.06764<br>0.2872	-1.06764<br>0.2872	-1.06764<br>0.2872	-1.06764<br>0.2872	-1.06764<br>0.2872	-0.04271<br>0.9660	-1.06764<br>0.2872	-1.06764<br>0.2872	-1.06764<br>0.2872	0.982231<br>0.3273	4.057043<br><.0001
**84**	-1.10208<br>0.2719	-1.10208<br>0.2719	-1.10208<br>0.2719	-1.10208<br>0.2719	-1.10208<br>0.2719	-0.07715<br>0.9386	-1.10208<br>0.2719	-1.10208<br>0.2719	-1.10208<br>0.2719	0.947791<br>0.3446	4.022603<br><.0001
**85**	-1.04585<br>0.2971	-1.04585<br>0.2971	-1.04585<br>0.2971	-1.04585<br>0.2971	-1.04585<br>0.2971	-0.02092<br>0.9833	-1.04585<br>0.2971	-1.04585<br>0.2971	-1.04585<br>0.2971	1.00402<br>0.3168	4.078832<br><.0001
**86**	0<br>1.0000	0<br>1.0000	0<br>1.0000	0<br>1.0000	0<br>1.0000	1.024937<br>0.3068	0<br>1.0000	0<br>1.0000	0<br>1.0000	2.049874<br>0.0419	5.124686<br><.0001
**87**	-2.22812<br>0.0272	-2.22812<br>0.0272	-2.22812<br>0.0272	-2.22812<br>0.0272	-2.22812<br>0.0272	-1.20319<br>0.2305	-2.22812<br>0.0272	-2.22812<br>0.0272	-2.22812<br>0.0272	-0.17825<br>0.8587	2.896562<br>0.0043

Least Squares Means for Effect Day*Device
t for H0: LSMean(i)=LSMean(j) / Pr > |t|

Dependent Variable: SR

i/j	12	13	14	15	16	17	18	19	20	21	22
**1**	4.130807<br><.0001	1.024937<br>0.3068	4.162506<br><.0001	3.137781<br>0.0020	0<br>1.0000	0<br>1.0000	1.045854<br>0.2971	0<br>1.0000	2.113497<br>0.0360	0<br>1.0000	0<br>1.0000
**2**	4.130807<br><.0001	1.024937<br>0.3068	4.162506<br><.0001	3.137781<br>0.0020	0<br>1.0000	0<br>1.0000	1.045854<br>0.2971	0<br>1.0000	2.113497<br>0.0360	0<br>1.0000	0<br>1.0000
**3**	4.130807<br><.0001	1.024937<br>0.3068	4.162506<br><.0001	3.137781<br>0.0020	0<br>1.0000	0<br>1.0000	1.045854<br>0.2971	0<br>1.0000	2.113497<br>0.0360	0<br>1.0000	0<br>1.0000
**4**	4.130807<br><.0001	1.024937<br>0.3068	4.162506<br><.0001	3.137781<br>0.0020	0<br>1.0000	0<br>1.0000	1.045854<br>0.2971	0<br>1.0000	2.113497<br>0.0360	0<br>1.0000	0<br>1.0000
**5**	4.130807<br><.0001	1.024937<br>0.3068	4.162506<br><.0001	3.137781<br>0.0020	0<br>1.0000	0<br>1.0000	1.045854<br>0.2971	0<br>1.0000	2.113497<br>0.0360	0<br>1.0000	0<br>1.0000

The SAS System

The GLM Procedure
Least Squares Means

Least Squares Means for Effect Day*Device
t for H0: LSMean(i)=LSMean(j) / Pr > |t|

Dependent Variable: SR

i/j	12	13	14	15	16	17	18	19	20	21	22
**6**	3.10587<br>0.0022	0<br>1.0000	3.137569<br>0.0020	2.112843<br>0.0360	-1.02494<br>0.3068	-1.02494<br>0.3068	0.020917<br>0.9833	-1.02494<br>0.3068	1.08856<br>0.2779	-1.02494<br>0.3068	-1.02494<br>0.3068
**7**	4.130807<br><.0001	1.024937<br>0.3068	4.162506<br><.0001	3.137781<br>0.0020	0<br>1.0000	0<br>1.0000	1.045854<br>0.2971	0<br>1.0000	2.113497<br>0.0360	0<br>1.0000	0<br>1.0000
**8**	4.130807<br><.0001	1.024937<br>0.3068	4.162506<br><.0001	3.137781<br>0.0020	0<br>1.0000	0<br>1.0000	1.045854<br>0.2971	0<br>1.0000	2.113497<br>0.0360	0<br>1.0000	0<br>1.0000
**9**	4.130807<br><.0001	1.024937<br>0.3068	4.162506<br><.0001	3.137781<br>0.0020	0<br>1.0000	0<br>1.0000	1.045854<br>0.2971	0<br>1.0000	2.113497<br>0.0360	0<br>1.0000	0<br>1.0000
**10**	2.080933<br>0.0389	-1.02494<br>0.3068	2.112632<br>0.0361	1.087906<br>0.2781	-2.04987<br>0.0419	-2.04987<br>0.0419	-1.00402<br>0.3168	-2.04987<br>0.0419	0.063623<br>0.9493	-2.04987<br>0.0419	-2.04987<br>0.0419
**11**	-0.99388<br>0.3217	-4.09975<br><.0001	-0.96218<br>0.3373	-1.98691<br>0.0485	-5.12469<br><.0001	-5.12469<br><.0001	-4.07883<br><.0001	-5.12469<br><.0001	-3.01119<br>0.0030	-5.12469<br><.0001	-5.12469<br><.0001
**12**		-3.10587<br>0.0022	0.031699<br>0.9747	-0.99303<br>0.3221	-4.13081<br><.0001	-4.13081<br><.0001	-3.08495<br>0.0024	-4.13081<br><.0001	-2.01731<br>0.0452	-4.13081<br><.0001	-4.13081<br><.0001
**13**	3.10587<br>0.0022		3.137569<br>0.0020	2.112843<br>0.0360	-1.02494<br>0.3068	-1.02494<br>0.3068	0.020917<br>0.9833	-1.02494<br>0.3068	1.08856<br>0.2779	-1.02494<br>0.3068	-1.02494<br>0.3068
**14**	-0.0317<br>0.9747	-3.13757<br>0.0020		-1.02473<br>0.3069	-4.16251<br><.0001	-4.16251<br><.0001	-3.11665<br>0.0021	-4.16251<br><.0001	-2.04901<br>0.0420	-4.16251<br><.0001	-4.16251<br><.0001
**15**	0.993027<br>0.3221	-2.11284<br>0.0360	1.024726<br>0.3069		-3.13778<br>0.0020	-3.13778<br>0.0020	-2.09193<br>0.0379	-3.13778<br>0.0020	-1.02428<br>0.3071	-3.13778<br>0.0020	-3.13778<br>0.0020
**16**	4.130807<br><.0001	1.024937<br>0.3068	4.162506<br><.0001	3.137781<br>0.0020		0<br>1.0000	1.045854<br>0.2971	0<br>1.0000	2.113497<br>0.0360	0<br>1.0000	0<br>1.0000
**17**	4.130807<br><.0001	1.024937<br>0.3068	4.162506<br><.0001	3.137781<br>0.0020	0<br>1.0000		1.045854<br>0.2971	0<br>1.0000	2.113497<br>0.0360	0<br>1.0000	0<br>1.0000
**18**	3.084953<br>0.0024	-0.02092<br>0.9833	3.116652<br>0.0021	2.091926<br>0.0379	-1.04585<br>0.2971	-1.04585<br>0.2971		-1.04585<br>0.2971	1.067643<br>0.2872	-1.04585<br>0.2971	-1.04585<br>0.2971
**19**	4.130807<br><.0001	1.024937<br>0.3068	4.162506<br><.0001	3.137781<br>0.0020	0<br>1.0000	0<br>1.0000	1.045854<br>0.2971		2.113497<br>0.0360	0<br>1.0000	0<br>1.0000
**20**	2.01731<br>0.0452	-1.08856<br>0.2779	2.049009<br>0.0420	1.024283<br>0.3071	-2.1135<br>0.0360	-2.1135<br>0.0360	-1.06764<br>0.2872	-2.1135<br>0.0360		-2.1135<br>0.0360	-2.1135<br>0.0360
**21**	4.130807<br><.0001	1.024937<br>0.3068	4.162506<br><.0001	3.137781<br>0.0020	0<br>1.0000	0<br>1.0000	1.045854<br>0.2971	0<br>1.0000	2.113497<br>0.0360		0<br>1.0000
**22**	4.130807<br><.0001	1.024937<br>0.3068	4.162506<br><.0001	3.137781<br>0.0020	0<br>1.0000	0<br>1.0000	1.045854<br>0.2971	0<br>1.0000	2.113497<br>0.0360	0<br>1.0000	
**23**	4.130807<br><.0001	1.024937<br>0.3068	4.162506<br><.0001	3.137781<br>0.0020	0<br>1.0000	0<br>1.0000	1.045854<br>0.2971	0<br>1.0000	2.113497<br>0.0360	0<br>1.0000	0<br>1.0000
**24**	4.130807<br><.0001	1.024937<br>0.3068	4.162506<br><.0001	3.137781<br>0.0020	0<br>1.0000	0<br>1.0000	1.045854<br>0.2971	0<br>1.0000	2.113497<br>0.0360	0<br>1.0000	0<br>1.0000

The SAS System

The GLM Procedure
Least Squares Means

Least Squares Means for Effect Day*Device
t for H0: LSMean(i)=LSMean(j) / Pr > |t|

Dependent Variable: SR

i/j	12	13	14	15	16	17	18	19	20	21	22
**25**	4.130807<br><.0001	1.024937<br>0.3068	4.162506<br><.0001	3.137781<br>0.0020	0<br>1.0000	0<br>1.0000	1.045854<br>0.2971	0<br>1.0000	2.113497<br>0.0360	0<br>1.0000	0<br>1.0000
**26**	4.130807<br><.0001	1.024937<br>0.3068	4.162506<br><.0001	3.137781<br>0.0020	0<br>1.0000	0<br>1.0000	1.045854<br>0.2971	0<br>1.0000	2.113497<br>0.0360	0<br>1.0000	0<br>1.0000
**27**	4.130807<br><.0001	1.024937<br>0.3068	4.162506<br><.0001	3.137781<br>0.0020	0<br>1.0000	0<br>1.0000	1.045854<br>0.2971	0<br>1.0000	2.113497<br>0.0360	0<br>1.0000	0<br>1.0000
**28**	4.130807<br><.0001	1.024937<br>0.3068	4.162506<br><.0001	3.137781<br>0.0020	0<br>1.0000	0<br>1.0000	1.045854<br>0.2971	0<br>1.0000	2.113497<br>0.0360	0<br>1.0000	0<br>1.0000
**29**	4.130807<br><.0001	1.024937<br>0.3068	4.162506<br><.0001	3.137781<br>0.0020	0<br>1.0000	0<br>1.0000	1.045854<br>0.2971	0<br>1.0000	2.113497<br>0.0360	0<br>1.0000	0<br>1.0000
**30**	4.130807<br><.0001	1.024937<br>0.3068	4.162506<br><.0001	3.137781<br>0.0020	0<br>1.0000	0<br>1.0000	1.045854<br>0.2971	0<br>1.0000	2.113497<br>0.0360	0<br>1.0000	0<br>1.0000
**31**	4.130807<br><.0001	1.024937<br>0.3068	4.162506<br><.0001	3.137781<br>0.0020	0<br>1.0000	0<br>1.0000	1.045854<br>0.2971	0<br>1.0000	2.113497<br>0.0360	0<br>1.0000	0<br>1.0000
**32**	4.130807<br><.0001	1.024937<br>0.3068	4.162506<br><.0001	3.137781<br>0.0020	0<br>1.0000	0<br>1.0000	1.045854<br>0.2971	0<br>1.0000	2.113497<br>0.0360	0<br>1.0000	0<br>1.0000
**33**	4.130807<br><.0001	1.024937<br>0.3068	4.162506<br><.0001	3.137781<br>0.0020	0<br>1.0000	0<br>1.0000	1.045854<br>0.2971	0<br>1.0000	2.113497<br>0.0360	0<br>1.0000	0<br>1.0000
**34**	4.130807<br><.0001	1.024937<br>0.3068	4.162506<br><.0001	3.137781<br>0.0020	0<br>1.0000	0<br>1.0000	1.045854<br>0.2971	0<br>1.0000	2.113497<br>0.0360	0<br>1.0000	0<br>1.0000
**35**	4.130807<br><.0001	1.024937<br>0.3068	4.162506<br><.0001	3.137781<br>0.0020	0<br>1.0000	0<br>1.0000	1.045854<br>0.2971	0<br>1.0000	2.113497<br>0.0360	0<br>1.0000	0<br>1.0000
**36**	2.017535<br>0.0452	-1.08834<br>0.2780	2.049234<br>0.0419	1.024508<br>0.3070	-2.11327<br>0.0360	-2.11327<br>0.0360	-1.06742<br>0.2873	-2.11327<br>0.0360	0.000225<br>0.9998	-2.11327<br>0.0360	-2.11327<br>0.0360
**37**	4.130807<br><.0001	1.024937<br>0.3068	4.162506<br><.0001	3.137781<br>0.0020	0<br>1.0000	0<br>1.0000	1.045854<br>0.2971	0<br>1.0000	2.113497<br>0.0360	0<br>1.0000	0<br>1.0000
**38**	4.130807<br><.0001	1.024937<br>0.3068	4.162506<br><.0001	3.137781<br>0.0020	0<br>1.0000	0<br>1.0000	1.045854<br>0.2971	0<br>1.0000	2.113497<br>0.0360	0<br>1.0000	0<br>1.0000
**39**	1.995522<br>0.0475	-1.11035<br>0.2684	2.027221<br>0.0442	1.002495<br>0.3175	-2.13529<br>0.0341	-2.13529<br>0.0341	-1.08943<br>0.2775	-2.13529<br>0.0341	-0.02179<br>0.9826	-2.13529<br>0.0341	-2.13529<br>0.0341
**40**	3.10587<br>0.0022	0<br>1.0000	3.137569<br>0.0020	2.112843<br>0.0360	-1.02494<br>0.3068	-1.02494<br>0.3068	0.020917<br>0.9833	-1.02494<br>0.3068	1.08856<br>0.2779	-1.02494<br>0.3068	-1.02494<br>0.3068
**41**	3.074171<br>0.0025	-0.0317<br>0.9747	3.10587<br>0.0022	2.081144<br>0.0389	-1.05664<br>0.2921	-1.05664<br>0.2921	-0.01078<br>0.9914	-1.05664<br>0.2921	1.056861<br>0.2920	-1.05664<br>0.2921	-1.05664<br>0.2921
**42**	4.130807<br><.0001	1.024937<br>0.3068	4.162506<br><.0001	3.137781<br>0.0020	0<br>1.0000	0<br>1.0000	1.045854<br>0.2971	0<br>1.0000	2.113497<br>0.0360	0<br>1.0000	0<br>1.0000
**43**	4.130807<br><.0001	1.024937<br>0.3068	4.162506<br><.0001	3.137781<br>0.0020	0<br>1.0000	0<br>1.0000	1.045854<br>0.2971	0<br>1.0000	2.113497<br>0.0360	0<br>1.0000	0<br>1.0000

The SAS System

The GLM Procedure
Least Squares Means

Least Squares Means for Effect Day*Device
t for H0: LSMean(i)=LSMean(j) / Pr > |t|

Dependent Variable: SR

i/j	12	13	14	15	16	17	18	19	20	21	22
**44**	4.130807<br><.0001	1.024937<br>0.3068	4.162506<br><.0001	3.137781<br>0.0020	0<br>1.0000	0<br>1.0000	1.045854<br>0.2971	0<br>1.0000	2.113497<br>0.0360	0<br>1.0000	0<br>1.0000
**45**	3.074171<br>0.0025	-0.0317<br>0.9747	3.10587<br>0.0022	2.081144<br>0.0389	-1.05664<br>0.2921	-1.05664<br>0.2921	-0.01078<br>0.9914	-1.05664<br>0.2921	1.056861<br>0.2920	-1.05664<br>0.2921	-1.05664<br>0.2921
**46**	4.130807<br><.0001	1.024937<br>0.3068	4.162506<br><.0001	3.137781<br>0.0020	0<br>1.0000	0<br>1.0000	1.045854<br>0.2971	0<br>1.0000	2.113497<br>0.0360	0<br>1.0000	0<br>1.0000
**47**	4.130807<br><.0001	1.024937<br>0.3068	4.162506<br><.0001	3.137781<br>0.0020	0<br>1.0000	0<br>1.0000	1.045854<br>0.2971	0<br>1.0000	2.113497<br>0.0360	0<br>1.0000	0<br>1.0000
**48**	4.130807<br><.0001	1.024937<br>0.3068	4.162506<br><.0001	3.137781<br>0.0020	0<br>1.0000	0<br>1.0000	1.045854<br>0.2971	0<br>1.0000	2.113497<br>0.0360	0<br>1.0000	0<br>1.0000
**49**	4.130807<br><.0001	1.024937<br>0.3068	4.162506<br><.0001	3.137781<br>0.0020	0<br>1.0000	0<br>1.0000	1.045854<br>0.2971	0<br>1.0000	2.113497<br>0.0360	0<br>1.0000	0<br>1.0000
**50**	4.130807<br><.0001	1.024937<br>0.3068	4.162506<br><.0001	3.137781<br>0.0020	0<br>1.0000	0<br>1.0000	1.045854<br>0.2971	0<br>1.0000	2.113497<br>0.0360	0<br>1.0000	0<br>1.0000
**51**	4.130807<br><.0001	1.024937<br>0.3068	4.162506<br><.0001	3.137781<br>0.0020	0<br>1.0000	0<br>1.0000	1.045854<br>0.2971	0<br>1.0000	2.113497<br>0.0360	0<br>1.0000	0<br>1.0000
**52**	4.130807<br><.0001	1.024937<br>0.3068	4.162506<br><.0001	3.137781<br>0.0020	0<br>1.0000	0<br>1.0000	1.045854<br>0.2971	0<br>1.0000	2.113497<br>0.0360	0<br>1.0000	0<br>1.0000
**53**	4.130807<br><.0001	1.024937<br>0.3068	4.162506<br><.0001	3.137781<br>0.0020	0<br>1.0000	0<br>1.0000	1.045854<br>0.2971	0<br>1.0000	2.113497<br>0.0360	0<br>1.0000	0<br>1.0000
**54**	3.051926<br>0.0026	-0.05394<br>0.9570	3.083625<br>0.0024	2.058899<br>0.0410	-1.07888<br>0.2821	-1.07888<br>0.2821	-0.03303<br>0.9737	-1.07888<br>0.2821	1.034616<br>0.3023	-1.07888<br>0.2821	-1.07888<br>0.2821
**55**	4.130807<br><.0001	1.024937<br>0.3068	4.162506<br><.0001	3.137781<br>0.0020	0<br>1.0000	0<br>1.0000	1.045854<br>0.2971	0<br>1.0000	2.113497<br>0.0360	0<br>1.0000	0<br>1.0000
**56**	4.130807<br><.0001	1.024937<br>0.3068	4.162506<br><.0001	3.137781<br>0.0020	0<br>1.0000	0<br>1.0000	1.045854<br>0.2971	0<br>1.0000	2.113497<br>0.0360	0<br>1.0000	0<br>1.0000
**57**	4.130807<br><.0001	1.024937<br>0.3068	4.162506<br><.0001	3.137781<br>0.0020	0<br>1.0000	0<br>1.0000	1.045854<br>0.2971	0<br>1.0000	2.113497<br>0.0360	0<br>1.0000	0<br>1.0000
**58**	4.130807<br><.0001	1.024937<br>0.3068	4.162506<br><.0001	3.137781<br>0.0020	0<br>1.0000	0<br>1.0000	1.045854<br>0.2971	0<br>1.0000	2.113497<br>0.0360	0<br>1.0000	0<br>1.0000
**59**	4.130807<br><.0001	1.024937<br>0.3068	4.162506<br><.0001	3.137781<br>0.0020	0<br>1.0000	0<br>1.0000	1.045854<br>0.2971	0<br>1.0000	2.113497<br>0.0360	0<br>1.0000	0<br>1.0000
**60**	4.130807<br><.0001	1.024937<br>0.3068	4.162506<br><.0001	3.137781<br>0.0020	0<br>1.0000	0<br>1.0000	1.045854<br>0.2971	0<br>1.0000	2.113497<br>0.0360	0<br>1.0000	0<br>1.0000
**61**	4.130807<br><.0001	1.024937<br>0.3068	4.162506<br><.0001	3.137781<br>0.0020	0<br>1.0000	0<br>1.0000	1.045854<br>0.2971	0<br>1.0000	2.113497<br>0.0360	0<br>1.0000	0<br>1.0000
**62**	4.130807<br><.0001	1.024937<br>0.3068	4.162506<br><.0001	3.137781<br>0.0020	0<br>1.0000	0<br>1.0000	1.045854<br>0.2971	0<br>1.0000	2.113497<br>0.0360	0<br>1.0000	0<br>1.0000

The SAS System

The GLM Procedure
Least Squares Means

Least Squares Means for Effect Day*Device
t for H0: LSMean(i)=LSMean(j) / Pr > |t|

Dependent Variable: SR

i/j	12	13	14	15	16	17	18	19	20	21	22
63	4.130807<br><.0001	1.024937<br>0.3068	4.162506<br><.0001	3.137781<br>0.0020	0<br>1.0000	0<br>1.0000	1.045854<br>0.2971	0<br>1.0000	2.113497<br>0.0360	0<br>1.0000	0<br>1.0000
64	4.130807<br><.0001	1.024937<br>0.3068	4.162506<br><.0001	3.137781<br>0.0020	0<br>1.0000	0<br>1.0000	1.045854<br>0.2971	0<br>1.0000	2.113497<br>0.0360	0<br>1.0000	0<br>1.0000
65	4.130807<br><.0001	1.024937<br>0.3068	4.162506<br><.0001	3.137781<br>0.0020	0<br>1.0000	0<br>1.0000	1.045854<br>0.2971	0<br>1.0000	2.113497<br>0.0360	0<br>1.0000	0<br>1.0000
66	4.130807<br><.0001	1.024937<br>0.3068	4.162506<br><.0001	3.137781<br>0.0020	0<br>1.0000	0<br>1.0000	1.045854<br>0.2971	0<br>1.0000	2.113497<br>0.0360	0<br>1.0000	0<br>1.0000
67	3.095517<br>0.0023	-0.01035<br>0.9918	3.127216<br>0.0021	2.102491<br>0.0369	-1.03529<br>0.3020	-1.03529<br>0.3020	0.010564<br>0.9916	-1.03529<br>0.3020	1.078207<br>0.2824	-1.03529<br>0.3020	-1.03529<br>0.3020
68	3.074171<br>0.0025	-0.0317<br>0.9747	3.10587<br>0.0022	2.081144<br>0.0389	-1.05664<br>0.2921	-1.05664<br>0.2921	-0.01078<br>0.9914	-1.05664<br>0.2921	1.056861<br>0.2920	-1.05664<br>0.2921	-1.05664<br>0.2921
69	3.063165<br>0.0025	-0.04271<br>0.9660	3.094864<br>0.0023	2.070138<br>0.0399	-1.06764<br>0.2872	-1.06764<br>0.2872	-0.02179<br>0.9826	-1.06764<br>0.2872	1.045854<br>0.2971	-1.06764<br>0.2872	-1.06764<br>0.2872
70	4.130807<br><.0001	1.024937<br>0.3068	4.162506<br><.0001	3.137781<br>0.0020	0<br>1.0000	0<br>1.0000	1.045854<br>0.2971	0<br>1.0000	2.113497<br>0.0360	0<br>1.0000	0<br>1.0000
71	4.130807<br><.0001	1.024937<br>0.3068	4.162506<br><.0001	3.137781<br>0.0020	0<br>1.0000	0<br>1.0000	1.045854<br>0.2971	0<br>1.0000	2.113497<br>0.0360	0<br>1.0000	0<br>1.0000
72	4.130807<br><.0001	1.024937<br>0.3068	4.162506<br><.0001	3.137781<br>0.0020	0<br>1.0000	0<br>1.0000	1.045854<br>0.2971	0<br>1.0000	2.113497<br>0.0360	0<br>1.0000	0<br>1.0000
73	4.130807<br><.0001	1.024937<br>0.3068	4.162506<br><.0001	3.137781<br>0.0020	0<br>1.0000	0<br>1.0000	1.045854<br>0.2971	0<br>1.0000	2.113497<br>0.0360	0<br>1.0000	0<br>1.0000
74	4.130807<br><.0001	1.024937<br>0.3068	4.162506<br><.0001	3.137781<br>0.0020	0<br>1.0000	0<br>1.0000	1.045854<br>0.2971	0<br>1.0000	2.113497<br>0.0360	0<br>1.0000	0<br>1.0000
75	1.961567<br>0.0514	-1.1443<br>0.2541	1.993267<br>0.0478	0.968541<br>0.3341	-2.16924<br>0.0314	-2.16924<br>0.0314	-1.12339<br>0.2628	-2.16924<br>0.0314	-0.05574<br>0.9556	-2.16924<br>0.0314	-2.16924<br>0.0314
76	3.095517<br>0.0023	-0.01035<br>0.9918	3.127216<br>0.0021	2.102491<br>0.0369	-1.03529<br>0.3020	-1.03529<br>0.3020	0.010564<br>0.9916	-1.03529<br>0.3020	1.078207<br>0.2824	-1.03529<br>0.3020	-1.03529<br>0.3020
77	4.130807<br><.0001	1.024937<br>0.3068	4.162506<br><.0001	3.137781<br>0.0020	0<br>1.0000	0<br>1.0000	1.045854<br>0.2971	0<br>1.0000	2.113497<br>0.0360	0<br>1.0000	0<br>1.0000
78	3.040449<br>0.0027	-0.06542<br>0.9479	3.072148<br>0.0025	2.047422<br>0.0421	-1.09036<br>0.2771	-1.09036<br>0.2771	-0.0445<br>0.9646	-1.09036<br>0.2771	1.023138<br>0.3077	-1.09036<br>0.2771	-1.09036<br>0.2771
79	4.130807<br><.0001	1.024937<br>0.3068	4.162506<br><.0001	3.137781<br>0.0020	0<br>1.0000	0<br>1.0000	1.045854<br>0.2971	0<br>1.0000	2.113497<br>0.0360	0<br>1.0000	0<br>1.0000
80	4.130807<br><.0001	1.024937<br>0.3068	4.162506<br><.0001	3.137781<br>0.0020	0<br>1.0000	0<br>1.0000	1.045854<br>0.2971	0<br>1.0000	2.113497<br>0.0360	0<br>1.0000	0<br>1.0000
81	4.130807<br><.0001	1.024937<br>0.3068	4.162506<br><.0001	3.137781<br>0.0020	0<br>1.0000	0<br>1.0000	1.045854<br>0.2971	0<br>1.0000	2.113497<br>0.0360	0<br>1.0000	0<br>1.0000

The SAS System

The GLM Procedure
Least Squares Means

Least Squares Means for Effect Day*Device
t for H0: LSMean(i)=LSMean(j) / Pr > |t|

Dependent Variable: SR

i/j	12	13	14	15	16	17	18	19	20	21	22
**82**	4.130807<br><.0001	1.024937<br>0.3068	4.162506<br><.0001	3.137781<br>0.0020	0<br>1.0000	0<br>1.0000	1.045854<br>0.2971	0<br>1.0000	2.113497<br>0.0360	0<br>1.0000	0<br>1.0000
**83**	3.063165<br>0.0025	-0.04271<br>0.9660	3.094864<br>0.0023	2.070138<br>0.0399	-1.06764<br>0.2872	-1.06764<br>0.2872	-0.02179<br>0.9826	-1.06764<br>0.2872	1.045854<br>0.2971	-1.06764<br>0.2872	-1.06764<br>0.2872
**84**	3.028724<br>0.0028	-0.07715<br>0.9386	3.060424<br>0.0026	2.035698<br>0.0433	-1.10208<br>0.2719	-1.10208<br>0.2719	-0.05623<br>0.9552	-1.10208<br>0.2719	1.011414<br>0.3132	-1.10208<br>0.2719	-1.10208<br>0.2719
**85**	3.084953<br>0.0024	-0.02092<br>0.9833	3.116652<br>0.0021	2.091926<br>0.0379	-1.04585<br>0.2971	-1.04585<br>0.2971	0<br>1.0000	-1.04585<br>0.2971	1.067643<br>0.2872	-1.04585<br>0.2971	-1.04585<br>0.2971
**86**	4.130807<br><.0001	1.024937<br>0.3068	4.162506<br><.0001	3.137781<br>0.0020	0<br>1.0000	0<br>1.0000	1.045854<br>0.2971	0<br>1.0000	2.113497<br>0.0360	0<br>1.0000	0<br>1.0000
**87**	1.902683<br>0.0587	-1.20319<br>0.2305	1.934382<br>0.0547	0.909656<br>0.3643	-2.22812<br>0.0272	-2.22812<br>0.0272	-1.18227<br>0.2387	-2.22812<br>0.0272	-0.11463<br>0.9089	-2.22812<br>0.0272	-2.22812<br>0.0272

Least Squares Means for Effect Day*Device
t for H0: LSMean(i)=LSMean(j) / Pr > |t|

Dependent Variable: SR

i/j	23	24	25	26	27	28	29	30	31	32	33
**1**	0<br>1.0000	0<br>1.0000	0<br>1.0000	0<br>1.0000	0<br>1.0000	0<br>1.0000	0<br>1.0000	0<br>1.0000	0<br>1.0000	0<br>1.0000	0<br>1.0000
**2**	0<br>1.0000	0<br>1.0000	0<br>1.0000	0<br>1.0000	0<br>1.0000	0<br>1.0000	0<br>1.0000	0<br>1.0000	0<br>1.0000	0<br>1.0000	0<br>1.0000
**3**	0<br>1.0000	0<br>1.0000	0<br>1.0000	0<br>1.0000	0<br>1.0000	0<br>1.0000	0<br>1.0000	0<br>1.0000	0<br>1.0000	0<br>1.0000	0<br>1.0000
**4**	0<br>1.0000	0<br>1.0000	0<br>1.0000	0<br>1.0000	0<br>1.0000	0<br>1.0000	0<br>1.0000	0<br>1.0000	0<br>1.0000	0<br>1.0000	0<br>1.0000
**5**	0<br>1.0000	0<br>1.0000	0<br>1.0000	0<br>1.0000	0<br>1.0000	0<br>1.0000	0<br>1.0000	0<br>1.0000	0<br>1.0000	0<br>1.0000	0<br>1.0000
**6**	-1.02494<br>0.3068	-1.02494<br>0.3068	-1.02494<br>0.3068	-1.02494<br>0.3068	-1.02494<br>0.3068	-1.02494<br>0.3068	-1.02494<br>0.3068	-1.02494<br>0.3068	-1.02494<br>0.3068	-1.02494<br>0.3068	-1.02494<br>0.3068
**7**	0<br>1.0000	0<br>1.0000	0<br>1.0000	0<br>1.0000	0<br>1.0000	0<br>1.0000	0<br>1.0000	0<br>1.0000	0<br>1.0000	0<br>1.0000	0<br>1.0000
**8**	0<br>1.0000	0<br>1.0000	0<br>1.0000	0<br>1.0000	0<br>1.0000	0<br>1.0000	0<br>1.0000	0<br>1.0000	0<br>1.0000	0<br>1.0000	0<br>1.0000
**9**	0<br>1.0000	0<br>1.0000	0<br>1.0000	0<br>1.0000	0<br>1.0000	0<br>1.0000	0<br>1.0000	0<br>1.0000	0<br>1.0000	0<br>1.0000	0<br>1.0000
**10**	-2.04987<br>0.0419	-2.04987<br>0.0419	-2.04987<br>0.0419	-2.04987<br>0.0419	-2.04987<br>0.0419	-2.04987<br>0.0419	-2.04987<br>0.0419	-2.04987<br>0.0419	-2.04987<br>0.0419	-2.04987<br>0.0419	-2.04987<br>0.0419

The SAS System

The GLM Procedure
Least Squares Means

Least Squares Means for Effect Day*Device
t for H0: LSMean(i)=LSMean(j) / Pr > |t|

Dependent Variable: SR

i/j	23	24	25	26	27	28	29	30	31	32	33
**11**	-5.12469<br><.0001	-5.12469<br><.0001	-5.12469<br><.0001	-5.12469<br><.0001	-5.12469<br><.0001	-5.12469<br><.0001	-5.12469<br><.0001	-5.12469<br><.0001	-5.12469<br><.0001	-5.12469<br><.0001	-5.12469<br><.0001
**12**	-4.13081<br><.0001	-4.13081<br><.0001	-4.13081<br><.0001	-4.13081<br><.0001	-4.13081<br><.0001	-4.13081<br><.0001	-4.13081<br><.0001	-4.13081<br><.0001	-4.13081<br><.0001	-4.13081<br><.0001	-4.13081<br><.0001
**13**	-1.02494<br>0.3068	-1.02494<br>0.3068	-1.02494<br>0.3068	-1.02494<br>0.3068	-1.02494<br>0.3068	-1.02494<br>0.3068	-1.02494<br>0.3068	-1.02494<br>0.3068	-1.02494<br>0.3068	-1.02494<br>0.3068	-1.02494<br>0.3068
**14**	-4.16251<br><.0001	-4.16251<br><.0001	-4.16251<br><.0001	-4.16251<br><.0001	-4.16251<br><.0001	-4.16251<br><.0001	-4.16251<br><.0001	-4.16251<br><.0001	-4.16251<br><.0001	-4.16251<br><.0001	-4.16251<br><.0001
**15**	-3.13778<br>0.0020	-3.13778<br>0.0020	-3.13778<br>0.0020	-3.13778<br>0.0020	-3.13778<br>0.0020	-3.13778<br>0.0020	-3.13778<br>0.0020	-3.13778<br>0.0020	-3.13778<br>0.0020	-3.13778<br>0.0020	-3.13778<br>0.0020
**16**	0<br>1.0000	0<br>1.0000	0<br>1.0000	0<br>1.0000	0<br>1.0000	0<br>1.0000	0<br>1.0000	0<br>1.0000	0<br>1.0000	0<br>1.0000	0<br>1.0000
**17**	0<br>1.0000	0<br>1.0000	0<br>1.0000	0<br>1.0000	0<br>1.0000	0<br>1.0000	0<br>1.0000	0<br>1.0000	0<br>1.0000	0<br>1.0000	0<br>1.0000
**18**	-1.04585<br>0.2971	-1.04585<br>0.2971	-1.04585<br>0.2971	-1.04585<br>0.2971	-1.04585<br>0.2971	-1.04585<br>0.2971	-1.04585<br>0.2971	-1.04585<br>0.2971	-1.04585<br>0.2971	-1.04585<br>0.2971	-1.04585<br>0.2971
**19**	0<br>1.0000	0<br>1.0000	0<br>1.0000	0<br>1.0000	0<br>1.0000	0<br>1.0000	0<br>1.0000	0<br>1.0000	0<br>1.0000	0<br>1.0000	0<br>1.0000
**20**	-2.1135<br>0.0360	-2.1135<br>0.0360	-2.1135<br>0.0360	-2.1135<br>0.0360	-2.1135<br>0.0360	-2.1135<br>0.0360	-2.1135<br>0.0360	-2.1135<br>0.0360	-2.1135<br>0.0360	-2.1135<br>0.0360	-2.1135<br>0.0360
**21**	0<br>1.0000	0<br>1.0000	0<br>1.0000	0<br>1.0000	0<br>1.0000	0<br>1.0000	0<br>1.0000	0<br>1.0000	0<br>1.0000	0<br>1.0000	0<br>1.0000
**22**	0<br>1.0000	0<br>1.0000	0<br>1.0000	0<br>1.0000	0<br>1.0000	0<br>1.0000	0<br>1.0000	0<br>1.0000	0<br>1.0000	0<br>1.0000	0<br>1.0000
**23**		0<br>1.0000	0<br>1.0000	0<br>1.0000	0<br>1.0000	0<br>1.0000	0<br>1.0000	0<br>1.0000	0<br>1.0000	0<br>1.0000	0<br>1.0000
**24**	0<br>1.0000		0<br>1.0000	0<br>1.0000	0<br>1.0000	0<br>1.0000	0<br>1.0000	0<br>1.0000	0<br>1.0000	0<br>1.0000	0<br>1.0000
**25**	0<br>1.0000	0<br>1.0000		0<br>1.0000	0<br>1.0000	0<br>1.0000	0<br>1.0000	0<br>1.0000	0<br>1.0000	0<br>1.0000	0<br>1.0000
**26**	0<br>1.0000	0<br>1.0000	0<br>1.0000		0<br>1.0000	0<br>1.0000	0<br>1.0000	0<br>1.0000	0<br>1.0000	0<br>1.0000	0<br>1.0000
**27**	0<br>1.0000	0<br>1.0000	0<br>1.0000	0<br>1.0000		0<br>1.0000	0<br>1.0000	0<br>1.0000	0<br>1.0000	0<br>1.0000	0<br>1.0000
**28**	0<br>1.0000	0<br>1.0000	0<br>1.0000	0<br>1.0000	0<br>1.0000		0<br>1.0000	0<br>1.0000	0<br>1.0000	0<br>1.0000	0<br>1.0000
**29**	0<br>1.0000	0<br>1.0000	0<br>1.0000	0<br>1.0000	0<br>1.0000	0<br>1.0000		0<br>1.0000	0<br>1.0000	0<br>1.0000	0<br>1.0000

The SAS System

The GLM Procedure
Least Squares Means

Least Squares Means for Effect Day*Device<br>t for H0: LSMean(i)=LSMean(j) / Pr > |t| <br>Dependent Variable: SR											
i/j	23	24	25	26	27	28	29	30	31	32	33
30	0<br>1.0000	0<br>1.0000	0<br>1.0000	0<br>1.0000	0<br>1.0000	0<br>1.0000	0<br>1.0000		0<br>1.0000	0<br>1.0000	0<br>1.0000
31	0<br>1.0000	0<br>1.0000	0<br>1.0000	0<br>1.0000	0<br>1.0000	0<br>1.0000	0<br>1.0000	0<br>1.0000		0<br>1.0000	0<br>1.0000
32	0<br>1.0000	0<br>1.0000	0<br>1.0000	0<br>1.0000	0<br>1.0000	0<br>1.0000	0<br>1.0000	0<br>1.0000	0<br>1.0000		0<br>1.0000
33	0<br>1.0000	0<br>1.0000	0<br>1.0000	0<br>1.0000	0<br>1.0000	0<br>1.0000	0<br>1.0000	0<br>1.0000	0<br>1.0000	0<br>1.0000	
34	0<br>1.0000	0<br>1.0000	0<br>1.0000	0<br>1.0000	0<br>1.0000	0<br>1.0000	0<br>1.0000	0<br>1.0000	0<br>1.0000	0<br>1.0000	0<br>1.0000
35	0<br>1.0000	0<br>1.0000	0<br>1.0000	0<br>1.0000	0<br>1.0000	0<br>1.0000	0<br>1.0000	0<br>1.0000	0<br>1.0000	0<br>1.0000	0<br>1.0000
36	-2.11327<br>0.0360	-2.11327<br>0.0360	-2.11327<br>0.0360	-2.11327<br>0.0360	-2.11327<br>0.0360	-2.11327<br>0.0360	-2.11327<br>0.0360	-2.11327<br>0.0360	-2.11327<br>0.0360	-2.11327<br>0.0360	-2.11327<br>0.0360
37	0<br>1.0000	0<br>1.0000	0<br>1.0000	0<br>1.0000	0<br>1.0000	0<br>1.0000	0<br>1.0000	0<br>1.0000	0<br>1.0000	0<br>1.0000	0<br>1.0000
38	0<br>1.0000	0<br>1.0000	0<br>1.0000	0<br>1.0000	0<br>1.0000	0<br>1.0000	0<br>1.0000	0<br>1.0000	0<br>1.0000	0<br>1.0000	0<br>1.0000
39	-2.13529<br>0.0341	-2.13529<br>0.0341	-2.13529<br>0.0341	-2.13529<br>0.0341	-2.13529<br>0.0341	-2.13529<br>0.0341	-2.13529<br>0.0341	-2.13529<br>0.0341	-2.13529<br>0.0341	-2.13529<br>0.0341	-2.13529<br>0.0341
40	-1.02494<br>0.3068	-1.02494<br>0.3068	-1.02494<br>0.3068	-1.02494<br>0.3068	-1.02494<br>0.3068	-1.02494<br>0.3068	-1.02494<br>0.3068	-1.02494<br>0.3068	-1.02494<br>0.3068	-1.02494<br>0.3068	-1.02494<br>0.3068
41	-1.05664<br>0.2921	-1.05664<br>0.2921	-1.05664<br>0.2921	-1.05664<br>0.2921	-1.05664<br>0.2921	-1.05664<br>0.2921	-1.05664<br>0.2921	-1.05664<br>0.2921	-1.05664<br>0.2921	-1.05664<br>0.2921	-1.05664<br>0.2921
42	0<br>1.0000	0<br>1.0000	0<br>1.0000	0<br>1.0000	0<br>1.0000	0<br>1.0000	0<br>1.0000	0<br>1.0000	0<br>1.0000	0<br>1.0000	0<br>1.0000
43	0<br>1.0000	0<br>1.0000	0<br>1.0000	0<br>1.0000	0<br>1.0000	0<br>1.0000	0<br>1.0000	0<br>1.0000	0<br>1.0000	0<br>1.0000	0<br>1.0000
44	0<br>1.0000	0<br>1.0000	0<br>1.0000	0<br>1.0000	0<br>1.0000	0<br>1.0000	0<br>1.0000	0<br>1.0000	0<br>1.0000	0<br>1.0000	0<br>1.0000
45	-1.05664<br>0.2921	-1.05664<br>0.2921	-1.05664<br>0.2921	-1.05664<br>0.2921	-1.05664<br>0.2921	-1.05664<br>0.2921	-1.05664<br>0.2921	-1.05664<br>0.2921	-1.05664<br>0.2921	-1.05664<br>0.2921	-1.05664<br>0.2921
46	0<br>1.0000	0<br>1.0000	0<br>1.0000	0<br>1.0000	0<br>1.0000	0<br>1.0000	0<br>1.0000	0<br>1.0000	0<br>1.0000	0<br>1.0000	0<br>1.0000
47	0<br>1.0000	0<br>1.0000	0<br>1.0000	0<br>1.0000	0<br>1.0000	0<br>1.0000	0<br>1.0000	0<br>1.0000	0<br>1.0000	0<br>1.0000	0<br>1.0000
48	0<br>1.0000	0<br>1.0000	0<br>1.0000	0<br>1.0000	0<br>1.0000	0<br>1.0000	0<br>1.0000	0<br>1.0000	0<br>1.0000	0<br>1.0000	0<br>1.0000

The SAS System

The GLM Procedure
Least Squares Means

Least Squares Means for Effect Day*Device
t for H0: LSMean(i)=LSMean(j) / Pr > |t|

Dependent Variable: SR

i/j	23	24	25	26	27	28	29	30	31	32	33
49	0<br>1.0000	0<br>1.0000	0<br>1.0000	0<br>1.0000	0<br>1.0000	0<br>1.0000	0<br>1.0000	0<br>1.0000	0<br>1.0000	0<br>1.0000	0<br>1.0000
50	0<br>1.0000	0<br>1.0000	0<br>1.0000	0<br>1.0000	0<br>1.0000	0<br>1.0000	0<br>1.0000	0<br>1.0000	0<br>1.0000	0<br>1.0000	0<br>1.0000
51	0<br>1.0000	0<br>1.0000	0<br>1.0000	0<br>1.0000	0<br>1.0000	0<br>1.0000	0<br>1.0000	0<br>1.0000	0<br>1.0000	0<br>1.0000	0<br>1.0000
52	0<br>1.0000	0<br>1.0000	0<br>1.0000	0<br>1.0000	0<br>1.0000	0<br>1.0000	0<br>1.0000	0<br>1.0000	0<br>1.0000	0<br>1.0000	0<br>1.0000
53	0<br>1.0000	0<br>1.0000	0<br>1.0000	0<br>1.0000	0<br>1.0000	0<br>1.0000	0<br>1.0000	0<br>1.0000	0<br>1.0000	0<br>1.0000	0<br>1.0000
54	-1.07888<br>0.2821	-1.07888<br>0.2821	-1.07888<br>0.2821	-1.07888<br>0.2821	-1.07888<br>0.2821	-1.07888<br>0.2821	-1.07888<br>0.2821	-1.07888<br>0.2821	-1.07888<br>0.2821	-1.07888<br>0.2821	-1.07888<br>0.2821
55	0<br>1.0000	0<br>1.0000	0<br>1.0000	0<br>1.0000	0<br>1.0000	0<br>1.0000	0<br>1.0000	0<br>1.0000	0<br>1.0000	0<br>1.0000	0<br>1.0000
56	0<br>1.0000	0<br>1.0000	0<br>1.0000	0<br>1.0000	0<br>1.0000	0<br>1.0000	0<br>1.0000	0<br>1.0000	0<br>1.0000	0<br>1.0000	0<br>1.0000
57	0<br>1.0000	0<br>1.0000	0<br>1.0000	0<br>1.0000	0<br>1.0000	0<br>1.0000	0<br>1.0000	0<br>1.0000	0<br>1.0000	0<br>1.0000	0<br>1.0000
58	0<br>1.0000	0<br>1.0000	0<br>1.0000	0<br>1.0000	0<br>1.0000	0<br>1.0000	0<br>1.0000	0<br>1.0000	0<br>1.0000	0<br>1.0000	0<br>1.0000
59	0<br>1.0000	0<br>1.0000	0<br>1.0000	0<br>1.0000	0<br>1.0000	0<br>1.0000	0<br>1.0000	0<br>1.0000	0<br>1.0000	0<br>1.0000	0<br>1.0000
60	0<br>1.0000	0<br>1.0000	0<br>1.0000	0<br>1.0000	0<br>1.0000	0<br>1.0000	0<br>1.0000	0<br>1.0000	0<br>1.0000	0<br>1.0000	0<br>1.0000
61	0<br>1.0000	0<br>1.0000	0<br>1.0000	0<br>1.0000	0<br>1.0000	0<br>1.0000	0<br>1.0000	0<br>1.0000	0<br>1.0000	0<br>1.0000	0<br>1.0000
62	0<br>1.0000	0<br>1.0000	0<br>1.0000	0<br>1.0000	0<br>1.0000	0<br>1.0000	0<br>1.0000	0<br>1.0000	0<br>1.0000	0<br>1.0000	0<br>1.0000
63	0<br>1.0000	0<br>1.0000	0<br>1.0000	0<br>1.0000	0<br>1.0000	0<br>1.0000	0<br>1.0000	0<br>1.0000	0<br>1.0000	0<br>1.0000	0<br>1.0000
64	0<br>1.0000	0<br>1.0000	0<br>1.0000	0<br>1.0000	0<br>1.0000	0<br>1.0000	0<br>1.0000	0<br>1.0000	0<br>1.0000	0<br>1.0000	0<br>1.0000
65	0<br>1.0000	0<br>1.0000	0<br>1.0000	0<br>1.0000	0<br>1.0000	0<br>1.0000	0<br>1.0000	0<br>1.0000	0<br>1.0000	0<br>1.0000	0<br>1.0000
66	0<br>1.0000	0<br>1.0000	0<br>1.0000	0<br>1.0000	0<br>1.0000	0<br>1.0000	0<br>1.0000	0<br>1.0000	0<br>1.0000	0<br>1.0000	0<br>1.0000
67	-1.03529<br>0.3020	-1.03529<br>0.3020	-1.03529<br>0.3020	-1.03529<br>0.3020	-1.03529<br>0.3020	-1.03529<br>0.3020	-1.03529<br>0.3020	-1.03529<br>0.3020	-1.03529<br>0.3020	-1.03529<br>0.3020	-1.03529<br>0.3020

The SAS System

The GLM Procedure
Least Squares Means

Least Squares Means for Effect Day*Device<br>t for H0: LSMean(i)=LSMean(j) / Pr > |t| <br>Dependent Variable: SR											
i/j	23	24	25	26	27	28	29	30	31	32	33
68	-1.05664<br>0.2921	-1.05664<br>0.2921	-1.05664<br>0.2921	-1.05664<br>0.2921	-1.05664<br>0.2921	-1.05664<br>0.2921	-1.05664<br>0.2921	-1.05664<br>0.2921	-1.05664<br>0.2921	-1.05664<br>0.2921	-1.05664<br>0.2921
69	-1.06764<br>0.2872	-1.06764<br>0.2872	-1.06764<br>0.2872	-1.06764<br>0.2872	-1.06764<br>0.2872	-1.06764<br>0.2872	-1.06764<br>0.2872	-1.06764<br>0.2872	-1.06764<br>0.2872	-1.06764<br>0.2872	-1.06764<br>0.2872
70	0<br>1.0000	0<br>1.0000	0<br>1.0000	0<br>1.0000	0<br>1.0000	0<br>1.0000	0<br>1.0000	0<br>1.0000	0<br>1.0000	0<br>1.0000	0<br>1.0000
71	0<br>1.0000	0<br>1.0000	0<br>1.0000	0<br>1.0000	0<br>1.0000	0<br>1.0000	0<br>1.0000	0<br>1.0000	0<br>1.0000	0<br>1.0000	0<br>1.0000
72	0<br>1.0000	0<br>1.0000	0<br>1.0000	0<br>1.0000	0<br>1.0000	0<br>1.0000	0<br>1.0000	0<br>1.0000	0<br>1.0000	0<br>1.0000	0<br>1.0000
73	0<br>1.0000	0<br>1.0000	0<br>1.0000	0<br>1.0000	0<br>1.0000	0<br>1.0000	0<br>1.0000	0<br>1.0000	0<br>1.0000	0<br>1.0000	0<br>1.0000
74	0<br>1.0000	0<br>1.0000	0<br>1.0000	0<br>1.0000	0<br>1.0000	0<br>1.0000	0<br>1.0000	0<br>1.0000	0<br>1.0000	0<br>1.0000	0<br>1.0000
75	-2.16924<br>0.0314	-2.16924<br>0.0314	-2.16924<br>0.0314	-2.16924<br>0.0314	-2.16924<br>0.0314	-2.16924<br>0.0314	-2.16924<br>0.0314	-2.16924<br>0.0314	-2.16924<br>0.0314	-2.16924<br>0.0314	-2.16924<br>0.0314
76	-1.03529<br>0.3020	-1.03529<br>0.3020	-1.03529<br>0.3020	-1.03529<br>0.3020	-1.03529<br>0.3020	-1.03529<br>0.3020	-1.03529<br>0.3020	-1.03529<br>0.3020	-1.03529<br>0.3020	-1.03529<br>0.3020	-1.03529<br>0.3020
77	0<br>1.0000	0<br>1.0000	0<br>1.0000	0<br>1.0000	0<br>1.0000	0<br>1.0000	0<br>1.0000	0<br>1.0000	0<br>1.0000	0<br>1.0000	0<br>1.0000
78	-1.09036<br>0.2771	-1.09036<br>0.2771	-1.09036<br>0.2771	-1.09036<br>0.2771	-1.09036<br>0.2771	-1.09036<br>0.2771	-1.09036<br>0.2771	-1.09036<br>0.2771	-1.09036<br>0.2771	-1.09036<br>0.2771	-1.09036<br>0.2771
79	0<br>1.0000	0<br>1.0000	0<br>1.0000	0<br>1.0000	0<br>1.0000	0<br>1.0000	0<br>1.0000	0<br>1.0000	0<br>1.0000	0<br>1.0000	0<br>1.0000
80	0<br>1.0000	0<br>1.0000	0<br>1.0000	0<br>1.0000	0<br>1.0000	0<br>1.0000	0<br>1.0000	0<br>1.0000	0<br>1.0000	0<br>1.0000	0<br>1.0000
81	0<br>1.0000	0<br>1.0000	0<br>1.0000	0<br>1.0000	0<br>1.0000	0<br>1.0000	0<br>1.0000	0<br>1.0000	0<br>1.0000	0<br>1.0000	0<br>1.0000
82	0<br>1.0000	0<br>1.0000	0<br>1.0000	0<br>1.0000	0<br>1.0000	0<br>1.0000	0<br>1.0000	0<br>1.0000	0<br>1.0000	0<br>1.0000	0<br>1.0000
83	-1.06764<br>0.2872	-1.06764<br>0.2872	-1.06764<br>0.2872	-1.06764<br>0.2872	-1.06764<br>0.2872	-1.06764<br>0.2872	-1.06764<br>0.2872	-1.06764<br>0.2872	-1.06764<br>0.2872	-1.06764<br>0.2872	-1.06764<br>0.2872
84	-1.10208<br>0.2719	-1.10208<br>0.2719	-1.10208<br>0.2719	-1.10208<br>0.2719	-1.10208<br>0.2719	-1.10208<br>0.2719	-1.10208<br>0.2719	-1.10208<br>0.2719	-1.10208<br>0.2719	-1.10208<br>0.2719	-1.10208<br>0.2719
85	-1.04585<br>0.2971	-1.04585<br>0.2971	-1.04585<br>0.2971	-1.04585<br>0.2971	-1.04585<br>0.2971	-1.04585<br>0.2971	-1.04585<br>0.2971	-1.04585<br>0.2971	-1.04585<br>0.2971	-1.04585<br>0.2971	-1.04585<br>0.2971

The SAS System

The GLM Procedure
Least Squares Means

Least Squares Means for Effect Day*Device
t for H0: LSMean(i)=LSMean(j) / Pr > |t|

Dependent Variable: SR

i/j	23	24	25	26	27	28	29	30	31	32	33
86	0<br>1.0000	0<br>1.0000	0<br>1.0000	0<br>1.0000	0<br>1.0000	0<br>1.0000	0<br>1.0000	0<br>1.0000	0<br>1.0000	0<br>1.0000	0<br>1.0000
87	-2.22812<br>0.0272	-2.22812<br>0.0272	-2.22812<br>0.0272	-2.22812<br>0.0272	-2.22812<br>0.0272	-2.22812<br>0.0272	-2.22812<br>0.0272	-2.22812<br>0.0272	-2.22812<br>0.0272	-2.22812<br>0.0272	-2.22812<br>0.0272

Least Squares Means for Effect Day*Device
t for H0: LSMean(i)=LSMean(j) / Pr > |t|

Dependent Variable: SR

i/j	34	35	36	37	38	39	40	41	42	43	44
1	0<br>1.0000	0<br>1.0000	2.113273<br>0.0360	0<br>1.0000	0<br>1.0000	2.135286<br>0.0341	1.024937<br>0.3068	1.056636<br>0.2921	0<br>1.0000	0<br>1.0000	0<br>1.0000
2	0<br>1.0000	0<br>1.0000	2.113273<br>0.0360	0<br>1.0000	0<br>1.0000	2.135286<br>0.0341	1.024937<br>0.3068	1.056636<br>0.2921	0<br>1.0000	0<br>1.0000	0<br>1.0000
3	0<br>1.0000	0<br>1.0000	2.113273<br>0.0360	0<br>1.0000	0<br>1.0000	2.135286<br>0.0341	1.024937<br>0.3068	1.056636<br>0.2921	0<br>1.0000	0<br>1.0000	0<br>1.0000
4	0<br>1.0000	0<br>1.0000	2.113273<br>0.0360	0<br>1.0000	0<br>1.0000	2.135286<br>0.0341	1.024937<br>0.3068	1.056636<br>0.2921	0<br>1.0000	0<br>1.0000	0<br>1.0000
5	0<br>1.0000	0<br>1.0000	2.113273<br>0.0360	0<br>1.0000	0<br>1.0000	2.135286<br>0.0341	1.024937<br>0.3068	1.056636<br>0.2921	0<br>1.0000	0<br>1.0000	0<br>1.0000
6	-1.02494<br>0.3068	-1.02494<br>0.3068	1.088335<br>0.2780	-1.02494<br>0.3068	-1.02494<br>0.3068	1.110349<br>0.2684	0<br>1.0000	0.031699<br>0.9747	-1.02494<br>0.3068	-1.02494<br>0.3068	-1.02494<br>0.3068
7	0<br>1.0000	0<br>1.0000	2.113273<br>0.0360	0<br>1.0000	0<br>1.0000	2.135286<br>0.0341	1.024937<br>0.3068	1.056636<br>0.2921	0<br>1.0000	0<br>1.0000	0<br>1.0000
8	0<br>1.0000	0<br>1.0000	2.113273<br>0.0360	0<br>1.0000	0<br>1.0000	2.135286<br>0.0341	1.024937<br>0.3068	1.056636<br>0.2921	0<br>1.0000	0<br>1.0000	0<br>1.0000
9	0<br>1.0000	0<br>1.0000	2.113273<br>0.0360	0<br>1.0000	0<br>1.0000	2.135286<br>0.0341	1.024937<br>0.3068	1.056636<br>0.2921	0<br>1.0000	0<br>1.0000	0<br>1.0000
10	-2.04987<br>0.0419	-2.04987<br>0.0419	0.063398<br>0.9495	-2.04987<br>0.0419	-2.04987<br>0.0419	0.085411<br>0.9320	-1.02494<br>0.3068	-0.99324<br>0.3220	-2.04987<br>0.0419	-2.04987<br>0.0419	-2.04987<br>0.0419
11	-5.12469<br><.0001	-5.12469<br><.0001	-3.01141<br>0.0030	-5.12469<br><.0001	-5.12469<br><.0001	-2.9894<br>0.0032	-4.09975<br><.0001	-4.06805<br><.0001	-5.12469<br><.0001	-5.12469<br><.0001	-5.12469<br><.0001
12	-4.13081<br><.0001	-4.13081<br><.0001	-2.01753<br>0.0452	-4.13081<br><.0001	-4.13081<br><.0001	-1.99552<br>0.0475	-3.10587<br>0.0022	-3.07417<br>0.0025	-4.13081<br><.0001	-4.13081<br><.0001	-4.13081<br><.0001
13	-1.02494<br>0.3068	-1.02494<br>0.3068	1.088335<br>0.2780	-1.02494<br>0.3068	-1.02494<br>0.3068	1.110349<br>0.2684	0<br>1.0000	0.031699<br>0.9747	-1.02494<br>0.3068	-1.02494<br>0.3068	-1.02494<br>0.3068
14	-4.16251<br><.0001	-4.16251<br><.0001	-2.04923<br>0.0419	-4.16251<br><.0001	-4.16251<br><.0001	-2.02722<br>0.0442	-3.13757<br>0.0020	-3.10587<br>0.0022	-4.16251<br><.0001	-4.16251<br><.0001	-4.16251<br><.0001

The SAS System

The GLM Procedure
Least Squares Means

Least Squares Means for Effect Day*Device
t for H0: LSMean(i)=LSMean(j) / Pr > |t|

Dependent Variable: SR

i/j	34	35	36	37	38	39	40	41	42	43	44
15	-3.13778<br>0.0020	-3.13778<br>0.0020	-1.02451<br>0.3070	-3.13778<br>0.0020	-3.13778<br>0.0020	-1.00249<br>0.3175	-2.11284<br>0.0360	-2.08114<br>0.0389	-3.13778<br>0.0020	-3.13778<br>0.0020	-3.13778<br>0.0020
16	0<br>1.0000	0<br>1.0000	2.113273<br>0.0360	0<br>1.0000	0<br>1.0000	2.135286<br>0.0341	1.024937<br>0.3068	1.056636<br>0.2921	0<br>1.0000	0<br>1.0000	0<br>1.0000
17	0<br>1.0000	0<br>1.0000	2.113273<br>0.0360	0<br>1.0000	0<br>1.0000	2.135286<br>0.0341	1.024937<br>0.3068	1.056636<br>0.2921	0<br>1.0000	0<br>1.0000	0<br>1.0000
18	-1.04585<br>0.2971	-1.04585<br>0.2971	1.067418<br>0.2873	-1.04585<br>0.2971	-1.04585<br>0.2971	1.089432<br>0.2775	-0.02092<br>0.9833	0.010782<br>0.9914	-1.04585<br>0.2971	-1.04585<br>0.2971	-1.04585<br>0.2971
19	0<br>1.0000	0<br>1.0000	2.113273<br>0.0360	0<br>1.0000	0<br>1.0000	2.135286<br>0.0341	1.024937<br>0.3068	1.056636<br>0.2921	0<br>1.0000	0<br>1.0000	0<br>1.0000
20	-2.1135<br>0.0360	-2.1135<br>0.0360	-0.00022<br>0.9998	-2.1135<br>0.0360	-2.1135<br>0.0360	0.021789<br>0.9826	-1.08856<br>0.2779	-1.05686<br>0.2920	-2.1135<br>0.0360	-2.1135<br>0.0360	-2.1135<br>0.0360
21	0<br>1.0000	0<br>1.0000	2.113273<br>0.0360	0<br>1.0000	0<br>1.0000	2.135286<br>0.0341	1.024937<br>0.3068	1.056636<br>0.2921	0<br>1.0000	0<br>1.0000	0<br>1.0000
22	0<br>1.0000	0<br>1.0000	2.113273<br>0.0360	0<br>1.0000	0<br>1.0000	2.135286<br>0.0341	1.024937<br>0.3068	1.056636<br>0.2921	0<br>1.0000	0<br>1.0000	0<br>1.0000
23	0<br>1.0000	0<br>1.0000	2.113273<br>0.0360	0<br>1.0000	0<br>1.0000	2.135286<br>0.0341	1.024937<br>0.3068	1.056636<br>0.2921	0<br>1.0000	0<br>1.0000	0<br>1.0000
24	0<br>1.0000	0<br>1.0000	2.113273<br>0.0360	0<br>1.0000	0<br>1.0000	2.135286<br>0.0341	1.024937<br>0.3068	1.056636<br>0.2921	0<br>1.0000	0<br>1.0000	0<br>1.0000
25	0<br>1.0000	0<br>1.0000	2.113273<br>0.0360	0<br>1.0000	0<br>1.0000	2.135286<br>0.0341	1.024937<br>0.3068	1.056636<br>0.2921	0<br>1.0000	0<br>1.0000	0<br>1.0000
26	0<br>1.0000	0<br>1.0000	2.113273<br>0.0360	0<br>1.0000	0<br>1.0000	2.135286<br>0.0341	1.024937<br>0.3068	1.056636<br>0.2921	0<br>1.0000	0<br>1.0000	0<br>1.0000
27	0<br>1.0000	0<br>1.0000	2.113273<br>0.0360	0<br>1.0000	0<br>1.0000	2.135286<br>0.0341	1.024937<br>0.3068	1.056636<br>0.2921	0<br>1.0000	0<br>1.0000	0<br>1.0000
28	0<br>1.0000	0<br>1.0000	2.113273<br>0.0360	0<br>1.0000	0<br>1.0000	2.135286<br>0.0341	1.024937<br>0.3068	1.056636<br>0.2921	0<br>1.0000	0<br>1.0000	0<br>1.0000
29	0<br>1.0000	0<br>1.0000	2.113273<br>0.0360	0<br>1.0000	0<br>1.0000	2.135286<br>0.0341	1.024937<br>0.3068	1.056636<br>0.2921	0<br>1.0000	0<br>1.0000	0<br>1.0000
30	0<br>1.0000	0<br>1.0000	2.113273<br>0.0360	0<br>1.0000	0<br>1.0000	2.135286<br>0.0341	1.024937<br>0.3068	1.056636<br>0.2921	0<br>1.0000	0<br>1.0000	0<br>1.0000
31	0<br>1.0000	0<br>1.0000	2.113273<br>0.0360	0<br>1.0000	0<br>1.0000	2.135286<br>0.0341	1.024937<br>0.3068	1.056636<br>0.2921	0<br>1.0000	0<br>1.0000	0<br>1.0000
32	0<br>1.0000	0<br>1.0000	2.113273<br>0.0360	0<br>1.0000	0<br>1.0000	2.135286<br>0.0341	1.024937<br>0.3068	1.056636<br>0.2921	0<br>1.0000	0<br>1.0000	0<br>1.0000
33	0<br>1.0000	0<br>1.0000	2.113273<br>0.0360	0<br>1.0000	0<br>1.0000	2.135286<br>0.0341	1.024937<br>0.3068	1.056636<br>0.2921	0<br>1.0000	0<br>1.0000	0<br>1.0000

The SAS System

The GLM Procedure
Least Squares Means

Least Squares Means for Effect Day*Device
t for H0: LSMean(i)=LSMean(j) / Pr > |t|

Dependent Variable: SR

i/j	34	35	36	37	38	39	40	41	42	43	44
**34**		0<br>1.0000	2.113273<br>0.0360	0<br>1.0000	0<br>1.0000	2.135286<br>0.0341	1.024937<br>0.3068	1.056636<br>0.2921	0<br>1.0000	0<br>1.0000	0<br>1.0000
**35**	0<br>1.0000		2.113273<br>0.0360	0<br>1.0000	0<br>1.0000	2.135286<br>0.0341	1.024937<br>0.3068	1.056636<br>0.2921	0<br>1.0000	0<br>1.0000	0<br>1.0000
**36**	-2.11327<br>0.0360	-2.11327<br>0.0360		-2.11327<br>0.0360	-2.11327<br>0.0360	0.022013<br>0.9825	-1.08834<br>0.2780	-1.05664<br>0.2921	-2.11327<br>0.0360	-2.11327<br>0.0360	-2.11327<br>0.0360
**37**	0<br>1.0000	0<br>1.0000	2.113273<br>0.0360		0<br>1.0000	2.135286<br>0.0341	1.024937<br>0.3068	1.056636<br>0.2921	0<br>1.0000	0<br>1.0000	0<br>1.0000
**38**	0<br>1.0000	0<br>1.0000	2.113273<br>0.0360	0<br>1.0000		2.135286<br>0.0341	1.024937<br>0.3068	1.056636<br>0.2921	0<br>1.0000	0<br>1.0000	0<br>1.0000
**39**	-2.13529<br>0.0341	-2.13529<br>0.0341	-0.02201<br>0.9825	-2.13529<br>0.0341	-2.13529<br>0.0341		-1.11035<br>0.2684	-1.07865<br>0.2822	-2.13529<br>0.0341	-2.13529<br>0.0341	-2.13529<br>0.0341
**40**	-1.02494<br>0.3068	-1.02494<br>0.3068	1.088335<br>0.2780	-1.02494<br>0.3068	-1.02494<br>0.3068	1.110349<br>0.2684		0.031699<br>0.9747	-1.02494<br>0.3068	-1.02494<br>0.3068	-1.02494<br>0.3068
**41**	-1.05664<br>0.2921	-1.05664<br>0.2921	1.056636<br>0.2921	-1.05664<br>0.2921	-1.05664<br>0.2921	1.07865<br>0.2822	-0.0317<br>0.9747		-1.05664<br>0.2921	-1.05664<br>0.2921	-1.05664<br>0.2921
**42**	0<br>1.0000	0<br>1.0000	2.113273<br>0.0360	0<br>1.0000	0<br>1.0000	2.135286<br>0.0341	1.024937<br>0.3068	1.056636<br>0.2921		0<br>1.0000	0<br>1.0000
**43**	0<br>1.0000	0<br>1.0000	2.113273<br>0.0360	0<br>1.0000	0<br>1.0000	2.135286<br>0.0341	1.024937<br>0.3068	1.056636<br>0.2921	0<br>1.0000		0<br>1.0000
**44**	0<br>1.0000	0<br>1.0000	2.113273<br>0.0360	0<br>1.0000	0<br>1.0000	2.135286<br>0.0341	1.024937<br>0.3068	1.056636<br>0.2921	0<br>1.0000	0<br>1.0000	
**45**	-1.05664<br>0.2921	-1.05664<br>0.2921	1.056636<br>0.2921	-1.05664<br>0.2921	-1.05664<br>0.2921	1.07865<br>0.2822	-0.0317<br>0.9747	0<br>1.0000	-1.05664<br>0.2921	-1.05664<br>0.2921	-1.05664<br>0.2921
**46**	0<br>1.0000	0<br>1.0000	2.113273<br>0.0360	0<br>1.0000	0<br>1.0000	2.135286<br>0.0341	1.024937<br>0.3068	1.056636<br>0.2921	0<br>1.0000	0<br>1.0000	0<br>1.0000
**47**	0<br>1.0000	0<br>1.0000	2.113273<br>0.0360	0<br>1.0000	0<br>1.0000	2.135286<br>0.0341	1.024937<br>0.3068	1.056636<br>0.2921	0<br>1.0000	0<br>1.0000	0<br>1.0000
**48**	0<br>1.0000	0<br>1.0000	2.113273<br>0.0360	0<br>1.0000	0<br>1.0000	2.135286<br>0.0341	1.024937<br>0.3068	1.056636<br>0.2921	0<br>1.0000	0<br>1.0000	0<br>1.0000
**49**	0<br>1.0000	0<br>1.0000	2.113273<br>0.0360	0<br>1.0000	0<br>1.0000	2.135286<br>0.0341	1.024937<br>0.3068	1.056636<br>0.2921	0<br>1.0000	0<br>1.0000	0<br>1.0000
**50**	0<br>1.0000	0<br>1.0000	2.113273<br>0.0360	0<br>1.0000	0<br>1.0000	2.135286<br>0.0341	1.024937<br>0.3068	1.056636<br>0.2921	0<br>1.0000	0<br>1.0000	0<br>1.0000
**51**	0<br>1.0000	0<br>1.0000	2.113273<br>0.0360	0<br>1.0000	0<br>1.0000	2.135286<br>0.0341	1.024937<br>0.3068	1.056636<br>0.2921	0<br>1.0000	0<br>1.0000	0<br>1.0000
**52**	0<br>1.0000	0<br>1.0000	2.113273<br>0.0360	0<br>1.0000	0<br>1.0000	2.135286<br>0.0341	1.024937<br>0.3068	1.056636<br>0.2921	0<br>1.0000	0<br>1.0000	0<br>1.0000

The SAS System

The GLM Procedure
Least Squares Means

Least Squares Means for Effect Day*Device
t for H0: LSMean(i)=LSMean(j) / Pr > |t|

Dependent Variable: SR

i/j	34	35	36	37	38	39	40	41	42	43	44
53	0<br>1.0000	0<br>1.0000	2.113273<br>0.0360	0<br>1.0000	0<br>1.0000	2.135286<br>0.0341	1.024937<br>0.3068	1.056636<br>0.2921	0<br>1.0000	0<br>1.0000	0<br>1.0000
54	-1.07888<br>0.2821	-1.07888<br>0.2821	1.034391<br>0.3024	-1.07888<br>0.2821	-1.07888<br>0.2821	1.056405<br>0.2922	-0.05394<br>0.9570	-0.02224<br>0.9823	-1.07888<br>0.2821	-1.07888<br>0.2821	-1.07888<br>0.2821
55	0<br>1.0000	0<br>1.0000	2.113273<br>0.0360	0<br>1.0000	0<br>1.0000	2.135286<br>0.0341	1.024937<br>0.3068	1.056636<br>0.2921	0<br>1.0000	0<br>1.0000	0<br>1.0000
56	0<br>1.0000	0<br>1.0000	2.113273<br>0.0360	0<br>1.0000	0<br>1.0000	2.135286<br>0.0341	1.024937<br>0.3068	1.056636<br>0.2921	0<br>1.0000	0<br>1.0000	0<br>1.0000
57	0<br>1.0000	0<br>1.0000	2.113273<br>0.0360	0<br>1.0000	0<br>1.0000	2.135286<br>0.0341	1.024937<br>0.3068	1.056636<br>0.2921	0<br>1.0000	0<br>1.0000	0<br>1.0000
58	0<br>1.0000	0<br>1.0000	2.113273<br>0.0360	0<br>1.0000	0<br>1.0000	2.135286<br>0.0341	1.024937<br>0.3068	1.056636<br>0.2921	0<br>1.0000	0<br>1.0000	0<br>1.0000
59	0<br>1.0000	0<br>1.0000	2.113273<br>0.0360	0<br>1.0000	0<br>1.0000	2.135286<br>0.0341	1.024937<br>0.3068	1.056636<br>0.2921	0<br>1.0000	0<br>1.0000	0<br>1.0000
60	0<br>1.0000	0<br>1.0000	2.113273<br>0.0360	0<br>1.0000	0<br>1.0000	2.135286<br>0.0341	1.024937<br>0.3068	1.056636<br>0.2921	0<br>1.0000	0<br>1.0000	0<br>1.0000
61	0<br>1.0000	0<br>1.0000	2.113273<br>0.0360	0<br>1.0000	0<br>1.0000	2.135286<br>0.0341	1.024937<br>0.3068	1.056636<br>0.2921	0<br>1.0000	0<br>1.0000	0<br>1.0000
62	0<br>1.0000	0<br>1.0000	2.113273<br>0.0360	0<br>1.0000	0<br>1.0000	2.135286<br>0.0341	1.024937<br>0.3068	1.056636<br>0.2921	0<br>1.0000	0<br>1.0000	0<br>1.0000
63	0<br>1.0000	0<br>1.0000	2.113273<br>0.0360	0<br>1.0000	0<br>1.0000	2.135286<br>0.0341	1.024937<br>0.3068	1.056636<br>0.2921	0<br>1.0000	0<br>1.0000	0<br>1.0000
64	0<br>1.0000	0<br>1.0000	2.113273<br>0.0360	0<br>1.0000	0<br>1.0000	2.135286<br>0.0341	1.024937<br>0.3068	1.056636<br>0.2921	0<br>1.0000	0<br>1.0000	0<br>1.0000
65	0<br>1.0000	0<br>1.0000	2.113273<br>0.0360	0<br>1.0000	0<br>1.0000	2.135286<br>0.0341	1.024937<br>0.3068	1.056636<br>0.2921	0<br>1.0000	0<br>1.0000	0<br>1.0000
66	0<br>1.0000	0<br>1.0000	2.113273<br>0.0360	0<br>1.0000	0<br>1.0000	2.135286<br>0.0341	1.024937<br>0.3068	1.056636<br>0.2921	0<br>1.0000	0<br>1.0000	0<br>1.0000
67	-1.03529<br>0.3020	-1.03529<br>0.3020	1.077982<br>0.2825	-1.03529<br>0.3020	-1.03529<br>0.3020	1.099996<br>0.2729	-0.01035<br>0.9918	0.021346<br>0.9830	-1.03529<br>0.3020	-1.03529<br>0.3020	-1.03529<br>0.3020
68	-1.05664<br>0.2921	-1.05664<br>0.2921	1.056636<br>0.2921	-1.05664<br>0.2921	-1.05664<br>0.2921	1.07865<br>0.2822	-0.0317<br>0.9747	0<br>1.0000	-1.05664<br>0.2921	-1.05664<br>0.2921	-1.05664<br>0.2921
69	-1.06764<br>0.2872	-1.06764<br>0.2872	1.04563<br>0.2972	-1.06764<br>0.2872	-1.06764<br>0.2872	1.067643<br>0.2872	-0.04271<br>0.9660	-0.01101<br>0.9912	-1.06764<br>0.2872	-1.06764<br>0.2872	-1.06764<br>0.2872
70	0<br>1.0000	0<br>1.0000	2.113273<br>0.0360	0<br>1.0000	0<br>1.0000	2.135286<br>0.0341	1.024937<br>0.3068	1.056636<br>0.2921	0<br>1.0000	0<br>1.0000	0<br>1.0000
71	0<br>1.0000	0<br>1.0000	2.113273<br>0.0360	0<br>1.0000	0<br>1.0000	2.135286<br>0.0341	1.024937<br>0.3068	1.056636<br>0.2921	0<br>1.0000	0<br>1.0000	0<br>1.0000

The SAS System

The GLM Procedure
Least Squares Means

Least Squares Means for Effect Day*Device<br>t for H0: LSMean(i)=LSMean(j) / Pr > |t| <br>Dependent Variable: SR											
i/j	34	35	36	37	38	39	40	41	42	43	44
72	0<br>1.0000	0<br>1.0000	2.113273<br>0.0360	0<br>1.0000	0<br>1.0000	2.135286<br>0.0341	1.024937<br>0.3068	1.056636<br>0.2921	0<br>1.0000	0<br>1.0000	0<br>1.0000
73	0<br>1.0000	0<br>1.0000	2.113273<br>0.0360	0<br>1.0000	0<br>1.0000	2.135286<br>0.0341	1.024937<br>0.3068	1.056636<br>0.2921	0<br>1.0000	0<br>1.0000	0<br>1.0000
74	0<br>1.0000	0<br>1.0000	2.113273<br>0.0360	0<br>1.0000	0<br>1.0000	2.135286<br>0.0341	1.024937<br>0.3068	1.056636<br>0.2921	0<br>1.0000	0<br>1.0000	0<br>1.0000
75	-2.16924<br>0.0314	-2.16924<br>0.0314	-0.05597<br>0.9554	-2.16924<br>0.0314	-2.16924<br>0.0314	-0.03395<br>0.9730	-1.1443<br>0.2541	-1.1126<br>0.2674	-2.16924<br>0.0314	-2.16924<br>0.0314	-2.16924<br>0.0314
76	-1.03529<br>0.3020	-1.03529<br>0.3020	1.077982<br>0.2825	-1.03529<br>0.3020	-1.03529<br>0.3020	1.099996<br>0.2729	-0.01035<br>0.9918	0.021346<br>0.9830	-1.03529<br>0.3020	-1.03529<br>0.3020	-1.03529<br>0.3020
77	0<br>1.0000	0<br>1.0000	2.113273<br>0.0360	0<br>1.0000	0<br>1.0000	2.135286<br>0.0341	1.024937<br>0.3068	1.056636<br>0.2921	0<br>1.0000	0<br>1.0000	0<br>1.0000
78	-1.09036<br>0.2771	-1.09036<br>0.2771	1.022914<br>0.3078	-1.09036<br>0.2771	-1.09036<br>0.2771	1.044927<br>0.2975	-0.06542<br>0.9479	-0.03372<br>0.9731	-1.09036<br>0.2771	-1.09036<br>0.2771	-1.09036<br>0.2771
79	0<br>1.0000	0<br>1.0000	2.113273<br>0.0360	0<br>1.0000	0<br>1.0000	2.135286<br>0.0341	1.024937<br>0.3068	1.056636<br>0.2921	0<br>1.0000	0<br>1.0000	0<br>1.0000
80	0<br>1.0000	0<br>1.0000	2.113273<br>0.0360	0<br>1.0000	0<br>1.0000	2.135286<br>0.0341	1.024937<br>0.3068	1.056636<br>0.2921	0<br>1.0000	0<br>1.0000	0<br>1.0000
81	0<br>1.0000	0<br>1.0000	2.113273<br>0.0360	0<br>1.0000	0<br>1.0000	2.135286<br>0.0341	1.024937<br>0.3068	1.056636<br>0.2921	0<br>1.0000	0<br>1.0000	0<br>1.0000
82	0<br>1.0000	0<br>1.0000	2.113273<br>0.0360	0<br>1.0000	0<br>1.0000	2.135286<br>0.0341	1.024937<br>0.3068	1.056636<br>0.2921	0<br>1.0000	0<br>1.0000	0<br>1.0000
83	-1.06764<br>0.2872	-1.06764<br>0.2872	1.04563<br>0.2972	-1.06764<br>0.2872	-1.06764<br>0.2872	1.067643<br>0.2872	-0.04271<br>0.9660	-0.01101<br>0.9912	-1.06764<br>0.2872	-1.06764<br>0.2872	-1.06764<br>0.2872
84	-1.10208<br>0.2719	-1.10208<br>0.2719	1.01119<br>0.3133	-1.10208<br>0.2719	-1.10208<br>0.2719	1.033203<br>0.3029	-0.07715<br>0.9386	-0.04545<br>0.9638	-1.10208<br>0.2719	-1.10208<br>0.2719	-1.10208<br>0.2719
85	-1.04585<br>0.2971	-1.04585<br>0.2971	1.067418<br>0.2873	-1.04585<br>0.2971	-1.04585<br>0.2971	1.089432<br>0.2775	-0.02092<br>0.9833	0.010782<br>0.9914	-1.04585<br>0.2971	-1.04585<br>0.2971	-1.04585<br>0.2971
86	0<br>1.0000	0<br>1.0000	2.113273<br>0.0360	0<br>1.0000	0<br>1.0000	2.135286<br>0.0341	1.024937<br>0.3068	1.056636<br>0.2921	0<br>1.0000	0<br>1.0000	0<br>1.0000
87	-2.22812<br>0.0272	-2.22812<br>0.0272	-0.11485<br>0.9087	-2.22812<br>0.0272	-2.22812<br>0.0272	-0.09284<br>0.9261	-1.20319<br>0.2305	-1.17149<br>0.2430	-2.22812<br>0.0272	-2.22812<br>0.0272	-2.22812<br>0.0272

The SAS System

The GLM Procedure
Least Squares Means

Least Squares Means for Effect Day*Device
t for H0: LSMean(i)=LSMean(j) / Pr > |t|

Dependent Variable: SR

i/j	45	46	47	48	49	50	51	52	53	54	55
1	1.056636<br>0.2921	0<br>1.0000	0<br>1.0000	0<br>1.0000	0<br>1.0000	0<br>1.0000	0<br>1.0000	0<br>1.0000	0<br>1.0000	1.078881<br>0.2821	0<br>1.0000
2	1.056636<br>0.2921	0<br>1.0000	0<br>1.0000	0<br>1.0000	0<br>1.0000	0<br>1.0000	0<br>1.0000	0<br>1.0000	0<br>1.0000	1.078881<br>0.2821	0<br>1.0000
3	1.056636<br>0.2921	0<br>1.0000	0<br>1.0000	0<br>1.0000	0<br>1.0000	0<br>1.0000	0<br>1.0000	0<br>1.0000	0<br>1.0000	1.078881<br>0.2821	0<br>1.0000
4	1.056636<br>0.2921	0<br>1.0000	0<br>1.0000	0<br>1.0000	0<br>1.0000	0<br>1.0000	0<br>1.0000	0<br>1.0000	0<br>1.0000	1.078881<br>0.2821	0<br>1.0000
5	1.056636<br>0.2921	0<br>1.0000	0<br>1.0000	0<br>1.0000	0<br>1.0000	0<br>1.0000	0<br>1.0000	0<br>1.0000	0<br>1.0000	1.078881<br>0.2821	0<br>1.0000
6	0.031699<br>0.9747	-1.02494<br>0.3068	-1.02494<br>0.3068	-1.02494<br>0.3068	-1.02494<br>0.3068	-1.02494<br>0.3068	-1.02494<br>0.3068	-1.02494<br>0.3068	-1.02494<br>0.3068	0.053944<br>0.9570	-1.02494<br>0.3068
7	1.056636<br>0.2921	0<br>1.0000	0<br>1.0000	0<br>1.0000	0<br>1.0000	0<br>1.0000	0<br>1.0000	0<br>1.0000	0<br>1.0000	1.078881<br>0.2821	0<br>1.0000
8	1.056636<br>0.2921	0<br>1.0000	0<br>1.0000	0<br>1.0000	0<br>1.0000	0<br>1.0000	0<br>1.0000	0<br>1.0000	0<br>1.0000	1.078881<br>0.2821	0<br>1.0000
9	1.056636<br>0.2921	0<br>1.0000	0<br>1.0000	0<br>1.0000	0<br>1.0000	0<br>1.0000	0<br>1.0000	0<br>1.0000	0<br>1.0000	1.078881<br>0.2821	0<br>1.0000
10	-0.99324<br>0.3220	-2.04987<br>0.0419	-2.04987<br>0.0419	-2.04987<br>0.0419	-2.04987<br>0.0419	-2.04987<br>0.0419	-2.04987<br>0.0419	-2.04987<br>0.0419	-2.04987<br>0.0419	-0.97099<br>0.3329	-2.04987<br>0.0419
11	-4.06805<br><.0001	-5.12469<br><.0001	-5.12469<br><.0001	-5.12469<br><.0001	-5.12469<br><.0001	-5.12469<br><.0001	-5.12469<br><.0001	-5.12469<br><.0001	-5.12469<br><.0001	-4.0458<br><.0001	-5.12469<br><.0001
12	-3.07417<br>0.0025	-4.13081<br><.0001	-4.13081<br><.0001	-4.13081<br><.0001	-4.13081<br><.0001	-4.13081<br><.0001	-4.13081<br><.0001	-4.13081<br><.0001	-4.13081<br><.0001	-3.05193<br>0.0026	-4.13081<br><.0001
13	0.031699<br>0.9747	-1.02494<br>0.3068	-1.02494<br>0.3068	-1.02494<br>0.3068	-1.02494<br>0.3068	-1.02494<br>0.3068	-1.02494<br>0.3068	-1.02494<br>0.3068	-1.02494<br>0.3068	0.053944<br>0.9570	-1.02494<br>0.3068
14	-3.10587<br>0.0022	-4.16251<br><.0001	-4.16251<br><.0001	-4.16251<br><.0001	-4.16251<br><.0001	-4.16251<br><.0001	-4.16251<br><.0001	-4.16251<br><.0001	-4.16251<br><.0001	-3.08363<br>0.0024	-4.16251<br><.0001
15	-2.08114<br>0.0389	-3.13778<br>0.0020	-3.13778<br>0.0020	-3.13778<br>0.0020	-3.13778<br>0.0020	-3.13778<br>0.0020	-3.13778<br>0.0020	-3.13778<br>0.0020	-3.13778<br>0.0020	-2.0589<br>0.0410	-3.13778<br>0.0020
16	1.056636<br>0.2921	0<br>1.0000	0<br>1.0000	0<br>1.0000	0<br>1.0000	0<br>1.0000	0<br>1.0000	0<br>1.0000	0<br>1.0000	1.078881<br>0.2821	0<br>1.0000
17	1.056636<br>0.2921	0<br>1.0000	0<br>1.0000	0<br>1.0000	0<br>1.0000	0<br>1.0000	0<br>1.0000	0<br>1.0000	0<br>1.0000	1.078881<br>0.2821	0<br>1.0000
18	0.010782<br>0.9914	-1.04585<br>0.2971	-1.04585<br>0.2971	-1.04585<br>0.2971	-1.04585<br>0.2971	-1.04585<br>0.2971	-1.04585<br>0.2971	-1.04585<br>0.2971	-1.04585<br>0.2971	0.033027<br>0.9737	-1.04585<br>0.2971
19	1.056636<br>0.2921	0<br>1.0000	0<br>1.0000	0<br>1.0000	0<br>1.0000	0<br>1.0000	0<br>1.0000	0<br>1.0000	0<br>1.0000	1.078881<br>0.2821	0<br>1.0000

The SAS System

The GLM Procedure
Least Squares Means

Least Squares Means for Effect Day*Device<br>t for H0: LSMean(i)=LSMean(j) / Pr > |t| <br>Dependent Variable: SR											
**i/j**	**45**	**46**	**47**	**48**	**49**	**50**	**51**	**52**	**53**	**54**	**55**
**20**	-1.05686<br>0.2920	-2.1135<br>0.0360	-2.1135<br>0.0360	-2.1135<br>0.0360	-2.1135<br>0.0360	-2.1135<br>0.0360	-2.1135<br>0.0360	-2.1135<br>0.0360	-2.1135<br>0.0360	-1.03462<br>0.3023	-2.1135<br>0.0360
**21**	1.056636<br>0.2921	0<br>1.0000	0<br>1.0000	0<br>1.0000	0<br>1.0000	0<br>1.0000	0<br>1.0000	0<br>1.0000	0<br>1.0000	1.078881<br>0.2821	0<br>1.0000
**22**	1.056636<br>0.2921	0<br>1.0000	0<br>1.0000	0<br>1.0000	0<br>1.0000	0<br>1.0000	0<br>1.0000	0<br>1.0000	0<br>1.0000	1.078881<br>0.2821	0<br>1.0000
**23**	1.056636<br>0.2921	0<br>1.0000	0<br>1.0000	0<br>1.0000	0<br>1.0000	0<br>1.0000	0<br>1.0000	0<br>1.0000	0<br>1.0000	1.078881<br>0.2821	0<br>1.0000
**24**	1.056636<br>0.2921	0<br>1.0000	0<br>1.0000	0<br>1.0000	0<br>1.0000	0<br>1.0000	0<br>1.0000	0<br>1.0000	0<br>1.0000	1.078881<br>0.2821	0<br>1.0000
**25**	1.056636<br>0.2921	0<br>1.0000	0<br>1.0000	0<br>1.0000	0<br>1.0000	0<br>1.0000	0<br>1.0000	0<br>1.0000	0<br>1.0000	1.078881<br>0.2821	0<br>1.0000
**26**	1.056636<br>0.2921	0<br>1.0000	0<br>1.0000	0<br>1.0000	0<br>1.0000	0<br>1.0000	0<br>1.0000	0<br>1.0000	0<br>1.0000	1.078881<br>0.2821	0<br>1.0000
**27**	1.056636<br>0.2921	0<br>1.0000	0<br>1.0000	0<br>1.0000	0<br>1.0000	0<br>1.0000	0<br>1.0000	0<br>1.0000	0<br>1.0000	1.078881<br>0.2821	0<br>1.0000
**28**	1.056636<br>0.2921	0<br>1.0000	0<br>1.0000	0<br>1.0000	0<br>1.0000	0<br>1.0000	0<br>1.0000	0<br>1.0000	0<br>1.0000	1.078881<br>0.2821	0<br>1.0000
**29**	1.056636<br>0.2921	0<br>1.0000	0<br>1.0000	0<br>1.0000	0<br>1.0000	0<br>1.0000	0<br>1.0000	0<br>1.0000	0<br>1.0000	1.078881<br>0.2821	0<br>1.0000
**30**	1.056636<br>0.2921	0<br>1.0000	0<br>1.0000	0<br>1.0000	0<br>1.0000	0<br>1.0000	0<br>1.0000	0<br>1.0000	0<br>1.0000	1.078881<br>0.2821	0<br>1.0000
**31**	1.056636<br>0.2921	0<br>1.0000	0<br>1.0000	0<br>1.0000	0<br>1.0000	0<br>1.0000	0<br>1.0000	0<br>1.0000	0<br>1.0000	1.078881<br>0.2821	0<br>1.0000
**32**	1.056636<br>0.2921	0<br>1.0000	0<br>1.0000	0<br>1.0000	0<br>1.0000	0<br>1.0000	0<br>1.0000	0<br>1.0000	0<br>1.0000	1.078881<br>0.2821	0<br>1.0000
**33**	1.056636<br>0.2921	0<br>1.0000	0<br>1.0000	0<br>1.0000	0<br>1.0000	0<br>1.0000	0<br>1.0000	0<br>1.0000	0<br>1.0000	1.078881<br>0.2821	0<br>1.0000
**34**	1.056636<br>0.2921	0<br>1.0000	0<br>1.0000	0<br>1.0000	0<br>1.0000	0<br>1.0000	0<br>1.0000	0<br>1.0000	0<br>1.0000	1.078881<br>0.2821	0<br>1.0000
**35**	1.056636<br>0.2921	0<br>1.0000	0<br>1.0000	0<br>1.0000	0<br>1.0000	0<br>1.0000	0<br>1.0000	0<br>1.0000	0<br>1.0000	1.078881<br>0.2821	0<br>1.0000
**36**	-1.05664<br>0.2921	-2.11327<br>0.0360	-2.11327<br>0.0360	-2.11327<br>0.0360	-2.11327<br>0.0360	-2.11327<br>0.0360	-2.11327<br>0.0360	-2.11327<br>0.0360	-2.11327<br>0.0360	-1.03439<br>0.3024	-2.11327<br>0.0360
**37**	1.056636<br>0.2921	0<br>1.0000	0<br>1.0000	0<br>1.0000	0<br>1.0000	0<br>1.0000	0<br>1.0000	0<br>1.0000	0<br>1.0000	1.078881<br>0.2821	0<br>1.0000
**38**	1.056636<br>0.2921	0<br>1.0000	0<br>1.0000	0<br>1.0000	0<br>1.0000	0<br>1.0000	0<br>1.0000	0<br>1.0000	0<br>1.0000	1.078881<br>0.2821	0<br>1.0000

The SAS System

The GLM Procedure
Least Squares Means

Least Squares Means for Effect Day*Device<br>t for H0: LSMean(i)=LSMean(j) / Pr > |t| <br>Dependent Variable: SR											
**i/j**	**45**	**46**	**47**	**48**	**49**	**50**	**51**	**52**	**53**	**54**	**55**
**39**	-1.07865<br>0.2822	-2.13529<br>0.0341	-2.13529<br>0.0341	-2.13529<br>0.0341	-2.13529<br>0.0341	-2.13529<br>0.0341	-2.13529<br>0.0341	-2.13529<br>0.0341	-2.13529<br>0.0341	-1.0564<br>0.2922	-2.13529<br>0.0341
**40**	0.031699<br>0.9747	-1.02494<br>0.3068	-1.02494<br>0.3068	-1.02494<br>0.3068	-1.02494<br>0.3068	-1.02494<br>0.3068	-1.02494<br>0.3068	-1.02494<br>0.3068	-1.02494<br>0.3068	0.053944<br>0.9570	-1.02494<br>0.3068
**41**	0<br>1.0000	-1.05664<br>0.2921	-1.05664<br>0.2921	-1.05664<br>0.2921	-1.05664<br>0.2921	-1.05664<br>0.2921	-1.05664<br>0.2921	-1.05664<br>0.2921	-1.05664<br>0.2921	0.022245<br>0.9823	-1.05664<br>0.2921
**42**	1.056636<br>0.2921	0<br>1.0000	0<br>1.0000	0<br>1.0000	0<br>1.0000	0<br>1.0000	0<br>1.0000	0<br>1.0000	0<br>1.0000	1.078881<br>0.2821	0<br>1.0000
**43**	1.056636<br>0.2921	0<br>1.0000	0<br>1.0000	0<br>1.0000	0<br>1.0000	0<br>1.0000	0<br>1.0000	0<br>1.0000	0<br>1.0000	1.078881<br>0.2821	0<br>1.0000
**44**	1.056636<br>0.2921	0<br>1.0000	0<br>1.0000	0<br>1.0000	0<br>1.0000	0<br>1.0000	0<br>1.0000	0<br>1.0000	0<br>1.0000	1.078881<br>0.2821	0<br>1.0000
**45**		-1.05664<br>0.2921	-1.05664<br>0.2921	-1.05664<br>0.2921	-1.05664<br>0.2921	-1.05664<br>0.2921	-1.05664<br>0.2921	-1.05664<br>0.2921	-1.05664<br>0.2921	0.022245<br>0.9823	-1.05664<br>0.2921
**46**	1.056636<br>0.2921		0<br>1.0000	0<br>1.0000	0<br>1.0000	0<br>1.0000	0<br>1.0000	0<br>1.0000	0<br>1.0000	1.078881<br>0.2821	0<br>1.0000
**47**	1.056636<br>0.2921	0<br>1.0000		0<br>1.0000	0<br>1.0000	0<br>1.0000	0<br>1.0000	0<br>1.0000	0<br>1.0000	1.078881<br>0.2821	0<br>1.0000
**48**	1.056636<br>0.2921	0<br>1.0000	0<br>1.0000		0<br>1.0000	0<br>1.0000	0<br>1.0000	0<br>1.0000	0<br>1.0000	1.078881<br>0.2821	0<br>1.0000
**49**	1.056636<br>0.2921	0<br>1.0000	0<br>1.0000	0<br>1.0000		0<br>1.0000	0<br>1.0000	0<br>1.0000	0<br>1.0000	1.078881<br>0.2821	0<br>1.0000
**50**	1.056636<br>0.2921	0<br>1.0000	0<br>1.0000	0<br>1.0000	0<br>1.0000		0<br>1.0000	0<br>1.0000	0<br>1.0000	1.078881<br>0.2821	0<br>1.0000
**51**	1.056636<br>0.2921	0<br>1.0000	0<br>1.0000	0<br>1.0000	0<br>1.0000	0<br>1.0000		0<br>1.0000	0<br>1.0000	1.078881<br>0.2821	0<br>1.0000
**52**	1.056636<br>0.2921	0<br>1.0000	0<br>1.0000	0<br>1.0000	0<br>1.0000	0<br>1.0000	0<br>1.0000		0<br>1.0000	1.078881<br>0.2821	0<br>1.0000
**53**	1.056636<br>0.2921	0<br>1.0000	0<br>1.0000	0<br>1.0000	0<br>1.0000	0<br>1.0000	0<br>1.0000	0<br>1.0000		1.078881<br>0.2821	0<br>1.0000
**54**	-0.02224<br>0.9823	-1.07888<br>0.2821	-1.07888<br>0.2821	-1.07888<br>0.2821	-1.07888<br>0.2821	-1.07888<br>0.2821	-1.07888<br>0.2821	-1.07888<br>0.2821	-1.07888<br>0.2821		-1.07888<br>0.2821
**55**	1.056636<br>0.2921	0<br>1.0000	0<br>1.0000	0<br>1.0000	0<br>1.0000	0<br>1.0000	0<br>1.0000	0<br>1.0000	0<br>1.0000	1.078881<br>0.2821	
**56**	1.056636<br>0.2921	0<br>1.0000	0<br>1.0000	0<br>1.0000	0<br>1.0000	0<br>1.0000	0<br>1.0000	0<br>1.0000	0<br>1.0000	1.078881<br>0.2821	0<br>1.0000
**57**	1.056636<br>0.2921	0<br>1.0000	0<br>1.0000	0<br>1.0000	0<br>1.0000	0<br>1.0000	0<br>1.0000	0<br>1.0000	0<br>1.0000	1.078881<br>0.2821	0<br>1.0000

The SAS System

The GLM Procedure
Least Squares Means

Least Squares Means for Effect Day*Device
t for H0: LSMean(i)=LSMean(j) / Pr > |t|

Dependent Variable: SR

i/j	45	46	47	48	49	50	51	52	53	54	55
**58**	1.056636<br>0.2921	0<br>1.0000	0<br>1.0000	0<br>1.0000	0<br>1.0000	0<br>1.0000	0<br>1.0000	0<br>1.0000	0<br>1.0000	1.078881<br>0.2821	0<br>1.0000
**59**	1.056636<br>0.2921	0<br>1.0000	0<br>1.0000	0<br>1.0000	0<br>1.0000	0<br>1.0000	0<br>1.0000	0<br>1.0000	0<br>1.0000	1.078881<br>0.2821	0<br>1.0000
**60**	1.056636<br>0.2921	0<br>1.0000	0<br>1.0000	0<br>1.0000	0<br>1.0000	0<br>1.0000	0<br>1.0000	0<br>1.0000	0<br>1.0000	1.078881<br>0.2821	0<br>1.0000
**61**	1.056636<br>0.2921	0<br>1.0000	0<br>1.0000	0<br>1.0000	0<br>1.0000	0<br>1.0000	0<br>1.0000	0<br>1.0000	0<br>1.0000	1.078881<br>0.2821	0<br>1.0000
**62**	1.056636<br>0.2921	0<br>1.0000	0<br>1.0000	0<br>1.0000	0<br>1.0000	0<br>1.0000	0<br>1.0000	0<br>1.0000	0<br>1.0000	1.078881<br>0.2821	0<br>1.0000
**63**	1.056636<br>0.2921	0<br>1.0000	0<br>1.0000	0<br>1.0000	0<br>1.0000	0<br>1.0000	0<br>1.0000	0<br>1.0000	0<br>1.0000	1.078881<br>0.2821	0<br>1.0000
**64**	1.056636<br>0.2921	0<br>1.0000	0<br>1.0000	0<br>1.0000	0<br>1.0000	0<br>1.0000	0<br>1.0000	0<br>1.0000	0<br>1.0000	1.078881<br>0.2821	0<br>1.0000
**65**	1.056636<br>0.2921	0<br>1.0000	0<br>1.0000	0<br>1.0000	0<br>1.0000	0<br>1.0000	0<br>1.0000	0<br>1.0000	0<br>1.0000	1.078881<br>0.2821	0<br>1.0000
**66**	1.056636<br>0.2921	0<br>1.0000	0<br>1.0000	0<br>1.0000	0<br>1.0000	0<br>1.0000	0<br>1.0000	0<br>1.0000	0<br>1.0000	1.078881<br>0.2821	0<br>1.0000
**67**	0.021346<br>0.9830	-1.03529<br>0.3020	-1.03529<br>0.3020	-1.03529<br>0.3020	-1.03529<br>0.3020	-1.03529<br>0.3020	-1.03529<br>0.3020	-1.03529<br>0.3020	-1.03529<br>0.3020	0.043591<br>0.9653	-1.03529<br>0.3020
**68**	0<br>1.0000	-1.05664<br>0.2921	-1.05664<br>0.2921	-1.05664<br>0.2921	-1.05664<br>0.2921	-1.05664<br>0.2921	-1.05664<br>0.2921	-1.05664<br>0.2921	-1.05664<br>0.2921	0.022245<br>0.9823	-1.05664<br>0.2921
**69**	-0.01101<br>0.9912	-1.06764<br>0.2872	-1.06764<br>0.2872	-1.06764<br>0.2872	-1.06764<br>0.2872	-1.06764<br>0.2872	-1.06764<br>0.2872	-1.06764<br>0.2872	-1.06764<br>0.2872	0.011238<br>0.9910	-1.06764<br>0.2872
**70**	1.056636<br>0.2921	0<br>1.0000	0<br>1.0000	0<br>1.0000	0<br>1.0000	0<br>1.0000	0<br>1.0000	0<br>1.0000	0<br>1.0000	1.078881<br>0.2821	0<br>1.0000
**71**	1.056636<br>0.2921	0<br>1.0000	0<br>1.0000	0<br>1.0000	0<br>1.0000	0<br>1.0000	0<br>1.0000	0<br>1.0000	0<br>1.0000	1.078881<br>0.2821	0<br>1.0000
**72**	1.056636<br>0.2921	0<br>1.0000	0<br>1.0000	0<br>1.0000	0<br>1.0000	0<br>1.0000	0<br>1.0000	0<br>1.0000	0<br>1.0000	1.078881<br>0.2821	0<br>1.0000
**73**	1.056636<br>0.2921	0<br>1.0000	0<br>1.0000	0<br>1.0000	0<br>1.0000	0<br>1.0000	0<br>1.0000	0<br>1.0000	0<br>1.0000	1.078881<br>0.2821	0<br>1.0000
**74**	1.056636<br>0.2921	0<br>1.0000	0<br>1.0000	0<br>1.0000	0<br>1.0000	0<br>1.0000	0<br>1.0000	0<br>1.0000	0<br>1.0000	1.078881<br>0.2821	0<br>1.0000
**75**	-1.1126<br>0.2674	-2.16924<br>0.0314	-2.16924<br>0.0314	-2.16924<br>0.0314	-2.16924<br>0.0314	-2.16924<br>0.0314	-2.16924<br>0.0314	-2.16924<br>0.0314	-2.16924<br>0.0314	-1.09036<br>0.2771	-2.16924<br>0.0314
**76**	0.021346<br>0.9830	-1.03529<br>0.3020	-1.03529<br>0.3020	-1.03529<br>0.3020	-1.03529<br>0.3020	-1.03529<br>0.3020	-1.03529<br>0.3020	-1.03529<br>0.3020	-1.03529<br>0.3020	0.043591<br>0.9653	-1.03529<br>0.3020

The SAS System

The GLM Procedure
Least Squares Means

Least Squares Means for Effect Day*Device
t for H0: LSMean(i)=LSMean(j) / Pr > |t|

Dependent Variable: SR

i/j	45	46	47	48	49	50	51	52	53	54	55
**77**	1.056636<br>0.2921	0<br>1.0000	0<br>1.0000	0<br>1.0000	0<br>1.0000	0<br>1.0000	0<br>1.0000	0<br>1.0000	0<br>1.0000	1.078881<br>0.2821	0<br>1.0000
**78**	-0.03372<br>0.9731	-1.09036<br>0.2771	-1.09036<br>0.2771	-1.09036<br>0.2771	-1.09036<br>0.2771	-1.09036<br>0.2771	-1.09036<br>0.2771	-1.09036<br>0.2771	-1.09036<br>0.2771	-0.01148<br>0.9909	-1.09036<br>0.2771
**79**	1.056636<br>0.2921	0<br>1.0000	0<br>1.0000	0<br>1.0000	0<br>1.0000	0<br>1.0000	0<br>1.0000	0<br>1.0000	0<br>1.0000	1.078881<br>0.2821	0<br>1.0000
**80**	1.056636<br>0.2921	0<br>1.0000	0<br>1.0000	0<br>1.0000	0<br>1.0000	0<br>1.0000	0<br>1.0000	0<br>1.0000	0<br>1.0000	1.078881<br>0.2821	0<br>1.0000
**81**	1.056636<br>0.2921	0<br>1.0000	0<br>1.0000	0<br>1.0000	0<br>1.0000	0<br>1.0000	0<br>1.0000	0<br>1.0000	0<br>1.0000	1.078881<br>0.2821	0<br>1.0000
**82**	1.056636<br>0.2921	0<br>1.0000	0<br>1.0000	0<br>1.0000	0<br>1.0000	0<br>1.0000	0<br>1.0000	0<br>1.0000	0<br>1.0000	1.078881<br>0.2821	0<br>1.0000
**83**	-0.01101<br>0.9912	-1.06764<br>0.2872	-1.06764<br>0.2872	-1.06764<br>0.2872	-1.06764<br>0.2872	-1.06764<br>0.2872	-1.06764<br>0.2872	-1.06764<br>0.2872	-1.06764<br>0.2872	0.011238<br>0.9910	-1.06764<br>0.2872
**84**	-0.04545<br>0.9638	-1.10208<br>0.2719	-1.10208<br>0.2719	-1.10208<br>0.2719	-1.10208<br>0.2719	-1.10208<br>0.2719	-1.10208<br>0.2719	-1.10208<br>0.2719	-1.10208<br>0.2719	-0.0232<br>0.9815	-1.10208<br>0.2719
**85**	0.010782<br>0.9914	-1.04585<br>0.2971	-1.04585<br>0.2971	-1.04585<br>0.2971	-1.04585<br>0.2971	-1.04585<br>0.2971	-1.04585<br>0.2971	-1.04585<br>0.2971	-1.04585<br>0.2971	0.033027<br>0.9737	-1.04585<br>0.2971
**86**	1.056636<br>0.2921	0<br>1.0000	0<br>1.0000	0<br>1.0000	0<br>1.0000	0<br>1.0000	0<br>1.0000	0<br>1.0000	0<br>1.0000	1.078881<br>0.2821	0<br>1.0000
**87**	-1.17149<br>0.2430	-2.22812<br>0.0272	-2.22812<br>0.0272	-2.22812<br>0.0272	-2.22812<br>0.0272	-2.22812<br>0.0272	-2.22812<br>0.0272	-2.22812<br>0.0272	-2.22812<br>0.0272	-1.14924<br>0.2520	-2.22812<br>0.0272

Least Squares Means for Effect Day*Device
t for H0: LSMean(i)=LSMean(j) / Pr > |t|

Dependent Variable: SR

i/j	56	57	58	59	60	61	62	63	64	65	66
**1**	0<br>1.0000	0<br>1.0000	0<br>1.0000	0<br>1.0000	0<br>1.0000	0<br>1.0000	0<br>1.0000	0<br>1.0000	0<br>1.0000	0<br>1.0000	0<br>1.0000
**2**	0<br>1.0000	0<br>1.0000	0<br>1.0000	0<br>1.0000	0<br>1.0000	0<br>1.0000	0<br>1.0000	0<br>1.0000	0<br>1.0000	0<br>1.0000	0<br>1.0000
**3**	0<br>1.0000	0<br>1.0000	0<br>1.0000	0<br>1.0000	0<br>1.0000	0<br>1.0000	0<br>1.0000	0<br>1.0000	0<br>1.0000	0<br>1.0000	0<br>1.0000
**4**	0<br>1.0000	0<br>1.0000	0<br>1.0000	0<br>1.0000	0<br>1.0000	0<br>1.0000	0<br>1.0000	0<br>1.0000	0<br>1.0000	0<br>1.0000	0<br>1.0000
**5**	0<br>1.0000	0<br>1.0000	0<br>1.0000	0<br>1.0000	0<br>1.0000	0<br>1.0000	0<br>1.0000	0<br>1.0000	0<br>1.0000	0<br>1.0000	0<br>1.0000

The SAS System

The GLM Procedure
Least Squares Means

Least Squares Means for Effect Day*Device
t for H0: LSMean(i)=LSMean(j) / Pr > |t|

Dependent Variable: SR

i/j	56	57	58	59	60	61	62	63	64	65	66
**6**	-1.02494<br>0.3068	-1.02494<br>0.3068	-1.02494<br>0.3068	-1.02494<br>0.3068	-1.02494<br>0.3068	-1.02494<br>0.3068	-1.02494<br>0.3068	-1.02494<br>0.3068	-1.02494<br>0.3068	-1.02494<br>0.3068	-1.02494<br>0.3068
**7**	0<br>1.0000	0<br>1.0000	0<br>1.0000	0<br>1.0000	0<br>1.0000	0<br>1.0000	0<br>1.0000	0<br>1.0000	0<br>1.0000	0<br>1.0000	0<br>1.0000
**8**	0<br>1.0000	0<br>1.0000	0<br>1.0000	0<br>1.0000	0<br>1.0000	0<br>1.0000	0<br>1.0000	0<br>1.0000	0<br>1.0000	0<br>1.0000	0<br>1.0000
**9**	0<br>1.0000	0<br>1.0000	0<br>1.0000	0<br>1.0000	0<br>1.0000	0<br>1.0000	0<br>1.0000	0<br>1.0000	0<br>1.0000	0<br>1.0000	0<br>1.0000
**10**	-2.04987<br>0.0419	-2.04987<br>0.0419	-2.04987<br>0.0419	-2.04987<br>0.0419	-2.04987<br>0.0419	-2.04987<br>0.0419	-2.04987<br>0.0419	-2.04987<br>0.0419	-2.04987<br>0.0419	-2.04987<br>0.0419	-2.04987<br>0.0419
**11**	-5.12469<br><.0001	-5.12469<br><.0001	-5.12469<br><.0001	-5.12469<br><.0001	-5.12469<br><.0001	-5.12469<br><.0001	-5.12469<br><.0001	-5.12469<br><.0001	-5.12469<br><.0001	-5.12469<br><.0001	-5.12469<br><.0001
**12**	-4.13081<br><.0001	-4.13081<br><.0001	-4.13081<br><.0001	-4.13081<br><.0001	-4.13081<br><.0001	-4.13081<br><.0001	-4.13081<br><.0001	-4.13081<br><.0001	-4.13081<br><.0001	-4.13081<br><.0001	-4.13081<br><.0001
**13**	-1.02494<br>0.3068	-1.02494<br>0.3068	-1.02494<br>0.3068	-1.02494<br>0.3068	-1.02494<br>0.3068	-1.02494<br>0.3068	-1.02494<br>0.3068	-1.02494<br>0.3068	-1.02494<br>0.3068	-1.02494<br>0.3068	-1.02494<br>0.3068
**14**	-4.16251<br><.0001	-4.16251<br><.0001	-4.16251<br><.0001	-4.16251<br><.0001	-4.16251<br><.0001	-4.16251<br><.0001	-4.16251<br><.0001	-4.16251<br><.0001	-4.16251<br><.0001	-4.16251<br><.0001	-4.16251<br><.0001
**15**	-3.13778<br>0.0020	-3.13778<br>0.0020	-3.13778<br>0.0020	-3.13778<br>0.0020	-3.13778<br>0.0020	-3.13778<br>0.0020	-3.13778<br>0.0020	-3.13778<br>0.0020	-3.13778<br>0.0020	-3.13778<br>0.0020	-3.13778<br>0.0020
**16**	0<br>1.0000	0<br>1.0000	0<br>1.0000	0<br>1.0000	0<br>1.0000	0<br>1.0000	0<br>1.0000	0<br>1.0000	0<br>1.0000	0<br>1.0000	0<br>1.0000
**17**	0<br>1.0000	0<br>1.0000	0<br>1.0000	0<br>1.0000	0<br>1.0000	0<br>1.0000	0<br>1.0000	0<br>1.0000	0<br>1.0000	0<br>1.0000	0<br>1.0000
**18**	-1.04585<br>0.2971	-1.04585<br>0.2971	-1.04585<br>0.2971	-1.04585<br>0.2971	-1.04585<br>0.2971	-1.04585<br>0.2971	-1.04585<br>0.2971	-1.04585<br>0.2971	-1.04585<br>0.2971	-1.04585<br>0.2971	-1.04585<br>0.2971
**19**	0<br>1.0000	0<br>1.0000	0<br>1.0000	0<br>1.0000	0<br>1.0000	0<br>1.0000	0<br>1.0000	0<br>1.0000	0<br>1.0000	0<br>1.0000	0<br>1.0000
**20**	-2.1135<br>0.0360	-2.1135<br>0.0360	-2.1135<br>0.0360	-2.1135<br>0.0360	-2.1135<br>0.0360	-2.1135<br>0.0360	-2.1135<br>0.0360	-2.1135<br>0.0360	-2.1135<br>0.0360	-2.1135<br>0.0360	-2.1135<br>0.0360
**21**	0<br>1.0000	0<br>1.0000	0<br>1.0000	0<br>1.0000	0<br>1.0000	0<br>1.0000	0<br>1.0000	0<br>1.0000	0<br>1.0000	0<br>1.0000	0<br>1.0000
**22**	0<br>1.0000	0<br>1.0000	0<br>1.0000	0<br>1.0000	0<br>1.0000	0<br>1.0000	0<br>1.0000	0<br>1.0000	0<br>1.0000	0<br>1.0000	0<br>1.0000
**23**	0<br>1.0000	0<br>1.0000	0<br>1.0000	0<br>1.0000	0<br>1.0000	0<br>1.0000	0<br>1.0000	0<br>1.0000	0<br>1.0000	0<br>1.0000	0<br>1.0000
**24**	0<br>1.0000	0<br>1.0000	0<br>1.0000	0<br>1.0000	0<br>1.0000	0<br>1.0000	0<br>1.0000	0<br>1.0000	0<br>1.0000	0<br>1.0000	0<br>1.0000

The SAS System

The GLM Procedure
Least Squares Means

Least Squares Means for Effect Day*Device<br>t for H0: LSMean(i)=LSMean(j) / Pr > |t| <br>Dependent Variable: SR											
**i/j**	**56**	**57**	**58**	**59**	**60**	**61**	**62**	**63**	**64**	**65**	**66**
**25**	0<br>1.0000	0<br>1.0000	0<br>1.0000	0<br>1.0000	0<br>1.0000	0<br>1.0000	0<br>1.0000	0<br>1.0000	0<br>1.0000	0<br>1.0000	0<br>1.0000
**26**	0<br>1.0000	0<br>1.0000	0<br>1.0000	0<br>1.0000	0<br>1.0000	0<br>1.0000	0<br>1.0000	0<br>1.0000	0<br>1.0000	0<br>1.0000	0<br>1.0000
**27**	0<br>1.0000	0<br>1.0000	0<br>1.0000	0<br>1.0000	0<br>1.0000	0<br>1.0000	0<br>1.0000	0<br>1.0000	0<br>1.0000	0<br>1.0000	0<br>1.0000
**28**	0<br>1.0000	0<br>1.0000	0<br>1.0000	0<br>1.0000	0<br>1.0000	0<br>1.0000	0<br>1.0000	0<br>1.0000	0<br>1.0000	0<br>1.0000	0<br>1.0000
**29**	0<br>1.0000	0<br>1.0000	0<br>1.0000	0<br>1.0000	0<br>1.0000	0<br>1.0000	0<br>1.0000	0<br>1.0000	0<br>1.0000	0<br>1.0000	0<br>1.0000
**30**	0<br>1.0000	0<br>1.0000	0<br>1.0000	0<br>1.0000	0<br>1.0000	0<br>1.0000	0<br>1.0000	0<br>1.0000	0<br>1.0000	0<br>1.0000	0<br>1.0000
**31**	0<br>1.0000	0<br>1.0000	0<br>1.0000	0<br>1.0000	0<br>1.0000	0<br>1.0000	0<br>1.0000	0<br>1.0000	0<br>1.0000	0<br>1.0000	0<br>1.0000
**32**	0<br>1.0000	0<br>1.0000	0<br>1.0000	0<br>1.0000	0<br>1.0000	0<br>1.0000	0<br>1.0000	0<br>1.0000	0<br>1.0000	0<br>1.0000	0<br>1.0000
**33**	0<br>1.0000	0<br>1.0000	0<br>1.0000	0<br>1.0000	0<br>1.0000	0<br>1.0000	0<br>1.0000	0<br>1.0000	0<br>1.0000	0<br>1.0000	0<br>1.0000
**34**	0<br>1.0000	0<br>1.0000	0<br>1.0000	0<br>1.0000	0<br>1.0000	0<br>1.0000	0<br>1.0000	0<br>1.0000	0<br>1.0000	0<br>1.0000	0<br>1.0000
**35**	0<br>1.0000	0<br>1.0000	0<br>1.0000	0<br>1.0000	0<br>1.0000	0<br>1.0000	0<br>1.0000	0<br>1.0000	0<br>1.0000	0<br>1.0000	0<br>1.0000
**36**	-2.11327<br>0.0360	-2.11327<br>0.0360	-2.11327<br>0.0360	-2.11327<br>0.0360	-2.11327<br>0.0360	-2.11327<br>0.0360	-2.11327<br>0.0360	-2.11327<br>0.0360	-2.11327<br>0.0360	-2.11327<br>0.0360	-2.11327<br>0.0360
**37**	0<br>1.0000	0<br>1.0000	0<br>1.0000	0<br>1.0000	0<br>1.0000	0<br>1.0000	0<br>1.0000	0<br>1.0000	0<br>1.0000	0<br>1.0000	0<br>1.0000
**38**	0<br>1.0000	0<br>1.0000	0<br>1.0000	0<br>1.0000	0<br>1.0000	0<br>1.0000	0<br>1.0000	0<br>1.0000	0<br>1.0000	0<br>1.0000	0<br>1.0000
**39**	-2.13529<br>0.0341	-2.13529<br>0.0341	-2.13529<br>0.0341	-2.13529<br>0.0341	-2.13529<br>0.0341	-2.13529<br>0.0341	-2.13529<br>0.0341	-2.13529<br>0.0341	-2.13529<br>0.0341	-2.13529<br>0.0341	-2.13529<br>0.0341
**40**	-1.02494<br>0.3068	-1.02494<br>0.3068	-1.02494<br>0.3068	-1.02494<br>0.3068	-1.02494<br>0.3068	-1.02494<br>0.3068	-1.02494<br>0.3068	-1.02494<br>0.3068	-1.02494<br>0.3068	-1.02494<br>0.3068	-1.02494<br>0.3068
**41**	-1.05664<br>0.2921	-1.05664<br>0.2921	-1.05664<br>0.2921	-1.05664<br>0.2921	-1.05664<br>0.2921	-1.05664<br>0.2921	-1.05664<br>0.2921	-1.05664<br>0.2921	-1.05664<br>0.2921	-1.05664<br>0.2921	-1.05664<br>0.2921
**42**	0<br>1.0000	0<br>1.0000	0<br>1.0000	0<br>1.0000	0<br>1.0000	0<br>1.0000	0<br>1.0000	0<br>1.0000	0<br>1.0000	0<br>1.0000	0<br>1.0000
**43**	0<br>1.0000	0<br>1.0000	0<br>1.0000	0<br>1.0000	0<br>1.0000	0<br>1.0000	0<br>1.0000	0<br>1.0000	0<br>1.0000	0<br>1.0000	0<br>1.0000

The SAS System

The GLM Procedure
Least Squares Means

Least Squares Means for Effect Day*Device
t for H0: LSMean(i)=LSMean(j) / Pr > |t|

Dependent Variable: SR

i/j	56	57	58	59	60	61	62	63	64	65	66
**44**	0<br>1.0000	0<br>1.0000	0<br>1.0000	0<br>1.0000	0<br>1.0000	0<br>1.0000	0<br>1.0000	0<br>1.0000	0<br>1.0000	0<br>1.0000	0<br>1.0000
**45**	-1.05664<br>0.2921	-1.05664<br>0.2921	-1.05664<br>0.2921	-1.05664<br>0.2921	-1.05664<br>0.2921	-1.05664<br>0.2921	-1.05664<br>0.2921	-1.05664<br>0.2921	-1.05664<br>0.2921	-1.05664<br>0.2921	-1.05664<br>0.2921
**46**	0<br>1.0000	0<br>1.0000	0<br>1.0000	0<br>1.0000	0<br>1.0000	0<br>1.0000	0<br>1.0000	0<br>1.0000	0<br>1.0000	0<br>1.0000	0<br>1.0000
**47**	0<br>1.0000	0<br>1.0000	0<br>1.0000	0<br>1.0000	0<br>1.0000	0<br>1.0000	0<br>1.0000	0<br>1.0000	0<br>1.0000	0<br>1.0000	0<br>1.0000
**48**	0<br>1.0000	0<br>1.0000	0<br>1.0000	0<br>1.0000	0<br>1.0000	0<br>1.0000	0<br>1.0000	0<br>1.0000	0<br>1.0000	0<br>1.0000	0<br>1.0000
**49**	0<br>1.0000	0<br>1.0000	0<br>1.0000	0<br>1.0000	0<br>1.0000	0<br>1.0000	0<br>1.0000	0<br>1.0000	0<br>1.0000	0<br>1.0000	0<br>1.0000
**50**	0<br>1.0000	0<br>1.0000	0<br>1.0000	0<br>1.0000	0<br>1.0000	0<br>1.0000	0<br>1.0000	0<br>1.0000	0<br>1.0000	0<br>1.0000	0<br>1.0000
**51**	0<br>1.0000	0<br>1.0000	0<br>1.0000	0<br>1.0000	0<br>1.0000	0<br>1.0000	0<br>1.0000	0<br>1.0000	0<br>1.0000	0<br>1.0000	0<br>1.0000
**52**	0<br>1.0000	0<br>1.0000	0<br>1.0000	0<br>1.0000	0<br>1.0000	0<br>1.0000	0<br>1.0000	0<br>1.0000	0<br>1.0000	0<br>1.0000	0<br>1.0000
**53**	0<br>1.0000	0<br>1.0000	0<br>1.0000	0<br>1.0000	0<br>1.0000	0<br>1.0000	0<br>1.0000	0<br>1.0000	0<br>1.0000	0<br>1.0000	0<br>1.0000
**54**	-1.07888<br>0.2821	-1.07888<br>0.2821	-1.07888<br>0.2821	-1.07888<br>0.2821	-1.07888<br>0.2821	-1.07888<br>0.2821	-1.07888<br>0.2821	-1.07888<br>0.2821	-1.07888<br>0.2821	-1.07888<br>0.2821	-1.07888<br>0.2821
**55**	0<br>1.0000	0<br>1.0000	0<br>1.0000	0<br>1.0000	0<br>1.0000	0<br>1.0000	0<br>1.0000	0<br>1.0000	0<br>1.0000	0<br>1.0000	0<br>1.0000
**56**		0<br>1.0000	0<br>1.0000	0<br>1.0000	0<br>1.0000	0<br>1.0000	0<br>1.0000	0<br>1.0000	0<br>1.0000	0<br>1.0000	0<br>1.0000
**57**	0<br>1.0000		0<br>1.0000	0<br>1.0000	0<br>1.0000	0<br>1.0000	0<br>1.0000	0<br>1.0000	0<br>1.0000	0<br>1.0000	0<br>1.0000
**58**	0<br>1.0000	0<br>1.0000		0<br>1.0000	0<br>1.0000	0<br>1.0000	0<br>1.0000	0<br>1.0000	0<br>1.0000	0<br>1.0000	0<br>1.0000
**59**	0<br>1.0000	0<br>1.0000	0<br>1.0000		0<br>1.0000	0<br>1.0000	0<br>1.0000	0<br>1.0000	0<br>1.0000	0<br>1.0000	0<br>1.0000
**60**	0<br>1.0000	0<br>1.0000	0<br>1.0000	0<br>1.0000		0<br>1.0000	0<br>1.0000	0<br>1.0000	0<br>1.0000	0<br>1.0000	0<br>1.0000
**61**	0<br>1.0000	0<br>1.0000	0<br>1.0000	0<br>1.0000	0<br>1.0000		0<br>1.0000	0<br>1.0000	0<br>1.0000	0<br>1.0000	0<br>1.0000
**62**	0<br>1.0000	0<br>1.0000	0<br>1.0000	0<br>1.0000	0<br>1.0000	0<br>1.0000		0<br>1.0000	0<br>1.0000	0<br>1.0000	0<br>1.0000

The SAS System

The GLM Procedure
Least Squares Means

Least Squares Means for Effect Day*Device<br>t for H0: LSMean(i)=LSMean(j) / Pr > |t| <br>Dependent Variable: SR											
i/j	56	57	58	59	60	61	62	63	64	65	66
63	0<br>1.0000	0<br>1.0000	0<br>1.0000	0<br>1.0000	0<br>1.0000	0<br>1.0000	0<br>1.0000		0<br>1.0000	0<br>1.0000	0<br>1.0000
64	0<br>1.0000	0<br>1.0000	0<br>1.0000	0<br>1.0000	0<br>1.0000	0<br>1.0000	0<br>1.0000	0<br>1.0000		0<br>1.0000	0<br>1.0000
65	0<br>1.0000	0<br>1.0000	0<br>1.0000	0<br>1.0000	0<br>1.0000	0<br>1.0000	0<br>1.0000	0<br>1.0000	0<br>1.0000		0<br>1.0000
66	0<br>1.0000	0<br>1.0000	0<br>1.0000	0<br>1.0000	0<br>1.0000	0<br>1.0000	0<br>1.0000	0<br>1.0000	0<br>1.0000	0<br>1.0000	
67	-1.03529<br>0.3020	-1.03529<br>0.3020	-1.03529<br>0.3020	-1.03529<br>0.3020	-1.03529<br>0.3020	-1.03529<br>0.3020	-1.03529<br>0.3020	-1.03529<br>0.3020	-1.03529<br>0.3020	-1.03529<br>0.3020	-1.03529<br>0.3020
68	-1.05664<br>0.2921	-1.05664<br>0.2921	-1.05664<br>0.2921	-1.05664<br>0.2921	-1.05664<br>0.2921	-1.05664<br>0.2921	-1.05664<br>0.2921	-1.05664<br>0.2921	-1.05664<br>0.2921	-1.05664<br>0.2921	-1.05664<br>0.2921
69	-1.06764<br>0.2872	-1.06764<br>0.2872	-1.06764<br>0.2872	-1.06764<br>0.2872	-1.06764<br>0.2872	-1.06764<br>0.2872	-1.06764<br>0.2872	-1.06764<br>0.2872	-1.06764<br>0.2872	-1.06764<br>0.2872	-1.06764<br>0.2872
70	0<br>1.0000	0<br>1.0000	0<br>1.0000	0<br>1.0000	0<br>1.0000	0<br>1.0000	0<br>1.0000	0<br>1.0000	0<br>1.0000	0<br>1.0000	0<br>1.0000
71	0<br>1.0000	0<br>1.0000	0<br>1.0000	0<br>1.0000	0<br>1.0000	0<br>1.0000	0<br>1.0000	0<br>1.0000	0<br>1.0000	0<br>1.0000	0<br>1.0000
72	0<br>1.0000	0<br>1.0000	0<br>1.0000	0<br>1.0000	0<br>1.0000	0<br>1.0000	0<br>1.0000	0<br>1.0000	0<br>1.0000	0<br>1.0000	0<br>1.0000
73	0<br>1.0000	0<br>1.0000	0<br>1.0000	0<br>1.0000	0<br>1.0000	0<br>1.0000	0<br>1.0000	0<br>1.0000	0<br>1.0000	0<br>1.0000	0<br>1.0000
74	0<br>1.0000	0<br>1.0000	0<br>1.0000	0<br>1.0000	0<br>1.0000	0<br>1.0000	0<br>1.0000	0<br>1.0000	0<br>1.0000	0<br>1.0000	0<br>1.0000
75	-2.16924<br>0.0314	-2.16924<br>0.0314	-2.16924<br>0.0314	-2.16924<br>0.0314	-2.16924<br>0.0314	-2.16924<br>0.0314	-2.16924<br>0.0314	-2.16924<br>0.0314	-2.16924<br>0.0314	-2.16924<br>0.0314	-2.16924<br>0.0314
76	-1.03529<br>0.3020	-1.03529<br>0.3020	-1.03529<br>0.3020	-1.03529<br>0.3020	-1.03529<br>0.3020	-1.03529<br>0.3020	-1.03529<br>0.3020	-1.03529<br>0.3020	-1.03529<br>0.3020	-1.03529<br>0.3020	-1.03529<br>0.3020
77	0<br>1.0000	0<br>1.0000	0<br>1.0000	0<br>1.0000	0<br>1.0000	0<br>1.0000	0<br>1.0000	0<br>1.0000	0<br>1.0000	0<br>1.0000	0<br>1.0000
78	-1.09036<br>0.2771	-1.09036<br>0.2771	-1.09036<br>0.2771	-1.09036<br>0.2771	-1.09036<br>0.2771	-1.09036<br>0.2771	-1.09036<br>0.2771	-1.09036<br>0.2771	-1.09036<br>0.2771	-1.09036<br>0.2771	-1.09036<br>0.2771
79	0<br>1.0000	0<br>1.0000	0<br>1.0000	0<br>1.0000	0<br>1.0000	0<br>1.0000	0<br>1.0000	0<br>1.0000	0<br>1.0000	0<br>1.0000	0<br>1.0000
80	0<br>1.0000	0<br>1.0000	0<br>1.0000	0<br>1.0000	0<br>1.0000	0<br>1.0000	0<br>1.0000	0<br>1.0000	0<br>1.0000	0<br>1.0000	0<br>1.0000
81	0<br>1.0000	0<br>1.0000	0<br>1.0000	0<br>1.0000	0<br>1.0000	0<br>1.0000	0<br>1.0000	0<br>1.0000	0<br>1.0000	0<br>1.0000	0<br>1.0000

The SAS System

The GLM Procedure
Least Squares Means

Least Squares Means for Effect Day*Device
t for H0: LSMean(i)=LSMean(j) / Pr > |t|

Dependent Variable: SR

i/j	56	57	58	59	60	61	62	63	64	65	66
**82**	0<br>1.0000	0<br>1.0000	0<br>1.0000	0<br>1.0000	0<br>1.0000	0<br>1.0000	0<br>1.0000	0<br>1.0000	0<br>1.0000	0<br>1.0000	0<br>1.0000
**83**	-1.06764<br>0.2872	-1.06764<br>0.2872	-1.06764<br>0.2872	-1.06764<br>0.2872	-1.06764<br>0.2872	-1.06764<br>0.2872	-1.06764<br>0.2872	-1.06764<br>0.2872	-1.06764<br>0.2872	-1.06764<br>0.2872	-1.06764<br>0.2872
**84**	-1.10208<br>0.2719	-1.10208<br>0.2719	-1.10208<br>0.2719	-1.10208<br>0.2719	-1.10208<br>0.2719	-1.10208<br>0.2719	-1.10208<br>0.2719	-1.10208<br>0.2719	-1.10208<br>0.2719	-1.10208<br>0.2719	-1.10208<br>0.2719
**85**	-1.04585<br>0.2971	-1.04585<br>0.2971	-1.04585<br>0.2971	-1.04585<br>0.2971	-1.04585<br>0.2971	-1.04585<br>0.2971	-1.04585<br>0.2971	-1.04585<br>0.2971	-1.04585<br>0.2971	-1.04585<br>0.2971	-1.04585<br>0.2971
**86**	0<br>1.0000	0<br>1.0000	0<br>1.0000	0<br>1.0000	0<br>1.0000	0<br>1.0000	0<br>1.0000	0<br>1.0000	0<br>1.0000	0<br>1.0000	0<br>1.0000
**87**	-2.22812<br>0.0272	-2.22812<br>0.0272	-2.22812<br>0.0272	-2.22812<br>0.0272	-2.22812<br>0.0272	-2.22812<br>0.0272	-2.22812<br>0.0272	-2.22812<br>0.0272	-2.22812<br>0.0272	-2.22812<br>0.0272	-2.22812<br>0.0272

Least Squares Means for Effect Day*Device
t for H0: LSMean(i)=LSMean(j) / Pr > |t|

Dependent Variable: SR

i/j	67	68	69	70	71	72	73	74	75	76	77
**1**	1.03529<br>0.3020	1.056636<br>0.2921	1.067643<br>0.2872	0<br>1.0000	0<br>1.0000	0<br>1.0000	0<br>1.0000	0<br>1.0000	2.16924<br>0.0314	1.03529<br>0.3020	0<br>1.0000
**2**	1.03529<br>0.3020	1.056636<br>0.2921	1.067643<br>0.2872	0<br>1.0000	0<br>1.0000	0<br>1.0000	0<br>1.0000	0<br>1.0000	2.16924<br>0.0314	1.03529<br>0.3020	0<br>1.0000
**3**	1.03529<br>0.3020	1.056636<br>0.2921	1.067643<br>0.2872	0<br>1.0000	0<br>1.0000	0<br>1.0000	0<br>1.0000	0<br>1.0000	2.16924<br>0.0314	1.03529<br>0.3020	0<br>1.0000
**4**	1.03529<br>0.3020	1.056636<br>0.2921	1.067643<br>0.2872	0<br>1.0000	0<br>1.0000	0<br>1.0000	0<br>1.0000	0<br>1.0000	2.16924<br>0.0314	1.03529<br>0.3020	0<br>1.0000
**5**	1.03529<br>0.3020	1.056636<br>0.2921	1.067643<br>0.2872	0<br>1.0000	0<br>1.0000	0<br>1.0000	0<br>1.0000	0<br>1.0000	2.16924<br>0.0314	1.03529<br>0.3020	0<br>1.0000
**6**	0.010353<br>0.9918	0.031699<br>0.9747	0.042706<br>0.9660	-1.02494<br>0.3068	-1.02494<br>0.3068	-1.02494<br>0.3068	-1.02494<br>0.3068	-1.02494<br>0.3068	1.144303<br>0.2541	0.010353<br>0.9918	-1.02494<br>0.3068
**7**	1.03529<br>0.3020	1.056636<br>0.2921	1.067643<br>0.2872	0<br>1.0000	0<br>1.0000	0<br>1.0000	0<br>1.0000	0<br>1.0000	2.16924<br>0.0314	1.03529<br>0.3020	0<br>1.0000
**8**	1.03529<br>0.3020	1.056636<br>0.2921	1.067643<br>0.2872	0<br>1.0000	0<br>1.0000	0<br>1.0000	0<br>1.0000	0<br>1.0000	2.16924<br>0.0314	1.03529<br>0.3020	0<br>1.0000
**9**	1.03529<br>0.3020	1.056636<br>0.2921	1.067643<br>0.2872	0<br>1.0000	0<br>1.0000	0<br>1.0000	0<br>1.0000	0<br>1.0000	2.16924<br>0.0314	1.03529<br>0.3020	0<br>1.0000
**10**	-1.01458<br>0.3117	-0.99324<br>0.3220	-0.98223<br>0.3273	-2.04987<br>0.0419	-2.04987<br>0.0419	-2.04987<br>0.0419	-2.04987<br>0.0419	-2.04987<br>0.0419	0.119366<br>0.9051	-1.01458<br>0.3117	-2.04987<br>0.0419

The SAS System

The GLM Procedure
Least Squares Means

Least Squares Means for Effect Day*Device
t for H0: LSMean(i)=LSMean(j) / Pr > |t|

Dependent Variable: SR

i/j	67	68	69	70	71	72	73	74	75	76	77
**11**	-4.0894<br><.0001	-4.06805<br><.0001	-4.05704<br><.0001	-5.12469<br><.0001	-5.12469<br><.0001	-5.12469<br><.0001	-5.12469<br><.0001	-5.12469<br><.0001	-2.95545<br>0.0036	-4.0894<br><.0001	-5.12469<br><.0001
**12**	-3.09552<br>0.0023	-3.07417<br>0.0025	-3.06316<br>0.0025	-4.13081<br><.0001	-4.13081<br><.0001	-4.13081<br><.0001	-4.13081<br><.0001	-4.13081<br><.0001	-1.96157<br>0.0514	-3.09552<br>0.0023	-4.13081<br><.0001
**13**	0.010353<br>0.9918	0.031699<br>0.9747	0.042706<br>0.9660	-1.02494<br>0.3068	-1.02494<br>0.3068	-1.02494<br>0.3068	-1.02494<br>0.3068	-1.02494<br>0.3068	1.144303<br>0.2541	0.010353<br>0.9918	-1.02494<br>0.3068
**14**	-3.12722<br>0.0021	-3.10587<br>0.0022	-3.09486<br>0.0023	-4.16251<br><.0001	-4.16251<br><.0001	-4.16251<br><.0001	-4.16251<br><.0001	-4.16251<br><.0001	-1.99327<br>0.0478	-3.12722<br>0.0021	-4.16251<br><.0001
**15**	-2.10249<br>0.0369	-2.08114<br>0.0389	-2.07014<br>0.0399	-3.13778<br>0.0020	-3.13778<br>0.0020	-3.13778<br>0.0020	-3.13778<br>0.0020	-3.13778<br>0.0020	-0.96854<br>0.3341	-2.10249<br>0.0369	-3.13778<br>0.0020
**16**	1.03529<br>0.3020	1.056636<br>0.2921	1.067643<br>0.2872	0<br>1.0000	0<br>1.0000	0<br>1.0000	0<br>1.0000	0<br>1.0000	2.16924<br>0.0314	1.03529<br>0.3020	0<br>1.0000
**17**	1.03529<br>0.3020	1.056636<br>0.2921	1.067643<br>0.2872	0<br>1.0000	0<br>1.0000	0<br>1.0000	0<br>1.0000	0<br>1.0000	2.16924<br>0.0314	1.03529<br>0.3020	0<br>1.0000
**18**	-0.01056<br>0.9916	0.010782<br>0.9914	0.021789<br>0.9826	-1.04585<br>0.2971	-1.04585<br>0.2971	-1.04585<br>0.2971	-1.04585<br>0.2971	-1.04585<br>0.2971	1.123386<br>0.2628	-0.01056<br>0.9916	-1.04585<br>0.2971
**19**	1.03529<br>0.3020	1.056636<br>0.2921	1.067643<br>0.2872	0<br>1.0000	0<br>1.0000	0<br>1.0000	0<br>1.0000	0<br>1.0000	2.16924<br>0.0314	1.03529<br>0.3020	0<br>1.0000
**20**	-1.07821<br>0.2824	-1.05686<br>0.2920	-1.04585<br>0.2971	-2.1135<br>0.0360	-2.1135<br>0.0360	-2.1135<br>0.0360	-2.1135<br>0.0360	-2.1135<br>0.0360	0.055743<br>0.9556	-1.07821<br>0.2824	-2.1135<br>0.0360
**21**	1.03529<br>0.3020	1.056636<br>0.2921	1.067643<br>0.2872	0<br>1.0000	0<br>1.0000	0<br>1.0000	0<br>1.0000	0<br>1.0000	2.16924<br>0.0314	1.03529<br>0.3020	0<br>1.0000
**22**	1.03529<br>0.3020	1.056636<br>0.2921	1.067643<br>0.2872	0<br>1.0000	0<br>1.0000	0<br>1.0000	0<br>1.0000	0<br>1.0000	2.16924<br>0.0314	1.03529<br>0.3020	0<br>1.0000
**23**	1.03529<br>0.3020	1.056636<br>0.2921	1.067643<br>0.2872	0<br>1.0000	0<br>1.0000	0<br>1.0000	0<br>1.0000	0<br>1.0000	2.16924<br>0.0314	1.03529<br>0.3020	0<br>1.0000
**24**	1.03529<br>0.3020	1.056636<br>0.2921	1.067643<br>0.2872	0<br>1.0000	0<br>1.0000	0<br>1.0000	0<br>1.0000	0<br>1.0000	2.16924<br>0.0314	1.03529<br>0.3020	0<br>1.0000
**25**	1.03529<br>0.3020	1.056636<br>0.2921	1.067643<br>0.2872	0<br>1.0000	0<br>1.0000	0<br>1.0000	0<br>1.0000	0<br>1.0000	2.16924<br>0.0314	1.03529<br>0.3020	0<br>1.0000
**26**	1.03529<br>0.3020	1.056636<br>0.2921	1.067643<br>0.2872	0<br>1.0000	0<br>1.0000	0<br>1.0000	0<br>1.0000	0<br>1.0000	2.16924<br>0.0314	1.03529<br>0.3020	0<br>1.0000
**27**	1.03529<br>0.3020	1.056636<br>0.2921	1.067643<br>0.2872	0<br>1.0000	0<br>1.0000	0<br>1.0000	0<br>1.0000	0<br>1.0000	2.16924<br>0.0314	1.03529<br>0.3020	0<br>1.0000
**28**	1.03529<br>0.3020	1.056636<br>0.2921	1.067643<br>0.2872	0<br>1.0000	0<br>1.0000	0<br>1.0000	0<br>1.0000	0<br>1.0000	2.16924<br>0.0314	1.03529<br>0.3020	0<br>1.0000
**29**	1.03529<br>0.3020	1.056636<br>0.2921	1.067643<br>0.2872	0<br>1.0000	0<br>1.0000	0<br>1.0000	0<br>1.0000	0<br>1.0000	2.16924<br>0.0314	1.03529<br>0.3020	0<br>1.0000

The SAS System

The GLM Procedure
Least Squares Means

Least Squares Means for Effect Day*Device
t for H0: LSMean(i)=LSMean(j) / Pr > |t|

Dependent Variable: SR

i/j	67	68	69	70	71	72	73	74	75	76	77
**30**	1.03529<br>0.3020	1.056636<br>0.2921	1.067643<br>0.2872	0<br>1.0000	0<br>1.0000	0<br>1.0000	0<br>1.0000	0<br>1.0000	2.16924<br>0.0314	1.03529<br>0.3020	0<br>1.0000
**31**	1.03529<br>0.3020	1.056636<br>0.2921	1.067643<br>0.2872	0<br>1.0000	0<br>1.0000	0<br>1.0000	0<br>1.0000	0<br>1.0000	2.16924<br>0.0314	1.03529<br>0.3020	0<br>1.0000
**32**	1.03529<br>0.3020	1.056636<br>0.2921	1.067643<br>0.2872	0<br>1.0000	0<br>1.0000	0<br>1.0000	0<br>1.0000	0<br>1.0000	2.16924<br>0.0314	1.03529<br>0.3020	0<br>1.0000
**33**	1.03529<br>0.3020	1.056636<br>0.2921	1.067643<br>0.2872	0<br>1.0000	0<br>1.0000	0<br>1.0000	0<br>1.0000	0<br>1.0000	2.16924<br>0.0314	1.03529<br>0.3020	0<br>1.0000
**34**	1.03529<br>0.3020	1.056636<br>0.2921	1.067643<br>0.2872	0<br>1.0000	0<br>1.0000	0<br>1.0000	0<br>1.0000	0<br>1.0000	2.16924<br>0.0314	1.03529<br>0.3020	0<br>1.0000
**35**	1.03529<br>0.3020	1.056636<br>0.2921	1.067643<br>0.2872	0<br>1.0000	0<br>1.0000	0<br>1.0000	0<br>1.0000	0<br>1.0000	2.16924<br>0.0314	1.03529<br>0.3020	0<br>1.0000
**36**	-1.07798<br>0.2825	-1.05664<br>0.2921	-1.04563<br>0.2972	-2.11327<br>0.0360	-2.11327<br>0.0360	-2.11327<br>0.0360	-2.11327<br>0.0360	-2.11327<br>0.0360	0.055967<br>0.9554	-1.07798<br>0.2825	-2.11327<br>0.0360
**37**	1.03529<br>0.3020	1.056636<br>0.2921	1.067643<br>0.2872	0<br>1.0000	0<br>1.0000	0<br>1.0000	0<br>1.0000	0<br>1.0000	2.16924<br>0.0314	1.03529<br>0.3020	0<br>1.0000
**38**	1.03529<br>0.3020	1.056636<br>0.2921	1.067643<br>0.2872	0<br>1.0000	0<br>1.0000	0<br>1.0000	0<br>1.0000	0<br>1.0000	2.16924<br>0.0314	1.03529<br>0.3020	0<br>1.0000
**39**	-1.1<br>0.2729	-1.07865<br>0.2822	-1.06764<br>0.2872	-2.13529<br>0.0341	-2.13529<br>0.0341	-2.13529<br>0.0341	-2.13529<br>0.0341	-2.13529<br>0.0341	0.033954<br>0.9730	-1.1<br>0.2729	-2.13529<br>0.0341
**40**	0.010353<br>0.9918	0.031699<br>0.9747	0.042706<br>0.9660	-1.02494<br>0.3068	-1.02494<br>0.3068	-1.02494<br>0.3068	-1.02494<br>0.3068	-1.02494<br>0.3068	1.144303<br>0.2541	0.010353<br>0.9918	-1.02494<br>0.3068
**41**	-0.02135<br>0.9830	0<br>1.0000	0.011007<br>0.9912	-1.05664<br>0.2921	-1.05664<br>0.2921	-1.05664<br>0.2921	-1.05664<br>0.2921	-1.05664<br>0.2921	1.112604<br>0.2674	-0.02135<br>0.9830	-1.05664<br>0.2921
**42**	1.03529<br>0.3020	1.056636<br>0.2921	1.067643<br>0.2872	0<br>1.0000	0<br>1.0000	0<br>1.0000	0<br>1.0000	0<br>1.0000	2.16924<br>0.0314	1.03529<br>0.3020	0<br>1.0000
**43**	1.03529<br>0.3020	1.056636<br>0.2921	1.067643<br>0.2872	0<br>1.0000	0<br>1.0000	0<br>1.0000	0<br>1.0000	0<br>1.0000	2.16924<br>0.0314	1.03529<br>0.3020	0<br>1.0000
**44**	1.03529<br>0.3020	1.056636<br>0.2921	1.067643<br>0.2872	0<br>1.0000	0<br>1.0000	0<br>1.0000	0<br>1.0000	0<br>1.0000	2.16924<br>0.0314	1.03529<br>0.3020	0<br>1.0000
**45**	-0.02135<br>0.9830	0<br>1.0000	0.011007<br>0.9912	-1.05664<br>0.2921	-1.05664<br>0.2921	-1.05664<br>0.2921	-1.05664<br>0.2921	-1.05664<br>0.2921	1.112604<br>0.2674	-0.02135<br>0.9830	-1.05664<br>0.2921
**46**	1.03529<br>0.3020	1.056636<br>0.2921	1.067643<br>0.2872	0<br>1.0000	0<br>1.0000	0<br>1.0000	0<br>1.0000	0<br>1.0000	2.16924<br>0.0314	1.03529<br>0.3020	0<br>1.0000
**47**	1.03529<br>0.3020	1.056636<br>0.2921	1.067643<br>0.2872	0<br>1.0000	0<br>1.0000	0<br>1.0000	0<br>1.0000	0<br>1.0000	2.16924<br>0.0314	1.03529<br>0.3020	0<br>1.0000
**48**	1.03529<br>0.3020	1.056636<br>0.2921	1.067643<br>0.2872	0<br>1.0000	0<br>1.0000	0<br>1.0000	0<br>1.0000	0<br>1.0000	2.16924<br>0.0314	1.03529<br>0.3020	0<br>1.0000

The SAS System

The GLM Procedure
Least Squares Means

Least Squares Means for Effect Day*Device
t for H0: LSMean(i)=LSMean(j) / Pr > |t|

Dependent Variable: SR

i/j	67	68	69	70	71	72	73	74	75	76	77
49	1.03529<br>0.3020	1.056636<br>0.2921	1.067643<br>0.2872	0<br>1.0000	0<br>1.0000	0<br>1.0000	0<br>1.0000	0<br>1.0000	2.16924<br>0.0314	1.03529<br>0.3020	0<br>1.0000
50	1.03529<br>0.3020	1.056636<br>0.2921	1.067643<br>0.2872	0<br>1.0000	0<br>1.0000	0<br>1.0000	0<br>1.0000	0<br>1.0000	2.16924<br>0.0314	1.03529<br>0.3020	0<br>1.0000
51	1.03529<br>0.3020	1.056636<br>0.2921	1.067643<br>0.2872	0<br>1.0000	0<br>1.0000	0<br>1.0000	0<br>1.0000	0<br>1.0000	2.16924<br>0.0314	1.03529<br>0.3020	0<br>1.0000
52	1.03529<br>0.3020	1.056636<br>0.2921	1.067643<br>0.2872	0<br>1.0000	0<br>1.0000	0<br>1.0000	0<br>1.0000	0<br>1.0000	2.16924<br>0.0314	1.03529<br>0.3020	0<br>1.0000
53	1.03529<br>0.3020	1.056636<br>0.2921	1.067643<br>0.2872	0<br>1.0000	0<br>1.0000	0<br>1.0000	0<br>1.0000	0<br>1.0000	2.16924<br>0.0314	1.03529<br>0.3020	0<br>1.0000
54	-0.04359<br>0.9653	-0.02224<br>0.9823	-0.01124<br>0.9910	-1.07888<br>0.2821	-1.07888<br>0.2821	-1.07888<br>0.2821	-1.07888<br>0.2821	-1.07888<br>0.2821	1.090359<br>0.2771	-0.04359<br>0.9653	-1.07888<br>0.2821
55	1.03529<br>0.3020	1.056636<br>0.2921	1.067643<br>0.2872	0<br>1.0000	0<br>1.0000	0<br>1.0000	0<br>1.0000	0<br>1.0000	2.16924<br>0.0314	1.03529<br>0.3020	0<br>1.0000
56	1.03529<br>0.3020	1.056636<br>0.2921	1.067643<br>0.2872	0<br>1.0000	0<br>1.0000	0<br>1.0000	0<br>1.0000	0<br>1.0000	2.16924<br>0.0314	1.03529<br>0.3020	0<br>1.0000
57	1.03529<br>0.3020	1.056636<br>0.2921	1.067643<br>0.2872	0<br>1.0000	0<br>1.0000	0<br>1.0000	0<br>1.0000	0<br>1.0000	2.16924<br>0.0314	1.03529<br>0.3020	0<br>1.0000
58	1.03529<br>0.3020	1.056636<br>0.2921	1.067643<br>0.2872	0<br>1.0000	0<br>1.0000	0<br>1.0000	0<br>1.0000	0<br>1.0000	2.16924<br>0.0314	1.03529<br>0.3020	0<br>1.0000
59	1.03529<br>0.3020	1.056636<br>0.2921	1.067643<br>0.2872	0<br>1.0000	0<br>1.0000	0<br>1.0000	0<br>1.0000	0<br>1.0000	2.16924<br>0.0314	1.03529<br>0.3020	0<br>1.0000
60	1.03529<br>0.3020	1.056636<br>0.2921	1.067643<br>0.2872	0<br>1.0000	0<br>1.0000	0<br>1.0000	0<br>1.0000	0<br>1.0000	2.16924<br>0.0314	1.03529<br>0.3020	0<br>1.0000
61	1.03529<br>0.3020	1.056636<br>0.2921	1.067643<br>0.2872	0<br>1.0000	0<br>1.0000	0<br>1.0000	0<br>1.0000	0<br>1.0000	2.16924<br>0.0314	1.03529<br>0.3020	0<br>1.0000
62	1.03529<br>0.3020	1.056636<br>0.2921	1.067643<br>0.2872	0<br>1.0000	0<br>1.0000	0<br>1.0000	0<br>1.0000	0<br>1.0000	2.16924<br>0.0314	1.03529<br>0.3020	0<br>1.0000
63	1.03529<br>0.3020	1.056636<br>0.2921	1.067643<br>0.2872	0<br>1.0000	0<br>1.0000	0<br>1.0000	0<br>1.0000	0<br>1.0000	2.16924<br>0.0314	1.03529<br>0.3020	0<br>1.0000
64	1.03529<br>0.3020	1.056636<br>0.2921	1.067643<br>0.2872	0<br>1.0000	0<br>1.0000	0<br>1.0000	0<br>1.0000	0<br>1.0000	2.16924<br>0.0314	1.03529<br>0.3020	0<br>1.0000
65	1.03529<br>0.3020	1.056636<br>0.2921	1.067643<br>0.2872	0<br>1.0000	0<br>1.0000	0<br>1.0000	0<br>1.0000	0<br>1.0000	2.16924<br>0.0314	1.03529<br>0.3020	0<br>1.0000
66	1.03529<br>0.3020	1.056636<br>0.2921	1.067643<br>0.2872	0<br>1.0000	0<br>1.0000	0<br>1.0000	0<br>1.0000	0<br>1.0000	2.16924<br>0.0314	1.03529<br>0.3020	0<br>1.0000
67		0.021346<br>0.9830	0.032353<br>0.9742	-1.03529<br>0.3020	-1.03529<br>0.3020	-1.03529<br>0.3020	-1.03529<br>0.3020	-1.03529<br>0.3020	1.13395<br>0.2584	0<br>1.0000	-1.03529<br>0.3020

The SAS System

The GLM Procedure
Least Squares Means

Least Squares Means for Effect Day*Device<br>t for H0: LSMean(i)=LSMean(j) / Pr > |t| <br>Dependent Variable: SR											
i/j	67	68	69	70	71	72	73	74	75	76	77
68	-0.02135<br>0.9830		0.011007<br>0.9912	-1.05664<br>0.2921	-1.05664<br>0.2921	-1.05664<br>0.2921	-1.05664<br>0.2921	-1.05664<br>0.2921	1.112604<br>0.2674	-0.02135<br>0.9830	-1.05664<br>0.2921
69	-0.03235<br>0.9742	-0.01101<br>0.9912		-1.06764<br>0.2872	-1.06764<br>0.2872	-1.06764<br>0.2872	-1.06764<br>0.2872	-1.06764<br>0.2872	1.101597<br>0.2722	-0.03235<br>0.9742	-1.06764<br>0.2872
70	1.03529<br>0.3020	1.056636<br>0.2921	1.067643<br>0.2872		0<br>1.0000	0<br>1.0000	0<br>1.0000	0<br>1.0000	2.16924<br>0.0314	1.03529<br>0.3020	0<br>1.0000
71	1.03529<br>0.3020	1.056636<br>0.2921	1.067643<br>0.2872	0<br>1.0000		0<br>1.0000	0<br>1.0000	0<br>1.0000	2.16924<br>0.0314	1.03529<br>0.3020	0<br>1.0000
72	1.03529<br>0.3020	1.056636<br>0.2921	1.067643<br>0.2872	0<br>1.0000	0<br>1.0000		0<br>1.0000	0<br>1.0000	2.16924<br>0.0314	1.03529<br>0.3020	0<br>1.0000
73	1.03529<br>0.3020	1.056636<br>0.2921	1.067643<br>0.2872	0<br>1.0000	0<br>1.0000	0<br>1.0000		0<br>1.0000	2.16924<br>0.0314	1.03529<br>0.3020	0<br>1.0000
74	1.03529<br>0.3020	1.056636<br>0.2921	1.067643<br>0.2872	0<br>1.0000	0<br>1.0000	0<br>1.0000	0<br>1.0000		2.16924<br>0.0314	1.03529<br>0.3020	0<br>1.0000
75	-1.13395<br>0.2584	-1.1126<br>0.2674	-1.1016<br>0.2722	-2.16924<br>0.0314	-2.16924<br>0.0314	-2.16924<br>0.0314	-2.16924<br>0.0314	-2.16924<br>0.0314		-1.13395<br>0.2584	-2.16924<br>0.0314
76	0<br>1.0000	0.021346<br>0.9830	0.032353<br>0.9742	-1.03529<br>0.3020	-1.03529<br>0.3020	-1.03529<br>0.3020	-1.03529<br>0.3020	-1.03529<br>0.3020	1.13395<br>0.2584		-1.03529<br>0.3020
77	1.03529<br>0.3020	1.056636<br>0.2921	1.067643<br>0.2872	0<br>1.0000	0<br>1.0000	0<br>1.0000	0<br>1.0000	0<br>1.0000	2.16924<br>0.0314	1.03529<br>0.3020	
78	-0.05507<br>0.9561	-0.03372<br>0.9731	-0.02272<br>0.9819	-1.09036<br>0.2771	-1.09036<br>0.2771	-1.09036<br>0.2771	-1.09036<br>0.2771	-1.09036<br>0.2771	1.078881<br>0.2821	-0.05507<br>0.9561	-1.09036<br>0.2771
79	1.03529<br>0.3020	1.056636<br>0.2921	1.067643<br>0.2872	0<br>1.0000	0<br>1.0000	0<br>1.0000	0<br>1.0000	0<br>1.0000	2.16924<br>0.0314	1.03529<br>0.3020	0<br>1.0000
80	1.03529<br>0.3020	1.056636<br>0.2921	1.067643<br>0.2872	0<br>1.0000	0<br>1.0000	0<br>1.0000	0<br>1.0000	0<br>1.0000	2.16924<br>0.0314	1.03529<br>0.3020	0<br>1.0000
81	1.03529<br>0.3020	1.056636<br>0.2921	1.067643<br>0.2872	0<br>1.0000	0<br>1.0000	0<br>1.0000	0<br>1.0000	0<br>1.0000	2.16924<br>0.0314	1.03529<br>0.3020	0<br>1.0000
82	1.03529<br>0.3020	1.056636<br>0.2921	1.067643<br>0.2872	0<br>1.0000	0<br>1.0000	0<br>1.0000	0<br>1.0000	0<br>1.0000	2.16924<br>0.0314	1.03529<br>0.3020	0<br>1.0000
83	-0.03235<br>0.9742	-0.01101<br>0.9912	0<br>1.0000	-1.06764<br>0.2872	-1.06764<br>0.2872	-1.06764<br>0.2872	-1.06764<br>0.2872	-1.06764<br>0.2872	1.101597<br>0.2722	-0.03235<br>0.9742	-1.06764<br>0.2872
84	-0.06679<br>0.9468	-0.04545<br>0.9638	-0.03444<br>0.9726	-1.10208<br>0.2719	-1.10208<br>0.2719	-1.10208<br>0.2719	-1.10208<br>0.2719	-1.10208<br>0.2719	1.067157<br>0.2874	-0.06679<br>0.9468	-1.10208<br>0.2719
85	-0.01056<br>0.9916	0.010782<br>0.9914	0.021789<br>0.9826	-1.04585<br>0.2971	-1.04585<br>0.2971	-1.04585<br>0.2971	-1.04585<br>0.2971	-1.04585<br>0.2971	1.123386<br>0.2628	-0.01056<br>0.9916	-1.04585<br>0.2971

The SAS System

The GLM Procedure
Least Squares Means

Least Squares Means for Effect Day*Device
t for H0: LSMean(i)=LSMean(j) / Pr > |t|

Dependent Variable: SR

i/j	67	68	69	70	71	72	73	74	75	76	77
**86**	1.03529<br>0.3020	1.056636<br>0.2921	1.067643<br>0.2872	0<br>1.0000	0<br>1.0000	0<br>1.0000	0<br>1.0000	0<br>1.0000	2.16924<br>0.0314	1.03529<br>0.3020	0<br>1.0000
**87**	-1.19283<br>0.2346	-1.17149<br>0.2430	-1.16048<br>0.2474	-2.22812<br>0.0272	-2.22812<br>0.0272	-2.22812<br>0.0272	-2.22812<br>0.0272	-2.22812<br>0.0272	-0.05888<br>0.9531	-1.19283<br>0.2346	-2.22812<br>0.0272

Least Squares Means for Effect Day*Device
t for H0: LSMean(i)=LSMean(j) / Pr > |t|

Dependent Variable: SR

i/j	78	79	80	81	82	83	84	85	86	87
**1**	1.090359<br>0.2771	0<br>1.0000	0<br>1.0000	0<br>1.0000	0<br>1.0000	1.067643<br>0.2872	1.102083<br>0.2719	1.045854<br>0.2971	0<br>1.0000	2.228124<br>0.0272
**2**	1.090359<br>0.2771	0<br>1.0000	0<br>1.0000	0<br>1.0000	0<br>1.0000	1.067643<br>0.2872	1.102083<br>0.2719	1.045854<br>0.2971	0<br>1.0000	2.228124<br>0.0272
**3**	1.090359<br>0.2771	0<br>1.0000	0<br>1.0000	0<br>1.0000	0<br>1.0000	1.067643<br>0.2872	1.102083<br>0.2719	1.045854<br>0.2971	0<br>1.0000	2.228124<br>0.0272
**4**	1.090359<br>0.2771	0<br>1.0000	0<br>1.0000	0<br>1.0000	0<br>1.0000	1.067643<br>0.2872	1.102083<br>0.2719	1.045854<br>0.2971	0<br>1.0000	2.228124<br>0.0272
**5**	1.090359<br>0.2771	0<br>1.0000	0<br>1.0000	0<br>1.0000	0<br>1.0000	1.067643<br>0.2872	1.102083<br>0.2719	1.045854<br>0.2971	0<br>1.0000	2.228124<br>0.0272
**6**	0.065422<br>0.9479	-1.02494<br>0.3068	-1.02494<br>0.3068	-1.02494<br>0.3068	-1.02494<br>0.3068	0.042706<br>0.9660	0.077146<br>0.9386	0.020917<br>0.9833	-1.02494<br>0.3068	1.203187<br>0.2305
**7**	1.090359<br>0.2771	0<br>1.0000	0<br>1.0000	0<br>1.0000	0<br>1.0000	1.067643<br>0.2872	1.102083<br>0.2719	1.045854<br>0.2971	0<br>1.0000	2.228124<br>0.0272
**8**	1.090359<br>0.2771	0<br>1.0000	0<br>1.0000	0<br>1.0000	0<br>1.0000	1.067643<br>0.2872	1.102083<br>0.2719	1.045854<br>0.2971	0<br>1.0000	2.228124<br>0.0272
**9**	1.090359<br>0.2771	0<br>1.0000	0<br>1.0000	0<br>1.0000	0<br>1.0000	1.067643<br>0.2872	1.102083<br>0.2719	1.045854<br>0.2971	0<br>1.0000	2.228124<br>0.0272
**10**	-0.95952<br>0.3386	-2.04987<br>0.0419	-2.04987<br>0.0419	-2.04987<br>0.0419	-2.04987<br>0.0419	-0.98223<br>0.3273	-0.94779<br>0.3446	-1.00402<br>0.3168	-2.04987<br>0.0419	0.17825<br>0.8587
**11**	-4.03433<br><.0001	-5.12469<br><.0001	-5.12469<br><.0001	-5.12469<br><.0001	-5.12469<br><.0001	-4.05704<br><.0001	-4.0226<br><.0001	-4.07883<br><.0001	-5.12469<br><.0001	-2.89656<br>0.0043
**12**	-3.04045<br>0.0027	-4.13081<br><.0001	-4.13081<br><.0001	-4.13081<br><.0001	-4.13081<br><.0001	-3.06316<br>0.0025	-3.02872<br>0.0028	-3.08495<br>0.0024	-4.13081<br><.0001	-1.90268<br>0.0587
**13**	0.065422<br>0.9479	-1.02494<br>0.3068	-1.02494<br>0.3068	-1.02494<br>0.3068	-1.02494<br>0.3068	0.042706<br>0.9660	0.077146<br>0.9386	0.020917<br>0.9833	-1.02494<br>0.3068	1.203187<br>0.2305
**14**	-3.07215<br>0.0025	-4.16251<br><.0001	-4.16251<br><.0001	-4.16251<br><.0001	-4.16251<br><.0001	-3.09486<br>0.0023	-3.06042<br>0.0026	-3.11665<br>0.0021	-4.16251<br><.0001	-1.93438<br>0.0547

The SAS System

The GLM Procedure
Least Squares Means

Least Squares Means for Effect Day*Device
t for H0: LSMean(i)=LSMean(j) / Pr > |t|

Dependent Variable: SR

i/j	78	79	80	81	82	83	84	85	86	87
15	-2.04742<br>0.0421	-3.13778<br>0.0020	-3.13778<br>0.0020	-3.13778<br>0.0020	-3.13778<br>0.0020	-2.07014<br>0.0399	-2.0357<br>0.0433	-2.09193<br>0.0379	-3.13778<br>0.0020	-0.90966<br>0.3643
16	1.090359<br>0.2771	0<br>1.0000	0<br>1.0000	0<br>1.0000	0<br>1.0000	1.067643<br>0.2872	1.102083<br>0.2719	1.045854<br>0.2971	0<br>1.0000	2.228124<br>0.0272
17	1.090359<br>0.2771	0<br>1.0000	0<br>1.0000	0<br>1.0000	0<br>1.0000	1.067643<br>0.2872	1.102083<br>0.2719	1.045854<br>0.2971	0<br>1.0000	2.228124<br>0.0272
18	0.044504<br>0.9646	-1.04585<br>0.2971	-1.04585<br>0.2971	-1.04585<br>0.2971	-1.04585<br>0.2971	0.021789<br>0.9826	0.056229<br>0.9552	0<br>1.0000	-1.04585<br>0.2971	1.18227<br>0.2387
19	1.090359<br>0.2771	0<br>1.0000	0<br>1.0000	0<br>1.0000	0<br>1.0000	1.067643<br>0.2872	1.102083<br>0.2719	1.045854<br>0.2971	0<br>1.0000	2.228124<br>0.0272
20	-1.02314<br>0.3077	-2.1135<br>0.0360	-2.1135<br>0.0360	-2.1135<br>0.0360	-2.1135<br>0.0360	-1.04585<br>0.2971	-1.01141<br>0.3132	-1.06764<br>0.2872	-2.1135<br>0.0360	0.114627<br>0.9089
21	1.090359<br>0.2771	0<br>1.0000	0<br>1.0000	0<br>1.0000	0<br>1.0000	1.067643<br>0.2872	1.102083<br>0.2719	1.045854<br>0.2971	0<br>1.0000	2.228124<br>0.0272
22	1.090359<br>0.2771	0<br>1.0000	0<br>1.0000	0<br>1.0000	0<br>1.0000	1.067643<br>0.2872	1.102083<br>0.2719	1.045854<br>0.2971	0<br>1.0000	2.228124<br>0.0272
23	1.090359<br>0.2771	0<br>1.0000	0<br>1.0000	0<br>1.0000	0<br>1.0000	1.067643<br>0.2872	1.102083<br>0.2719	1.045854<br>0.2971	0<br>1.0000	2.228124<br>0.0272
24	1.090359<br>0.2771	0<br>1.0000	0<br>1.0000	0<br>1.0000	0<br>1.0000	1.067643<br>0.2872	1.102083<br>0.2719	1.045854<br>0.2971	0<br>1.0000	2.228124<br>0.0272
25	1.090359<br>0.2771	0<br>1.0000	0<br>1.0000	0<br>1.0000	0<br>1.0000	1.067643<br>0.2872	1.102083<br>0.2719	1.045854<br>0.2971	0<br>1.0000	2.228124<br>0.0272
26	1.090359<br>0.2771	0<br>1.0000	0<br>1.0000	0<br>1.0000	0<br>1.0000	1.067643<br>0.2872	1.102083<br>0.2719	1.045854<br>0.2971	0<br>1.0000	2.228124<br>0.0272
27	1.090359<br>0.2771	0<br>1.0000	0<br>1.0000	0<br>1.0000	0<br>1.0000	1.067643<br>0.2872	1.102083<br>0.2719	1.045854<br>0.2971	0<br>1.0000	2.228124<br>0.0272
28	1.090359<br>0.2771	0<br>1.0000	0<br>1.0000	0<br>1.0000	0<br>1.0000	1.067643<br>0.2872	1.102083<br>0.2719	1.045854<br>0.2971	0<br>1.0000	2.228124<br>0.0272
29	1.090359<br>0.2771	0<br>1.0000	0<br>1.0000	0<br>1.0000	0<br>1.0000	1.067643<br>0.2872	1.102083<br>0.2719	1.045854<br>0.2971	0<br>1.0000	2.228124<br>0.0272
30	1.090359<br>0.2771	0<br>1.0000	0<br>1.0000	0<br>1.0000	0<br>1.0000	1.067643<br>0.2872	1.102083<br>0.2719	1.045854<br>0.2971	0<br>1.0000	2.228124<br>0.0272
31	1.090359<br>0.2771	0<br>1.0000	0<br>1.0000	0<br>1.0000	0<br>1.0000	1.067643<br>0.2872	1.102083<br>0.2719	1.045854<br>0.2971	0<br>1.0000	2.228124<br>0.0272
32	1.090359<br>0.2771	0<br>1.0000	0<br>1.0000	0<br>1.0000	0<br>1.0000	1.067643<br>0.2872	1.102083<br>0.2719	1.045854<br>0.2971	0<br>1.0000	2.228124<br>0.0272
33	1.090359<br>0.2771	0<br>1.0000	0<br>1.0000	0<br>1.0000	0<br>1.0000	1.067643<br>0.2872	1.102083<br>0.2719	1.045854<br>0.2971	0<br>1.0000	2.228124<br>0.0272

The SAS System

The GLM Procedure
Least Squares Means

Least Squares Means for Effect Day*Device
t for H0: LSMean(i)=LSMean(j) / Pr > |t|

Dependent Variable: SR

i/j	78	79	80	81	82	83	84	85	86	87
34	1.090359<br>0.2771	0<br>1.0000	0<br>1.0000	0<br>1.0000	0<br>1.0000	1.067643<br>0.2872	1.102083<br>0.2719	1.045854<br>0.2971	0<br>1.0000	2.228124<br>0.0272
35	1.090359<br>0.2771	0<br>1.0000	0<br>1.0000	0<br>1.0000	0<br>1.0000	1.067643<br>0.2872	1.102083<br>0.2719	1.045854<br>0.2971	0<br>1.0000	2.228124<br>0.0272
36	-1.02291<br>0.3078	-2.11327<br>0.0360	-2.11327<br>0.0360	-2.11327<br>0.0360	-2.11327<br>0.0360	-1.04563<br>0.2972	-1.01119<br>0.3133	-1.06742<br>0.2873	-2.11327<br>0.0360	0.114852<br>0.9087
37	1.090359<br>0.2771	0<br>1.0000	0<br>1.0000	0<br>1.0000	0<br>1.0000	1.067643<br>0.2872	1.102083<br>0.2719	1.045854<br>0.2971	0<br>1.0000	2.228124<br>0.0272
38	1.090359<br>0.2771	0<br>1.0000	0<br>1.0000	0<br>1.0000	0<br>1.0000	1.067643<br>0.2872	1.102083<br>0.2719	1.045854<br>0.2971	0<br>1.0000	2.228124<br>0.0272
39	-1.04493<br>0.2975	-2.13529<br>0.0341	-2.13529<br>0.0341	-2.13529<br>0.0341	-2.13529<br>0.0341	-1.06764<br>0.2872	-1.0332<br>0.3029	-1.08943<br>0.2775	-2.13529<br>0.0341	0.092839<br>0.9261
40	0.065422<br>0.9479	-1.02494<br>0.3068	-1.02494<br>0.3068	-1.02494<br>0.3068	-1.02494<br>0.3068	0.042706<br>0.9660	0.077146<br>0.9386	0.020917<br>0.9833	-1.02494<br>0.3068	1.203187<br>0.2305
41	0.033722<br>0.9731	-1.05664<br>0.2921	-1.05664<br>0.2921	-1.05664<br>0.2921	-1.05664<br>0.2921	0.011007<br>0.9912	0.045447<br>0.9638	-0.01078<br>0.9914	-1.05664<br>0.2921	1.171488<br>0.2430
42	1.090359<br>0.2771	0<br>1.0000	0<br>1.0000	0<br>1.0000	0<br>1.0000	1.067643<br>0.2872	1.102083<br>0.2719	1.045854<br>0.2971	0<br>1.0000	2.228124<br>0.0272
43	1.090359<br>0.2771	0<br>1.0000	0<br>1.0000	0<br>1.0000	0<br>1.0000	1.067643<br>0.2872	1.102083<br>0.2719	1.045854<br>0.2971	0<br>1.0000	2.228124<br>0.0272
44	1.090359<br>0.2771	0<br>1.0000	0<br>1.0000	0<br>1.0000	0<br>1.0000	1.067643<br>0.2872	1.102083<br>0.2719	1.045854<br>0.2971	0<br>1.0000	2.228124<br>0.0272
45	0.033722<br>0.9731	-1.05664<br>0.2921	-1.05664<br>0.2921	-1.05664<br>0.2921	-1.05664<br>0.2921	0.011007<br>0.9912	0.045447<br>0.9638	-0.01078<br>0.9914	-1.05664<br>0.2921	1.171488<br>0.2430
46	1.090359<br>0.2771	0<br>1.0000	0<br>1.0000	0<br>1.0000	0<br>1.0000	1.067643<br>0.2872	1.102083<br>0.2719	1.045854<br>0.2971	0<br>1.0000	2.228124<br>0.0272
47	1.090359<br>0.2771	0<br>1.0000	0<br>1.0000	0<br>1.0000	0<br>1.0000	1.067643<br>0.2872	1.102083<br>0.2719	1.045854<br>0.2971	0<br>1.0000	2.228124<br>0.0272
48	1.090359<br>0.2771	0<br>1.0000	0<br>1.0000	0<br>1.0000	0<br>1.0000	1.067643<br>0.2872	1.102083<br>0.2719	1.045854<br>0.2971	0<br>1.0000	2.228124<br>0.0272
49	1.090359<br>0.2771	0<br>1.0000	0<br>1.0000	0<br>1.0000	0<br>1.0000	1.067643<br>0.2872	1.102083<br>0.2719	1.045854<br>0.2971	0<br>1.0000	2.228124<br>0.0272
50	1.090359<br>0.2771	0<br>1.0000	0<br>1.0000	0<br>1.0000	0<br>1.0000	1.067643<br>0.2872	1.102083<br>0.2719	1.045854<br>0.2971	0<br>1.0000	2.228124<br>0.0272
51	1.090359<br>0.2771	0<br>1.0000	0<br>1.0000	0<br>1.0000	0<br>1.0000	1.067643<br>0.2872	1.102083<br>0.2719	1.045854<br>0.2971	0<br>1.0000	2.228124<br>0.0272
52	1.090359<br>0.2771	0<br>1.0000	0<br>1.0000	0<br>1.0000	0<br>1.0000	1.067643<br>0.2872	1.102083<br>0.2719	1.045854<br>0.2971	0<br>1.0000	2.228124<br>0.0272

The SAS System

The GLM Procedure
Least Squares Means

Least Squares Means for Effect Day*Device
t for H0: LSMean(i)=LSMean(j) / Pr > |t|

Dependent Variable: SR

i/j	78	79	80	81	82	83	84	85	86	87
**53**	1.090359<br>0.2771	0<br>1.0000	0<br>1.0000	0<br>1.0000	0<br>1.0000	1.067643<br>0.2872	1.102083<br>0.2719	1.045854<br>0.2971	0<br>1.0000	2.228124<br>0.0272
**54**	0.011477<br>0.9909	-1.07888<br>0.2821	-1.07888<br>0.2821	-1.07888<br>0.2821	-1.07888<br>0.2821	-0.01124<br>0.9910	0.023202<br>0.9815	-0.03303<br>0.9737	-1.07888<br>0.2821	1.149243<br>0.2520
**55**	1.090359<br>0.2771	0<br>1.0000	0<br>1.0000	0<br>1.0000	0<br>1.0000	1.067643<br>0.2872	1.102083<br>0.2719	1.045854<br>0.2971	0<br>1.0000	2.228124<br>0.0272
**56**	1.090359<br>0.2771	0<br>1.0000	0<br>1.0000	0<br>1.0000	0<br>1.0000	1.067643<br>0.2872	1.102083<br>0.2719	1.045854<br>0.2971	0<br>1.0000	2.228124<br>0.0272
**57**	1.090359<br>0.2771	0<br>1.0000	0<br>1.0000	0<br>1.0000	0<br>1.0000	1.067643<br>0.2872	1.102083<br>0.2719	1.045854<br>0.2971	0<br>1.0000	2.228124<br>0.0272
**58**	1.090359<br>0.2771	0<br>1.0000	0<br>1.0000	0<br>1.0000	0<br>1.0000	1.067643<br>0.2872	1.102083<br>0.2719	1.045854<br>0.2971	0<br>1.0000	2.228124<br>0.0272
**59**	1.090359<br>0.2771	0<br>1.0000	0<br>1.0000	0<br>1.0000	0<br>1.0000	1.067643<br>0.2872	1.102083<br>0.2719	1.045854<br>0.2971	0<br>1.0000	2.228124<br>0.0272
**60**	1.090359<br>0.2771	0<br>1.0000	0<br>1.0000	0<br>1.0000	0<br>1.0000	1.067643<br>0.2872	1.102083<br>0.2719	1.045854<br>0.2971	0<br>1.0000	2.228124<br>0.0272
**61**	1.090359<br>0.2771	0<br>1.0000	0<br>1.0000	0<br>1.0000	0<br>1.0000	1.067643<br>0.2872	1.102083<br>0.2719	1.045854<br>0.2971	0<br>1.0000	2.228124<br>0.0272
**62**	1.090359<br>0.2771	0<br>1.0000	0<br>1.0000	0<br>1.0000	0<br>1.0000	1.067643<br>0.2872	1.102083<br>0.2719	1.045854<br>0.2971	0<br>1.0000	2.228124<br>0.0272
**63**	1.090359<br>0.2771	0<br>1.0000	0<br>1.0000	0<br>1.0000	0<br>1.0000	1.067643<br>0.2872	1.102083<br>0.2719	1.045854<br>0.2971	0<br>1.0000	2.228124<br>0.0272
**64**	1.090359<br>0.2771	0<br>1.0000	0<br>1.0000	0<br>1.0000	0<br>1.0000	1.067643<br>0.2872	1.102083<br>0.2719	1.045854<br>0.2971	0<br>1.0000	2.228124<br>0.0272
**65**	1.090359<br>0.2771	0<br>1.0000	0<br>1.0000	0<br>1.0000	0<br>1.0000	1.067643<br>0.2872	1.102083<br>0.2719	1.045854<br>0.2971	0<br>1.0000	2.228124<br>0.0272
**66**	1.090359<br>0.2771	0<br>1.0000	0<br>1.0000	0<br>1.0000	0<br>1.0000	1.067643<br>0.2872	1.102083<br>0.2719	1.045854<br>0.2971	0<br>1.0000	2.228124<br>0.0272
**67**	0.055069<br>0.9561	-1.03529<br>0.3020	-1.03529<br>0.3020	-1.03529<br>0.3020	-1.03529<br>0.3020	0.032353<br>0.9742	0.066793<br>0.9468	0.010564<br>0.9916	-1.03529<br>0.3020	1.192834<br>0.2346
**68**	0.033722<br>0.9731	-1.05664<br>0.2921	-1.05664<br>0.2921	-1.05664<br>0.2921	-1.05664<br>0.2921	0.011007<br>0.9912	0.045447<br>0.9638	-0.01078<br>0.9914	-1.05664<br>0.2921	1.171488<br>0.2430
**69**	0.022716<br>0.9819	-1.06764<br>0.2872	-1.06764<br>0.2872	-1.06764<br>0.2872	-1.06764<br>0.2872	0<br>1.0000	0.03444<br>0.9726	-0.02179<br>0.9826	-1.06764<br>0.2872	1.160481<br>0.2474
**70**	1.090359<br>0.2771	0<br>1.0000	0<br>1.0000	0<br>1.0000	0<br>1.0000	1.067643<br>0.2872	1.102083<br>0.2719	1.045854<br>0.2971	0<br>1.0000	2.228124<br>0.0272
**71**	1.090359<br>0.2771	0<br>1.0000	0<br>1.0000	0<br>1.0000	0<br>1.0000	1.067643<br>0.2872	1.102083<br>0.2719	1.045854<br>0.2971	0<br>1.0000	2.228124<br>0.0272

The SAS System

The GLM Procedure
Least Squares Means

Least Squares Means for Effect Day*Device
t for H0: LSMean(i)=LSMean(j) / Pr > |t|

Dependent Variable: SR

i/j	78	79	80	81	82	83	84	85	86	87
**72**	1.090359<br>0.2771	0<br>1.0000	0<br>1.0000	0<br>1.0000	0<br>1.0000	1.067643<br>0.2872	1.102083<br>0.2719	1.045854<br>0.2971	0<br>1.0000	2.228124<br>0.0272
**73**	1.090359<br>0.2771	0<br>1.0000	0<br>1.0000	0<br>1.0000	0<br>1.0000	1.067643<br>0.2872	1.102083<br>0.2719	1.045854<br>0.2971	0<br>1.0000	2.228124<br>0.0272
**74**	1.090359<br>0.2771	0<br>1.0000	0<br>1.0000	0<br>1.0000	0<br>1.0000	1.067643<br>0.2872	1.102083<br>0.2719	1.045854<br>0.2971	0<br>1.0000	2.228124<br>0.0272
**75**	-1.07888<br>0.2821	-2.16924<br>0.0314	-2.16924<br>0.0314	-2.16924<br>0.0314	-2.16924<br>0.0314	-1.1016<br>0.2722	-1.06716<br>0.2874	-1.12339<br>0.2628	-2.16924<br>0.0314	0.058884<br>0.9531
**76**	0.055069<br>0.9561	-1.03529<br>0.3020	-1.03529<br>0.3020	-1.03529<br>0.3020	-1.03529<br>0.3020	0.032353<br>0.9742	0.066793<br>0.9468	0.010564<br>0.9916	-1.03529<br>0.3020	1.192834<br>0.2346
**77**	1.090359<br>0.2771	0<br>1.0000	0<br>1.0000	0<br>1.0000	0<br>1.0000	1.067643<br>0.2872	1.102083<br>0.2719	1.045854<br>0.2971	0<br>1.0000	2.228124<br>0.0272
**78**		-1.09036<br>0.2771	-1.09036<br>0.2771	-1.09036<br>0.2771	-1.09036<br>0.2771	-0.02272<br>0.9819	0.011724<br>0.9907	-0.0445<br>0.9646	-1.09036<br>0.2771	1.137766<br>0.2568
**79**	1.090359<br>0.2771		0<br>1.0000	0<br>1.0000	0<br>1.0000	1.067643<br>0.2872	1.102083<br>0.2719	1.045854<br>0.2971	0<br>1.0000	2.228124<br>0.0272
**80**	1.090359<br>0.2771	0<br>1.0000		0<br>1.0000	0<br>1.0000	1.067643<br>0.2872	1.102083<br>0.2719	1.045854<br>0.2971	0<br>1.0000	2.228124<br>0.0272
**81**	1.090359<br>0.2771	0<br>1.0000	0<br>1.0000		0<br>1.0000	1.067643<br>0.2872	1.102083<br>0.2719	1.045854<br>0.2971	0<br>1.0000	2.228124<br>0.0272
**82**	1.090359<br>0.2771	0<br>1.0000	0<br>1.0000	0<br>1.0000		1.067643<br>0.2872	1.102083<br>0.2719	1.045854<br>0.2971	0<br>1.0000	2.228124<br>0.0272
**83**	0.022716<br>0.9819	-1.06764<br>0.2872	-1.06764<br>0.2872	-1.06764<br>0.2872	-1.06764<br>0.2872		0.03444<br>0.9726	-0.02179<br>0.9826	-1.06764<br>0.2872	1.160481<br>0.2474
**84**	-0.01172<br>0.9907	-1.10208<br>0.2719	-1.10208<br>0.2719	-1.10208<br>0.2719	-1.10208<br>0.2719	-0.03444<br>0.9726		-0.05623<br>0.9552	-1.10208<br>0.2719	1.126041<br>0.2617
**85**	0.044504<br>0.9646	-1.04585<br>0.2971	-1.04585<br>0.2971	-1.04585<br>0.2971	-1.04585<br>0.2971	0.021789<br>0.9826	0.056229<br>0.9552		-1.04585<br>0.2971	1.18227<br>0.2387
**86**	1.090359<br>0.2771	0<br>1.0000	0<br>1.0000	0<br>1.0000	0<br>1.0000	1.067643<br>0.2872	1.102083<br>0.2719	1.045854<br>0.2971		2.228124<br>0.0272
**87**	-1.13777<br>0.2568	-2.22812<br>0.0272	-2.22812<br>0.0272	-2.22812<br>0.0272	-2.22812<br>0.0272	-1.16048<br>0.2474	-1.12604<br>0.2617	-1.18227<br>0.2387	-2.22812<br>0.0272	

The SAS System

The GLM Procedure
Least Squares Means

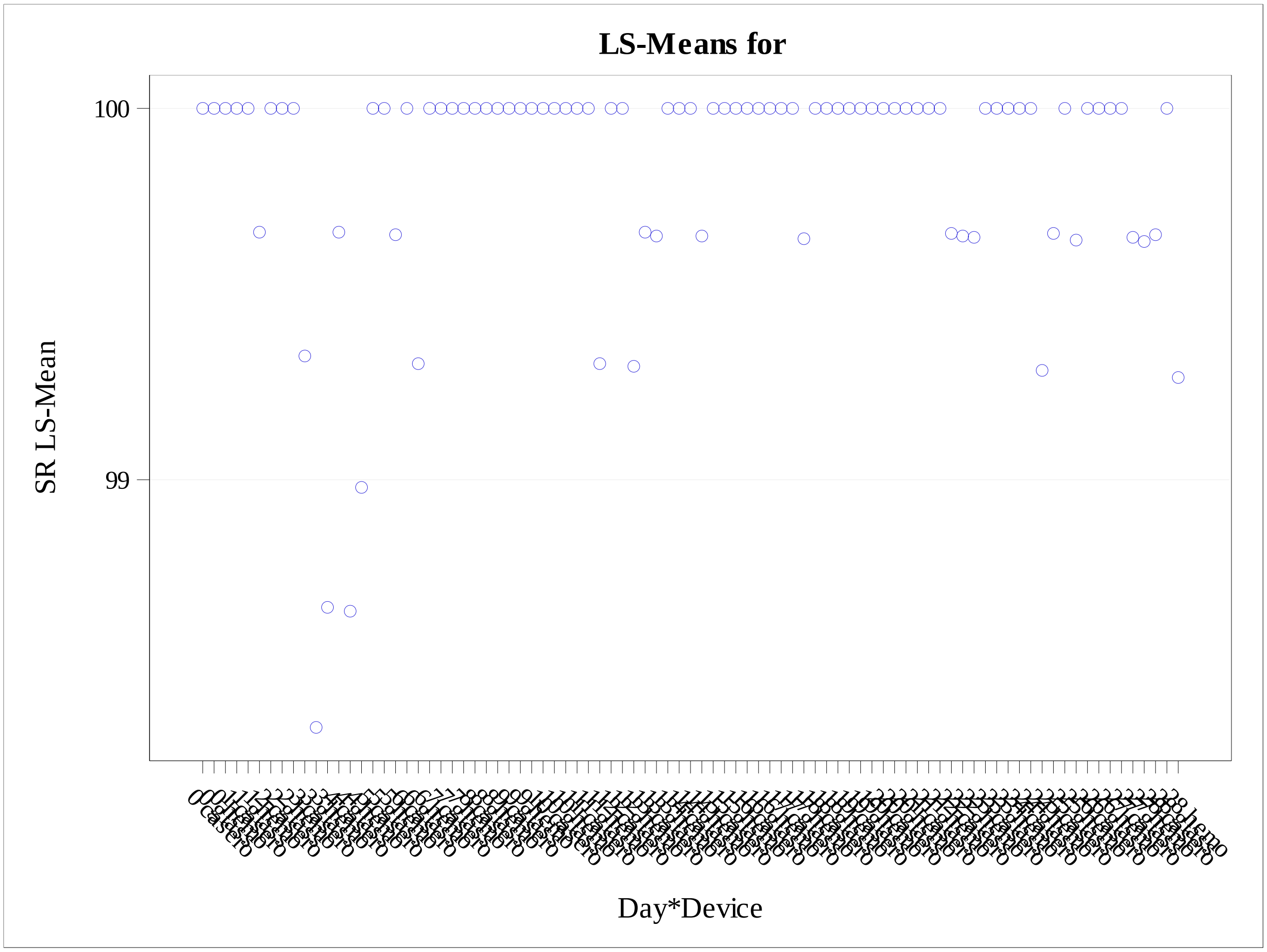

The SAS System

The GLM Procedure
Least Squares Means

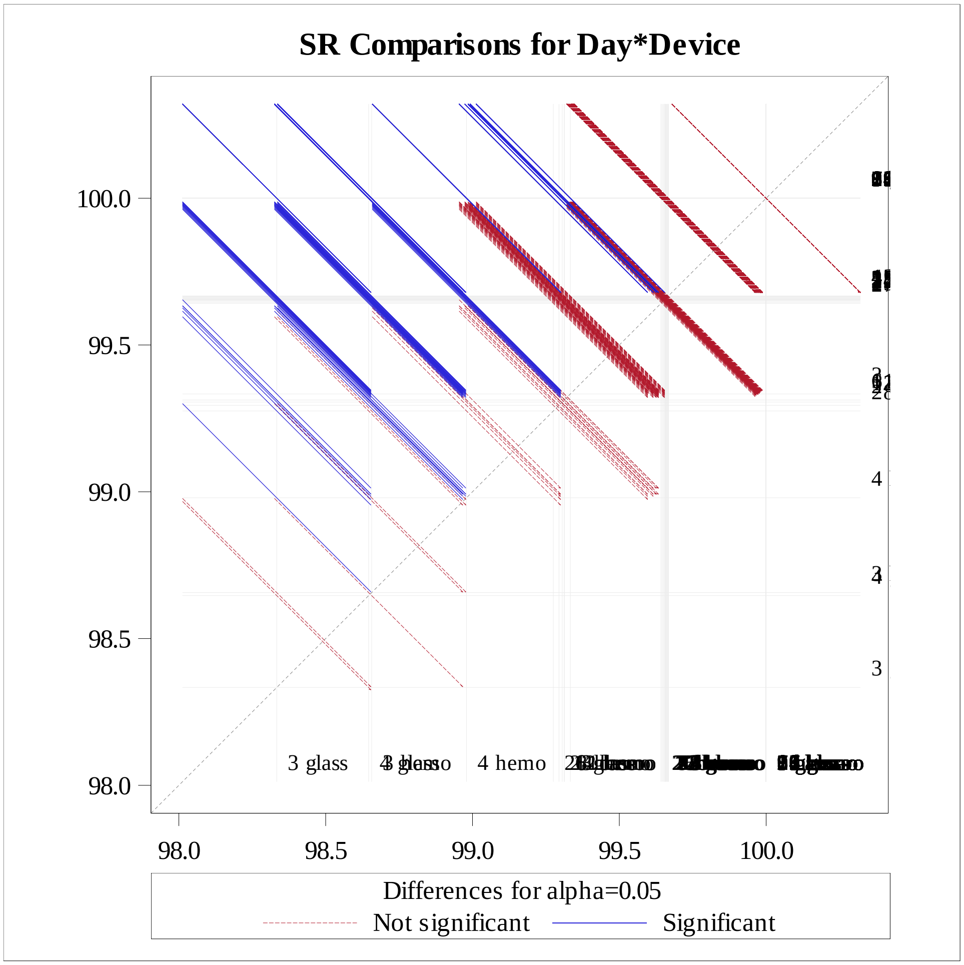

Note: To ensure overall protection level, only probabilities associated with pre-planned comparisons should be used.

The SAS System

The UNIVARIATE Procedure
Variable: RES

Moments			
**N**	261	**Sum Weights**	261
**Mean**	0	**Sum Observations**	0
**Std Deviation**	0.32584801	**Variance**	0.10617693
**Skewness**	-1.4984961	**Kurtosis**	5.6957599
**Uncorrected SS**	27.6060015	**Corrected SS**	27.6060015
**Coeff Variation**	.	**Std Error Mean**	0.02016949

Basic Statistical Measures			
**Location**		**Variability**	
**Mean**	0	**Std Deviation**	0.32585
**Median**	0	**Variance**	0.10618
**Mode**	0	**Range**	2.17391
		**Interquartile Range**	1.4211E-14

Tests for Location: Mu0=0				
**Test**	**Statistic**		**p Value**	
**Student's t**	**t**	0	**Pr > |t|**	1.0000
**Sign**	**M**	30	**Pr >= |M|**	<.0001
**Signed Rank**	**S**	810.5	**Pr >= |S|**	0.0206

Tests for Normality				
**Test**	**Statistic**		**p Value**	
**Shapiro-Wilk**	**W**	0.680851	**Pr < W**	<0.0001
**Kolmogorov-Smirnov**	**D**	0.396552	**Pr > D**	<0.0100
**Cramer-von Mises**	**W-Sq**	8.634324	**Pr > W-Sq**	<0.0050
**Anderson-Darling**	**A-Sq**	38.73335	**Pr > A-Sq**	<0.0050

The SAS System

The UNIVARIATE Procedure
Variable:
RES

Quantiles (Definition 5)	
**Level**	**Quantile**
**100% Max**	0.7246377
**99%**	0.7054871
**95%**	0.3584229
**90%**	0.3436426
**75% Q3**	0.0000000
**50% Median**	0.0000000
**25% Q1**	0.0000000
**10%**	-0.0104488
**5%**	-0.6802721
**1%**	-1.3745704
**0% Min**	-1.4492754

Extreme Observations			
**Lowest**		**Highest**	
**Value**	**Obs**	**Value**	**Obs**
-1.44928	29	0.694444	42
-1.38889	13	0.694444	71
-1.37457	41	0.705487	54
-1.33333	178	0.724638	58
-1.33333	120	0.724638	87

The SAS System

The UNIVARIATE Procedure

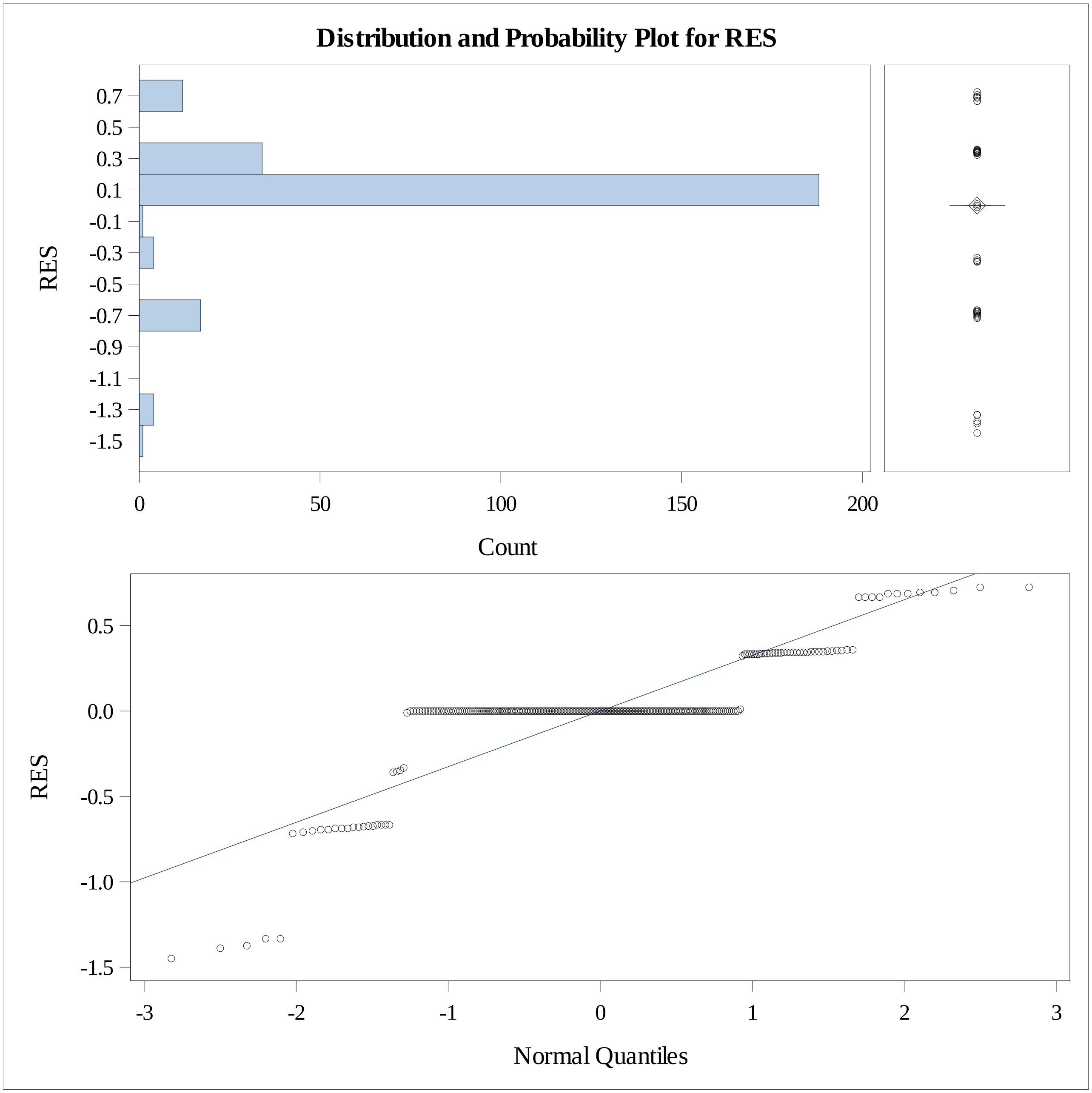

The SAS System

The MEANS Procedure

Analysis Variable : SR SR						
**Device**	**N Obs**	**N**	**Mean**	**Std Dev**	**Minimum**	**Maximum**
casero	87	87	99.9190734	0.3146070	98.0000000	100.0000000
glass	87	87	99.8364732	0.4843464	97.0000000	100.0000000
hemo	87	87	99.7377927	0.5539148	97.8260870	100.0000000

Egg production

```
/*Eggs/mosquitoes Experiment #2*/
/*Import S1_File_July2023 Sheet Eggs$*/

DATA EXP2_EGGS;
SET EGGS;
IF EXP = 2;
RUN;

ODS RTF FILE='EggsExp2.RTF';
PROC GLM DATA=EXP2_EGGS;
CLASS  Week repeat Device;
MODEL egg_mosq =  Week Device /SS3;
OUTPUT OUT=R RESIDUAL = RES;
LSMEANS Week / STDERR PDIFF TDIFF;
LSMEANS device / STDERR PDIFF TDIFF;
*LSMEANS repeat / STDERR PDIFF TDIFF;
*LSMEANS week*DEVICE / STDERR PDIFF TDIFF;
*LSMEANS Day*Device / STDERR PDIFF TDIFF;
PROC UNIVARIATE NORMAL PLOT DATA=R; VAR RES; RUN;

proc means data=EXP2_EGGS ;
var egg_mosq;
class week device;
run;

proc means data=EXP2_EGGS;
var egg_mosq;
class device;
run;

ODS RTF CLOSE;
```

The SAS System

The GLM Procedure

Class Level Information		
**Class**	**Levels**	**Values**
**Week**	4	1 2 3 4
**Repeat**	3	A B C
**Device**	3	CASERO GLASS HEMO

**Number of Observations Read**	36
**Number of Observations Used**	36

The SAS System

The GLM Procedure

Dependent Variable: egg_mosq
egg_mosq

Source	DF	Sum of Squares	Mean Square	F Value	Pr > F
**Model**	5	1330.006721	266.001344	8.75	<.0001
**Error**	30	912.483544	30.416118		
**Corrected Total**	35	2242.490265			

R-Square	Coeff Var	Root MSE	egg_mosq Mean
0.593094	21.98921	5.515081	25.08085

Source	DF	Type III SS	Mean Square	F Value	Pr > F
**Week**	3	457.7431415	152.5810472	5.02	0.0062
**Device**	2	872.2635798	436.1317899	14.34	<.0001

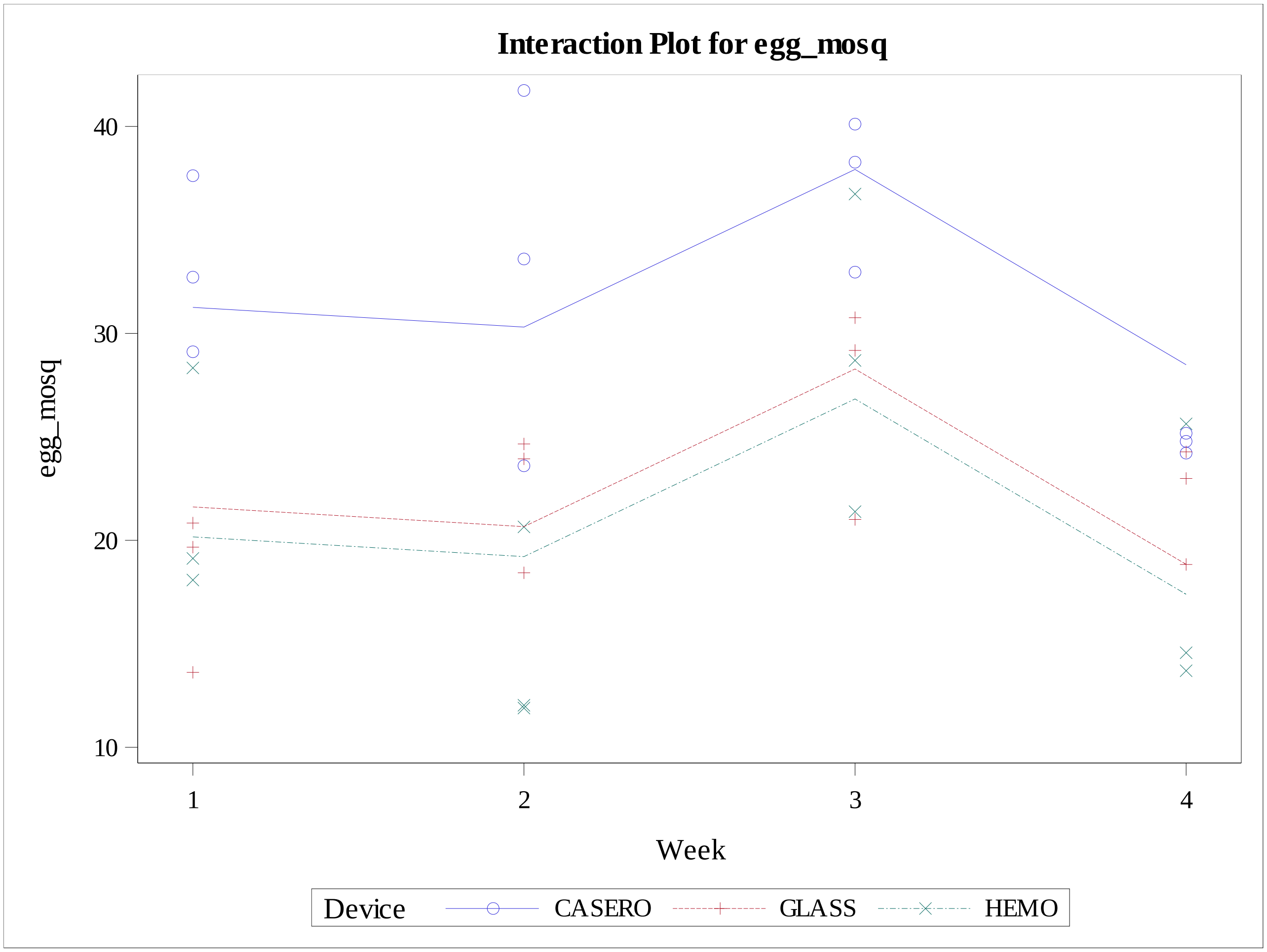

The SAS System

The GLM Procedure
Least Squares Means

Week	egg_mosq LSMEAN	Standard Error	Pr > |t|	LSMEAN Number
**1**	24.3458883	1.8383603	<.0001	1
**2**	23.3928831	1.8383603	<.0001	2
**3**	31.0112005	1.8383603	<.0001	3
**4**	21.5734447	1.8383603	<.0001	4

Least Squares Means for Effect Week
t for H0: LSMean(i)=LSMean(j) / Pr > |t|

Dependent Variable: egg_mosq

i/j	1	2	3	4
**1**		0.366564<br>0.7165	-2.56375<br>0.0156	1.066393<br>0.2948
**2**	-0.36656<br>0.7165		-2.93031<br>0.0064	0.699829<br>0.4894
**3**	2.563745<br>0.0156	2.930309<br>0.0064		3.630138<br>0.0010
**4**	-1.06639<br>0.2948	-0.69983<br>0.4894	-3.63014<br>0.0010	

The SAS System

The GLM Procedure
Least Squares Means

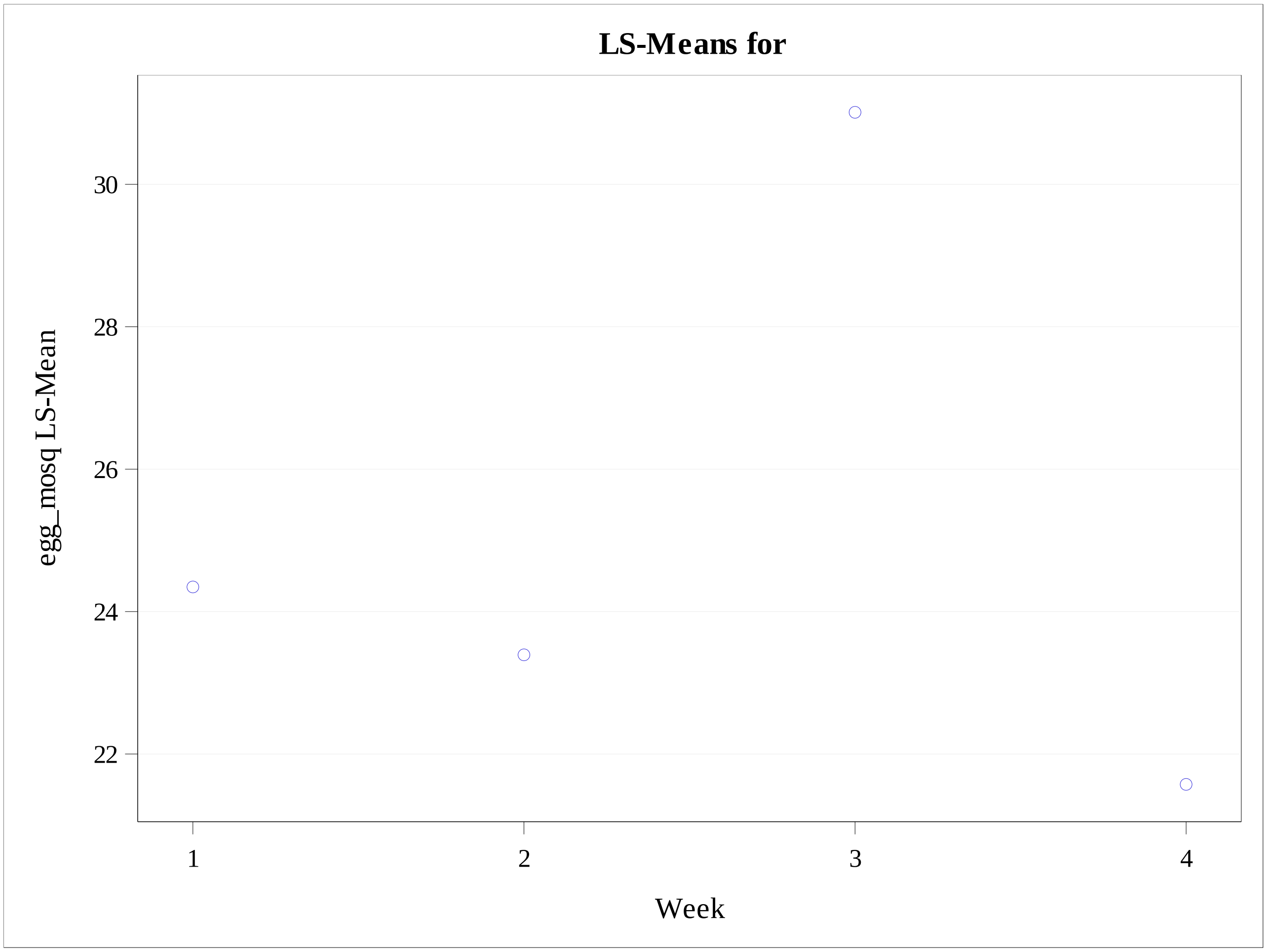

The SAS System

The GLM Procedure
Least Squares Means

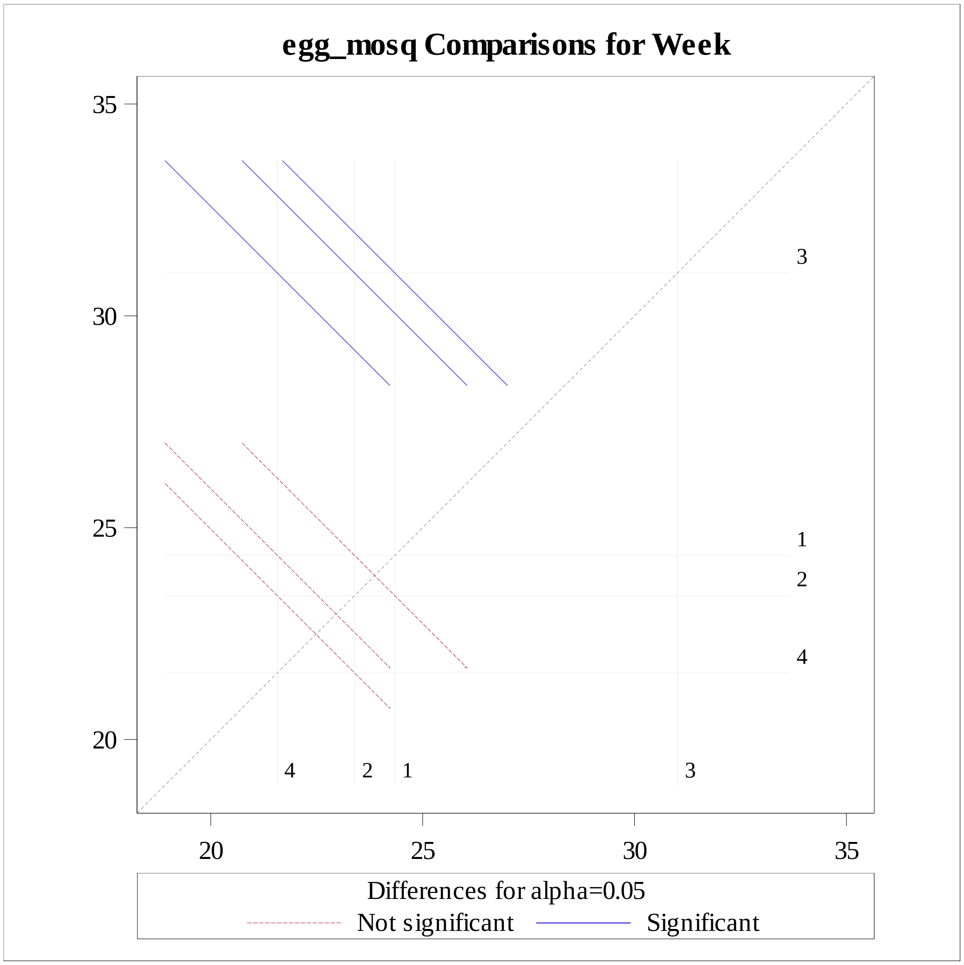

Note: To ensure overall protection level, only probabilities associated with pre-planned comparisons should be used.

The SAS System

The GLM Procedure
Least Squares Means

Device	egg_mosq LSMEAN	Standard Error	Pr > |t|	LSMEAN Number
**CASERO**	31.9917183	1.5920667	<.0001	1
**GLASS**	22.3494966	1.5920667	<.0001	2
**HEMO**	20.9013476	1.5920667	<.0001	3

Least Squares Means for Effect Device
t for H0: LSMean(i)=LSMean(j) / Pr > |t|

Dependent Variable: egg_mosq

i/j	1	2	3
**1**		4.282534<br>0.0002	4.925721<br><.0001
**2**	-4.28253<br>0.0002		0.643187<br>0.5250
**3**	-4.92572<br><.0001	-0.64319<br>0.5250	

The SAS System

The GLM Procedure
Least Squares Means

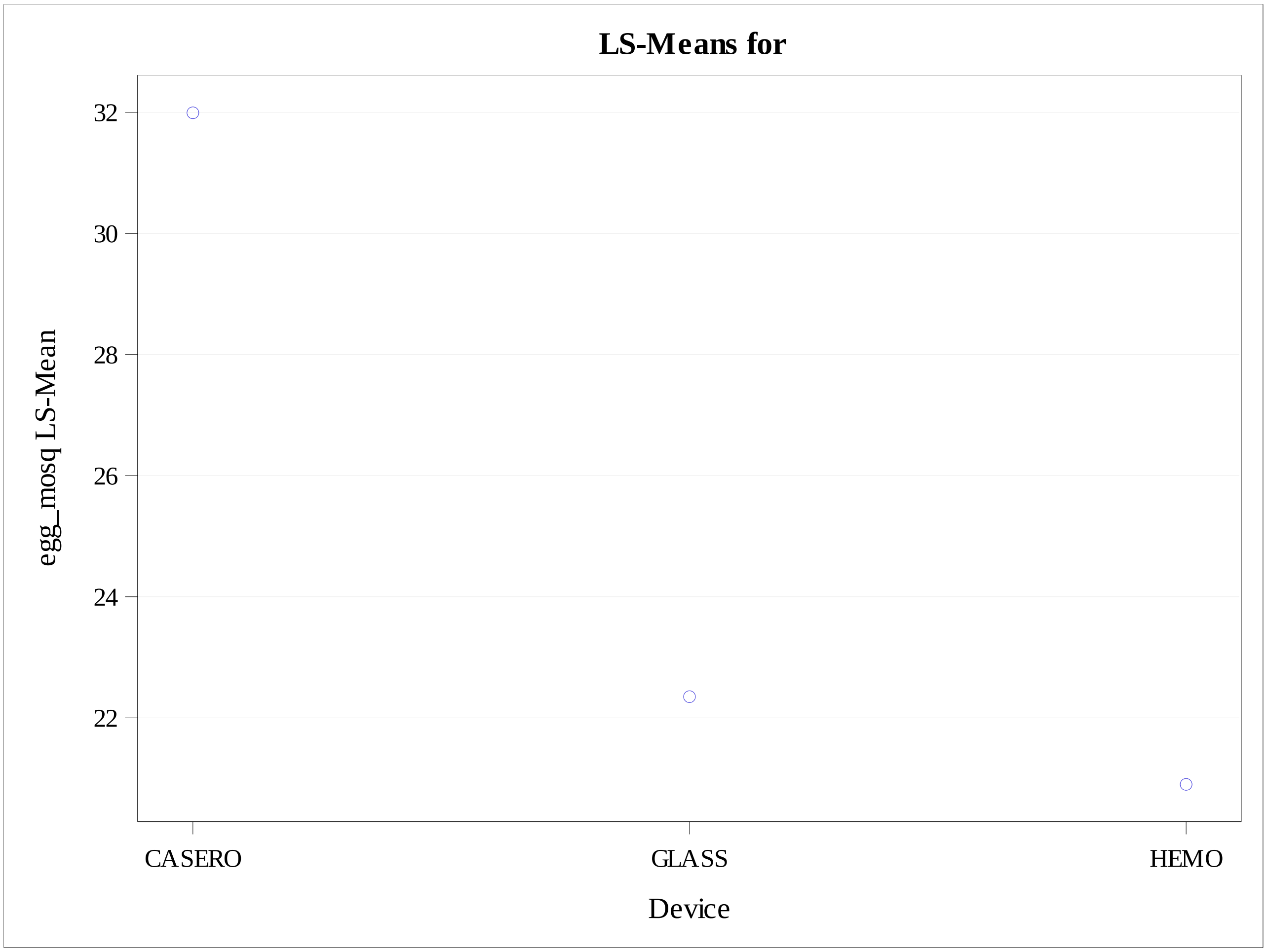

The SAS System

The GLM Procedure
Least Squares Means

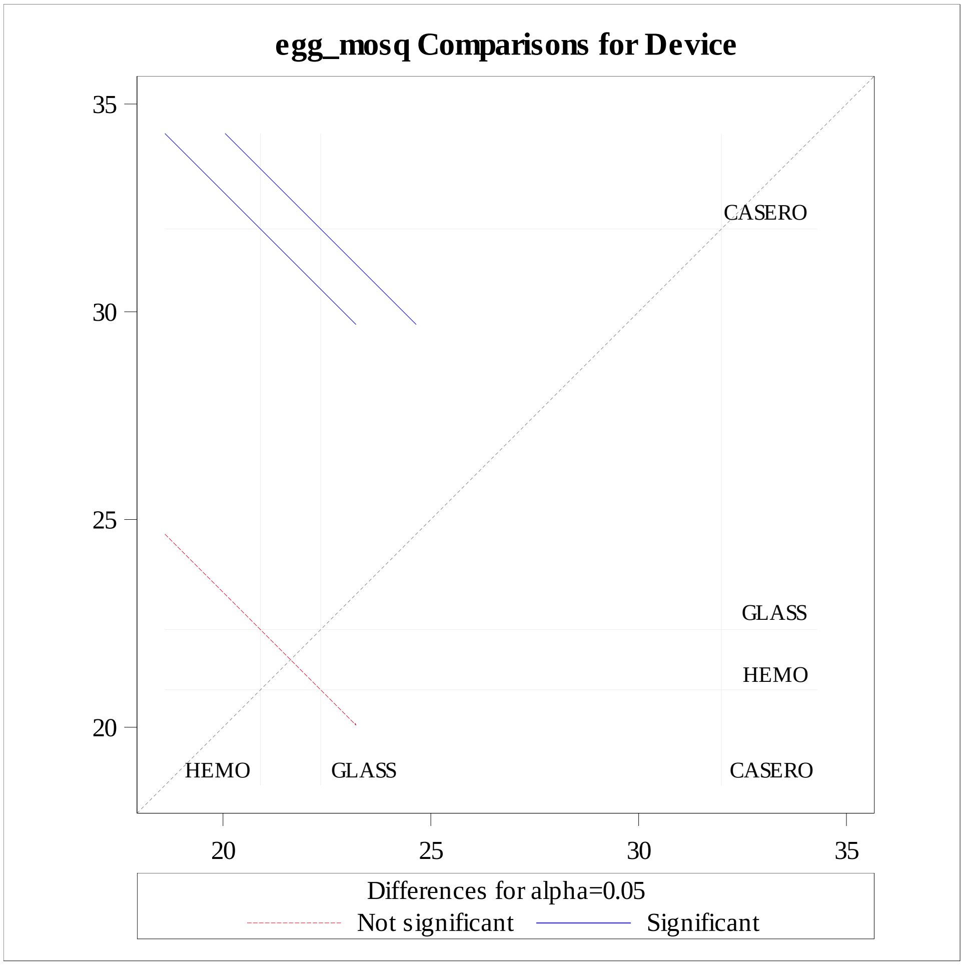

Note: To ensure overall protection level, only probabilities associated with pre-planned comparisons should be used.

The SAS System

The UNIVARIATE Procedure
Variable: RES

Moments			
**N**	36	**Sum Weights**	36
**Mean**	0	**Sum Observations**	0
**Std Deviation**	5.10597281	**Variance**	26.0709584
**Skewness**	0.38900579	**Kurtosis**	-0.4809371
**Uncorrected SS**	912.483544	**Corrected SS**	912.483544
**Coeff Variation**	.	**Std Error Mean**	0.85099547

Basic Statistical Measures			
**Location**		**Variability**	
**Mean**	0.00000	**Std Deviation**	5.10597
**Median**	-0.39412	**Variance**	26.07096
**Mode**	.	**Range**	19.42710
		**Interquartile Range**	6.98232

Tests for Location: Mu0=0				
**Test**	**Statistic**		**p Value**	
**Student's t**	**t**	0	**Pr > |t|**	1.0000
**Sign**	**M**	-1	**Pr >= |M|**	0.8679
**Signed Rank**	**S**	-14	**Pr >= |S|**	0.8295

Tests for Normality				
**Test**	**Statistic**		**p Value**	
**Shapiro-Wilk**	**W**	0.969388	**Pr < W**	0.4091
**Kolmogorov-Smirnov**	**D**	0.092774	**Pr > D**	>0.1500
**Cramer-von Mises**	**W-Sq**	0.031047	**Pr > W-Sq**	>0.2500
**Anderson-Darling**	**A-Sq**	0.255957	**Pr > A-Sq**	>0.2500

The SAS System

The UNIVARIATE Procedure
Variable: RES

Quantiles (Definition 5)	
**Level**	**Quantile**
**100% Max**	11.433626
**99%**	11.433626
**95%**	9.902349
**90%**	8.163515
**75% Q3**	3.284416
**50% Median**	-0.394117
**25% Q1**	-3.697909
**10%**	-7.182449
**5%**	-7.318640
**1%**	-7.993478
**0% Min**	-7.993478

Extreme Observations			
**Lowest**		**Highest**	
**Value**	**Obs**	**Value**	**Obs**
-7.99348	5	6.36325	4
-7.31864	15	8.16352	6
-7.26953	20	8.23372	33
-7.18245	18	9.90235	21
-6.70171	10	11.43363	16

The SAS System

The UNIVARIATE Procedure

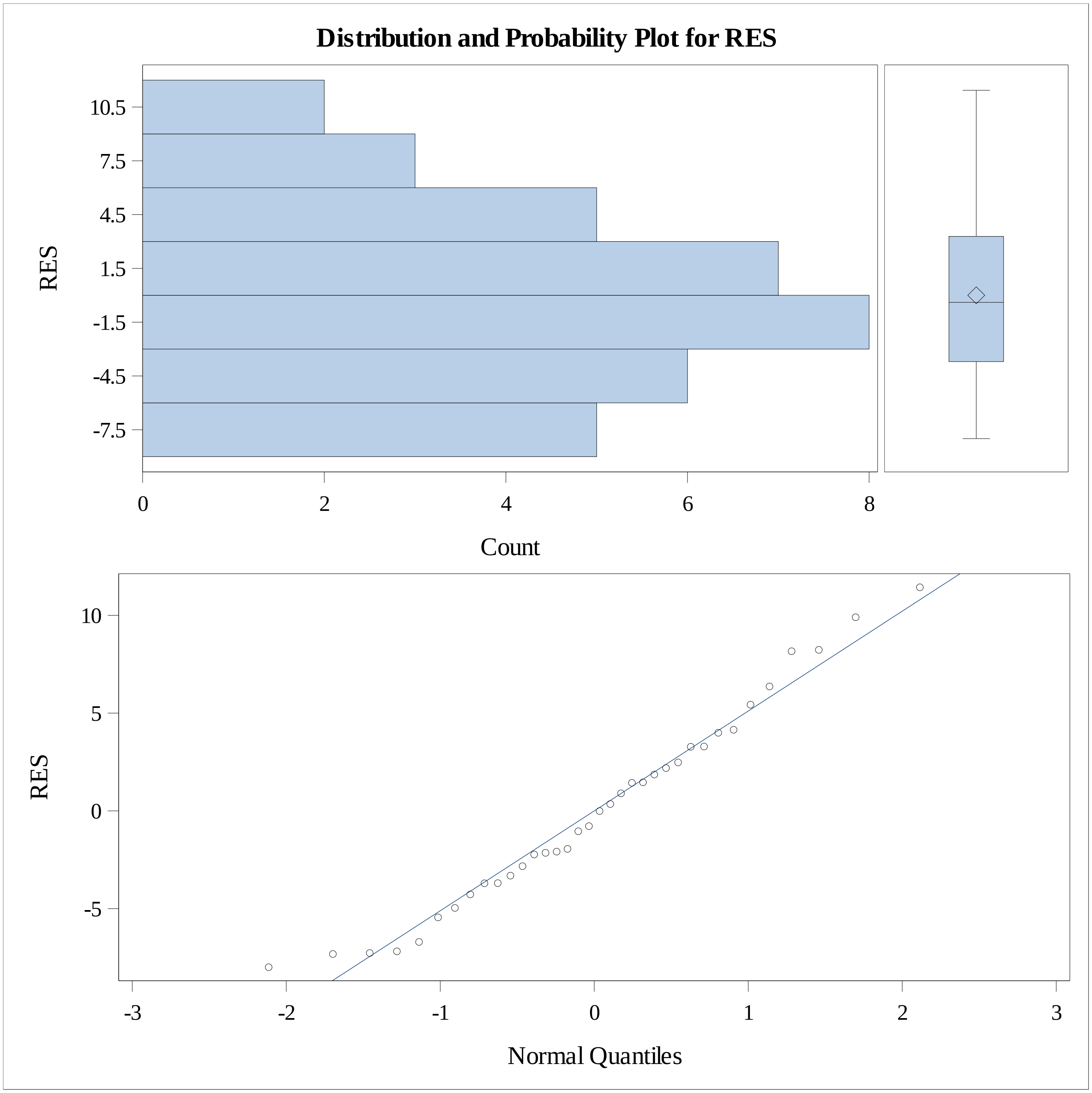

The SAS System

The MEANS Procedure

Analysis Variable : egg_mosq egg_mosq			
**Week**	**Device**	**N Obs**	**95th Pctl**
1	CASERO	3	37.6200000
	GLASS	3	20.8350515
	HEMO	3	28.3298969
2	CASERO	3	41.7373737
	GLASS	3	24.6562500
	HEMO	3	20.6489362
3	CASERO	3	40.1122449
	GLASS	3	30.7578947
	HEMO	3	36.7340426
4	CASERO	3	25.1734694
	GLASS	3	24.2736842
	HEMO	3	25.6276596

The SAS System

The MEANS Procedure

Analysis Variable : egg_mosq egg_mosq						
**Device**	**N Obs**	**N**	**Mean**	**Std Dev**	**Minimum**	**Maximum**
CASERO	12	12	31.9917183	6.5622849	23.6020408	41.7373737
GLASS	12	12	22.3494966	4.7139967	13.6210526	30.7578947
HEMO	12	12	20.9013476	7.6993970	11.8947368	36.7340426

Experiment #3 General Linear Models (GLM) for:

1. Feeding Rate
2. Egg production and hatch rate

The study design for Experiment #3 carried out the same experiment during 3 different weeks (day variable in feeding data sheet), but no long term for the Anopheles cohorts were carried out. We present our SAS code used to analyze S1 Data File followed by model output directly from SAS.

Feeding Rate

```
/*Anopheles-Experiment#3*/
/*Import S1_File_July2023 Sheet FEED$*/

DATA EXP3_FEED;
SET FEED;
IF EXP = 3;
RUN;

ODS RTF FILE='Feed_AN.RTF';
PROC GLM DATA=EXP3_FEED;
CLASS  Day Repeat Device;
MODEL Feedrate =  Device repeat repeat*device /SS3;
OUTPUT OUT=R RESIDUAL = RES;
*LSMEANS DAY / STDERR PDIFF TDIFF;
LSMEANS Repeat / STDERR PDIFF TDIFF;
LSMEANS DEVICE / STDERR PDIFF TDIFF;
LSMEANS repeat*Device / STDERR PDIFF TDIFF;

PROC UNIVARIATE NORMAL PLOT DATA=R; VAR RES; RUN;


proc means data=EXP3_FEED mean std stderr;
var Feedrate;
class  device;
run;
proc means data=EXP3_FEED mean std stderr;
var Feedrate;
class  day device;
run;
ODS RTF CLOSE;
```

The SAS System

The GLM Procedure

Class Level Information		
**Class**	**Levels**	**Values**
**Day**	3	1 2 3
**Repeat**	3	A B C
**Device**	3	casero glass hemo

**Number of Observations Read**	27
**Number of Observations Used**	27

The SAS System

The GLM Procedure

Dependent Variable: feedrate feedrate

Source	DF	Sum of Squares	Mean Square	F Value	Pr > F
**Model**	8	998.489933	124.811242	0.92	0.5230
**Error**	18	2442.476334	135.693130		
**Corrected Total**	26	3440.966266			

R-Square	Coeff Var	Root MSE	feedrate Mean
0.290177	15.33217	11.64874	75.97582

Source	DF	Type III SS	Mean Square	F Value	Pr > F
**Device**	2	387.4922774	193.7461387	1.43	0.2657
**Repeat**	2	121.1375807	60.5687903	0.45	0.6468
**Repeat*Device**	4	489.8600744	122.4650186	0.90	0.4832

The SAS System

The GLM Procedure

Dependent Variable: feedrate feedrate

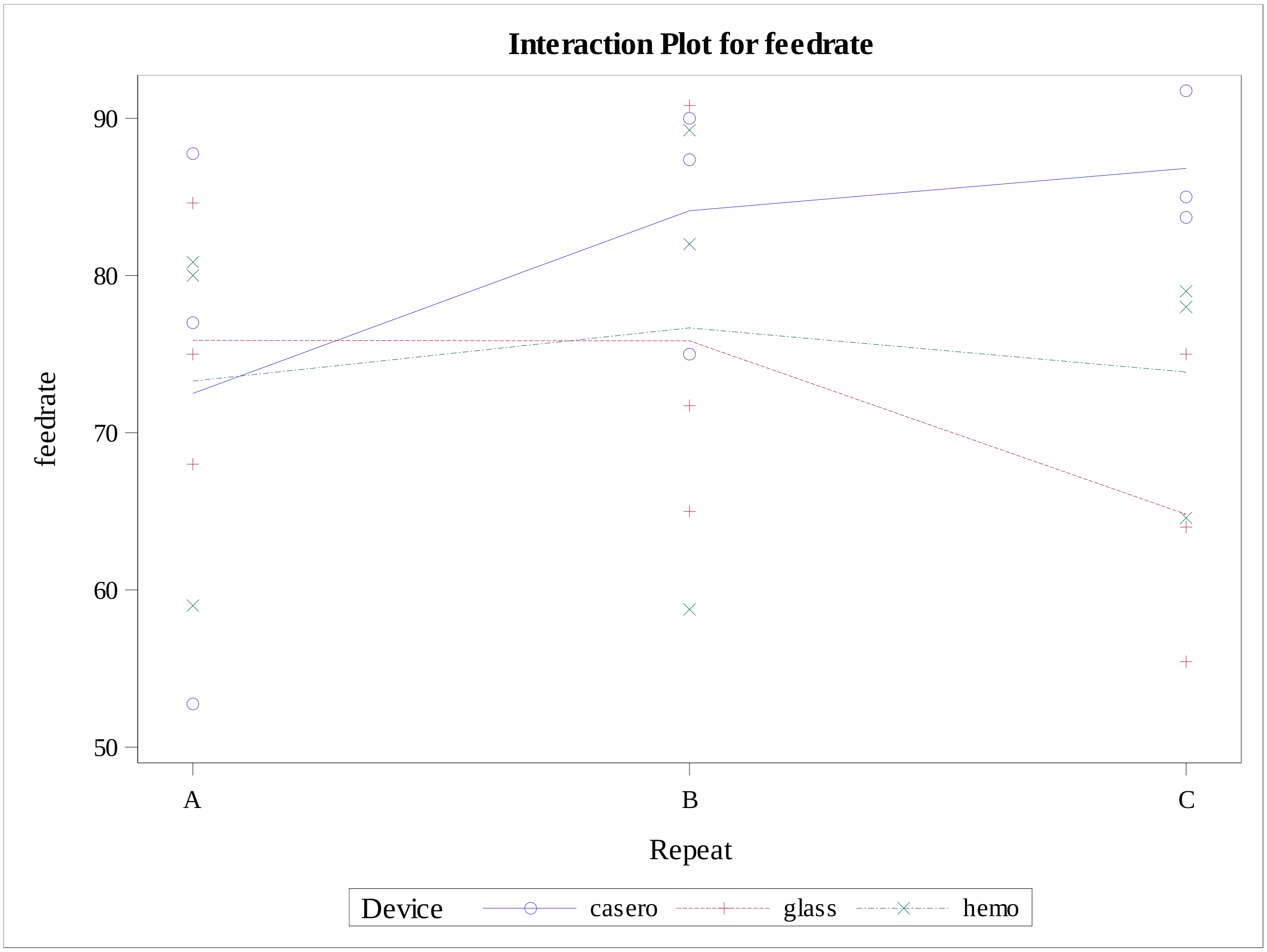

The SAS System

The GLM Procedure
Least Squares Means

Repeat	feedrate LSMEAN	Standard Error	Pr > |t|	LSMEAN Number
**A**	73.8854226	3.8829131	<.0001	1
**B**	78.8791242	3.8829131	<.0001	2
**C**	75.1629273	3.8829131	<.0001	3

Least Squares Means for Effect Repeat
t for H0: LSMean(i)=LSMean(j) / Pr > |t|

Dependent Variable: feedrate

i/j	1	2	3
**1**		-0.90939<br>0.3752	-0.23264<br>0.8187
**2**	0.909389<br>0.3752		0.676747<br>0.5072
**3**	0.232643<br>0.8187	-0.67675<br>0.5072	

The SAS System

The GLM Procedure
Least Squares Means

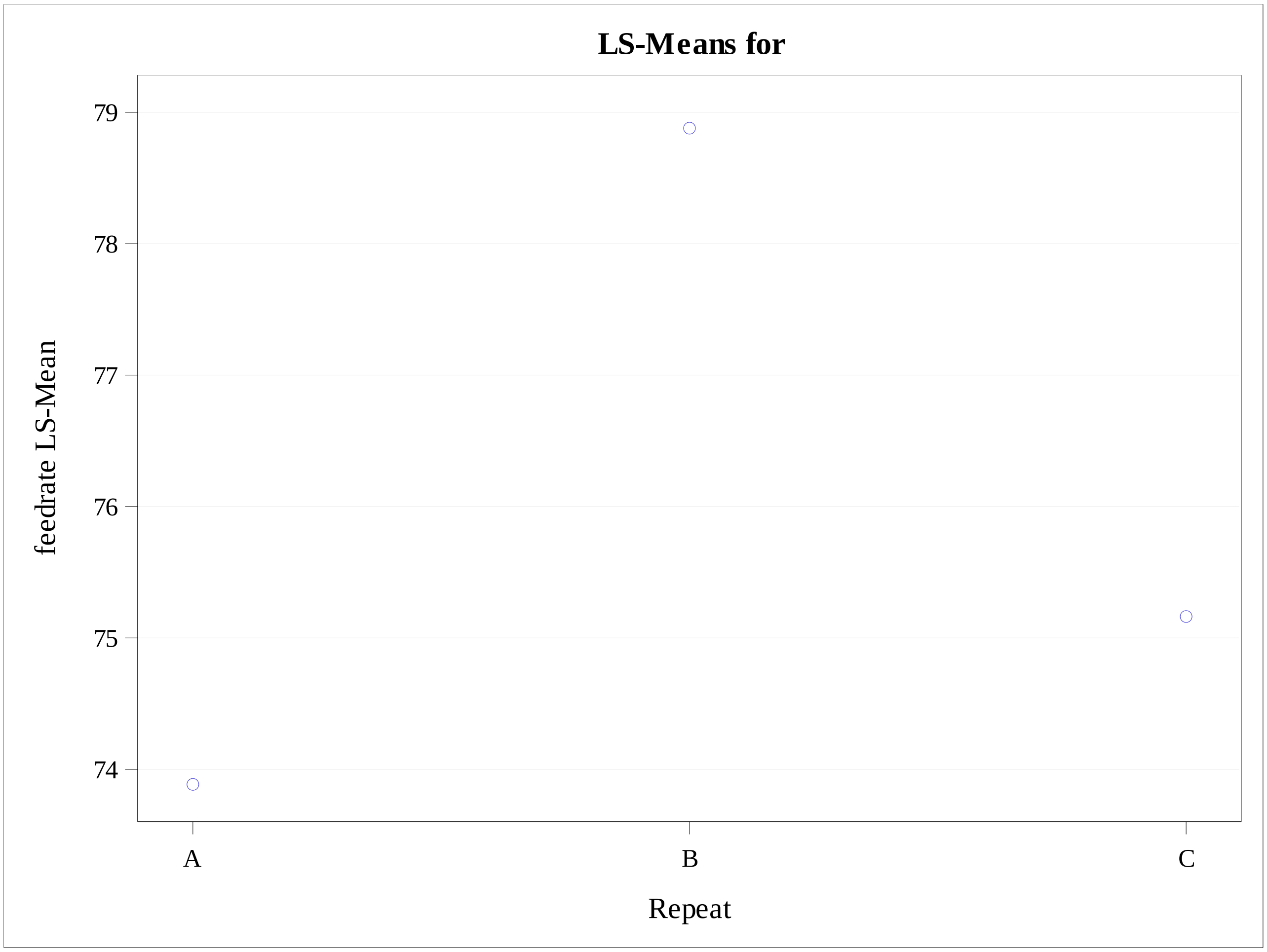

The SAS System

The GLM Procedure
Least Squares Means

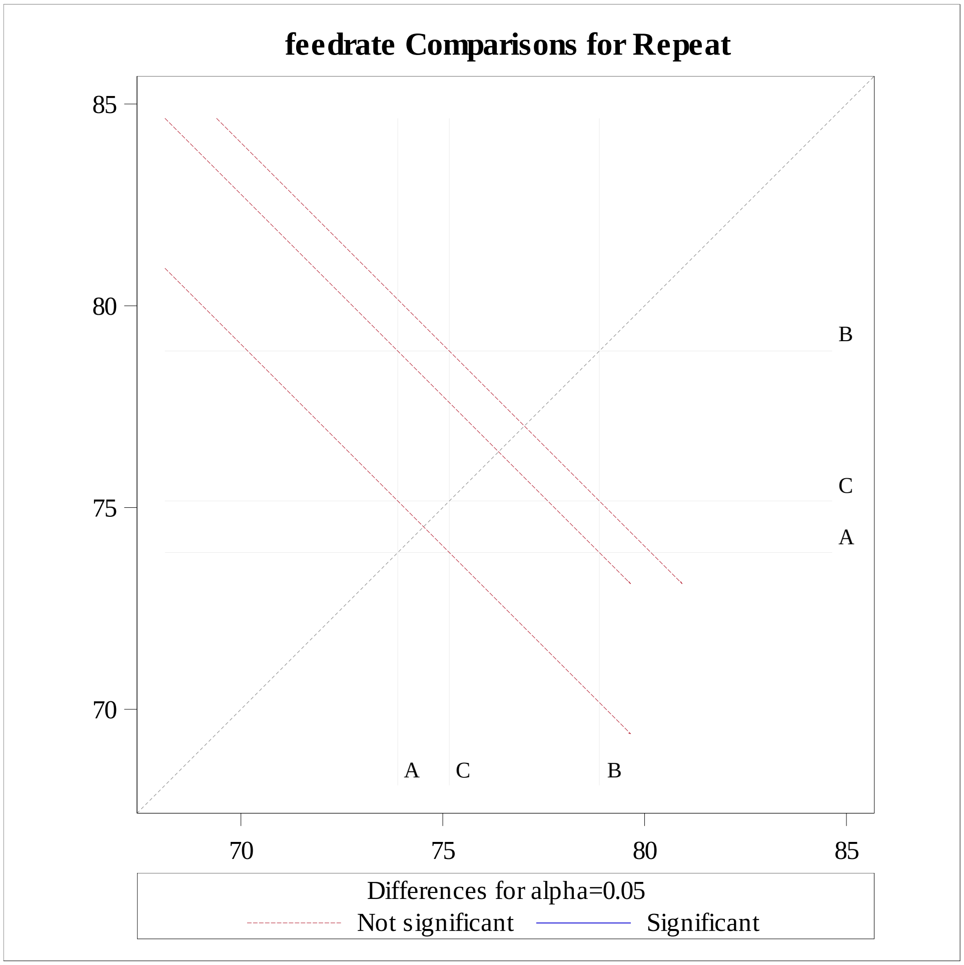

Note: To ensure overall protection level, only probabilities associated with pre-planned comparisons should be used.

The SAS System

The GLM Procedure
Least Squares Means

Device	feedrate LSMEAN	Standard Error	Pr > |t|	LSMEAN Number
**casero**	81.1465561	3.8829131	<.0001	1
**glass**	72.1759628	3.8829131	<.0001	2
**hemo**	74.6049551	3.8829131	<.0001	3

Least Squares Means for Effect Device
t for H0: LSMean(i)=LSMean(j) / Pr > |t|

Dependent Variable: feedrate

i/j	1	2	3
**1**		1.63361<br>0.1197	1.191273<br>0.2490
**2**	-1.63361<br>0.1197		-0.44234<br>0.6635
**3**	-1.19127<br>0.2490	0.442337<br>0.6635	

The SAS System

The GLM Procedure
Least Squares Means

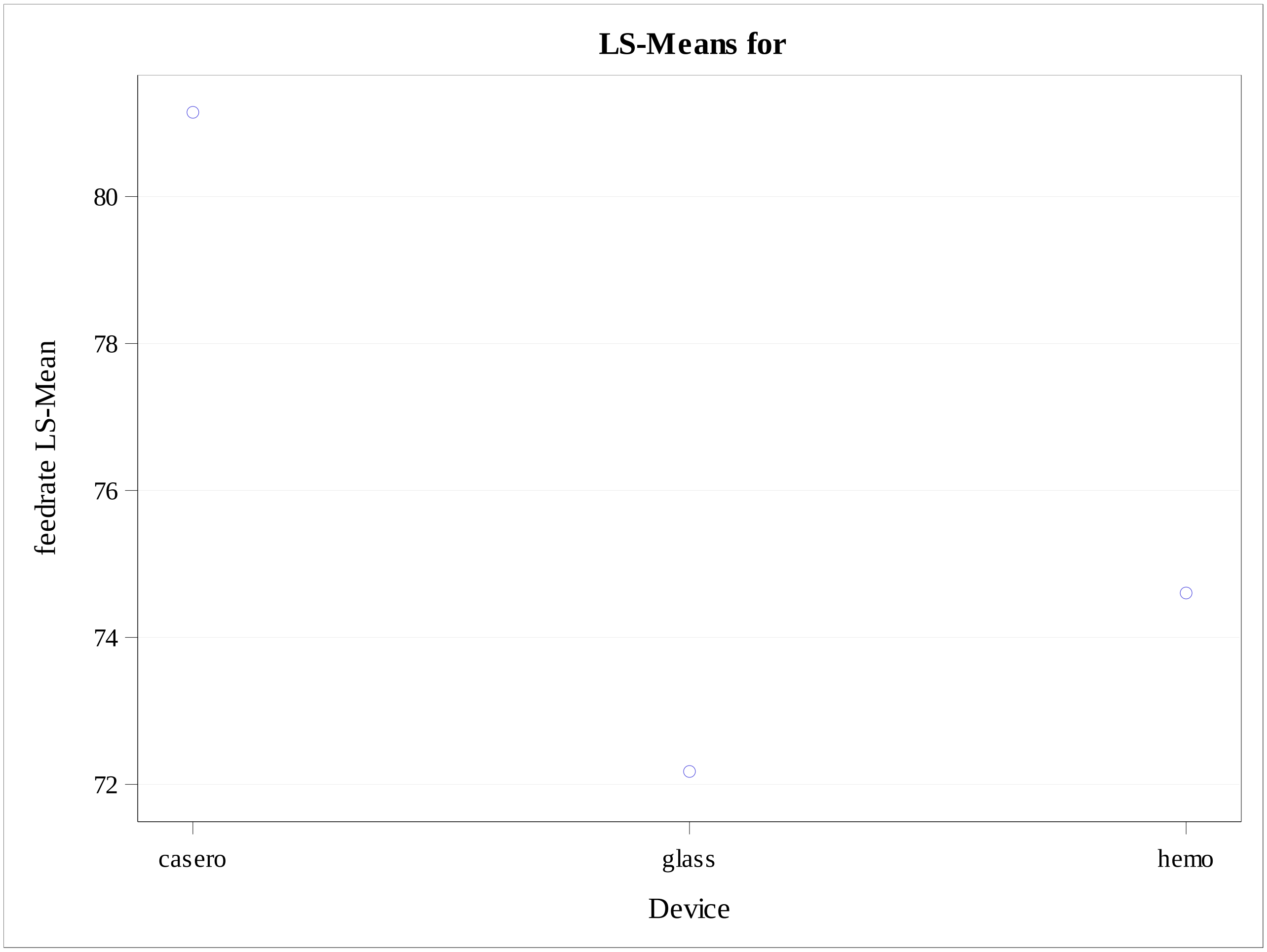

The SAS System

The GLM Procedure
Least Squares Means

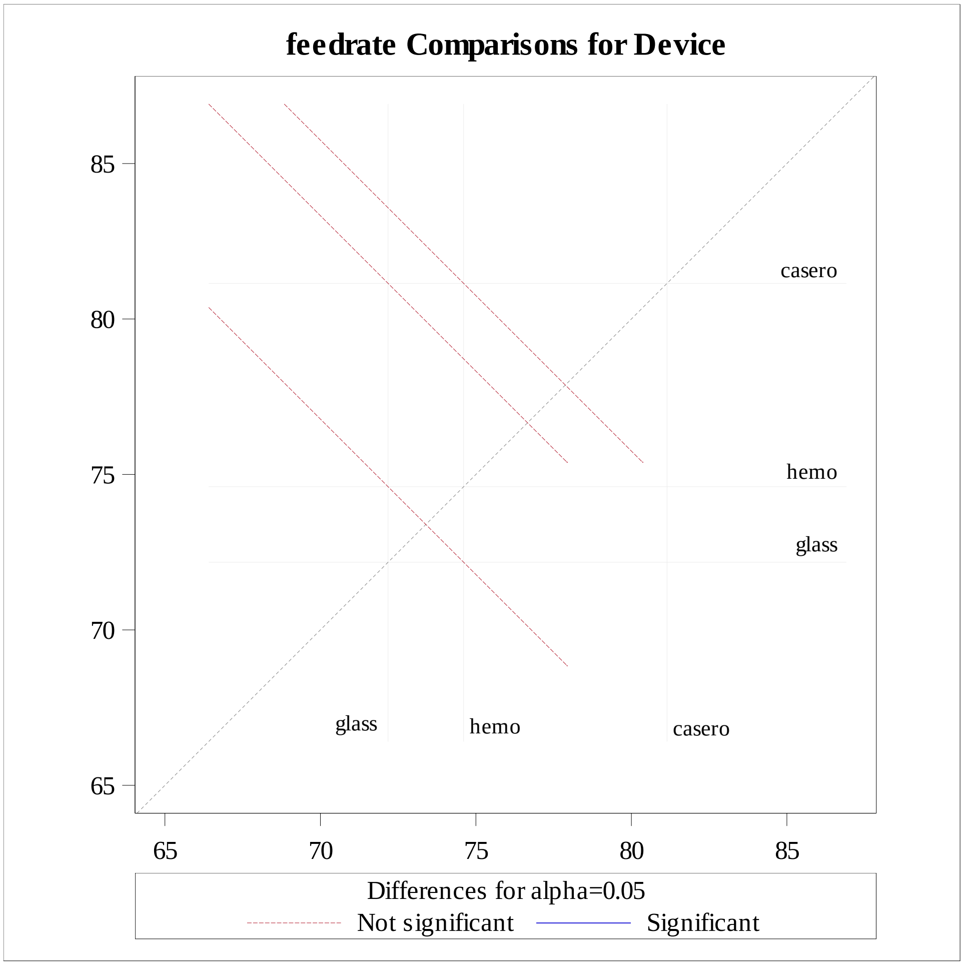

Note: To ensure overall protection level, only probabilities associated with pre-planned comparisons should be used.

The SAS System

The GLM Procedure
Least Squares Means

Repeat	Device	feedrate LSMEAN	Standard Error	Pr > |t|	LSMEAN Number
**A**	**casero**	72.5007849	6.7254028	<.0001	1
**A**	**glass**	75.8717949	6.7254028	<.0001	2
**A**	**hemo**	73.2836879	6.7254028	<.0001	3
**B**	**casero**	84.1228070	6.7254028	<.0001	4
**B**	**glass**	75.8444994	6.7254028	<.0001	5
**B**	**hemo**	76.6700661	6.7254028	<.0001	6
**C**	**casero**	86.8160765	6.7254028	<.0001	7
**C**	**glass**	64.8115942	6.7254028	<.0001	8
**C**	**hemo**	73.8611111	6.7254028	<.0001	9

Least Squares Means for Effect Repeat*Device
t for H0: LSMean(i)=LSMean(j) / Pr > |t|

Dependent Variable: feedrate

i/j	1	2	3	4	5	6	7	8	9
**1**		-0.35443<br>0.7271	-0.08231<br>0.9353	-1.22194<br>0.2375	-0.35156<br>0.7293	-0.43836<br>0.6663	-1.50511<br>0.1496	0.808439<br>0.4294	-0.14302<br>0.8879
**2**	0.354427<br>0.7271		0.272113<br>0.7886	-0.86751<br>0.3971	0.00287<br>0.9977	-0.08393<br>0.9340	-1.15068<br>0.2649	1.162866<br>0.2601	0.211403<br>0.8349
**3**	0.082314<br>0.9353	-0.27211<br>0.7886		-1.13962<br>0.2694	-0.26924<br>0.7908	-0.35604<br>0.7259	-1.42279<br>0.1719	0.890753<br>0.3848	-0.06071<br>0.9523
**4**	1.221936<br>0.2375	0.867509<br>0.3971	1.139622<br>0.2694		0.870379<br>0.3956	0.783579<br>0.4435	-0.28317<br>0.7803	2.030375<br>0.0574	1.078912<br>0.2949
**5**	0.351557<br>0.7293	-0.00287<br>0.9977	0.269243<br>0.7908	-0.87038<br>0.3956		-0.0868<br>0.9318	-1.15355<br>0.2638	1.159996<br>0.2612	0.208533<br>0.8372
**6**	0.438357<br>0.6663	0.08393<br>0.9340	0.356043<br>0.7259	-0.78358<br>0.4435	0.0868<br>0.9318		-1.06675<br>0.3002	1.246796<br>0.2285	0.295333<br>0.7711
**7**	1.505105<br>0.1496	1.150678<br>0.2649	1.422791<br>0.1719	0.28317<br>0.7803	1.153548<br>0.2638	1.066748<br>0.3002		2.313544<br>0.0327	1.362081<br>0.1900
**8**	-0.80844<br>0.4294	-1.16287<br>0.2601	-0.89075<br>0.3848	-2.03037<br>0.0574	-1.16<br>0.2612	-1.2468<br>0.2285	-2.31354<br>0.0327		-0.95146<br>0.3540
**9**	0.143024<br>0.8879	-0.2114<br>0.8349	0.06071<br>0.9523	-1.07891<br>0.2949	-0.20853<br>0.8372	-0.29533<br>0.7711	-1.36208<br>0.1900	0.951463<br>0.3540	

The SAS System

The GLM Procedure
Least Squares Means

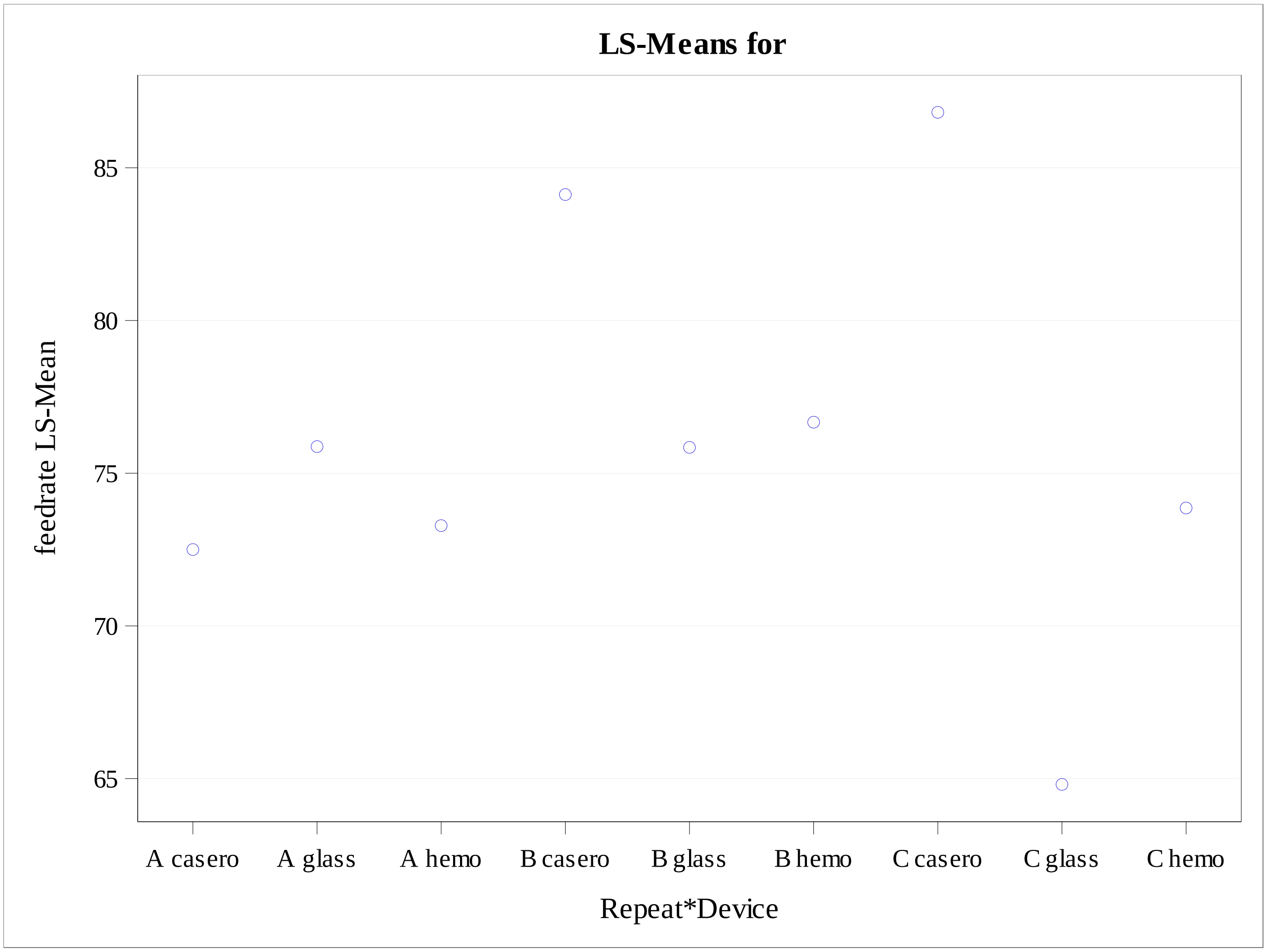

The SAS System

The GLM Procedure
Least Squares Means

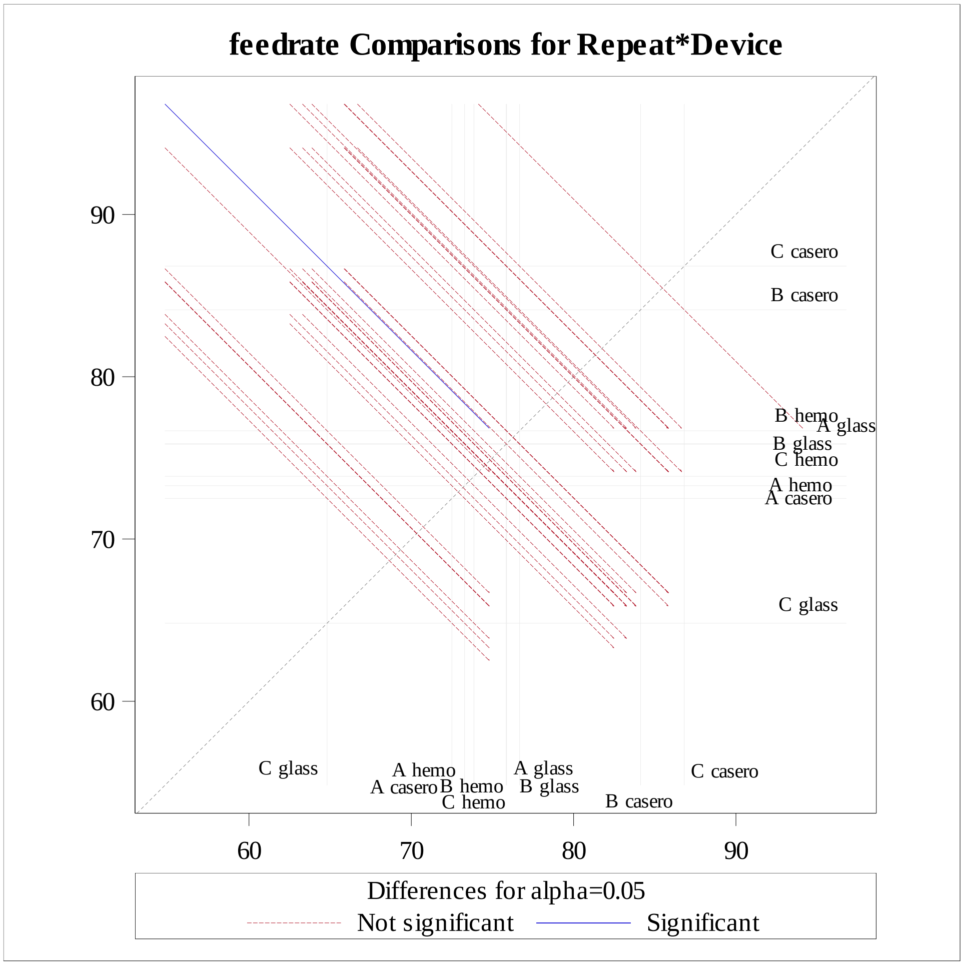

Note: To ensure overall protection level, only probabilities associated with pre-planned comparisons should be used.

The SAS System

The UNIVARIATE Procedure
Variable: RES

Moments			
**N**	27	**Sum Weights**	27
**Mean**	0	**Sum Observations**	0
**Std Deviation**	9.69233705	**Variance**	93.9413974
**Skewness**	-0.3856579	**Kurtosis**	-0.6765872
**Uncorrected SS**	2442.47633	**Corrected SS**	2442.47633
**Coeff Variation**	.	**Std Error Mean**	1.86529113

Basic Statistical Measures			
**Location**		**Variability**	
**Mean**	0.000000	**Std Deviation**	9.69234
**Median**	3.245614	**Variance**	93.94140
**Mode**	.	**Range**	35.00785
		**Interquartile Range**	15.83912

Tests for Location: Mu0=0				
**Test**	**Statistic**		**p Value**	
**Student's t**	**t**	0	**Pr > |t|**	1.0000
**Sign**	**M**	0.5	**Pr >= |M|**	1.0000
**Signed Rank**	**S**	7	**Pr >= |S|**	0.8701

Tests for Normality				
**Test**	**Statistic**		**p Value**	
**Shapiro-Wilk**	**W**	0.958635	**Pr < W**	0.3439
**Kolmogorov-Smirnov**	**D**	0.149655	**Pr > D**	0.1209
**Cramer-von Mises**	**W-Sq**	0.075872	**Pr > W-Sq**	0.2307
**Anderson-Darling**	**A-Sq**	0.417495	**Pr > A-Sq**	>0.2500

The SAS System

The UNIVARIATE Procedure
Variable:
RES

Quantiles (Definition 5)	
**Level**	**Quantile**
**100% Max**	15.25432
**99%**	15.25432
**95%**	14.97183
**90%**	12.57725
**75% Q3**	6.71631
**50% Median**	3.24561
**25% Q1**	-9.12281
**10%**	-14.28369
**5%**	-17.90718
**1%**	-19.75353
**0% Min**	-19.75353

Extreme Observations			
**Lowest**		**Highest**	
**Value**	**Obs**	**Value**	**Obs**
-19.75353	16	8.74359	10
-17.90718	23	10.18841	12
-14.28369	22	12.57725	14
-10.84450	2	14.97183	20
-9.37681	21	15.25432	25

The SAS System

The UNIVARIATE Procedure

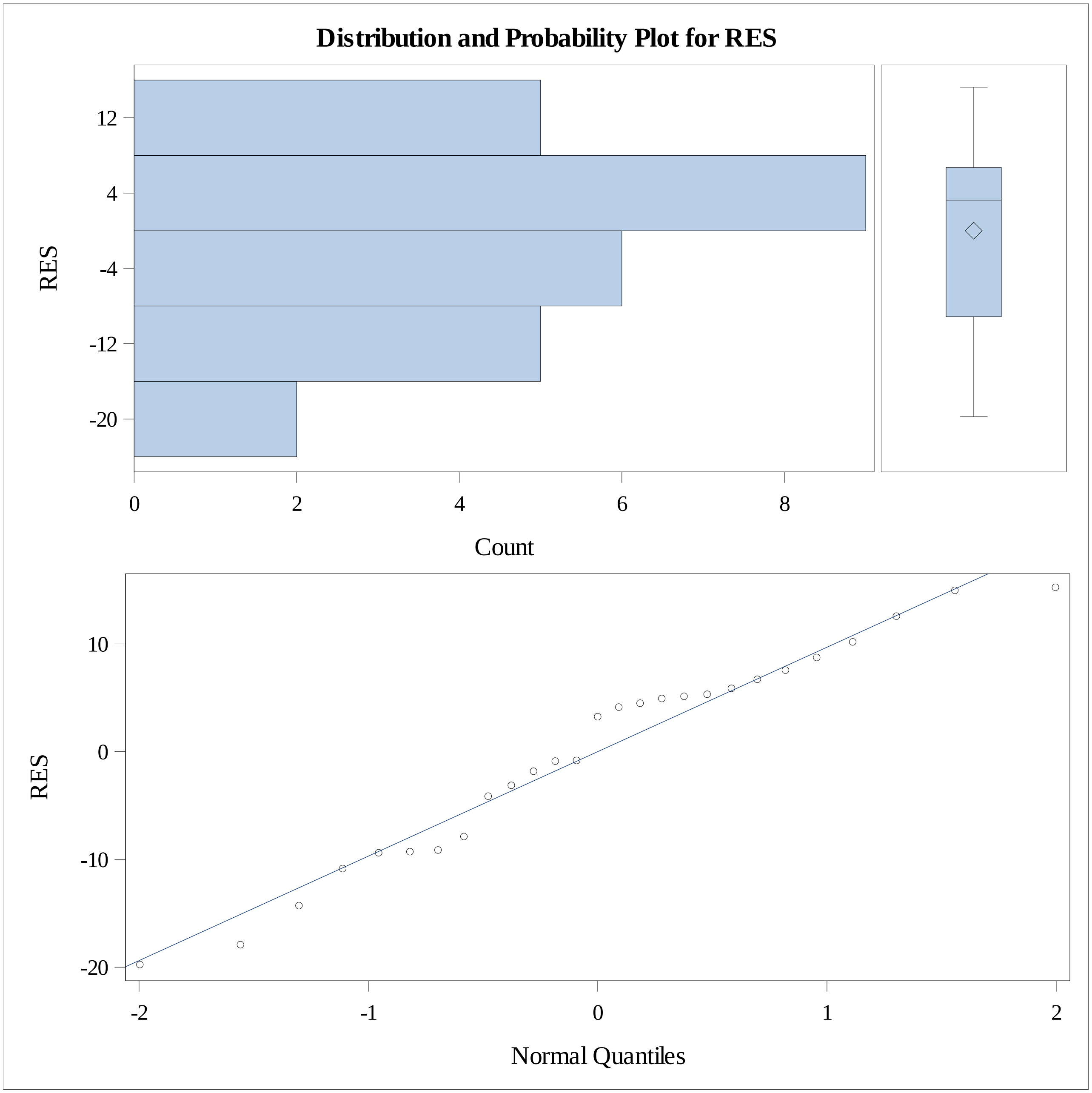

The SAS System

The MEANS Procedure

Analysis Variable : feedrate feedrate				
**Device**	**N Obs**	**Mean**	**Std Dev**	**Std Error**
casero	9	81.1465561	12.0213200	4.0071067
glass	9	72.1759628	10.8074617	3.6024872
hemo	9	74.6049551	10.9713667	3.6571222

The SAS System

The MEANS Procedure

Analysis Variable : feedrate feedrate					
**Day**	**Device**	**N Obs**	**Mean**	**Std Dev**	**Std Error**
1	casero	3	84.0000000	6.5574385	3.7859389
	glass	3	65.6666667	2.0816660	1.2018504
	hemo	3	80.0000000	2.0000000	1.1547005
2	casero	3	70.4809683	15.9614251	9.2153331
	glass	3	77.1108521	6.7031893	3.8700881
	hemo	3	83.0327919	5.4609343	3.1528719
3	casero	3	88.9587001	2.4272810	1.4013913
	glass	3	73.7503697	17.7238425	10.2328653
	hemo	3	60.7820733	3.2941219	1.9018622

Egg production and Hatch rate

```
/*Anopheles-Experiment#3*/
/*Import S1_File_July2023 Sheet FEED$*/

DATA EXP3_EGGS;
SET EGGS;
IF EXP = 3;
RUN;

ODS RTF FILE='EggsAnopheles.RTF';
PROC GLM DATA=EXP3_EGGS;
CLASS Device;
MODEL egg_mosq =  Device /SS3;
OUTPUT OUT=R RESIDUAL = RES;
*LSMEANS Week / STDERR PDIFF TDIFF;
LSMEANS device / STDERR PDIFF TDIFF;
*LSMEANS repeat / STDERR PDIFF TDIFF;
*LSMEANS week*DEVICE / STDERR PDIFF TDIFF;
*LSMEANS Day*Device / STDERR PDIFF TDIFF;
PROC UNIVARIATE NORMAL PLOT DATA=R; VAR RES; RUN;

PROC GLM DATA=EXP3_EGGS;
CLASS Device;
MODEL hatch_rate =  Device /SS3;
OUTPUT OUT=R RESIDUAL = RES;
*LSMEANS Week / STDERR PDIFF TDIFF;
LSMEANS device / STDERR PDIFF TDIFF;
*LSMEANS repeat / STDERR PDIFF TDIFF;
*LSMEANS week*DEVICE / STDERR PDIFF TDIFF;
*LSMEANS Day*Device / STDERR PDIFF TDIFF;
PROC UNIVARIATE NORMAL PLOT DATA=R; VAR RES; RUN;

proc means data=EXP3_eggs;
var egg_mosq hatch_rate;
class device;
run;

proc means data=EXP3_EGGS;
var egg_mosq;
class device;
run;

ODS RTF CLOSE;
```

The SAS System

The GLM Procedure

Class Level Information		
**Class**	**Levels**	**Values**
**Device**	3	CASERO GLASS HEMO

**Number of Observations Read**	9
**Number of Observations Used**	9

The SAS System

The GLM Procedure

Dependent Variable: egg_mosq egg_mosq

Source	DF	Sum of Squares	Mean Square	F Value	Pr > F
Model	2	13.26780000	6.63390000	0.76	0.5059
Error	6	52.03700000	8.67283333		
Corrected Total	8	65.30480000			

R-Square	Coeff Var	Root MSE	egg_mosq Mean
0.203167	23.44097	2.944967	12.56333

Source	DF	Type III SS	Mean Square	F Value	Pr > F
Device	2	13.26780000	6.63390000	0.76	0.5059

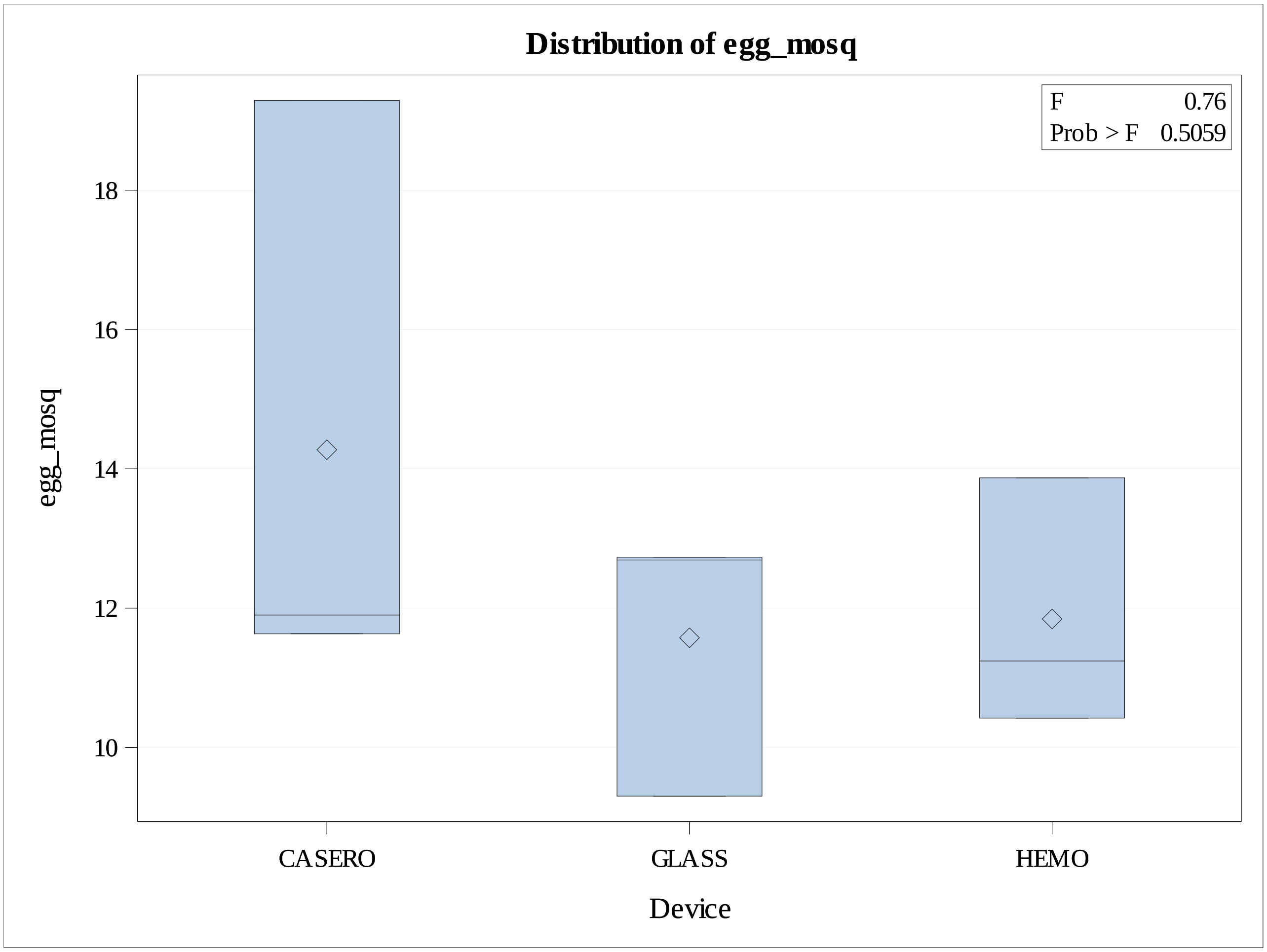

The SAS System

The GLM Procedure
Least Squares Means

Device	egg_mosq LSMEAN	Standard Error	Pr > |t|	LSMEAN Number
**CASERO**	14.2733333	1.7002778	0.0002	1
**GLASS**	11.5733333	1.7002778	0.0005	2
**HEMO**	11.8433333	1.7002778	0.0004	3

Least Squares Means for Effect Device
t for H0: LSMean(i)=LSMean(j) / Pr > |t|

Dependent Variable: egg_mosq

i/j	1	2	3
**1**		1.122868<br>0.3044	1.010582<br>0.3512
**2**	-1.12287<br>0.3044		-0.11229<br>0.9143
**3**	-1.01058<br>0.3512	0.112287<br>0.9143	

The SAS System

The GLM Procedure
Least Squares Means

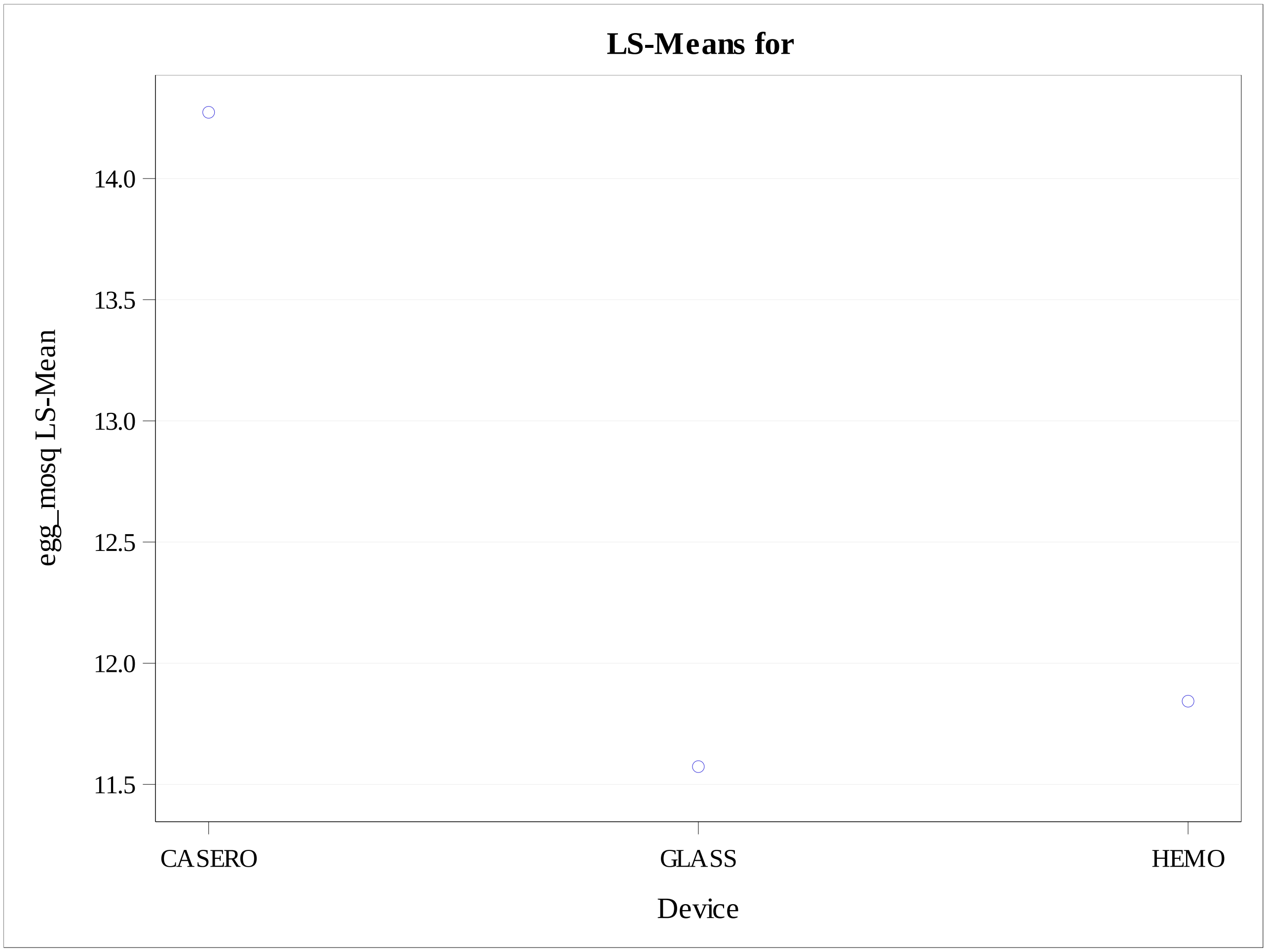

The SAS System

The GLM Procedure
Least Squares Means

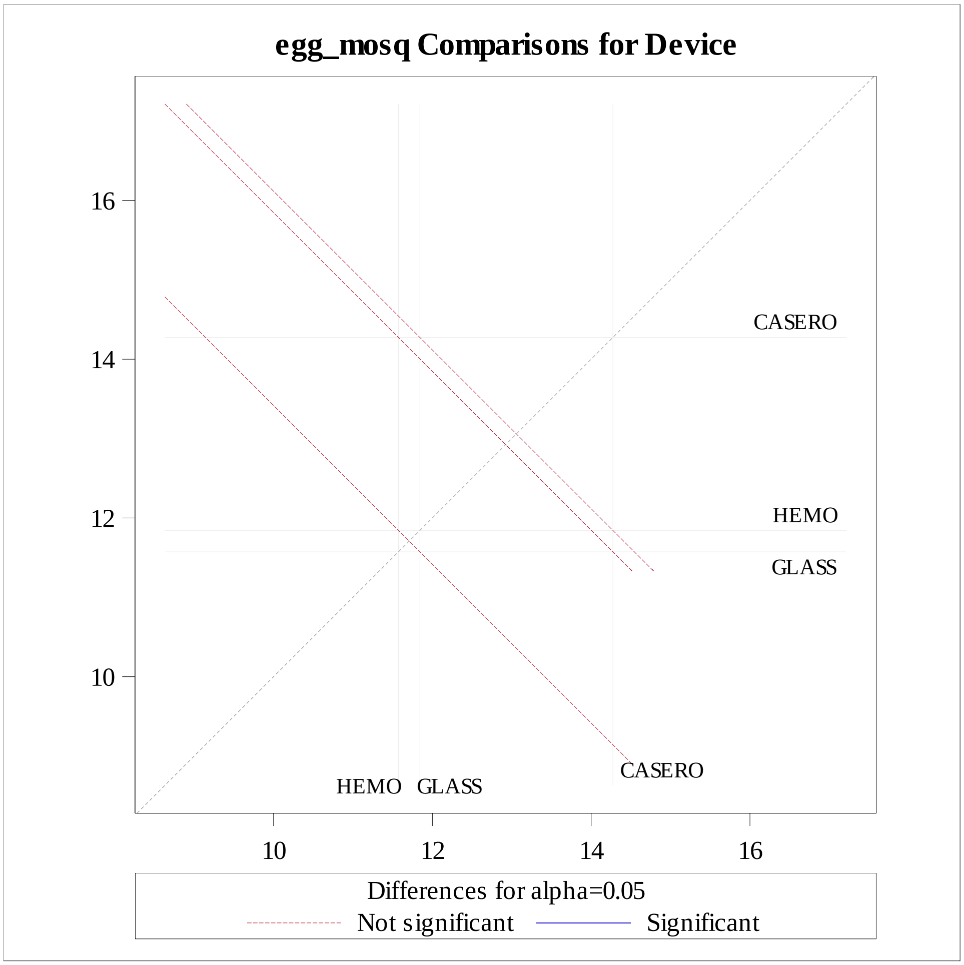

Note: To ensure overall protection level, only probabilities associated with pre-planned comparisons should be used.

The SAS System

The UNIVARIATE Procedure
Variable:
RES

Moments			
**N**	9	**Sum Weights**	9
**Mean**	0	**Sum Observations**	0
**Std Deviation**	2.55041663	**Variance**	6.504625
**Skewness**	0.87992222	**Kurtosis**	0.27216869
**Uncorrected SS**	52.037	**Corrected SS**	52.037
**Coeff Variation**	.	**Std Error Mean**	0.85013888

Basic Statistical Measures			
**Location**		**Variability**	
**Mean**	0.00000	**Std Deviation**	2.55042
**Median**	-0.60333	**Variance**	6.50462
**Mode**	.	**Range**	7.66000
		**Interquartile Range**	3.43000

Tests for Location: Mu0=0				
**Test**	**Statistic**		**p Value**	
**Student's t**	**t**	0	**Pr > |t|**	1.0000
**Sign**	**M**	-0.5	**Pr >= |M|**	1.0000
**Signed Rank**	**S**	-3.5	**Pr >= |S|**	0.7344

Tests for Normality				
**Test**	**Statistic**		**p Value**	
**Shapiro-Wilk**	**W**	0.903636	**Pr < W**	0.2738
**Kolmogorov-Smirnov**	**D**	0.156049	**Pr > D**	>0.1500
**Cramer-von Mises**	**W-Sq**	0.054226	**Pr > W-Sq**	>0.2500
**Anderson-Darling**	**A-Sq**	0.372813	**Pr > A-Sq**	>0.2500

The SAS System

The UNIVARIATE Procedure
Variable: RES

Quantiles (Definition 5)	
**Level**	**Quantile**
**100% Max**	5.016667
**99%**	5.016667
**95%**	5.016667
**90%**	5.016667
**75% Q3**	1.156667
**50% Median**	-0.603333
**25% Q1**	-2.273333
**10%**	-2.643333
**5%**	-2.643333
**1%**	-2.643333
**0% Min**	-2.643333

Extreme Observations			
**Lowest**		**Highest**	
**Value**	**Obs**	**Value**	**Obs**
-2.643333	9	-0.603333	6
-2.373333	7	1.116667	1
-2.273333	3	1.156667	2
-1.423333	4	2.026667	5
-0.603333	6	5.016667	8

The SAS System

The UNIVARIATE Procedure

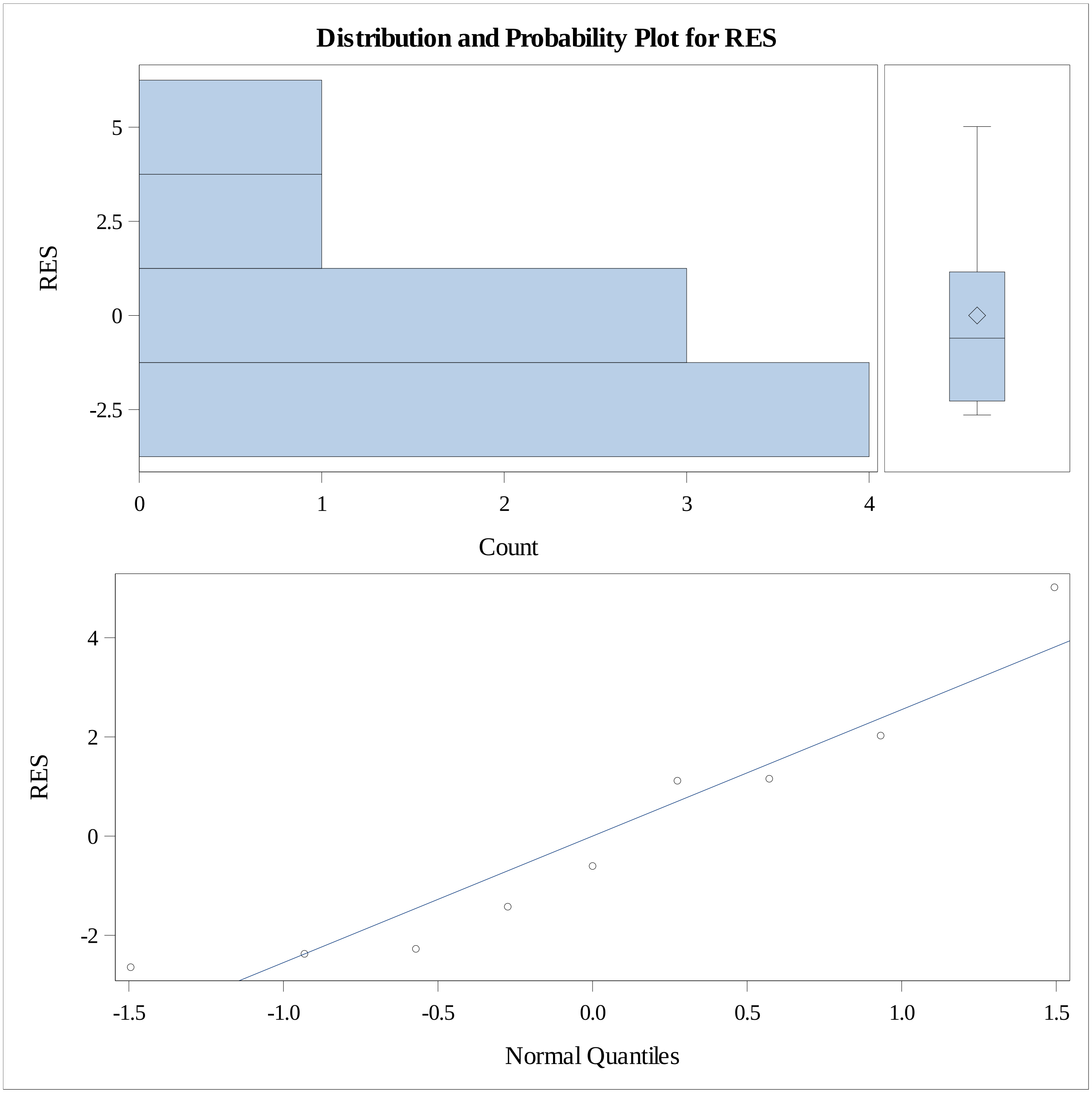

The SAS System

The UNIVARIATE Procedure
Variable: RES

Moments			
**N**	9	**Sum Weights**	9
**Mean**	0	**Sum Observations**	0
**Std Deviation**	2.55041663	**Variance**	6.504625
**Skewness**	0.87992222	**Kurtosis**	0.27216869
**Uncorrected SS**	52.037	**Corrected SS**	52.037
**Coeff Variation**	.	**Std Error Mean**	0.85013888

Basic Statistical Measures			
**Location**		**Variability**	
**Mean**	0.00000	**Std Deviation**	2.55042
**Median**	-0.60333	**Variance**	6.50462
**Mode**	.	**Range**	7.66000
		**Interquartile Range**	3.43000

Tests for Location: Mu0=0				
**Test**	**Statistic**		**p Value**	
**Student's t**	**t**	0	**Pr > |t|**	1.0000
**Sign**	**M**	-0.5	**Pr >= |M|**	1.0000
**Signed Rank**	**S**	-3.5	**Pr >= |S|**	0.7344

Tests for Normality				
**Test**	**Statistic**		**p Value**	
**Shapiro-Wilk**	**W**	0.903636	**Pr < W**	0.2738
**Kolmogorov-Smirnov**	**D**	0.156049	**Pr > D**	>0.1500
**Cramer-von Mises**	**W-Sq**	0.054226	**Pr > W-Sq**	>0.2500
**Anderson-Darling**	**A-Sq**	0.372813	**Pr > A-Sq**	>0.2500

The SAS System

The UNIVARIATE Procedure
Variable: RES

Quantiles (Definition 5)	
**Level**	**Quantile**
**100% Max**	5.016667
**99%**	5.016667
**95%**	5.016667
**90%**	5.016667
**75% Q3**	1.156667
**50% Median**	-0.603333
**25% Q1**	-2.273333
**10%**	-2.643333
**5%**	-2.643333
**1%**	-2.643333
**0% Min**	-2.643333

Extreme Observations			
**Lowest**		**Highest**	
**Value**	**Obs**	**Value**	**Obs**
-2.643333	9	-0.603333	6
-2.373333	7	1.116667	1
-2.273333	3	1.156667	2
-1.423333	4	2.026667	5
-0.603333	6	5.016667	8

The SAS System

The UNIVARIATE Procedure

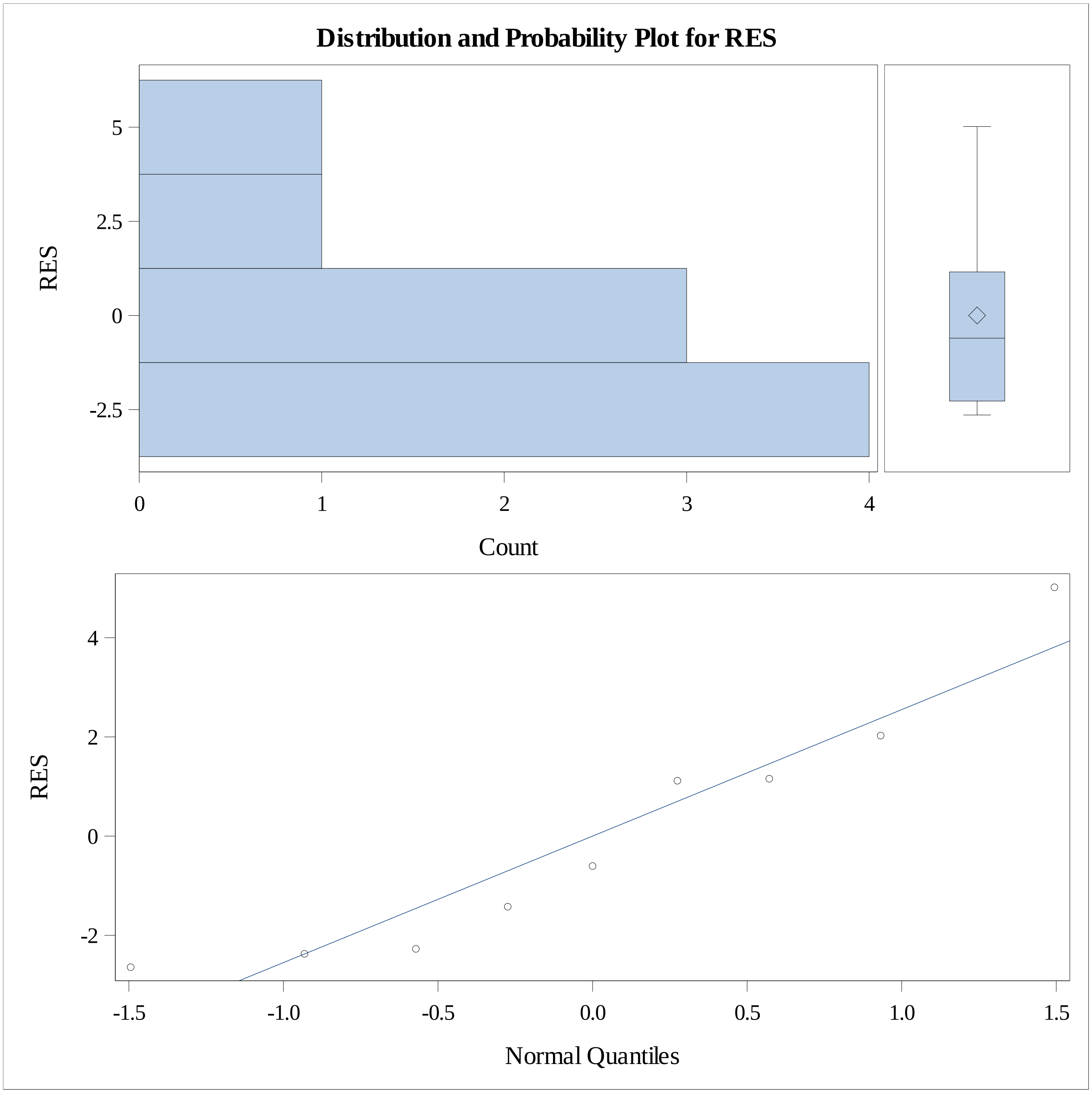

The SAS System

The MEANS Procedure

Analysis Variable : egg_mosq egg_mosq						
**Device**	**N Obs**	**N**	**Mean**	**Std Dev**	**Minimum**	**Maximum**
CASERO	3	3	14.2733333	4.3466577	11.6300000	19.2900000
GLASS	3	3	11.5733333	1.9688660	9.3000000	12.7300000
HEMO	3	3	11.8433333	1.8023966	10.4200000	13.8700000
