# Supplementary material for: Evaluation of “Caserotek” a low cost and effective artificial blood-feeding device for mosquitoes
Source: PLoS Negl Trop Dis. 2023 Aug 25;17(8):e0011563. doi: 10.1371/journal.pntd.0011563 (PMC10484425; doi:10.1371/journal.pntd.0011563)
Supplement: Attachment — Submitted filename: Reviewer.docx [file pntd.0011563.s012.docx]

Reviewer's Responses to Questions

Reviewer #1: In this submission, the authors describe and evaluate a low-tech, affordable blood-feeding device that they developed and have been using in an operational setting for several years already. The device, Caserotek, is constructed of common off-the-shelf products and can be run with minimal infrastructure requirements making the device desirable for low-resource settings. The authors conducted a series of experiments to benchmark the Caserotek against two current gold-standard blood-feeding devices. Overall, the Caserotek performed very well providing evidence that this device could provide a robust alternative to the more resource intensive existing options. The experiments are simple and robust, the analysis is appropriate, and the paper is well written.

Minor comments:

Spanish spelling of ‘mosquitoes’ in a number of locations. Need to check and use English spelling for this journal.

Response: Done.

The small sample sizes per replicate resulting from removal of unfed females in earlier feedings makes it difficult to interpret later feedings in exp 1. I appreciate that the authors reveal and acknowledge this issue in the results description. Since the analysis is based on rates and percentages, small sample sizes limit resolution of the variability. I suggest removing any data points based on less than 10 females.

Response: We have followed the reviewer’s advice and rerun our models eliminating any replicates with less than 10 females for Experiment #1 where this was a issue. We now provide all model results for models with replicates with less than 10 females eliminates as well as a model where all data is included for comparison. Conclusions and qualitative results are not very different. It was error not to upload our model results as had been our intention. We also provide our SAS code so readers can attempt to duplicate our results.

Figure 3: Please make the points larger and consider removing the black border for points and lines. As is, it is very difficult to see the colors for the points, and the color of the lines is obscured by the border.

Response: Done, also we now only include data with replicates based on 10 or more females.

The authors make reference to availability of the full model results, but I could not find the model output in the main doc or supplementary files.

Response: This was an oversite on our part, we now have an extensive supplementary text file with all model results.

Reviewer #2: Authors are suggested to provide information on how much blood (or the minimum amount of blood to be used) to be filled into their device for each feeding, and how long blood can be maintained at 37 °C during the feeding. It would also be helpful to provide a standard operation protocol which can result in over 70% blood feeding rate when using their new device, including the information on mosquito age and feeding time et al. It is unclear how this device can be scaled up to large production facilities, which needs to be discussed.

Response: we now indicate the device requires 2ml of blood (Line 184) and provide an SOP used by the NAMRU-6 laboratory in supplementary information. Finally, we have added a paragraph Lines 372-379 discussing the scalability of Caserotek for mass-rearing and for use in vector competence studies.

Line 287, Survival curves should be provided, with an appropriate statistical analysis, to support their conclusion.

Response: We now provide a supplementary figure showing mortality for Experiment #1 and the full model results are presented in the supplementary text. We apologize for not having the model results available for the initial review, this was an oversight and would have probably addressed concerns. Our GLM analysis is appropriate to measure overall survival associated with mosquitoes who fed on blood when exposed.

Line 196, The manufacture and dimension of glass membrane feeders should be provided.

Response: The glass membrane feeders were custom made locally in Peru by a local Glass blower (Jesus Ramirez Toribio, Glass blower technician, Servicio Tecnico Ramirez, Fabricacion y Reparacion de equipos y materiales de vidrio para laboratorio. They were made in 2014 and we are not sure of his status or his business, but we can provide an email: [tjesusramirez9@gmail.com](mailto:tjesusramirez9@gmail.com). We now provide the diameter of the glass feeder in the text.

Line 212, when oviposition sites are provided to mosquitoes?

Response: done (now line 213)
